# Highly selective Diels–Alder and Heck arylation reactions in a divergent synthesis of isoindolo- and pyrrolo-fused polycyclic indoles from 2-formylpyrrole

**DOI:** 10.3762/bjoc.16.113

**Published:** 2020-06-17

**Authors:** Carlos H Escalante, Eder I Martínez-Mora, Carlos Espinoza-Hicks, Alejandro A Camacho-Dávila, Fernando R Ramos-Morales, Francisco Delgado, Joaquín Tamariz

**Affiliations:** 1 Departamento de Química Orgánica, Escuela Nacional de Ciencias Biológicas, Instituto Politécnico Nacional, Prolongación de Carpio y Plan de Ayala S/N, 11340 Mexico City, Mexico; 2 Departamento de Química Orgánica, Facultad de Ciencias Químicas, Universidad Autónoma de Coahuila, Blvd. Venustiano Carranza e Ing. J. Cárdenas S/N, 25280 Saltillo, Coah., Mexico; 3 Departamento de Química Orgánica, Facultad de Ciencias Químicas, Universidad Autónoma de Chihuahua, Circuito Universitario S/N, 31125 Chihuahua, Chih., Mexico; 4 Unidad de Servicios de Apoyo en Resolución Analítica, Universidad Veracruzana, Luis Castelazo Ayala S/N, 91190 Xalapa, Ver., Mexico

**Keywords:** *endo-*Diels–Alder stereocontrol, 2-formylpyrrole, intramolecular Heck arylation reaction, non-covalent interactions, pyrroloindoles, pyrroloisoindoles

## Abstract

A highly regio-, chemo- and stereoselective divergent synthesis of isoindolo- and pyrrolo-fused polycyclic indoles is herein described, starting from 2-formylpyrrole and employing Diels–Alder and Heck arylation reactions. 3-(*N-*Benzyl-2-pyrrolyl)acrylates and 4-(pyrrol-2-yl)butenones underwent a highly *endo-*Diels–Alder cycloaddition with maleimides to furnish octahydropyrrolo[3,4-*e*]indoles, which served as precursors in the regioselective synthesis of aza-polycyclic skeletons via an intramolecular Heck arylation reaction. Through the latter reaction, the 3-(*N-*benzyl-2-pyrrolyl)acrylates give rise to 3-(pyrrolo[2,1-*a*]isoindol-3-yl)acrylates. A further oxidative aromatization of the polycyclic intermediates provides the corresponding polycyclic pyrrolo-isoindoles and isoindolo-pyrrolo-indoles. A theoretical study on the stereoselective Diels–Alder reactions, carried out by calculating the *endo/exo* transition states, revealed the assistance of non-covalent interactions in governing the *endo* stereocontrol.

## Introduction

**Figure 1:**
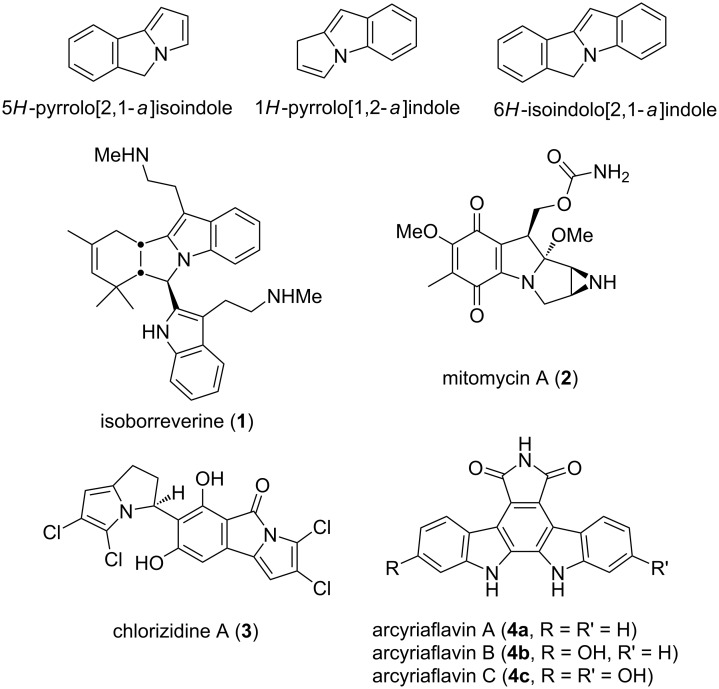
Fused aza-hetero polycyclic frames and natural pyrrolizine- and isoindole-containing alkaloids.

Pyrrolizines [1–2] and pyrrolizidines [3–4], abundant in nature [4–5], are among the simplest pyrrole-fused heterocyclic aza-bridged compounds. The numerous series of pyrrolizine-containing polycycles include pyrrolo[2,1-*a*]isoindoles [6], pyrrolo[1,2-*a*]indoles [7–10] and isoindolo[2,1-*a*]indoles [11] (Figure 1). These three series constitute the core of natural occurring alkaloids that are known to have varied and robust pharmacological activity, such as isoborreverine (**1**) [12], mitomycin A (**2**) [13] and chlorizidine A (**3**) [14]. The indole moiety is incorporated into their structure, which is important because it is a seminal heterocycle that exhibits a wide range of biological activity [15–16] and great diversity as building block [17–18].

Among the tetracyclic scaffolds, isoindolo[2,1-*a*]indoles have attracted a particular interest from the synthetic point of view, either as the target or the motif for designing novel annulation methodologies [19–21]. Indeed, isoindole-based heterocycles represent a varied and ubiquitous scaffold found in a large number of natural and synthetic bioactive compounds [22–25], such as isoindolocarbazoles (e.g., arcyriaflavins A–C (**4a**–**c**)) [26–27].

**Scheme 1:**
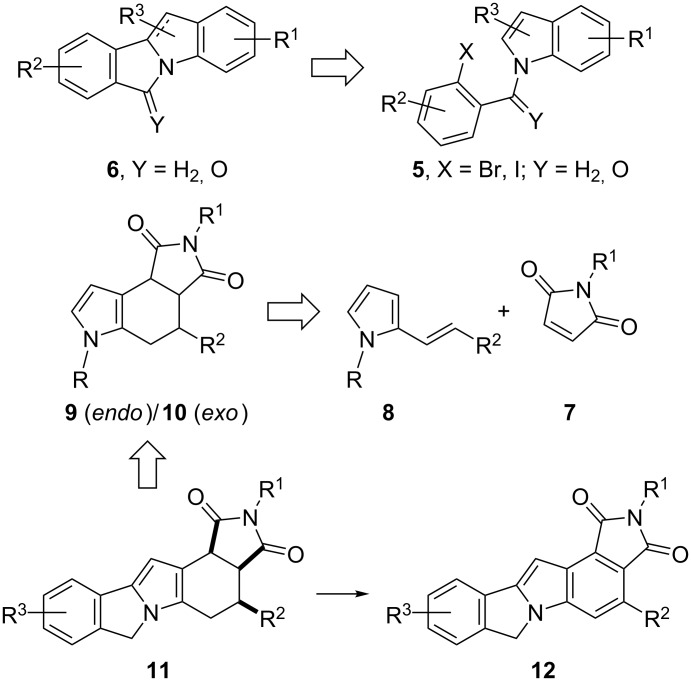
Synthetic approaches for the preparation of pyrrolo-fused aza-hetero polycyclic frames.

The challenge of the synthesis of this series of aza-heterocycles is associated with the complexity of its structures [11,19–27]. The intramolecular Heck arylation coupling reaction is among the most useful strategies for the construction of these compounds (Scheme 1) [11,21]. For example, isoindolo[2,1-*a*]indoles **6** have been prepared by the annulation process through a Pd- or Ni-catalyzed coupling starting from the *N-*functionalized indoles **5** [11,19,21,28–30]. On the other hand, the formation of pyrrolo[3,4-*e*]indoles **9**/**10** can be achieved via a Diels–Alder cyloaddition of 2-vinylpyrroles **8** with maleimides **7** [31–35]. This is a representative example of the less common approach for the construction of the indole skeleton on substituted pyrroles by generating the benzene ring via an annulation process [36–39].

Due to our interest in transforming simple five-membered heterocycles into natural products [40–41] and complex aza-polycyclic structures [42], we herein combined both approaches shown in Scheme 1 to design the construction of pentacycles **11**, after carrying out an uncommon but highly diastereoselective Diels–Alder cycloaddition followed by a coupling reaction. This route also allowed out the aromatization of the B ring to provide compounds **12**. To account for the strong *endo* preference of adducts **9**, a computational study was performed to analyze the stationary points on the potential surfaces of the Diels–Alder reactions.

## Results and Discussion

### Synthesis

**Scheme 2:**
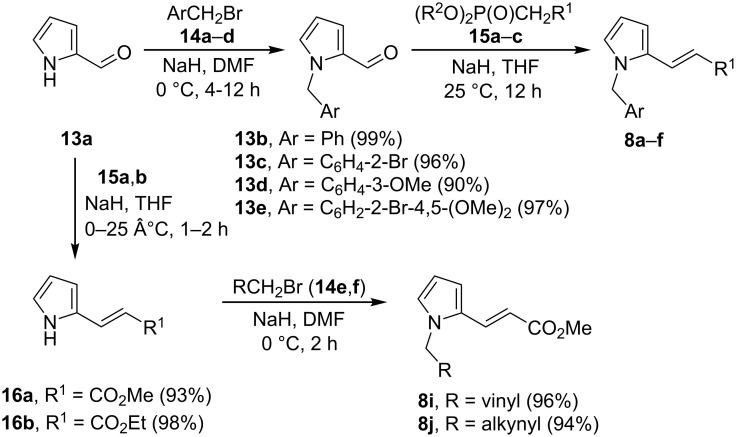
Preparation of 1,2-substituted pyrroles **8a**–**f** and **8i**,**j**.

Pyrroles **8a**–**j** were prepared through a straightforward and efficient route based on the functionalization of 2-formylpyrrole (**13a**), using recently described methodologies [41]. Firstly, **13a** was reacted with the series of benzyl bromides **14a**–**d** to produce adequately *N-*substituted five-membered aza-units **13b**–**e**, respectively (Scheme 2), which were treated with the phosphonates **15a**–**c** to afford derivatives **8a**–**f** in high yields (Table 1, entries 1–6). For the synthesis of pyrroles **8g**,**h** (R^1^ = Ac), pyrroles **13c** and **13e** were subjected to condensation reactions at a higher temperature, with acetone as the nucleophile (instead the phosphonate **15**) in the presence of KOH as the base (Table 1, entries 7 and 8). The second methodology consisted of an inverted sequence of reactions in relation to the previous approach, starting from the treatment of **13a** with **15a**,**b** to give 3-(pyrrol-2-yl)acrylates **16a**–**b** (Scheme 2). The latter compounds were alkylated with allyl bromide (**14e**) and propargyl bromide (**14f**) to generate pyrroles **8i**,**j**, respectively, in high yields.

**Table 1:** Preparation of 1,2-substituted pyrroles **8a**–**h**.^a^

entry	**13**	**15**	*T* (°C)	*t* (h)	R^1^	Ar	**8** (%)^b^

1	**13b**	**15b**	25	12	CO_2_Et	Ph	**8a** (98)
2	**13b**	**15c**	25	12	CN	Ph	**8b** (99)^c^
3	**13c**	**15a**	25	12	CO_2_Me	C_6_H_4_-2-Br	**8c** (99)
4	**13c**	**15b**	25	12	CO_2_Et	C_6_H_4_-2-Br	**8d (**93)
5	**13d**	**15b**	25	12	CO_2_Et	C_6_H_4_-3-OMe	**8e** (90)
6	**13e**	**15a**	25	12	CO_2_Me	C_6_H_2_-2-Br-4,5-(OMe)_2_	**8f** (98)
7	**13c**	^d^	100	4	COMe	C_6_H_4_-2-Br	**8g** (81)
8	**13e**	^d^	100	4	COMe	C_6_H_2_-2-Br-4,5-(OMe)_2_	**8h** (72)

^a^Reagents and conditions: **13** (1.0 mol equiv), **15** (1.2 mol equiv) and NaH (1.5 mol equiv). ^b^After purification by column chromatography. ^c^As an *E*/*Z* (95:5) mixture. ^d^The reaction was carried out with acetone (1.3 mol equiv) and KOH (1.5 mol equiv) in MeOH.

**Scheme 3:**
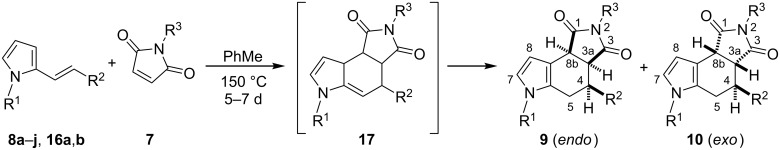
Diels–Alder cycloadditions of pyrroles **8a**–**j** and **16a**–**b** with maleimides **7b**–**c**.

The synthesis of octahydropyrrolo[3,4-*e*]indoles **9**/**10** was achieved through a Diels–Alder reaction between the 2-vinylpyrroles **8a**–**j** and **16a**,**b** and maleimides **7a**–**c** under thermal conditions (Scheme 3). N-Unsubstituted 2-vinylpyrroles **16a**,**b** were chosen as the dienes to evaluate the reactivity with diverse dienophiles. The reaction of acrolein, methyl acrylate, maleic anhydride and maleimide (R^3^ = H, **7a**) in xylene by heating up to 150 °C failed to provide the corresponding adducts. It was possible to recover the starting materials, which were accompanied by a decomposition residue. In contrast, maleimides **7b** and **7c** reacted efficiently to furnish the respective *endo/exo* adducts **9a**–**d**/**10a**–**d** in good to high *endo* diastereoselectivity and yields (Table 2, entries 1–4). The cycloaddition with **7c** led to the best selectivity (vide infra).

**Table 2:** Diels–Alder reactions of 2-vinylpyrroles **8a**–**j** and **16a**,**b** with maleimides **7b**,**c**.^a^

entry	diene	**7**	R^1^	R^2^	R^3^	**9**/**10** (ratio)^b^ (%)^c^

1	**16a**	**7b**	H	CO_2_Me	Me	**9a**/**10a** (70:30) (72)
2	**16b**	**7b**	H	CO_2_Et	Me	**9b**/**10b** (75:25) (74)
3	**16a**	**7c**	H	CO_2_Me	Ph	**9c**/**10c** (94:6) (73)
4	**16b**	**7c**	H	CO_2_Et	Ph	**9d**/**10d** (97:3) (71)^d^
5	**8a**	**7c**	Bn	CO_2_Et	Ph	**9e**/**10e** (95:5) (95)
6	**8b**	**7c**	Bn	CN	Ph	**9f**/**10f** (76:24) (94)
7	**8c**	**7b**	CH_2_C_6_H_4_-2-Br	CO_2_Me	Me	**9g**/**10g** (99:1) (94)^d^
8	**8c**	**7c**	CH_2_C_6_H_4_-2-Br	CO_2_Me	Ph	**9h**/**10h** (99:1) (98)^d^
9	**8d**	**7b**	CH_2_C_6_H_4_-2-Br	CO_2_Et	Me	**9i**/**10i** (99:1) (97)^d^
10	**8d**	**7c**	CH_2_C_6_H_4_-2-Br	CO_2_Et	Ph	**9j**/**10j** (97:3) (96)
11	**8e**	**7c**	CH_2_C_6_H_4_-3-OMe	CO_2_Et	Ph	**9k**/**10k** (98:2) (97)
12	**8f**	**7c**	CH_2_C_6_H_2_-2-Br-4,5-(OMe)_2_	CO_2_Me	Ph	**9l**/**10l** (93:7) (95)
13	**8g**	**7c**	CH_2_C_6_H_4_-2-Br	COMe	Ph	**9m**/**10m** (91:9) (94)
14	**8h**	**7c**	CH_2_C_6_H_2_-2-Br-4,5-(OMe)_2_	COMe	Ph	**9n**/**10n** (90:10) (93)
15	**8i**	**7c**	allyl	CO_2_Me	Ph	**9o**/**10o** (98:2) (92)^d^
16	**8j**	**7c**	propargyl	CO_2_Me	Ph	**9p**/**10p** (99:1) (95)^d^

^a^Reagents: **8** and **16** (1.0 mol equiv) and **7** (1.2 mol equiv). ^b^Calculated by ^1^H NMR from the crude reaction mixtures. ^c^After purification by column chromatography and as the addition of the yields of both isomers. ^d^Following purification by column chromatography and as the yield of the major isomer.

**Figure 2:**
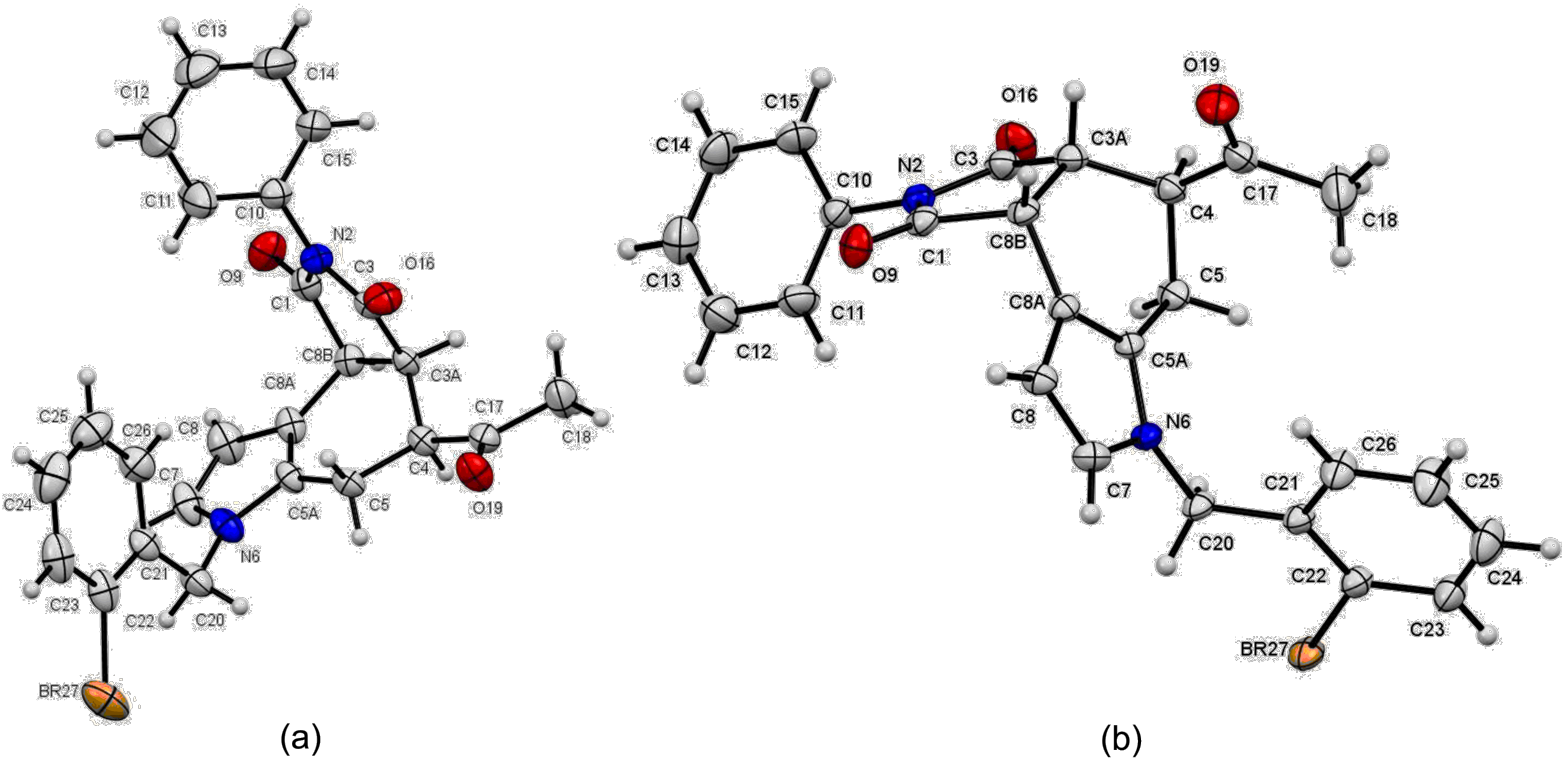
Structures of **9m** (a) and **10m** (b) as determined by single-crystal X-ray diffraction crystallography (ellipsoids at the 30% probability level).

The structure of the *endo* and *exo* diastereoisomers was established by ROESY experiments. In the *endo* isomer **9m**, for example, the irradiation of the signal of proton H-4 enhanced the signals of protons H-3a and H-8b, indicating that the three protons are on the same side. In the case of the *exo* isomer **10m**, the irradiation of the singlet of the methyl group of the C-4 acetyl group increased the size of the signals of the protons H-3a and H-8b, which are located on the same side as the acetyl group. The unambiguous assignment of their structures was accomplished by a single-crystal X-ray diffraction crystallography of both *endo* **9m** and *exo* **10m** adducts (Figure 2) [43].

In addition, the thermal cycloadditions of 2-vinylpyrroles **8a**–**j** and maleimides **7b**,**c** took place satisfactorily to deliver the expected adducts **9e**–**p**/**10e**–**p**, also with high *endo* diastereoselectivity and good yields (Table 2, entries 5–16). None of the series of mixtures **9a**–**p**/**10a**–**p** corresponds to the direct products of the Diels–Alder cycloadditions, since they are derivatives resulting from the double bond isomerization of adducts **17** (i.e., migration from the pyrrole-fused *exo* cyclic position to the *endo* cyclic aromatic tetrahydroindole skeleton) [32]. Any attempt to isolate or detect **17** during the trials turned out to be unsuccessful [35]. It is noteworthy that there have been almost no reports to date on the uncommon highly diastereoselective Diels–Alder reactions of analogous 2-vinyl or 3-vinylpyrroles [32], nor on the reactivity of 2-vinylpyrroles substituted by electron-withdrawing groups [34–35].

Interestingly, all the 2-vinylpyrroles, including the N-unsubstituted **16a**,**b** or the N-substituted ones **8a** and **8c**–**j**, furnished a high *endo* stereoselectivity with dienophile **7c**, except the *N-*substituted 2-acrylonitrile pyrrole **8b**. Accordingly, the R^2^ substituent in the diene would play an important role in directing the *endo/exo* orientation of the approach to the maleimide at the transition state (TS). Therefore, the *endo* diastereoselectivity is apparently not controlled by the presence or the absence of the substituent bonded to the nitrogen atom of the heterocycle. Since it is not clear what factors favor this relevant selectivity, the geometry and energy of the TSs were calculated for some of the diene–dienophile pairs depicted in Table 2 (vide infra).

### Cyclization via an intramolecular Heck arylation reaction

**Scheme 4:**
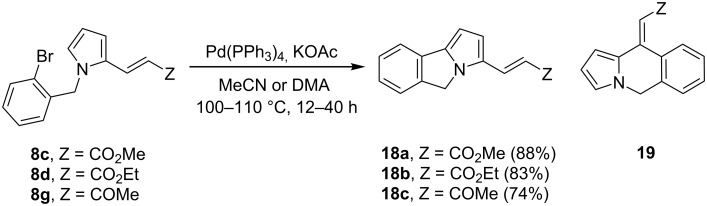
Pd(0)-catalyzed intramolecular Heck cross-coupling reaction of 2-vinylpyrroles **8c**,**d** and **8g**.

Before attempting the intramolecular Heck cross-coupling reaction of the octahydroindoles **9**/**10** to the pentacycles **11**, the process was explored with the simple substrates **8c**,**d** and **8g** (Scheme 4). Thus, the Pd(0)-catalyzed cyclization of the latter vinylpyrroles with potassium acetate and using acetonitrile or dimethylacetamide (DMA) as the solvent at 100 °C afforded pyrrolo[2,1-*a*]isoindoles **18a**–**c** in good yields. This annulation process was regioselective, showing a preference of the cross-coupling reaction with the C-5 pyrrolic position and not with the vinyl moiety, which would give the dihydropyrrolo[1,2-*b*]isoquinoline **19**. A similar chemoselectivity has been previously observed, being explained as a consequence of the acidity of the C–H bond being cleaved [6,44].

**Scheme 5:**
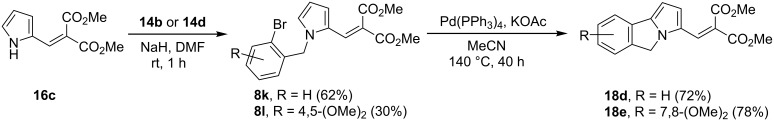
Synthesis of 2-vinylpyrroles **8k**,**l** and their Pd(0)-catalyzed intramolecular Heck cross-coupling to pyrrolo[2,1-*a*]isoindoles **18d**,**e**.

Additional intramolecular cross-coupling reactions were carried out by utilizing dimethyl malonates **8k** and **8l**, which were prepared through the N-benzylation of **16c** with benzyl bromides **14b** and **14d**, respectively (Scheme 5). Pyrrole **16c** was synthesized by Knoevenagel reaction of **13a** with dimethyl malonate [42]. The Pd(0)-catalyzed cyclization of **8k** and **8l** required a temperature of 140 °C, but provided pyrrolo[2,1-*a*]isoindoles **18d** and **18e**, respectively, in good yields.

Once having the optimal reaction conditions for the palladium(0)-catalyzed cross-coupling reaction leading to the cyclization of *N-*(2-bromobenzyl)pyrroles **8**, the *endo-*octahydropyrrolo[3,4-*e*]indole-1,3-diones **9g**–**j** and **9m** were converted into pentacycles **11a**–**e** through the same methodology (Table 3). Optimized reaction conditions were established by using **9j** as the model substrate and changing the catalyst, base and solvent. Palladium acetate was not efficient for the conversion, even though the base was modified (Table 3, entries 1 and 2). Pd(PPh_3_)_4_ was a better catalyst when employing potassium acetate as the base, and MeCN was more efficient than DMF, affording **11a** in a better yield (Table 3, entries 3 and 4). The cyclization of **9g**–**i** and **9m** was performed under similar conditions, with MeCN as the solvent, to produce the aza-pentacycles **11b**–**e**, respectively, in modest to good yields (Table 3, entries 5–8).

**Table 3:** Heck cross-coupling reactions of **9g**–**j** and **9m** for the preparation of pentacycles **11a**–**e**.^a^

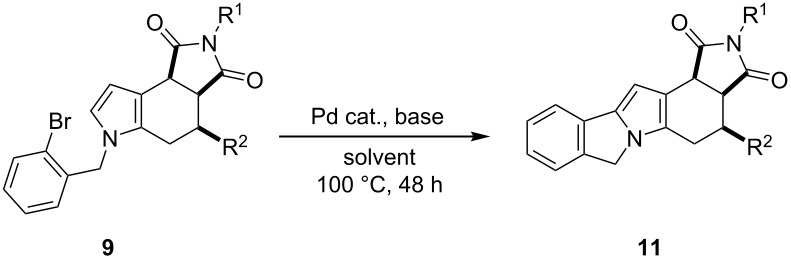							

entry	**9**	R^1^	R^2^	catalyst	base	solvent	**11** (%)^b^

1	**9j**	Ph	CO_2_Et	Pd(OAc)_2_	K_2_CO_3_	DMF	^c^
2	**9j**	Ph	CO_2_Et	Pd(OAc)_2_	Cs_2_CO_3_	DMF	^c^
3	**9j**	Ph	CO_2_Et	Pd(PPh_3_)_4_	KOAc	DMF	**11a** (53)
4	**9j**	Ph	CO_2_Et	Pd(PPh_3_)_4_	KOAc	MeCN	**11a** (75)
5	**9h**	Ph	CO_2_Me	Pd(PPh_3_)_4_	KOAc	MeCN	**11b** (77)
6	**9i**	Me	CO_2_Et	Pd(PPh_3_)_4_	KOAc	MeCN	**11c** (81)
7	**9m**	Ph	COMe	Pd(PPh_3_)_4_	KOAc	MeCN	**11d** (48)
8	**9g**	Me	CO_2_Me	Pd(PPh_3_)_4_	KOAc	MeCN	**11e** (86)

^a^Reagents: **9** (1.0 mol equiv), catalyst (0.2 mol equiv) and base (2.0 mol equiv). ^b^After purification by column chromatography. ^c^No reaction and **9j** was recovered.

**Scheme 6:**
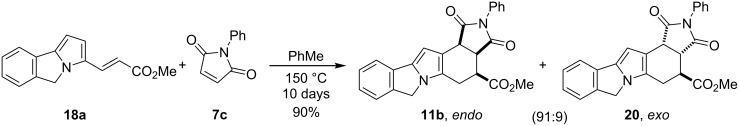
Diastereoselective Diels–Alder reaction of pyrrolo[2,1-*a*]isoindole **18a** with **7c**.

An alternative approach for the synthesis of pentacycles **11** would be the Diels–Alder cycloaddition of pyrrolo[2,1-*a*]isoindoles **18** with maleimides **7**. Indeed, the reaction between **18a** and **7c** successfully proceeded to give a diastereoisomeric mixture of adducts **11b**/**20** (91:9) in high yield (Scheme 6). Although the reactivity was much lower (10 days) compared to the cycloadditions with 2-vinylpyrroles **8a**–**h** (7 days) (Table 2), the diastereoselectivity remained rather high and similar to derivatives **8g** and **8h**.

Considering the significant biological activity of indoles, octahydropyrrolo[3,4-*e*]indole-1,3-diones **9** were submitted to aromatization to generate the corresponding tetrahydropyrrolo[3,4-*e*]indole-1,3-diones **21** (Table 4). When **9e** was treated with manganese oxide [32] at 25 or 100 °C for 24 h, no reaction occurred and the starting material was recovered (Table 4, entries 1 and 2). However, the use of 2,3-dichloro-5,6-dicyano-1,4-benzoquinone (DDQ) at 25 °C for 48 h led to the aromatized compound **21a** in high yield (Table 4, entry 3). Under similar reaction conditions, the series of pyrrolo[3,4-*e*]indole-1,3-diones **21b**–**g** was resulted in high yields (Table 4, entries 4 and 6–10). Derivative **21c** was also prepared by the oxidation of **9h** with manganese oxide at higher temperature, but the yield was lower (Table 4, entry 5).

**Table 4:** Synthesis of tetrahydropyrrolo[3,4-*e*]indole-1,3-diones **21a**–**g** by aromatization of **9e**–**f, 9h**–**k** and **9m**.^a^

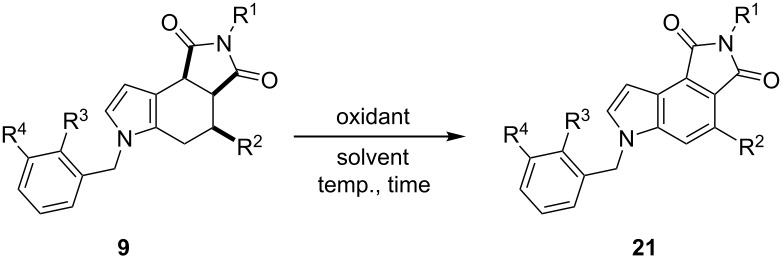										

entry	**9**	R^1^	R^2^	R^3^	R^4^	oxidant	solvent	*T* (°C)	*t* (h)	**21** (%)^b^

1	**9e**	Ph	CO_2_Et	H	H	MnO_2_	CH_2_Cl_2_	25	24	(c)
2	**9e**	Ph	CO_2_Et	H	H	MnO_2_	PhMe	100	24	(c)
3	**9e**	Ph	CO_2_Et	H	H	DDQ	CH_2_Cl_2_	25	48	**21a** (88)
4	**9f**	Ph	CN	H	H	DDQ	CH_2_Cl_2_	25	48	**21b** (94)
5	**9h**	Ph	CO_2_Me	Br	H	MnO_2_	PhMe	140	24	**21c** (69)
6	**9h**	Ph	CO_2_Me	Br	H	DDQ	CH_2_Cl_2_	25	48	**21c** (90)
7	**9i**	Me	CO_2_Et	Br	H	DDQ	CH_2_Cl_2_	25	48	**21d** (86)
8	**9j**	Ph	CO_2_Et	Br	H	DDQ	CH_2_Cl_2_	25	48	**21e** (89)
9	**9k**	Ph	CO_2_Et	H	OMe	DDQ	CH_2_Cl_2_	25	48	**21f** (90)
10	**9m**	Ph	COMe	Br	H	DDQ	CH_2_Cl_2_	25	48	**21g** (90)

^a^Reagents: **9** (1.0 mol equiv), MnO_2_ (4.0 mol equiv) and DDQ (2.5 mol equiv). ^b^Following purification by column chromatography. ^c^Substrate **9e** was recovered.

As part of our interest in further functionalizing octahydropyrrolo[3,4-*e*]indole-1,3-diones **9**, formylation was herein investigated under usual Vilsmeier–Haack reaction conditions (Table 5). Fortunately, the series of 7-formyl derivatives **22a**–**e** was attained in good yields. This synthetic approach allows for the preparation of widely polysubstituted octahydropyrrolo[3,4-*e*]indole-1,3-dione derivatives. However, the conversion into their aromatic skeleton either by using manganese oxide or DDQ as the oxidizing reagents, or even including Pd/C at high temperatures (250 °C) [45], failed to obtain the series of indoles **23**. It is likely that the electron withdrawing effect of the formyl group at the C-7 position counterbalance the delocalization direction of the electronic density provided by the nitrogen lone-pair, which plausibly stabilizes the cationic or radical species formed by the oxidant reagent during the aromatization process [46], as efficiently occurred with derivatives **9**.

**Table 5:** Preparation of derivatives **22a**–**e** by formylation of octahydropyrrolo[3,4-*e*]indole-1,3-diones **9**.^a^

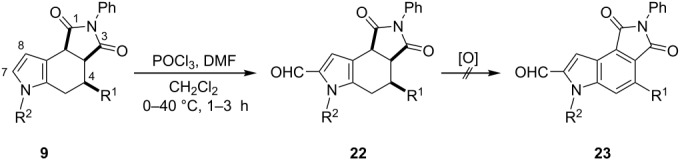						

entry	**9**	R^1^	R^2^	*T* (°C)	*t* (h)	**22** (%)^b^

1	**9f**	CN	Bn	40	1	**22a** (95)
2	**9h**	CO_2_Me	CH_2_C_6_H_4_-2-Br	0	3	**22b** (88)
3	**9l**	CO_2_Me	CH_2_C_6_H_2_-2-Br-4,5-(OMe)_2_	0	2	**22c** (89)
4	**9o**	CO_2_Me	allyl	25	2	**22d** (84)
5	**9p**	CO_2_Me	propargyl	25	2	**22e** (80)

^a^Reagents: **9** (1.0 mol equiv), POCl_3_ (1.2 mol equiv) and DMF (1.2 mol equiv). ^b^After purification by column chromatography.

**Scheme 7:**
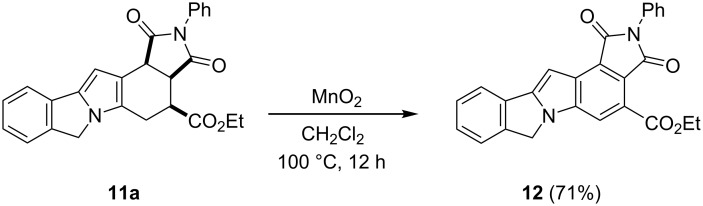
Synthetic approach to the fused aza-heterocyclic pentacycle **12**.

Finally, in order to obtain the aromatic indole-based pentacycles **12**, the aromatization of pentacycles **11** was explored. Although the use of DDQ under the oxidative reaction conditions shown in Table 4 was efficient for the preparation of derivatives **21**, the conversion of **11a** into **12** was unsuccessful (Scheme 7). The action of active MnO_2_ in toluene at high temperature was able to promote the aromatization of **11a** affording pentacycle **12**, in low yield (30%) [47]. Moreover, when the oxidation was carried out in methylene chloride, pentacycle **12** was delivered in a higher yield (71%). The synthesis of analogs to the latter compound has attracted much attention in recent years [21].

### Theoretical calculations

The Diels–Alder reaction of vinylpyrroles **8b**, **8c** and **8g** with maleimides **7b** and **7c** resulted in a highly diastereoselective cycloaddition leading to the mixture of *endo/exo* cycloadducts **9**/**10**, where the *endo* product **9** was the major one (Scheme 3 and Table 2). Considering the synthetic and biological potential value of the resulting products in the construction of a series of pyrrolo- and isoindolo-fused polycyclic indoles and octahydroindoles, a quantum theoretical study of the TSs was conducted to gain insight into the main factors that control this *endo* diastereoselectivity.

**Figure 3:**
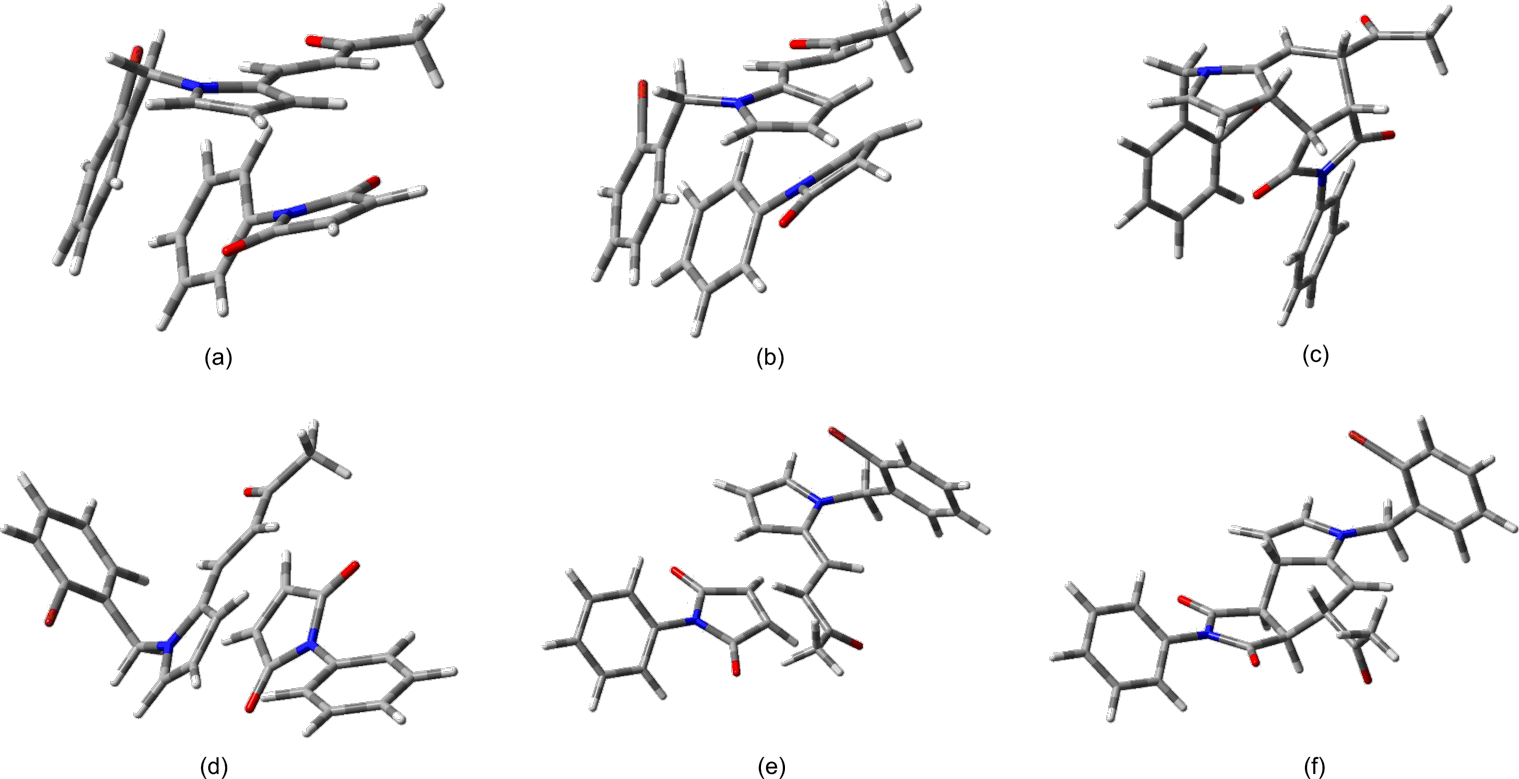
M06-2X/6-31+G(d,p) Optimized geometry for each of the SCs (a and d), TSs (b and e) and ADs (c and f) of the Diels–Alder reactions of diene **8g** and dienophile **7c**, for the *endo* (a–c) and the *exo* (d–f) approaches, respectively.

Firstly, the geometry and energy of the TSs and the associated minima along the reaction coordinates of the N-substituted diene **8g** with maleimide **7c** were calculated at the M06-2X/6-31+G(d,p) level of theory [48–51] on the Gaussian 09 program [52]. For each stationary point of the Diels–Alder cycloadditions, the relative energy is summarized in Table 6 and the geometry is displayed in Figure 3. The data indicate a single concerted TS not only for the cycloaddition of **8g**/**7c**, but also for all the *endo* and *exo* processes (Supporting Information File 1, Appendix 5). The Gibbs energy was also calculated for each reaction and in most cases showed a good correlation with the ZPE-corrected energy (Table 6). All the *endo* approaches were lower in energy than the *exo* approaches, reaching the TSs from the supramolecular complexes (SCs) [53]. For most of the cycloadditions, the difference in energy of the TSs were large enough (>3 kcal/mol) to find an *endo/exo* diastereoisomeric ratio greater than 99:1, which is in agreement with the observed experimental results.

**Table 6:** Calculated [M06-2X/6-31+G(d,p)] relative ZPE-corrected energy (kcal/mol) of the supramolecular complexes (SCs), TSs and adducts (ADs) located on the potential surfaces for the Diels–Alder reactions of the dienes **8b**, **8c**, **8g**, **8j**, **16a** and **18a**, and dienophiles **7b**,**c**.^a^

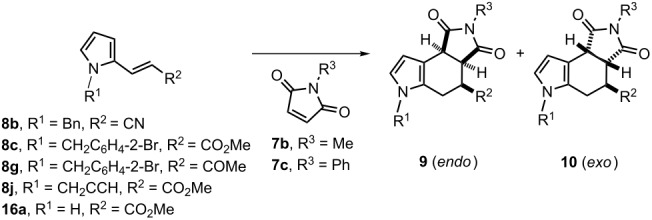					

cycloaddends	SC^b^	TS^b^	AD^b^	diff^c^	**9**/**10** or **11b**/**20** (%)^d^ (%)^e^ (%)^f^

**8b**/**7c**-*endo*	0.00 (0.00)	26.92 (29.72)	−9.76 (−6.47)	0.00 (0.00)	**9f** (99.4) (79.9) (76)
**8b**/**7c**-*exo*	0.76 (0.65)	29.99 (30.53)	−7.94 (−7.31)	3.07 (0.82)	**10f** (0.6) (20.1) (24)
**8c**/**7b**-*endo*	0.00 (0.00)	24.10 (26.21)	−12.99 (−10.79)	0.00 (0.00)	**9g** (99.9) (88.8) (99)
**8c**/**7b**-*exo*	2.96 (0.94)	28.32 (27.44)	−10.44 (−11.12)	4.22 (1.23)	**10g** (0.1) (11.2) (1)
**8c/7c***-endo*	0.00 (0.00)	23.97 (26.20)	−10.31 (−7.66)	0.00 (0.00)	**9h** (100) (99.9) (99)
**8c/7c***-exo*	5.40 (3.80)	32.39 (31.58)	−5.38 (−6.62)	8.42 (5.40)	**10h** (0) (0.01) (1)
**8g**/**7c**-*endo*	0.00 (0.00)	24.50 (26.83)	−9.60 (−6.55)	0.00 (0.00)	**9m** (100) (99.9) (91)
**8g**/**7c**-*exo*	4.25 (2.25)	30.93 (30.81)	−6.41 (−6.98)	6.43 (3.98)	**10m** (0) (0.1) (9)
**8j**/**7c**-*endo*	0.00 (0.00)	23.18 (25.49)	−11.22 (−9.62)	0.00 (0.00)	**9p** (99:9) (99.6) (99)
**8j**/**7c**-*exo*	3.11 (0.58)	28.96 (28.82)	−9.02 (−9.28)	5.77 (3.33)	**10p** (0.1) (0.4) (1)
**16a**/**7c**-*endo*	0.00 (0.00)	25.10 (26.70)	−7.72 (−6.22)	0.00 (0.00)	**9c** (100) (99.9) (94)
**16a**/**7c**-*exo*	3.70 (1.95)	30.31 (31.05)	−6.90 (−6.22)	5.21 (4.35)	**10c** (0) (0.1) (6)
**18a**/**7c**-*endo*	0.00 (0.00)	24.42 (26.68)	−6.25 (−5.24)	0.00 (0.00)	**11b** (100) (100) (91)^g^
**18a**/**7c**-*exo*	2.40 (2.07)	34.14 (34.34)	−4.39 (−4.11)	9.72 (7.66)	**20** (0) (0) (9)^g^

^a^The *endo/exo* TSs for each pair of cycloaddends lead to a mixture of adducts **9**/**10** or **11b**/**20**, whose ratios are indicated in both rows of the pairs **8**/**7**, **16a**/**7c** or **18a**/**7c**. ^b^Numbers in parentheses correspond to relative Gibbs energy (kcal/mol). ^c^Energy differences for the TSs, relative to the most stable *endo* approach of the pairs **8**/**7**, **16a**/**7c** or **18a**/**7c**. ^d^Percentage ratio of the pairs **9**/**10** or **11b**/**20**, calculated from the Boltzmann distribution (at 298 K) from the TSs obtained from the relative zero-point corrected energy. ^e^Percentage ratio of the pairs **9**/**10** or **11b**/**20**, calculated from the Boltzmann distribution (at 298 K) of the TSs obtained from the relative Gibbs energies. ^f^Percentage ratio of the pairs **9**/**10** or **11b**/**20**, determined from the experimental Diels–Alder cycloadditions (Table 2). ^g^For the ratio of **11b**/**20**, see Scheme 6.

For the reaction with **8b** (R^2^ = CN) the calculated *endo/exo* ratio (99.4:0.6) and the experimental one (76:24) did not concur. However, the ratio determined through the Gibbs energy (*endo/exo*, 79.9:20.1) matched better. Actually, the reactivity of diene **8b** would be expected to be lower due to a greater deactivation of the cyano group, as suggested by comparing the most stable HOMO of this diene with the values for dienes **8c** and **8g** (Supporting Information File 1, Table S1). Hence, the latter dienes should be more reactive than diene **8b**, and consequently more stereoselective [54–56].

In contrast, the Gibbs energy for the diene/dienophile **8c**/**7b** (88.8:11.2) did not completely match the experimental *endo/exo* (99:1) ratio, but the ZPE ratio (99.9:0.1) was in good agreement. For the cycloaddition of the pyrrolo[2,1-*a*]isoindole **18a** with **7c**, there was an additional conjugated system involved capable of perturbing the electronic density of the diene. If one assumes that the nitrogen lone-pair was partially delocalized to the benzene ring, a decrease in the electronic density should be anticipated for the methyl acrylate dienic moiety. According to the perturbation theory [57], such an effect would cause a reduction in the cycloaddition reactivity, because the diene needs electron-donating substituents to enhance it (under normal electron demand). Indeed, under the same reaction conditions as those employed for dienes **8**, it took 10 days of heating for diene **18a** to be consumed (Scheme 6). Thus, the limited reactivity of this diene resulted in a lower diastereoselectivity than most of the other dienes **8** as well as **16a**.

Non-covalent intermolecular interactions (NCIs) between aromatic rings (π···π) [58–59] and alkyl and aromatic ring (Csp*^3^*–H)···π stackings [59–62] are widely recognized, from both the experimental and theoretical viewpoint, as a major factor governing the conformational equilibrium, supramolecular assembly and stereoselective approaches of substrates and reagents or catalysts in a variety of processes. Examples include the substrate–enzyme recognition (the host–guest interaction) responsible for inducing pharmacological activity, the DNA-intercalation capable of generating biomolecular activity, molecular dynamics [63–70], and pericyclic reactions (Diels–Alder and 1,3-dipolar cycloadditions) [71–75].

**Figure 4:**
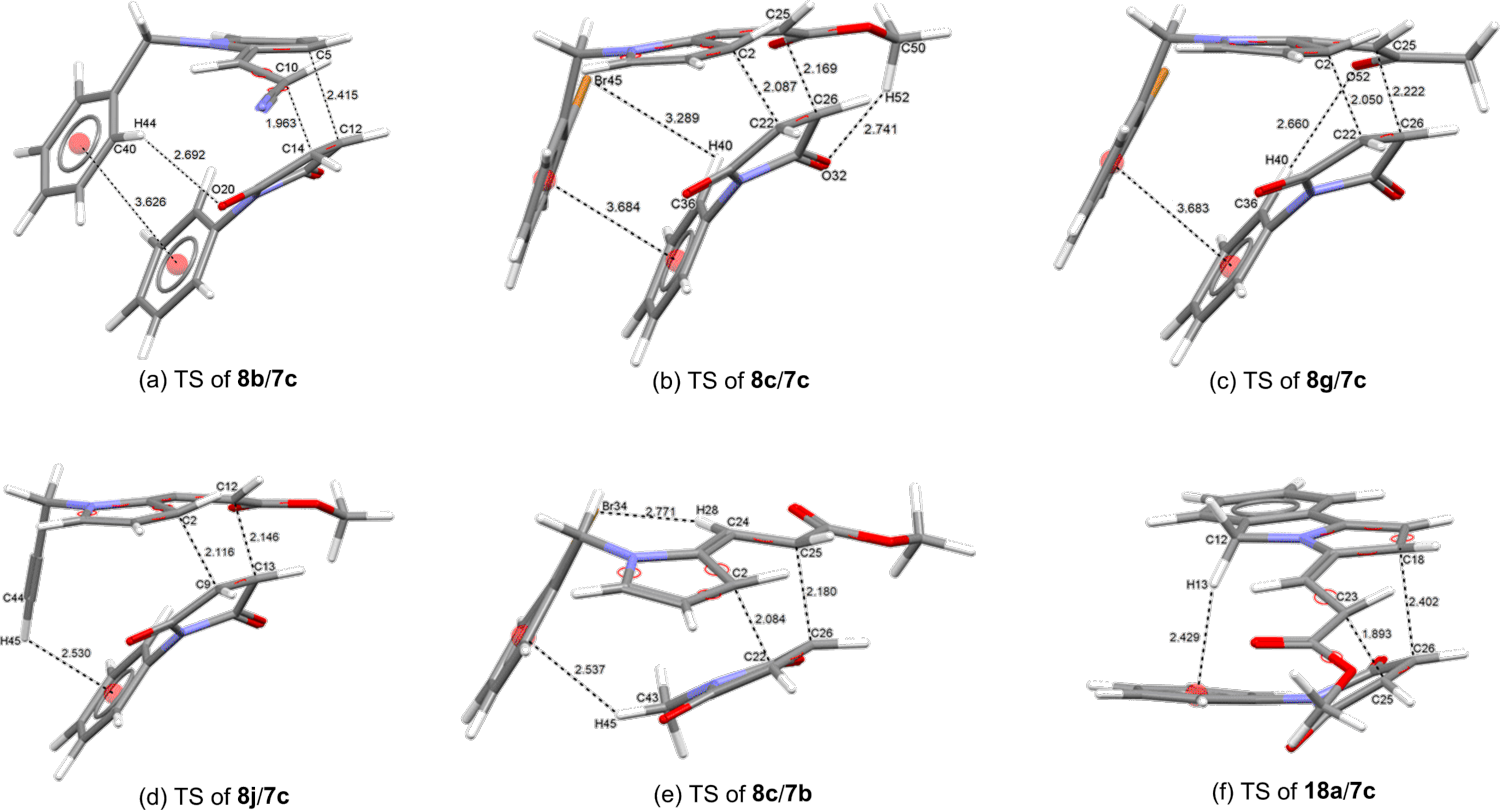
M06-2X/6-31+G(d,p) Optimized geometry for each of the TSs of the Diels–Alder reactions of dienes **8b**, **8c**, **8g**, **8j** and **18a** with dienophiles **7b**,**c** for the *endo* approach, showing the π···π, C–H···π, C–H···OC and C–H···Br stacking interactions and their distances (Å).

To account for the *endo* selectivity of all the tested dienes, the geometry of cycloaddends at the TSs was carefully analyzed (Figure 4 and Supporting Information File 1, Appendix 6) to determine the effects, such as NCIs, that occur to stabilize it. For example, in the *endo* TS of the cycloaddition of **8g**/**7c**, the benzene rings of both reagents closely approach each other in an observable π···π offset stacked interaction (3.683 Å) (π···σ attraction) (Figure 4c and Table 7), which is more stable than the eclipsed face-to-face geometry, overcoming the π···π repulsions [58–59]. The same interaction is also noticed for the SC (Figure 3a). Of course, this interaction is not present at the *exo* TS (Figure 3e). Similar interactions are perceptible in the approaches of cycloaddends **8b**/**7c** and **8c**/**7c** (Figure 4a,b and Supporting Information File 1, Appendix 6). All the observable π···π interactions were parallel-displaced (offset) π-stacking, and the distance values were within the range of the known values, whether determined from X-ray structures or calculated values (3.2–4.0 Å) [58–59,65].

**Table 7:** Calculated [M06-2X/6-31+G(d,p)] NCIs, including distances, angles and contact type from the ZPE-corrected geometry of each of the *endo* TSs of the Diels–Alder reactions of dienes **8b**, **8c**, **8g**, **8j** and **18a** with dienophiles **7b**,**c**.^a^

TS cycloaddends	interaction^b^	distance (Å)	angle (º)	contact type^c^

**8b/7c**	C–H···O	2.962	131.89	D–H···A
**8b/7c**	π···π	3.626	4.24	π-stacking (parallel-displaced)
**8c/7c**	C–H···O	2.741	119.78	D–H···A
**8c/7c**	C–H···Br^d^	3.289	98.02	D–H···A
**8c/7c**	π···π	3.684	16.02	π-stacking (parallel-displaced)
**8g/7c**	C–H···O^e^	2.660	163.14	D–H···A
**8g/7c**	π···π	3.683	15.90	π-stacking (parallel-displaced)
**8j/7c**	C_ethynyl_–H···π^f^	2.530	115.19	D–H···A
**8c/7b**	C_methyl_–H···π^g^	2.537	126.61	D–H···A
**8c/7b**	C–H···Br^h^	2.771	160.54	D–H···A
**18a/7c**	C_methylene_–H···π^i^	2.429	154.71	D–H···A

^a^Data taken from the TS geometry, obtained from the relative zero-point corrected energy (Figure 4). ^b^The intermolecular C–H···O interaction derived from the proton of the benzene ring or the proton of the methoxy group of the ester of the diene and a carbonyl oxygen of the dienophile. ^c^D = donating atom; A = acceptor atom or aromatic ring. ^d^The intermolecular C–H···Br derived from the aryl proton of **7c** and the bromine atom of diene **8c**. ^e^The intermolecular C–H···O interaction derived from the proton of the benzene ring of **7c** and of the carbonyl oxygen of diene **8g**. ^f^The C–H···π interaction derived from the proton of the terminal acetylene of diene **8j** and the benzene ring of **7c**. ^g^The C–H···π interaction derived from the proton of the *N-*methyl group of **7b** and the benzene ring of **8c**. ^h^The intramolecular C–H···Br interaction derived from the vinylic proton and the bromine atom of diene **8c**. ^i^The C–H···π interaction derived from the proton of methylene of the pyrrolizine moiety of diene **18a** and the benzene ring of **7c**.

**Figure 5:**
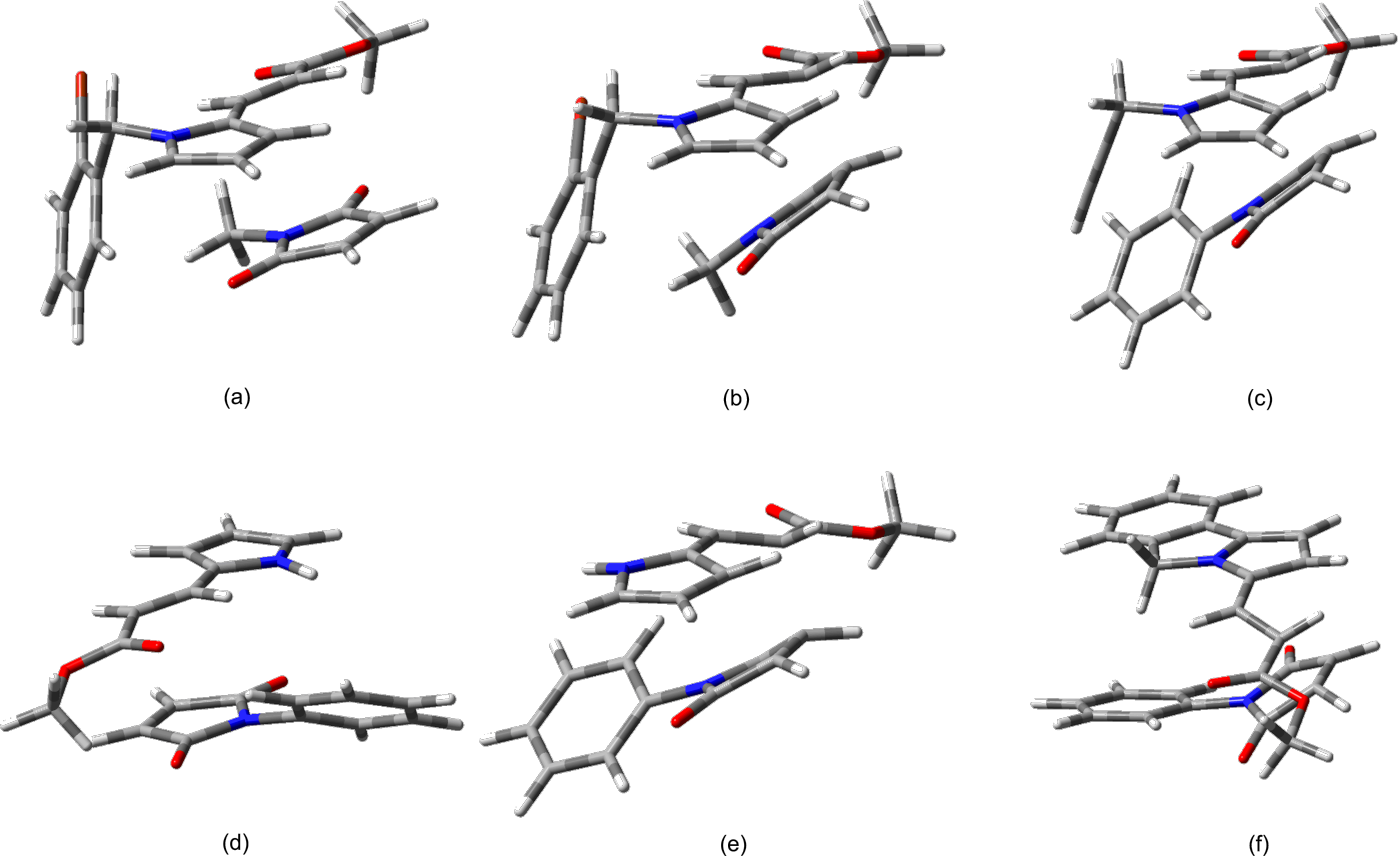
M06-2X/6-31+G(d,p) Optimized geometry of the *endo* SCs (a) and TSs (b) for the Diels–Alder reaction of diene **8c** and dienophile **7b**, the *endo* TSs (c) of the reaction of **8j** and **7c**, the *endo* approach of the SC (d) and TS (e) for the cycloaddition of **16a** and **7c**, and the *endo* TS (f) for the cycloaddition of **18a** and **7c**.

Regarding the addition of diene **8c** with the *N*-methylmaleimide (**7b**), a π···π interaction is discarded. Nevertheless, the *endo* adduct **9g** was obtained as the major diastereoisomer (Table 2, entry 7). The calculated geometry at the *endo* TS reveals a plausible C_methyl_–H···π interaction, based on the distance value (2.537 Å) to the benzene ring centroid. This interaction formed between one of the proton of the methyl group of **7b** and the *N-*aryl ring of the pyrrole **8c** (Table 7 and Figure 4e and Figure 5a,b). The aforementioned value matched well with that for the expected C_methyl_–H···π interaction (2.5–3.4 Å and 2.79 Å) [58–59,62,66] according to the calculations and the X-ray calculated average taken from the Cambridge Structural Database (CSD) [76].

Interestingly, in the case of the reaction between diene **8j** and **7c**, where the diene does not have an *N-*benzyl group, the *endo* TS also displays a C_ethynyl_–H···π interaction (2.530 Å) between the acetylene proton of **8j** and the *N-*phenyl ring of the *N*-phenylmaleimide (**7c**) (Table 7, and Figure 4d and Figure 5c). Such an interaction may explain the greater stabilization of the *endo* approach to give **9p** as the major isomer. Due to the scarce X-ray data from CSD about the distance of the C_ethynyl_–H···π interaction [76], an average distance value (ca. 3.0 Å) above the benzene ring centroid can be considered for the statistical distribution of the C_ethenyl_–H···π interaction [76], which is longer than present calculated value.

On the other hand, the cycloaddition of the *N-*unsubstituted pyrrole **16a** and *N-*phenylmaleimide (**7c**) proved to be highly *endo* diastereoselective as well (Table 2, entry 3). Hence, a supplementary N–H···π interaction between the N–H bond of pyrrole **16a** and the *N-*phenyl ring of **7c** may be involved at the SC and TS to improve the stability of such an approach (Figure 5d,e). Despite the fact that this interaction was not clearly detected at the *endo* TS, an incipient N–H···π interaction seems to be present (Supporting Information File 1, Appendix 6).

The preference of the *endo* selectivity for the cycloaddition of the isoindolyl diene **18a** and **7c** can be also accounted for by a perceptible C_methylene_–H···π interaction (2.429 Å to the benzene ring centroid) (Table 7 and Figure 4f) between a proton of the C-5 methylene group of the pyrrolo[2,1-*a*]isoindole ring of **18a** and the *N-*phenyl ring of **7c** (Figure 5f). Additional hydrogen bonding, which was found for these and the other cycloaddends between a proton of the benzene ring of the diene and an oxygen atom of the dienophile (Table 7), also stabilize the *endo* TS.

Therefore, this theoretical study indicated that the non-covalent π···π and C–H···π interactions control the *endo* selectivity in the Diels–Alder cycloadditions. Since experimental and theoretical results have demonstrated that the nature of the C–H···π interaction mainly depends on the dispersion interactions [64,67,77], these are probably not only at the origin of the *endo* stereoselectivity of the present cycloadditions, but also involved in the general *endo*-Alder rule [53]. The latter rule has been traditionally attributed to secondary orbital interactions (SOI) [78–80], although a controversy exists due to the lack of solid evidence for this hypothesis [81]. Consequently, a lot of research has been carried out to provide additional insights into the factors controlling the *endo* outcome [53,82–85], documenting in some cases a key role of NCIs in the preferential stereocontrol [86].

## Conclusion

The Diels–Alder reaction of *N*-substituted-2-vinylpyrroles **8a**–**j** and *N*-unsubstituted-2-vinylpyrroles **16a**,**b** with maleimides **7b**,**c** proved to be highly *endo* diastereoselective. This selectivity turned out to be significant in the case of vinyl benzopyrrolizine **18a** as well. The consecutive Pd(0) cross-coupling reaction of some of the Diels–Alder adducts allowed for the synthesis of a numerous series of pyrrolizine-containing polycycles, including isoindolo[2,1-*a*]indole-based tetrahydro and fully aromatic pentacycles. These findings reveal the synthetic value of 2-formylpyrrole (**13a**) for the diastereo- and regioselective construction of isoindolo- and pyrrolo-fused polycyclic indoles. Theoretical calculations suggest that the greater stability of the *endo* TSs in the Diels–Alder cycloadditions is associated with NCIs between the *N-*substituents of both cycloaddends. These interactions are also involved in the case of pyrrolo[2,1-*a*]isoindole **18a** with dienophile **7c**.

## Supporting Information

**Appendix 1**: Energies and coefficients of the Frontier Molecular Orbitals [HF/6-31G(d,p)] of dienes **8b**, **8c** and **8g**, and dienophile **7c**. **Appendix 2**: Relative zero point-corrected energies of the SCs, TSs, and ADs located on the potential surfaces of the Diels–Alder reactions of dienes **8b**, **8c**, **8g**, **8j**, **16a**, and **18a**, and dienophiles **7b**,**c**. **Appendix 3**: X-ray crystallographic structures of **9m** and **10m**. **Appendix 4**: M06-2X/6-31+G(d,p) relative Gibbs free energies (kcal/mol) of the stationary points in the Diels–Alder cycloadditions of dienes **8b**, **8c**, **8g**, **8j**, **16a**, and **18a**, and dienophiles **7b**,**c**. **Appendix 5**: Calculation [M06-2X/6-31+G(d,p)] of Z-matrices of the optimized geometries of the SCs, TSs, and ADs of the Diels–Alder cycloadditions of dienes **8b**, **8c**, **8g**, **8j**, **16a**, and **18a**, and dienophiles **7b**,**c**. **Appendix 6**: Calculation [M06-2X/6-31+G(d,p)] of the NCIs, including distances, angles and contact type from the ZPE-corrected geometries of the *endo* TSs of the Diels–Alder reactions of dienes **8b**, **8c**, **8g**, **8j** and **18a** and dienophiles **7b**,**c**. **Appendix 7**: Experimental section. **Appendix 8**: ^1^H and ^13^C NMR spectra for all new compounds.

File 1 Experimental and analytical data, X-ray crystallographic structures, NMR-spectra and all calculated data.
