# Supplementary material for: Highly selective Diels–Alder and Heck arylation reactions in a divergent synthesis of isoindolo- and pyrrolo-fused polycyclic indoles from 2-formylpyrrole
Source: Beilstein J Org Chem. 2020 Jun 17;16:1320–34. doi: 10.3762/bjoc.16.113 (PMC7308616; doi:10.3762/bjoc.16.113)

BEILSTEIN JOURNAL OF ORGANIC CHEMISTRY

Supporting Information

for

Highly selective Diels–Alder and Heck arylation reactions in a divergent synthesis of isoindolo- and pyrrolo-fused polycyclic indoles from 2-formylpyrrole

Carlos H. Escalante, Eder I. Martínez-Mora, Carlos Espinoza-Hicks, Alejandro A. Camacho-Dávila, Fernando R. Ramos-Morales, Francisco Delgado and Joaquín Tamariz

Beilstein J. Org. Chem. **2020,** *16,* 1320–1334. doi:10.3762/bjoc.16.113

Experimental and analytical data, X-ray crystallographic structures, NMR-spectra and all calculated data

License and Terms: This is a supporting information file under the terms of the Creative Commons Attribution License (http://creativecommons.org/licenses/by/4.0). Please note that the reuse, redistribution and reproduction in particular requires that the authors and source are credited.
The license is subject to the *Beilstein Journal of Organic Chemistry* terms and conditions: (https://www.beilstein-journals.org/bjoc)

Appendix 1. Energies and coefficients of the frontier molecular orbitals [HF/6-31G(d,p)] for dienes **8b**, **8c** and **8g**, and dienophile **7c**. S2

Appendix 2. Relative zero point-corrected energies of the SCs, TSs, and ADs located on the potential surfaces of the Diels–Alder reactions of dienes **8b**,**8c**, **8g**, **8j**, **16a**, and **18a**, and dienophiles **7b-c**. S3

Appendix 3. X-Ray crystallographic structures of **9m** and **10m**. S4–S7

Appendix 4. M06-2X/6-31+G(d,p) relative Gibbs free energies (kcal/mol) of the stationary points for the two possible approaches *endo* and *exo* in the Diels–Alder cycloadditions of dienes **8b**, **8c**, **8g**, **8j**, **16a**, and **18a**, and dienophiles **7b-c**. S8–S9

Appendix 5. Calculation [M06-2X/6-31+G(d,p)] of Z-matrices of the optimized geometries of the SCs, TSs, and ADs of the Diels–Alder cycloadditions of dienes **8b**, **8c**, **8g**, **8j**, **16a**, and **18a**, and dienophiles **7b-c**. S10–S51

Appendix 6. Calculated [M06-2X/6-31+G(d,p)] NCIs, including distances, angles and contact type from the ZPE-corrected geometries of the *endo* TSs of the Diels–Alder reactions of dienes **8b**, **8c**, **8g**, **8j** and **18a**, and dienophiles **7b-c**. S52–S58

Appendix 7. Experimental section. S59–S95

Appendix 8. ^{1}H and ^{13}C NMR spectra for all new compounds. S96–S152

Appendix 1

Table S1. Energies (eV) and coefficients (C_i) of the frontier molecular orbitals[HF/6-31G(d,p)] for dienes **8b**,**8c** and**8g**, and dienophile **7c**.[a]

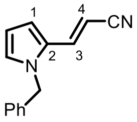

8b

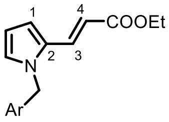

8c

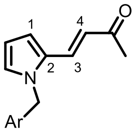

8g

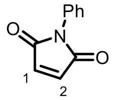

7c

		HOMO						LUMO				
Compd[b]	*E* (eV)	C_1	C_2	C_3	C_4	ΔC_I[c]	*E* (eV)	C_1	C_2	C_3	C_4	ΔC_I[c]
**8b**	-7.993	-0.153	-0.245	0.089	0.222	0.069	1.320	-0.084	0.056	0.134	-0.133	0.049
		-0.129	-0.221	0.084	0.196	0.067		-0.103	0.051	0.179	-0.172	0.069
**8c**	-7.665	0.190	0.296	-0.098	-0.247	0.057	2.244	-0.189	0.106	0.276	-0.221	0.032
		0.173	0.274	-0.092	-0.230	0.057		-0.293	0.156	0.422	-0.376	0.083
**8g**	-7.625	0.189	0.292	-0.103	-0.248	0.059	2.131	-0.185	0.098	0.273	-0.204	0.019
		0.174	0.268	-0.092	-0.237	0.063		-0.282	0.140	0.418	-0.351	0.069
**7c**	-8.982	0.047	0.047			0.000	0.860	0.196	-0.196			0.000
		0.032	0.032			0.000		0.251	-0.251			0.000

[a]These are the values of the p_z coefficients, relative p_z' contributions and their ΔC_i are analogous. [b] For the most stable planar *s-cis* conformation for dienes**8**. [c] Carbon 4–carbon 1 for the dienes; carbon 1–carbon 2 for the dienophile.Carbon 4–carbon 1 for the dienes; carbon 1–carbon 2 for the dienophile.

Appendix 2

Table S2. Relative zero point-corrected energies (kcal/mol) of the SCs, TSs, and ADs located in the potential surfaces of the Diels–Alder reactions of dienes**8b**,**8c**, **8g**, **8j**, **16a**, and **18a**,and dienophiles **7b**,**c**.

Cycloaddends	SC	TS	Adduct	diff	Boltz.[b]
**8b/7c-*exo***	0.76	29.99	-7.94	-----	0.56
**8b/7c-*endo***	0.00	26.92	-9.76	3.07	99.44
**8b/7c-*exo*[a]**	0.65	30.53	-7.31	-----	20.12
**8b/7c-*endo*[a]**	0.00	29.72	-6.47	0.82	79.88
**8c/7c-*exo***	5.40	32.39	-5.38	-----	0
**8c/7c-*endo***	0.00	23.97	-10.31	8.42	100
**8c/7c-*exo*[a]**	3.80	31.58	-6.62	----	0.01
**8c/7c-*endo*[a]**	0.00	26.20	-7.66	5.40	99.99
**8g/7c-*exo***	4.25	30.93	-6.41	-----	0
**8g/7c-*endo***	0.00	24.50	-9.60	6.43	100
**8g/7c-*exo*[a]**	2.25	30.81	-6.98	----	0.12
**8g/7c-*endo*[a]**	0.00	26.83	-6.55	3.98	99.88
**8c/7b-*exo***	2.96	28.32	-10.44	---	0.08
**8c/7b-*endo***	0.00	24.10	-12.99	4.22	99.92
**8c/7b-*exo*[a]**	0.94	27.44	-11.12	---	11.22
**8c/7b-*endo*[a]**	0.00	26.21	-10.79	1.23	88.78
**16a/7c-*exo***	3.70	30.31	-6.90	---	0.02
**16a/7c-*endo***	0.00	25.10	-7.72	5.21	99.98
**16a/7c-*exo*[a]**	1.95	31.05	-6.22	---	0.06
**16a/7c-*endo*[a]**	0.00	26.70	-6.22	4.35	99.94
**8j/7c-*exo***	3.11	28.96	-9.02	---	0.01
**8j/7c-*endo***	0	23.18	-11.22	5.77	99.99
**8j/7c-*exo* [a]**	0.58	28.82	-9.28	---	0.36
**8j/7c-*endo*[a]**	0	25.49	-9.62	3.33	99.64
**18a/7c-*exo***	2.40	34.14	-4.39	---	0
**18a/7c-*endo***	0	24.42	-6.25	9.72	100
**18a/7c-*exo* [a]**	2.07	34.34	-4.11	---	0
**18a/7c-*endo*[a]**	0	26.68	-5.24	7.66	100

[a]Relative Gibbs Free Energy (kcal/mol). [b]Boltzmann distribution (%) of the transition states located in the potential surfaces at 298.15 K (25 °C).

Appendix 3

X-Ray crystallographic structures of **9m** and **10m**

(3a*S,4*S**,8b*S**)-4-Acetyl-6-(2-bromobenzyl)-2-phenyl-4,5,6,8b-tetrahydropyrrolo[3,4-*e*]indole-1,3(2*H*,3a*H*)-dione (9m).**

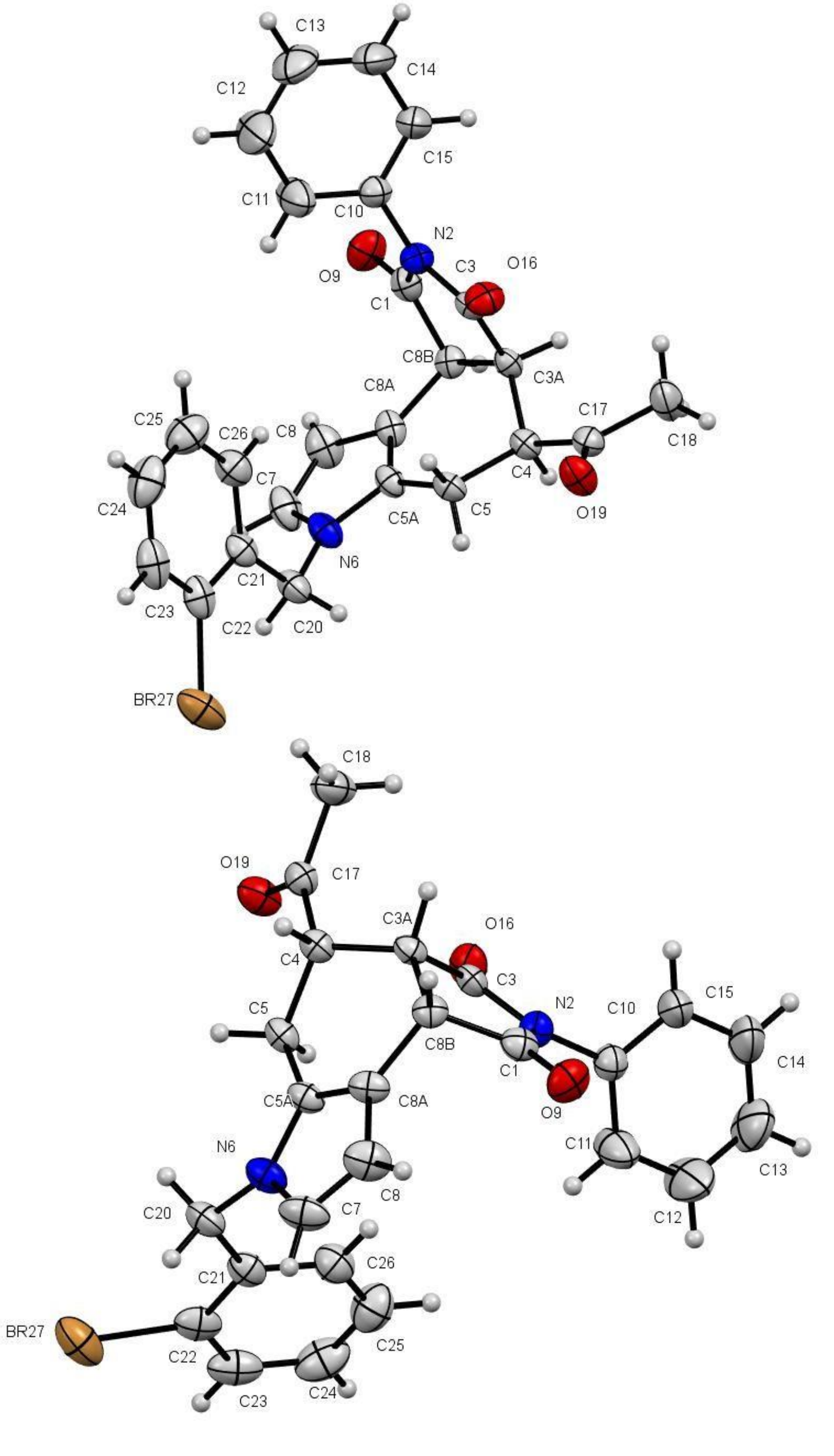

Table 1. Crystal data and structure refinement for 0130-jt.

Identification code	**CCDC1987245**	
Empirical formula	C25 H21 Br N2 O3	
Formula weight	477.35	
Temperature	291(2) K	
Wavelength	0.71073 Å	
Crystal system	Orthorhombic	
Space group	P c a 21	
Unit cell dimensions	a = 10.8111(4) Å	α= 90°.
	b = 11.5651(7) Å	β= 90°.
	c = 17.8855(7) Å	γ = 90°.
Volume	2236.25(18) $Å^3$	
Z	4	
Density (calculated)	1.418 Mg/m^3	
Absorption coefficient	1.867 mm^{-1}	
F(000)	976	
Crystal size	0.230 x 0.200 x 0.080 mm^3	
Theta range for data collection	3.442 to 32.624°.	
Index ranges	-15<=h<=14, -17<=k<=16, -27<=l<=26	
Reflections collected	12660	
Independent reflections	6914 [R(int) = 0.0495]	
Observed reflections	2904	
Completeness to theta = 25.242°	99.8 %	
Refinement method	Full-matrix least-squares on F^2	
Data / restraints / parameters	6914 / 1 / 281	
Goodness-of-fit on F^2	0.946	
Final R indices [I>2sigma(I)]	R1 = 0.0558, wR2 = 0.0680	
R indices (all data)	R1 = 0.1694, wR2 = 0.0921	
Absolute structure parameter	0.013(6)	
Extinction coefficient	n/a	
Largest diff. peak and hole	0.434 and -0.372 e.$Å^{-3}$	

(3a*R,4*S**,8b*R**)-4-acetyl-6-(2-bromobenzyl)-2-phenyl-4,5,6,8b-tetrahydropyrrolo[3,4-e]indole-1,3(2H,3aH)-dione (10m)**

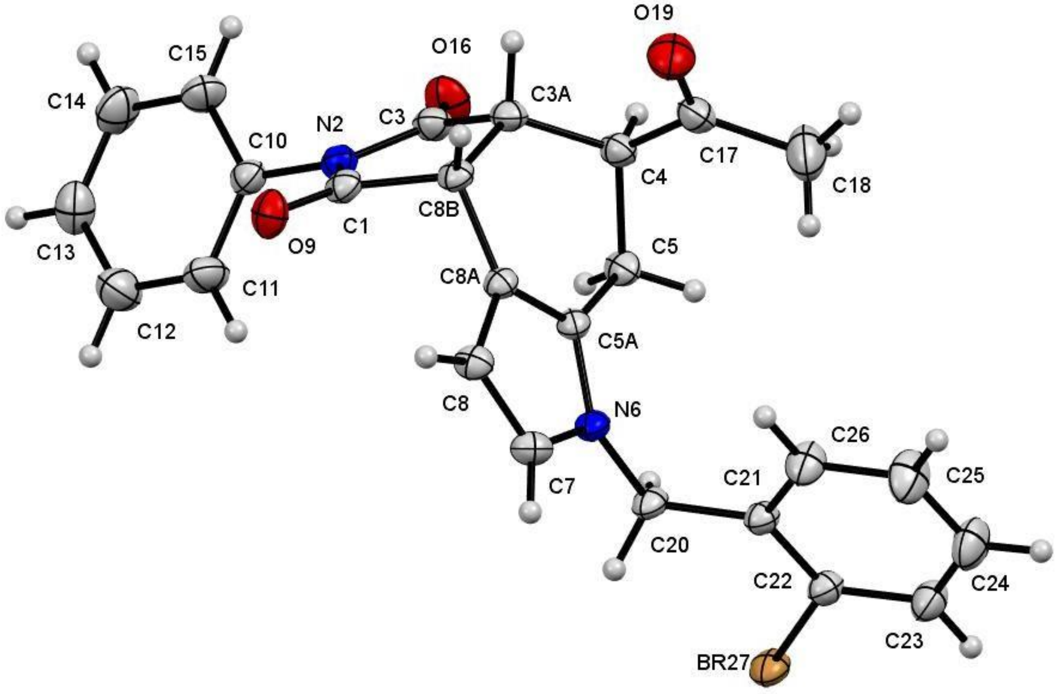

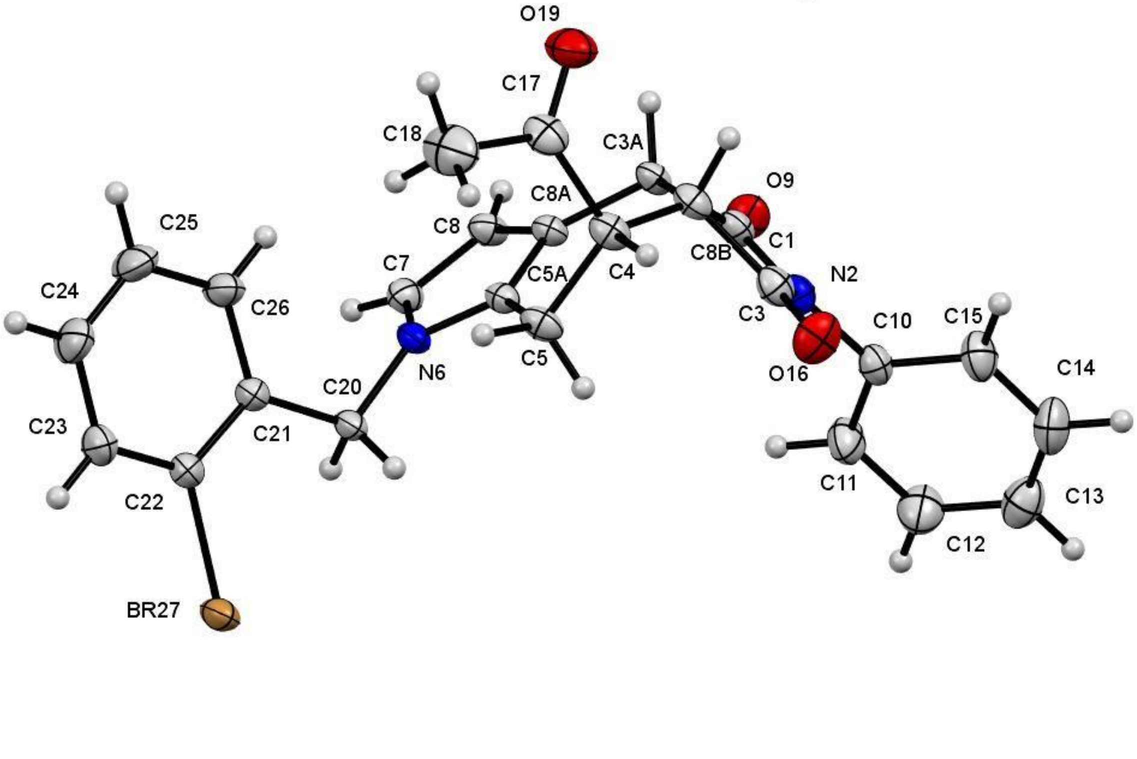

Table 1. Crystal data and structure refinement for 0129-jt.

Identification code	**CCDC 1987244**	
Empirical formula	C25 H21 Br N2 O3	
Formula weight	477.35	
Temperature	291(2) K	
Wavelength	0.71073 Å	
Crystal system	Triclinic	
Space group	P -1	
Unit cell dimensions	a = 9.3102(3) Å	α= 97.296(3)°.
	b = 10.9639(5) Å	β= 103.708(3)°.
	c = 11.1414(4) Å	γ = 98.674(3)°.
Volume	1076.45(7) $Å^3$	
Z	2	
Density (calculated)	1.473 Mg/m^3	
Absorption coefficient	1.939 mm^{-1}	
F(000)	488	
Crystal size	0.700 x 0.590 x 0.470 mm^3	
Theta range for data collection	3.246 to 32.547°.	
Index ranges	-13<=h<=13, -16<=k<=16, -16<=l<=16	
Reflections collected	23063	
Independent reflections	7192 [R(int) = 0.0255]	
Observed reflections	5385	
Completeness to theta = 25.242°	99.7 %	
Refinement method	Full-matrix least-squares on F^2	
Data / restraints / parameters	7192 / 0 / 281	
Goodness-of-fit on F^2	1.009	
Final R indices [I>2sigma(I)]	R1 = 0.0395, wR2 = 0.0844	
R indices (all data)	R1 = 0.0608, wR2 = 0.0921	
Extinction coefficient	n/a	
Largest diff. peak and hole	0.605 and -0.532 e.$Å^{-3}$	

Appendix 4

M06-2X/6-31+G(d,p) relative Gibbs free energies (kcal/mol) of the stationary points for the two possible approaches *endo* (blue) and *exo*(red) of the Diels–Alder cycloadditions of dienes **8b**, **8c**, **8g**, **8j**, **16a**, and **18a**,and dienophiles **7b**,**c**. **SC** = supramolecular complex; **TS** = transition state; **AD** = adduct.

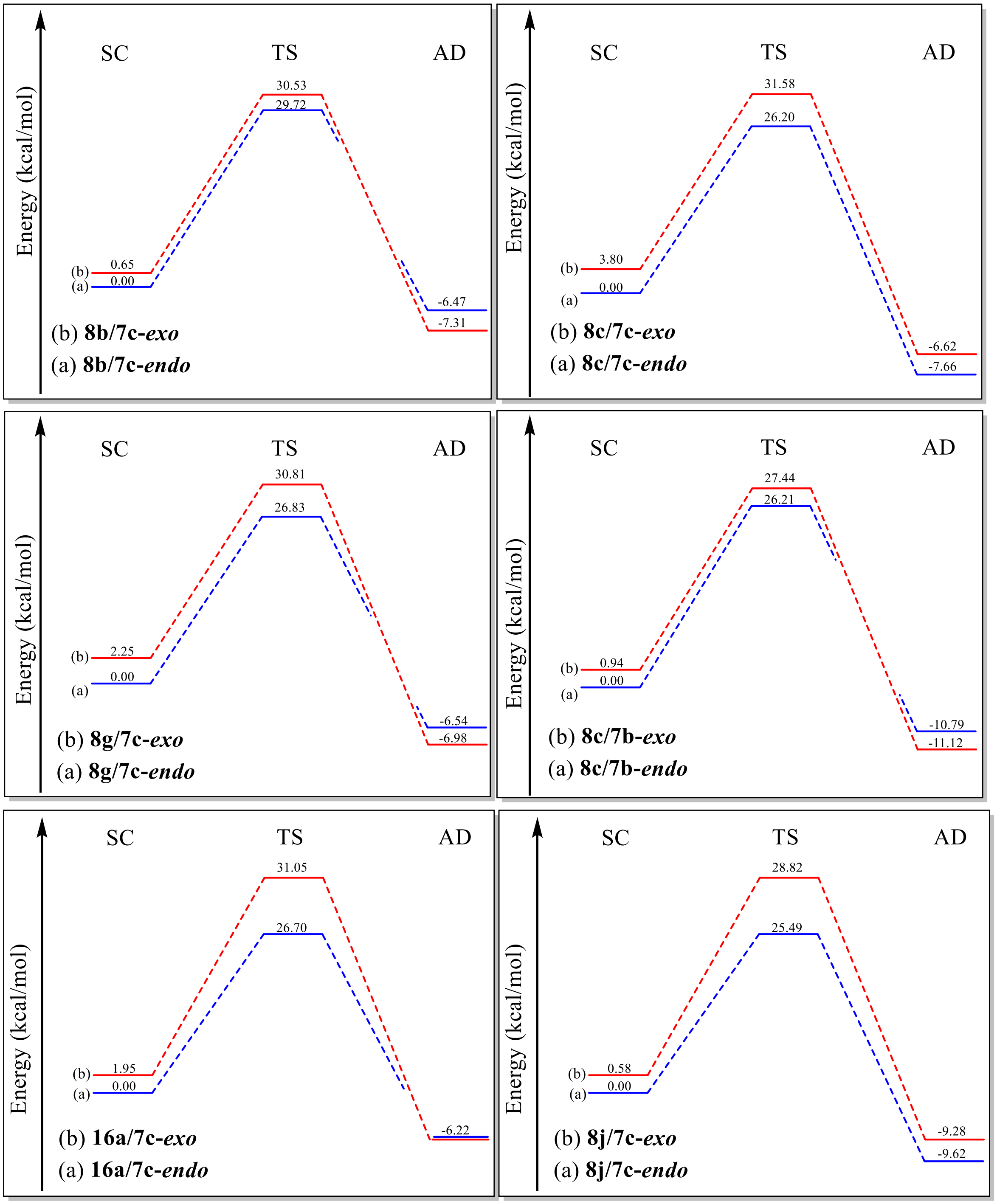

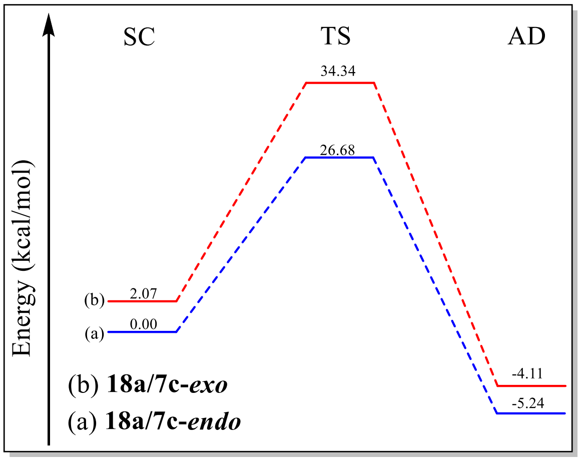
SC
TS
AD
Energy (kcal/mol)
34.34
26.68
(b)
2.07
(a)
0.00
-4.11
-5.24
(b) **18a/7c-*exo***
(a) **18a/7c-*endo***

Appendix 5

Calculation [M06-2X/6-31+G(d,p)] of the Z-matrices for the optimized geometries of the SCs, TSs, and ADs of the Diels–Alder cycloadditions of dienes **8b**, **8c**, **8g**, **8j**, **16a**, and **18a**,and dienophiles **7b-c**.

Supramolecular complex (**SC**) **8b/7c-*endo***.

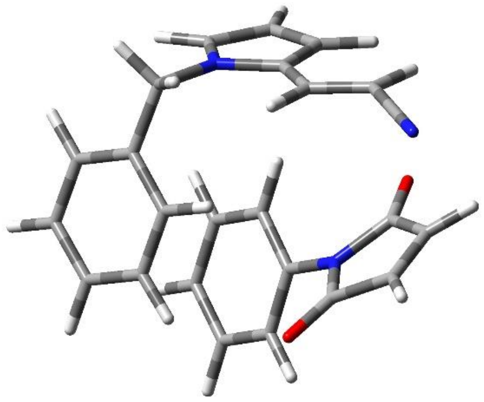

C	-1.18286800	2.19344200	0.42136000	C	1.03480900	-0.44354600	1.39584900
N	0.05684000	2.68633100	0.04601000	C	2.36240200	-0.29764400	1.78649800
C	0.71624300	3.11727600	1.15381800	C	2.79670000	-2.60255000	1.22103900
C	-0.08984900	2.92137700	2.26309100	H	1.11126300	-3.71423300	0.45543500
C	-1.28586000	2.34159400	1.80323000	H	0.34174600	0.38808700	1.45769500
H	1.71089400	3.53265100	1.07075800	H	2.70305000	0.66654000	2.15163800
H	-2.12513700	2.02508900	2.40590300	H	3.47908500	-3.44365100	1.15100300
C	-2.12780100	1.64188500	-0.51568800	H	4.28462200	-1.24875100	1.98899600
H	-1.82822600	1.55498500	-1.55721100	C	-4.24381300	0.65663400	-1.16588600
C	-3.36788500	1.21973400	-0.18814000	N	-4.95904500	0.17007400	-1.93906200
H	-3.74346200	1.27705300	0.82914400	C	0.65082700	2.70113700	-1.29237900
C	-3.06928400	-1.87086400	0.36842300	H	1.14246900	3.67015600	-1.41746200
H	-4.08647500	-1.68630900	0.68834000	H	-0.15425300	2.65504900	-2.02739400
C	-2.62881600	-2.46584000	-0.73773600	C	1.64340800	1.57962600	-1.49533500
H	-3.18815700	-2.88270600	-1.56425800	C	3.48464100	-0.50901600	-1.74990000
H	0.16531600	3.16658200	3.28323600	C	1.21046700	0.28993700	-1.80938400
C	-1.12913500	-2.45838500	-0.71837400	C	3.00859400	1.81717200	-1.32330900
C	-1.89019700	-1.41884600	1.18242600	C	3.92763900	0.77738000	-1.44809900
N	-0.74826100	-1.82743600	0.47528800	C	2.12471100	-0.75343700	-1.93504200
O	-0.37910100	-2.89470100	-1.55894400	H	0.14869100	0.08628400	-1.94038000
O	-1.90368700	-0.82019500	2.22951200	H	3.35512000	2.82205500	-1.09164600
C	0.59820000	-1.67714000	0.91651800	H	4.98659300	0.97176700	-1.30870200
C	3.24707600	-1.37136900	1.69442100	H	1.76441400	-1.75354200	-2.15400800
C	1.46827700	-2.76309100	0.83508900	H	4.19708000	-1.32351200	-1.83624100

Transition State (**TS**) **8b/7c-*endo***.

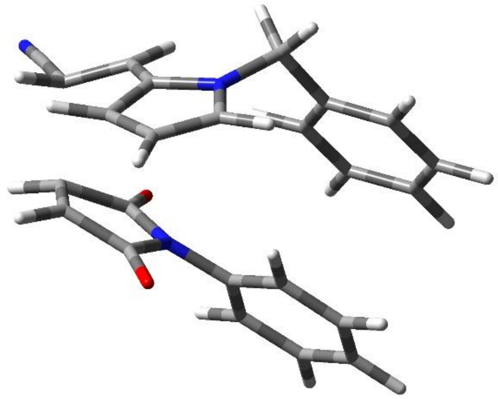

C	-2.01430900	1.51289900	0.14259700	C	1.94960700	-0.50453600	1.33267300
N	-0.87145800	2.28940700	0.27853600	C	3.32833800	-0.68094500	1.36582800
C	-0.70010200	2.58106300	1.59889600	C	3.11013600	-2.72407800	0.10646200
C	-1.72265100	2.04332600	2.34962300	H	1.09675400	-3.29619900	-0.41873400
C	-2.56856700	1.34626400	1.45203500	H	1.48553500	0.35627400	1.80078400
H	0.14558800	3.17948300	1.91298800	H	3.94778400	0.05980700	1.86173100
H	-3.59612400	1.07403000	1.64072900	H	3.55724900	-3.58858400	-0.37426900
C	-2.39795900	0.84405200	-1.00615500	H	4.99170600	-1.91867500	0.77767200
H	-1.81908100	0.92071200	-1.92010200	C	-3.76199800	-0.85984900	-2.11332500
C	-3.36087400	-0.18347100	-0.89912000	N	-4.10569700	-1.42508300	-3.06278100
H	-4.19848700	-0.02689700	-0.22359600	C	-0.04128000	2.78837200	-0.81588600
C	-2.40247900	-1.06005400	1.57344700	H	0.21709800	3.82363700	-0.57347100
H	-3.13952100	-1.10957300	2.36256200	H	-0.67476700	2.82334000	-1.70658900
C	-2.54657600	-1.54030000	0.26244300	C	1.22199900	2.00365300	-1.09311800
H	-3.26693000	-2.30473400	-0.01501900	C	3.59152800	0.64176100	-1.70113000
H	-1.83620400	2.13109300	3.41873900	C	1.18096300	0.76304100	-1.73577500
C	-1.14033600	-1.70801400	-0.28475300	C	2.46158100	2.54966900	-0.75102800
C	-0.98306800	-0.97333600	1.90896100	C	3.64246100	1.87368500	-1.05235500
N	-0.26309200	-1.30286200	0.71904900	C	2.35904700	0.08544100	-2.03789500
O	-0.83102600	-2.09488400	-1.38937100	H	0.23300900	0.30159100	-1.99943900
O	-0.46330600	-0.69460100	2.96506700	H	2.50428500	3.51905400	-0.25912500
C	1.15034200	-1.45692800	0.69714700	H	4.59915200	2.31064300	-0.78328300
C	3.91427000	-1.78835300	0.75341800	H	2.30619800	-0.88334000	-2.52415500
C	1.72569600	-2.56921800	0.08136900	H	4.50852800	0.10911900	-1.93277100

Adduct (**AD**) **8b/7c-*endo***.

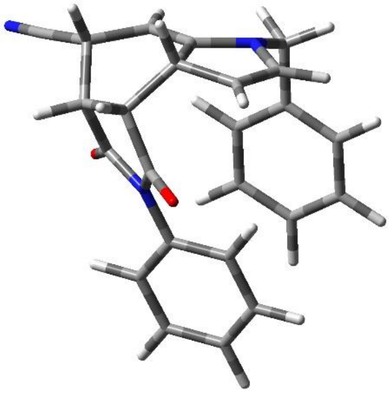

C	-2.15204500	1.25325100	0.23673300
N	-1.42016700	2.42451000	0.37759200
C	-0.97053200	2.50073300	1.70438300
C	-1.46810300	1.52627000	2.47602400
C	-2.34942400	0.64876400	1.61790700
H	-0.31772000	3.31889800	1.98419100
H	-3.40621600	0.76785600	1.89951600
C	-2.61622200	0.65497900	-0.87066100
H	-2.48809000	1.03732100	-1.87533700
C	-3.23258000	-0.70861200	-0.65730300
H	-4.19923600	-0.63207800	-0.13529700
C	-2.07002300	-0.89249500	1.63165600
H	-2.65622500	-1.35616700	2.42693700
C	-2.29323700	-1.53453500	0.25495700
H	-2.66756700	-2.55891000	0.36000800
H	-1.30307500	1.39527100	3.53506500
C	-0.88465500	-1.61380200	-0.34134200
C	-0.60018700	-1.14403600	1.91315600
N	0.03449000	-1.41526800	0.69363800
O	-0.60649600	-1.83216100	-1.49415500
O	-0.05831900	-1.09041400	2.98854400
C	1.45183200	-1.50410200	0.55139100
C	4.21406400	-1.66272000	0.30414200
C	2.01220900	-2.48327300	-0.26802500
C	2.25728600	-0.59823000	1.24093600
C	3.63972000	-0.68982900	1.12063400
C	3.39769600	-2.55109500	-0.39375900
H	1.37219500	-3.16854900	-0.81098900
H	1.80189200	0.16666800	1.86023200
H	4.26832500	0.01407500	1.65674400
H	3.83755700	-3.30854300	-1.03451900
H	5.29349700	-1.72659300	0.20915200
C	-3.52001000	-1.39969100	-1.92194500
N	-3.80582800	-1.93382000	-2.90595000
C	-0.69240700	3.03562900	-0.72886200
H	-0.53424700	4.08861400	-0.47445900
H	-1.34846400	3.01462300	-1.60395400
C	0.63735500	2.37467500	-1.03350000
C	3.11507700	1.18652200	-1.59665300
C	0.70781200	1.20032900	-1.79150900
C	1.82343700	2.93927900	-0.55390100
C	3.05600800	2.35310700	-0.83599800
C	1.93839700	0.60580600	-2.06512200
H	-0.19645000	0.74077600	-2.17777900
H	1.78317500	3.85735100	0.02883500
H	3.96883600	2.80966500	-0.46528100
H	1.96910400	-0.31347900	-2.64174300
H	4.07296300	0.72430700	-1.81344700

Supramolecular Complex (**SC**) **8b/7c-*exo***.

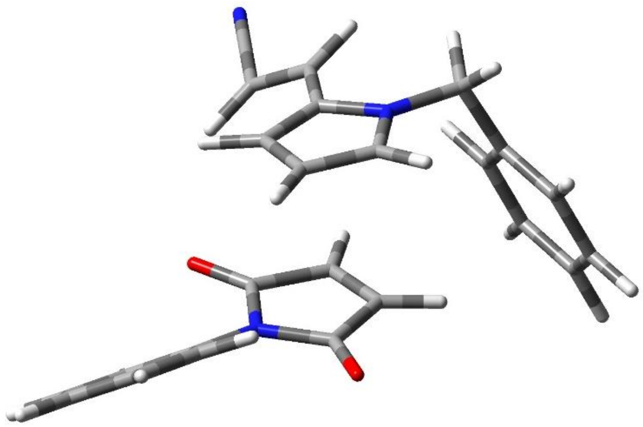

N	-1.62162000	0.08530300	-1.90476000	C	-3.58811700	-0.37016200	0.66236500
C	0.57563300	-0.12303200	-2.24698100	C	-3.80708300	-1.07432700	1.84445700
H	1.52902900	-0.52521700	-2.55700200	C	-3.56400200	-3.15650300	0.64765600
C	-3.02950200	-0.29056400	-1.80187400	H	-3.15815800	-2.98562900	-1.45955800
H	-3.63608400	0.61487500	-1.87004100	H	-3.60161500	0.71790900	0.67108600
H	-3.26366700	-0.90297400	-2.67694700	H	-3.99470900	-0.53525100	2.76776800
C	0.33298800	1.14887100	-1.68946100	H	-3.55878200	-4.24168300	0.63600500
H	1.06194800	1.92122500	-1.48413400	H	-3.96356700	-3.01853800	2.75957700
C	-1.03491000	1.25697900	-1.46227000	C	1.10302000	0.58801000	1.17700400
C	-1.78834400	2.34668000	-0.87806600	C	1.14684200	-1.64089800	0.63723700
H	-2.84189300	2.44275300	-1.13334700	N	1.92569400	-0.47590000	0.78162800
C	-1.25142500	3.25581600	-0.04077300	C	3.30166800	-0.35623900	0.44293300
C	-0.27617400	0.02646900	1.33483700	C	5.99759500	-0.10093400	-0.21964900
C	-0.25396100	-1.26664200	1.01874600	C	4.12206100	0.49505200	1.18690200
H	-0.21978000	3.18852200	0.29760400	C	3.82586400	-1.08663000	-0.62648700
C	-2.03935300	4.33720600	0.46847800	C	5.17462700	-0.95758200	-0.94758900
N	-2.68039300	5.20524900	0.89218400	C	5.46533500	0.62347800	0.84512300
H	-1.10379700	0.64915400	1.64807200	H	3.70682900	1.05820500	2.01356800
C	-0.64772100	-0.75899700	-2.35123700	H	3.18979200	-1.76503100	-1.18125700
H	-0.90050900	-1.74165500	-2.72426400	H	5.58041700	-1.53196600	-1.77420400
H	-1.06001000	-1.98984900	1.01113400	H	6.09802800	1.29064300	1.42153800
C	-3.34008500	-1.05133400	-0.53208900	H	7.04684300	-0.00139200	-0.47770900
C	-3.79195000	-2.46974800	1.83924800	O	1.54679500	-2.72315200	0.28562200
C	-3.33998900	-2.44764400	-0.53223600	O	1.44893200	1.73432400	1.34826600

Transition State (**TS**) **8b/7c-*exo***.

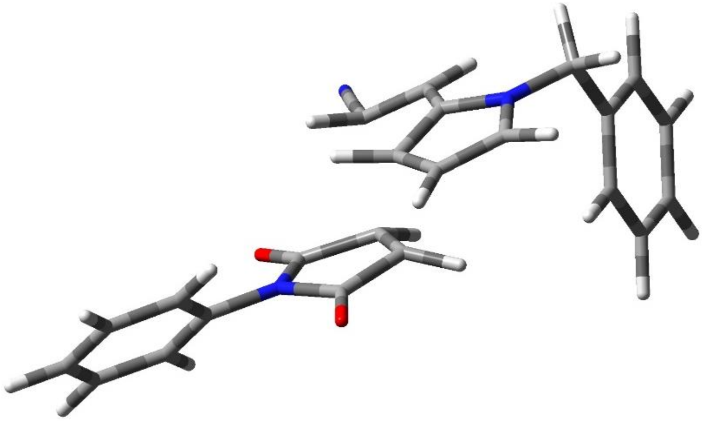

N	-2.50096300	-1.77450700	-1.08656700	C	-5.10945600	0.63460200	-0.17185400
C	-0.62630400	-2.91439800	-0.57303100	C	-5.42936400	1.51119900	0.86469700
H	0.04018600	-3.70632900	-0.26804800	C	-4.01200100	0.25520900	2.35767400
C	-3.89991700	-1.38849000	-1.08138900	H	-3.01882100	-1.45249800	1.50114700
H	-4.14649900	-0.93069500	-2.04575900	H	-5.52858500	0.79302600	-1.16297100
H	-4.48598700	-2.30957300	-1.00235700	H	-6.09734900	2.34590600	0.67821800
C	-0.24814000	-1.58789200	-0.96857500	H	-3.57986200	0.10465200	3.34203100
H	0.71901700	-1.33792500	-1.38965800	H	-5.11972700	2.01072100	2.93580000
C	-1.45241500	-0.90742800	-1.33611400	C	1.67588800	1.34487500	0.29528900
C	-1.55902200	0.44524700	-1.62058600	C	1.57324400	-0.88488500	0.90619700
H	-2.52825500	0.93397700	-1.64172100	N	2.41732300	0.15965200	0.47607300
C	-0.39654800	1.22566500	-1.54988400	C	3.83083700	0.05103500	0.35115600
C	0.23031600	0.97941400	0.44373900	C	6.59408900	-0.18333100	0.09672600
C	0.17768000	-0.38030800	0.78642700	C	4.65599300	1.08492100	0.79649400
H	0.54522400	0.81842900	-1.90958800	C	4.38016600	-1.09938500	-0.21836100
C	-0.52813700	2.64993900	-1.73187600	C	5.76178400	-1.21389100	-0.33718800
N	-0.67149600	3.79220900	-1.85332700	C	6.03650000	0.96301700	0.65895200
H	-0.45540000	1.73341900	0.80836600	H	4.21880100	1.97661800	1.22829700
C	-1.99164100	-2.96546400	-0.62352600	H	3.72904000	-1.90145900	-0.54718400
H	-2.66649400	-3.77299900	-0.37064100	H	6.18688200	-2.11173100	-0.77408700
H	-0.60004800	-0.84721000	1.37546100	H	6.67701100	1.77041600	0.99881200
C	-4.24355300	-0.43617500	0.04984200	H	7.67088800	-0.27383600	-0.00267100
C	-4.87933800	1.32420100	2.13060700	O	1.94286500	-1.97825300	1.26601200
C	-3.69734100	-0.62110700	1.32223100	O	2.14038600	2.42777800	0.03491300

Adduct (**AD**) **8b/7c-*exo***.

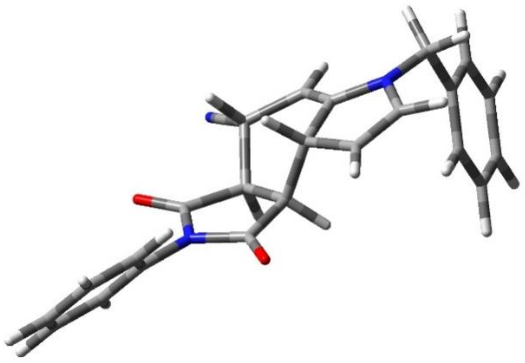

N	-2.55526700	-1.53928400	-1.23523500	C	-5.51771600	0.45763700	-0.22847200
C	-0.72542500	-2.68887700	-0.50941300	C	-6.03650700	1.11269200	0.88741100
H	-0.08130500	-3.49327200	-0.18688300	C	-4.49073600	-0.08241900	2.29873000
C	-3.95813200	-1.20403000	-1.31309100	H	-3.16291900	-1.45095000	1.29950200
H	-4.12230700	-0.58837900	-2.20621700	H	-5.91174500	0.67996400	-1.21769700
H	-4.50917300	-2.13843700	-1.46548900	H	-6.83270300	1.84013400	0.76496600
C	-0.26690800	-1.27805400	-0.79061100	H	-4.08267300	-0.29200300	3.28265700
H	0.44337900	-1.25025300	-1.63439500	H	-5.92073100	1.35701300	3.02396000
C	-1.55785300	-0.57954700	-1.16579400	C	1.97033100	1.23673700	-0.08001100
C	-1.63624500	0.75358800	-1.28195200	C	1.78437800	-0.97251900	0.62937500
H	-2.55773000	1.29476500	-1.46045200	N	2.65506900	0.07394400	0.30250700
C	-0.33066700	1.50755200	-1.12677300	C	4.07964200	-0.03483700	0.31755400
C	0.47775000	0.99949200	0.10669100	C	6.85084500	-0.23647200	0.34684100
C	0.35884600	-0.50449800	0.39230500	C	4.84044700	1.01231000	0.83658900
H	0.29851800	1.36775700	-2.01885100	C	4.69127100	-1.18143300	-0.18794800
C	-0.56932200	2.95295700	-1.01410500	C	6.07993000	-1.27792400	-0.16599200
N	-0.79719800	4.08154900	-0.91545600	C	6.22822900	0.90690300	0.84437900
H	0.16367200	1.57532600	0.98301200	H	4.35050200	1.90285400	1.21277800
C	-2.03569100	-2.76026700	-0.78170000	H	4.08586500	-1.99123200	-0.57757300
H	-2.69996000	-3.61392300	-0.71807400	H	6.55822600	-2.17125000	-0.55395100
H	-0.23525600	-0.71326600	1.28552200	H	6.82236600	1.72293000	1.24228100
C	-4.48981800	-0.47549900	-0.08940200	H	7.93307000	-0.31516200	0.35806900
C	-5.52365100	0.84318800	2.15449900	O	2.12124500	-2.07396200	0.99061400
C	-3.97576000	-0.73786100	1.18248400	O	2.48616100	2.24283900	-0.49629500

Supramolecular complex (**SC**) **8c/7b-*endo***.

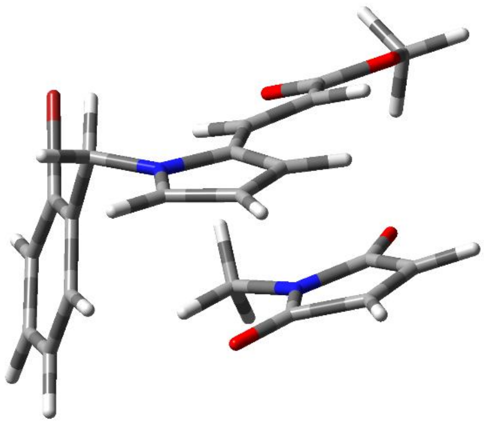

C	0.29397700	1.37484000	-1.68536900	C	0.73624100	0.05427900	-1.31158900
C	1.01045800	2.52294200	-2.01100000	C	1.99214300	-0.41568100	-1.40978100
C	0.08367600	3.56381500	-2.22488200	C	3.38379300	1.66292900	0.93387100
C	-1.17586000	3.02617100	-2.03241600	H	4.42630500	1.71525300	0.64964600
N	-1.04621400	1.70978900	-1.70726300	H	0.00283200	-0.62222500	-0.87155800
H	2.08797000	2.58510900	-2.07353000	C	2.77869000	0.40705600	1.49843400
H	0.29923800	4.58854900	-2.48845000	C	1.15103500	2.00635400	1.40043600
H	-2.15474600	3.47617200	-2.12047100	N	1.44466800	0.69393500	1.75390500
C	-2.15237000	0.78157500	-1.52051100	O	3.32185300	-0.65359900	1.70894900
H	-1.95601300	-0.12005400	-2.10144400	O	0.07853000	2.55337500	1.51832500
H	-3.03821100	1.25593300	-1.95312700	Br	-2.29020300	-2.35405500	-0.76722400
C	-2.43591200	0.43656800	-0.07217400	H	2.80821900	0.14721400	-1.85169100
C	-3.16538700	-0.10565500	2.58806900	C	2.29801900	-1.74000700	-0.83242600
C	-2.54385500	-0.87604000	0.39337700	O	1.49470400	-2.46093700	-0.27577700
C	-2.64606900	1.47131900	0.84503300	O	3.59565900	-2.04784800	-0.97024300
C	-3.00868300	1.21264900	2.16097900	C	4.01189000	-3.22575700	-0.27710000
C	-2.92849300	-1.15278500	1.70431400	H	5.07611200	-3.32696300	-0.48242600
H	-2.50305600	2.49694700	0.51577000	H	3.83277900	-3.09806700	0.79278000
H	-3.16100700	2.03661600	2.84960700	H	3.46194800	-4.09594200	-0.64123300
H	-3.03204100	-2.18364500	2.02432700	C	0.47688500	-0.28993000	2.19981400
H	-3.46030200	-0.32320500	3.60953800	H	0.78452000	-0.70641000	3.16146800
C	2.43406000	2.59662900	0.87799800	H	-0.47995800	0.22327000	2.30428800
H	2.48430800	3.62143900	0.53548700	H	0.39761300	-1.09967200	1.46688900

Transition State (**TS**) **8c/7b-*endo***.

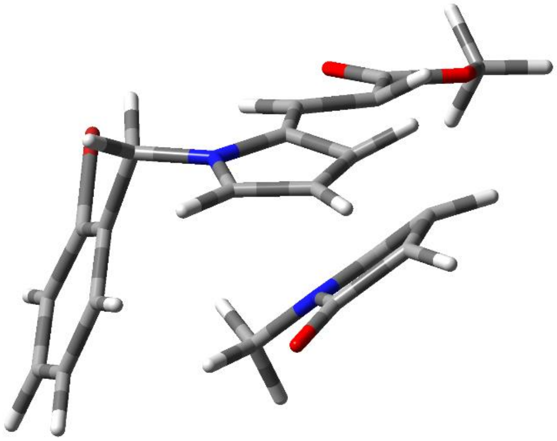

C	0.47371900	1.38414900	-1.50354400	C	1.14461800	0.18892000	-1.30914900
C	1.04268400	2.68809900	-1.31919400	C	2.49065600	0.24813200	-0.92812200
C	-0.02150800	3.63029200	-1.48408200	C	2.52477100	1.32198900	0.85782800
C	-1.16575100	2.90558800	-1.66964500	H	3.60299500	1.39254500	0.93187800
N	-0.88864200	1.56249700	-1.68281900	H	0.62131800	-0.75878300	-1.21809500
H	2.08089300	2.90501100	-1.53126800	C	1.82431000	0.27885000	1.66048200
H	0.04944200	4.70649800	-1.45195300	C	0.34768700	2.01828000	1.35503900
H	-2.18109000	3.24864300	-1.81845900	N	0.50012700	0.68882600	1.76422800
C	-1.86728800	0.49948600	-1.81530800	O	2.27132100	-0.74478800	2.13428400
H	-1.38825800	-0.34249500	-2.31649300	O	-0.67196400	2.66676500	1.47839100
H	-2.65367100	0.86677800	-2.48303400	Br	-1.43874200	-2.58758400	-0.91598100
C	-2.51093800	0.05466000	-0.51429800	H	3.14738400	1.00097300	-1.35331800
C	-3.95783500	-0.72406200	1.77331900	C	3.15268500	-1.04385000	-0.58335000
C	-2.45070800	-1.25327100	-0.03000900	O	2.61476000	-2.12504800	-0.61872500
C	-3.27026200	0.97719900	0.21692900	O	4.42259000	-0.85272000	-0.19374400
C	-3.98501000	0.60417200	1.34787300	C	5.06640600	-2.01615600	0.33388800
C	-3.18837200	-1.65314200	1.08439000	H	6.06632900	-1.69524700	0.62021100
H	-3.29645900	2.01239300	-0.11045400	H	4.50734100	-2.37564800	1.20037400
H	-4.56314400	1.34456500	1.89006600	H	5.11327800	-2.80035900	-0.42431200
H	-3.14295500	-2.68627200	1.41095200	C	-0.49593100	-0.06972700	2.48874800
H	-4.52598500	-1.03697800	2.64321700	H	-0.27115700	-0.07405600	3.55955500
C	1.65119400	2.42357800	0.75708700	H	-1.46345200	0.40177000	2.31558900
H	1.94945500	3.46213100	0.78725700	H	-0.50108300	-1.09807100	2.12281500

Adduct (**AD**) **8c/7b-*endo***.

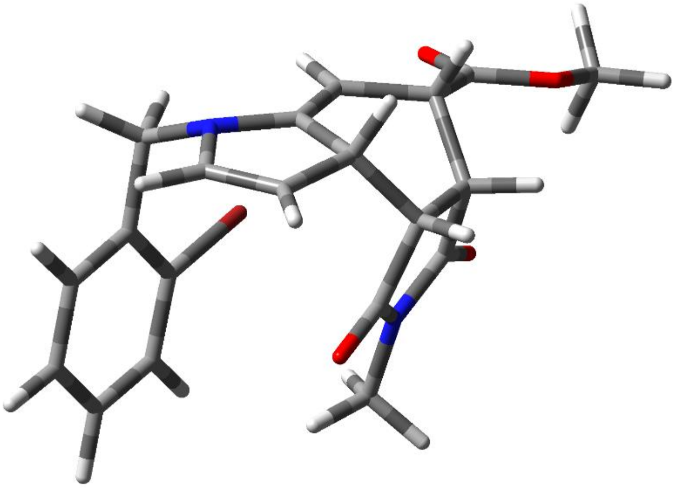

C	0.10889000	1.59367400	-1.26815900	C	1.11739500	0.74753900	-1.50104300
C	0.26938600	2.75111600	-0.30346900	C	2.37524000	0.98024600	-0.71572200
C	-1.11877500	3.33710700	-0.26642900	C	2.05790600	1.25026500	0.78416800
C	-1.89099300	2.64356700	-1.10879600	H	2.97625800	1.57709900	1.27966800
N	-1.19947300	1.60789900	-1.76622000	H	1.07117600	-0.10074800	-2.17154300
H	0.98885500	3.47394100	-0.71747800	C	1.61536700	-0.03750900	1.47385900
H	-1.42275300	4.19099600	0.32076600	C	-0.11375100	1.45179400	1.86039500
H	-2.93409600	2.80800100	-1.34792100	N	0.37187500	0.16398600	2.04138400
C	-1.88788200	0.38700300	-2.17110300	O	2.24278300	-1.07093800	1.54919800
H	-1.15695800	-0.27309000	-2.64159100	O	-1.15172000	1.86085300	2.32582500
H	-2.62747700	0.65337700	-2.93346600	Br	-0.39547600	-2.13972300	-0.67061600
C	-2.60607400	-0.31215100	-1.02806400	H	2.91800800	1.86736600	-1.07974000
C	-4.06180400	-1.46553700	1.09836100	C	3.33994400	-0.18863100	-0.84828500
C	-2.06224600	-1.33865600	-0.24864500	O	3.14660800	-1.17795400	-1.51022000
C	-3.89982900	0.11559900	-0.70340700	O	4.45987000	0.03352400	-0.14632700
C	-4.62352500	-0.43686700	0.34665200	C	5.34999800	-1.08256000	-0.06459000
C	-2.78973100	-1.92910200	0.78399700	H	6.19033100	-0.74641300	0.54002600
H	-4.35527200	0.89481000	-1.30942200	H	4.83557700	-1.91862800	0.41418200
H	-5.62237700	-0.07566400	0.56716300	H	5.68274600	-1.37762400	-1.06154400
H	-2.35443300	-2.75664500	1.33386900	C	-0.29559100	-0.83914700	2.84809800
H	-4.61601100	-1.92333000	1.91121300	H	0.13487800	-0.86460500	3.85305800
C	0.89159500	2.23688100	1.02177400	H	-1.35195500	-0.57584800	2.90334900
H	1.20270900	3.09580000	1.62414800	H	-0.15778400	-1.81333900	2.37530300

Supramolecular complex (**SC**) **8c/7b-*exo***.

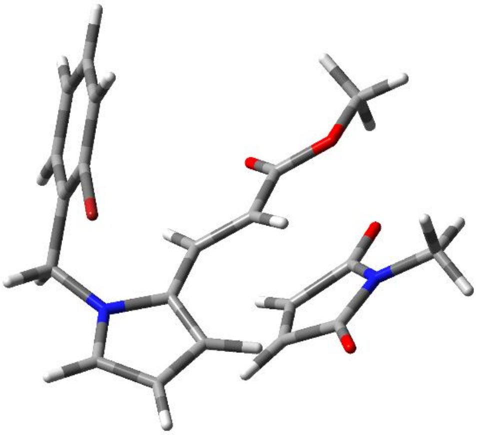

C	0.75080100	-1.42306900	0.33431800	C	-0.83803800	0.22461700	-0.59719400
C	0.53347000	-2.59577200	-0.37460800	C	-3.51141900	-0.67750500	1.88911100
C	1.58392200	-3.48751400	-0.05610200	H	-0.10994400	0.31764600	1.34670000
C	2.41731300	-2.83138900	0.82517000	Br	3.72916800	-0.40282700	-1.17746000
N	1.90541900	-1.59078100	1.07729700	H	-3.51571500	-0.31762100	2.90865100
H	-0.33189000	-2.78861900	-0.99492300	H	-2.10293400	-2.35373400	1.78009100
H	1.71943200	-4.49319100	-0.42539400	H	-0.83460000	-0.22053800	-1.58730000
H	3.35453900	-3.13572800	1.26924200	C	-1.74275300	1.35804500	-0.33923300
C	2.60910700	-0.56242600	1.83474900	O	-1.93112400	1.87647200	0.74389400
H	3.63603000	-0.92096000	1.96176400	C	-4.35064400	-0.02864200	0.82356300
H	2.16599300	-0.46359500	2.83188200	C	-3.15918400	-1.73446800	-0.12745300
C	2.61541600	0.79993400	1.17351900	N	-4.05534000	-0.69904300	-0.36261600
C	2.56570900	3.37413000	0.04121200	O	-2.76429800	-2.51542500	-0.96304500
C	3.03960800	1.01992400	-0.13981600	O	-5.13847900	0.88058700	0.93696500
C	2.16514800	1.90453000	1.90204400	O	-2.37739400	1.75664400	-1.45761400
C	2.13652500	3.18158200	1.35148800	C	-3.31456500	2.82365500	-1.27892700
C	3.02843600	2.29233800	-0.70248400	H	-2.80261800	3.71733400	-0.91570400
H	1.81255900	1.75095200	2.91888100	H	-3.74461100	3.00187100	-2.26363600
H	1.76867500	4.01592100	1.93875200	H	-4.08623200	2.53398400	-0.56098700
H	3.37474900	2.42721900	-1.72112000	C	-4.66590700	-0.43227800	-1.64858200
H	2.54537400	4.36251000	-0.40593300	H	-5.45228900	0.30730300	-1.48949800
C	-2.81889800	-1.67369600	1.33686800	H	-3.92106600	-0.03644100	-2.34300700
C	-0.07131400	-0.23375300	0.40754100	H	-5.09292300	-1.35228100	-2.05282100

Transition State (**TS**) **8c/7b-*exo***.

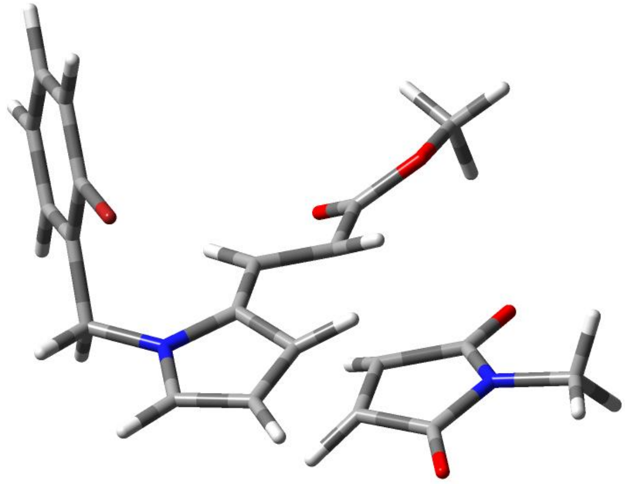

C	0.25524700	-0.69977700	0.38370400	C	-1.29203800	1.02824600	-0.16697900
C	-0.57423700	-1.73823500	-0.14457300	C	-2.65653800	0.06257000	1.07252800
C	0.02629300	-2.98324400	0.22622200	H	0.45951500	1.36165200	1.01973100
C	1.11433300	-2.68028200	0.99652500	Br	3.34042600	-1.38338600	-1.19930300
N	1.26002900	-1.31976100	1.10991100	H	-2.52733900	0.77166200	1.88108800
H	-1.20971400	-1.60651700	-1.01072700	H	-1.85759700	-1.80000600	1.97372000
H	-0.32534200	-3.96926000	-0.03557800	H	-1.64864800	0.52601300	-1.06278900
H	1.83341900	-3.33634800	1.46918300	C	-1.71926500	2.44740100	-0.04022200
C	2.36488100	-0.66158300	1.78535000	O	-1.50023500	3.13799000	0.93173500
H	3.10207500	-1.44190500	2.00374700	C	-3.88169600	0.17929400	0.21609900
H	2.02722700	-0.24530900	2.74177200	C	-3.30540500	-2.04599500	0.29431800
C	3.01405000	0.44763700	0.98228100	N	-4.11499200	-1.08266800	-0.32283800
C	4.22421500	2.57715500	-0.40122300	O	-3.40494300	-3.24098400	0.11728400
C	3.46549100	0.29311100	-0.33059800	O	-4.55667000	1.16096000	-0.00489600
C	3.18461200	1.69854100	1.58186300	O	-2.40680500	2.85272500	-1.11100500
C	3.78545900	2.75696700	0.90780500	C	-3.04164400	4.12683200	-0.98364100
C	4.07045100	1.33990600	-1.01964700	H	-2.30163700	4.90221400	-0.77641900
H	2.82666900	1.84302600	2.59825700	H	-3.53568100	4.30508000	-1.93660300
H	3.89872700	3.71717900	1.39934700	H	-3.77266400	4.08684200	-0.17329200
H	4.41450300	1.18162300	-2.03569800	C	-5.22437500	-1.38605600	-1.20200200
H	4.68920600	3.39345100	-0.94393000	H	-6.17034200	-1.12173700	-0.72277100
C	-2.33096800	-1.30169400	1.13922800	H	-5.13189700	-0.82417500	-2.13354900
C	-0.07132100	0.64982900	0.39459300	H	-5.19670700	-2.45721400	-1.40414500

Adduct (**AD**) **8c/7b-*exo***.

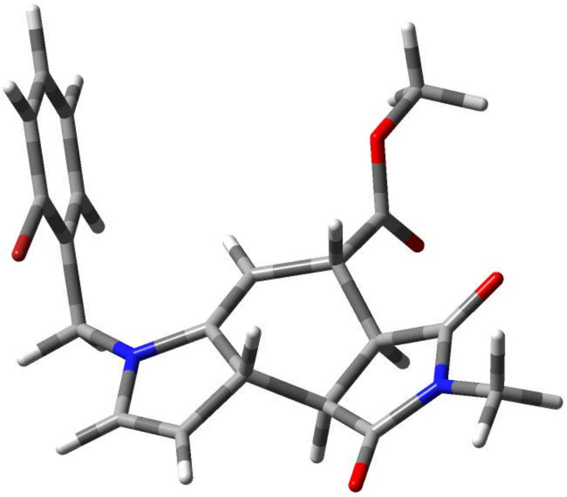

C	0.08139500	-0.52158400	0.72530600	C	-1.52316700	1.14882800	0.01426300
C	-0.91808400	-1.56875500	0.27390500	C	-2.65981300	0.27727800	0.59446100
C	-0.29234600	-2.85704700	0.74866100	H	0.49497300	1.56889500	0.95357800
C	0.89930700	-2.56405000	1.28423800	Br	3.22404600	-1.74779300	-1.02552100
N	1.16762500	-1.18998400	1.26291300	H	-3.00184100	0.76307400	1.51417200
H	-0.99116800	-1.55312100	-0.82706400	H	-2.30836100	-1.44936000	1.91191000
H	-0.72910300	-3.83666000	0.62460900	H	-1.47887700	1.00850700	-1.07521600
H	1.65221800	-3.23131700	1.68577300	C	-1.83226800	2.61371600	0.26206600
C	2.33777200	-0.55663400	1.81489000	O	-2.38236900	3.04696400	1.24627400
H	3.03154000	-1.35542000	2.09954800	C	-3.83514300	0.22384300	-0.36680800
H	2.07622800	0.00036000	2.72658500	C	-3.39718000	-1.99298800	0.15268900
C	3.02266200	0.40005000	0.85689000	N	-4.22058800	-1.10044000	-0.52218100
C	4.28780700	2.24604400	-0.84936400	O	-3.52820400	-3.19597700	0.13955500
C	3.43085900	0.03709000	-0.42770500	O	-4.35783000	1.15441700	-0.93718900
C	3.26704200	1.71405500	1.26333900	O	-1.36506400	3.38599400	-0.72591400
C	3.89497000	2.63402500	0.42887500	C	-1.59429500	4.78803200	-0.56292700
C	4.06392200	0.94015000	-1.27608400	H	-1.10564700	5.14857100	0.34470200
H	2.94841000	2.01785500	2.25780100	H	-1.16771800	5.26002100	-1.44582800
H	4.06938100	3.64782700	0.77347300	H	-2.66592400	4.98521300	-0.49628700
H	4.37528800	0.61881400	-2.26384800	C	-5.33506900	-1.49876500	-1.36076800
H	4.77464500	2.95127300	-1.51488000	H	-6.25124400	-1.02226600	-1.00699800
C	-2.30197100	-1.19908800	0.84766100	H	-5.15311400	-1.19035100	-2.39237700
C	-0.18822800	0.79012700	0.63564400	H	-5.42185000	-2.58337000	-1.29875700

Supramolecular complex (**SC**)**8c/7c-*endo***.

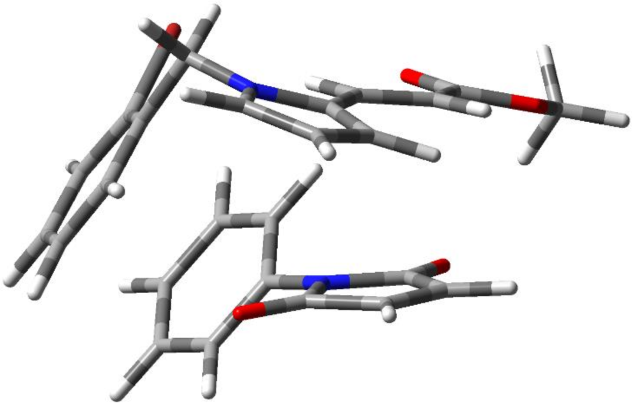

C	1.10282900	2.44890700	-0.48906700	H	0.69593800	0.47863900	-1.34646400
C	1.83401700	3.39601100	0.22569700	C	2.12083400	-0.85181200	1.70977400
C	1.01165400	4.52339200	0.41880100	C	0.52139800	0.65898900	2.37001500
C	-0.20008300	4.24309300	-0.18744900	N	0.74143900	-0.60267800	1.80158900
N	-0.14291800	2.99640900	-0.73029600	O	2.63134100	-1.87131000	1.31467100
H	2.85488000	3.27093500	0.55899800	O	-0.55235400	1.14159200	2.63744300
H	1.26511700	5.43885200	0.93230200	C	-0.25300300	-1.58575700	1.53170100
H	-1.09317400	4.84406400	-0.28631900	C	-2.07551400	-3.62844600	1.06638200
C	-1.22659600	2.36225900	-1.46799600	C	-0.19937800	-2.28963800	0.32968900
H	-0.81005700	1.84425100	-2.33177800	C	-1.22339800	-1.87277500	2.49201900
H	-1.85965200	3.16756600	-1.85258500	C	-2.13699500	-2.89521600	2.25131500
C	-2.06657200	1.42555000	-0.62461000	C	-1.11131200	-3.31956800	0.10818900
C	-3.79102600	-0.17422700	0.91210600	H	0.55566800	-2.04848000	-0.41352400
C	-2.34622900	0.10767800	-0.98835800	H	-1.25728300	-1.29911600	3.41207700
C	-2.64338800	1.90751900	0.55489600	H	-2.89119000	-3.12546900	2.99763000
C	-3.49227900	1.12268200	1.32316100	H	-1.06589200	-3.87379900	-0.82417400
C	-3.22141300	-0.68257300	-0.24847600	H	-2.78110600	-4.43468000	0.88952600
H	-2.40163900	2.91555100	0.88122500	Br	-1.63140600	-0.64837200	-2.57533400
H	-3.90960500	1.51934800	2.24229700	H	3.58152100	1.23093800	-0.60059300
H	-3.43186300	-1.69474600	-0.57275600	C	2.98348800	-0.73843100	-1.36705700
H	-4.45582700	-0.80084400	1.49804300	O	2.15349200	-1.49425000	-1.83129300
C	1.87897200	1.25773400	2.60269300	O	4.26093200	-1.08760600	-1.15576700
H	1.98816700	2.25004700	3.01834600	C	4.55330500	-2.46864700	-1.37888600
C	1.47617700	1.13831800	-0.96317500	H	5.61406100	-2.58291600	-1.16308900
C	2.73041300	0.65066500	-0.94237100	H	3.95097200	-3.08051000	-0.70368800
C	2.80599600	0.38025800	2.22030700	H	4.33655700	-2.74049000	-2.41391300
H	3.88532400	0.45454400	2.23984300				

Transition State (**TS**) **8c/7c-*endo***.

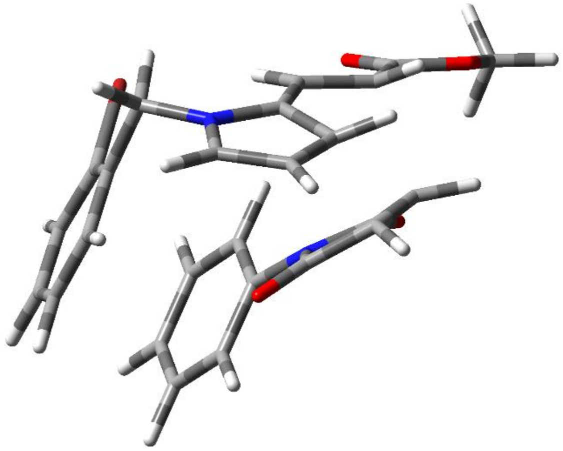

C	-1.15285900	2.19769700	0.67481400	H	-0.98025600	0.25291900	1.61629800
C	-1.81544400	3.02225300	-0.30116700	C	-1.93985900	-0.67175700	-1.52568300
C	-0.92901300	4.12100100	-0.57311900	C	-0.51902900	1.10428800	-1.94166900
C	0.21540300	3.88864200	0.13138000	N	-0.58907100	-0.25835100	-1.60608800
N	0.09986600	2.74168900	0.87851000	O	-2.31543900	-1.82164100	-1.49111200
H	-2.89382900	3.11245200	-0.33720500	O	0.49035900	1.70803400	-2.23350700
H	-1.11858600	4.96470900	-1.21879600	C	0.47867900	-1.19750700	-1.62451400
H	1.12367200	4.47418100	0.18168400	C	2.42633300	-3.17981400	-1.74564000
C	1.15653600	2.15395600	1.68419900	C	0.45481600	-2.24666000	-0.70147400
H	0.69133400	1.60338100	2.50279100	C	1.48210200	-1.11873600	-2.59071900
H	1.71891600	2.98153200	2.12911400	C	2.45453500	-2.11526300	-2.64407400
C	2.11566400	1.26983000	0.91150400	C	1.42633400	-3.23977400	-0.77478200
C	4.07373800	-0.22335900	-0.44946600	H	-0.33245800	-2.28890800	0.04585100
C	2.32140300	-0.08105900	1.19088100	H	1.49566100	-0.28807500	-3.28662900
C	2.88926300	1.83948300	-0.10678600	H	3.23351600	-2.05844300	-3.39849000
C	3.85348600	1.11043000	-0.78791300	H	1.39959100	-4.06153900	-0.06561600
C	3.31035900	-0.81911300	0.54492400	H	3.18056800	-3.95920800	-1.79986700
H	2.72464400	2.87934300	-0.37272100	Br	1.34126400	-0.96982200	2.55047400
H	4.43029900	1.58117600	-1.57681000	H	-3.69184400	1.26253000	0.54673100
H	3.45679000	-1.86013000	0.80745700	C	-3.28270800	-0.84347900	0.98337000
H	4.82898800	-0.80778400	-0.96496500	O	-2.56338900	-1.66405500	1.50737300
C	-1.91898700	1.63673900	-1.85840400	O	-4.52762500	-1.10080900	0.56149900
H	-2.20801800	2.42289200	-2.54319400	C	-4.91224900	-2.47769900	0.63128200
C	-1.62617900	0.97350200	1.12126000	H	-5.92717600	-2.51906500	0.24030100
C	-2.87961400	0.55460100	0.67313900	H	-4.23163300	-3.07197500	0.01803700
C	-2.74945100	0.55986400	-1.49150800	H	-4.88052800	-2.82756600	1.66493000
H	-3.81027000	0.48734400	-1.69549800				

Adduct (**AD**) **8c/7c-*endo***.

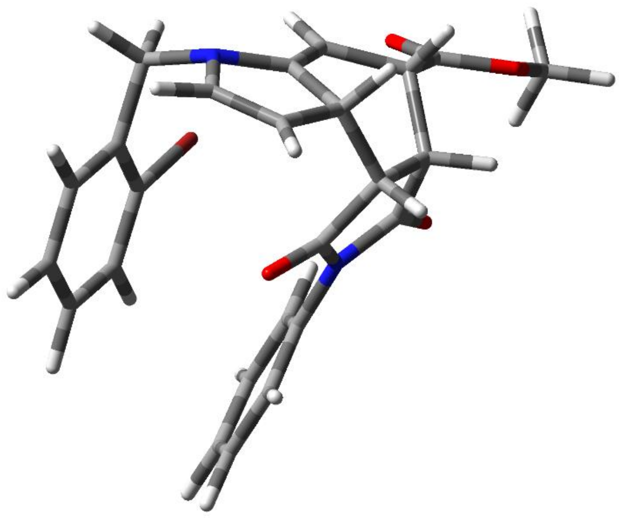

C	0.96970200	-2.40319300	-0.12454100
C	1.05242400	-2.48060900	-1.63450700
C	-0.12390100	-3.35538400	-1.97967200
C	-0.72637400	-3.71700900	-0.84322300
N	-0.10678000	-3.18897000	0.30734100
H	1.99723300	-2.95723300	-1.93865700
H	-0.39710900	-3.67336600	-2.97536700
H	-1.58125000	-4.36959400	-0.71843300
C	-0.91763100	-2.79486900	1.45909800
H	-0.24559400	-2.43929600	2.24187700
H	-1.41850200	-3.69235100	1.83621900
C	-1.96892000	-1.75362900	1.11253100
C	-3.97376400	0.05458800	0.29487300
C	-1.82909000	-0.37555300	1.30490000
C	-3.15537700	-2.19255500	0.51195300
C	-4.14712100	-1.31197700	0.09736500
C	-2.82353800	0.52139200	0.91988100
H	-3.30545100	-3.26054800	0.37776900
H	-5.04917300	-1.69185800	-0.37066600
H	-2.69138200	1.58263100	1.10347800
H	-4.73349000	0.76319400	-0.01916900
C	1.10715200	-1.03303100	-2.17862600
H	1.23263800	-1.05226500	-3.26703600
C	1.82121000	-1.62971200	0.55843100
C	2.84945900	-0.90538700	-0.26221100
C	2.21309600	-0.20046200	-1.50752100
H	3.01908800	0.06824400	-2.19472300
H	1.81018500	-1.49097600	1.63161400
C	1.52780100	1.09462700	-1.09886300
C	-0.17916400	-0.25539100	-1.90752200
N	0.15486300	0.98766800	-1.33741100
O	2.08860900	2.06529200	-0.64606400
O	-1.29761300	-0.61602900	-2.17722300
C	-0.77900800	2.03816000	-1.07691000
C	-2.62494900	4.06434700	-0.54711600
C	-0.55389500	2.92639800	-0.02079500
C	-1.91260200	2.17620800	-1.88280900
C	-2.83245700	3.18406900	-1.60758000
C	-1.47872000	3.93539200	0.23459400
H	0.32969400	2.82885800	0.59330400
H	-2.09015000	1.48453700	-2.69537100
H	-3.71618400	3.27809100	-2.23083400
H	-1.29923800	4.61849400	1.05859400
H	-3.34552900	4.84844500	-0.33744400
Br	-0.35234000	0.34465600	2.25816400
H	3.58871200	-1.61004100	-0.67328700
C	3.63305500	0.10234600	0.56169400
O	3.38842100	0.40789000	1.70249900
O	4.64675500	0.61289200	-0.14982100
C	5.32194700	1.71258900	0.46681700
H	6.10461400	2.00679300	-0.22990100
H	4.61233700	2.52824400	0.62194500
H	5.74847400	1.40765400	1.42424300

Supramolecular complex (**SC**)**8c/7c-*exo***.

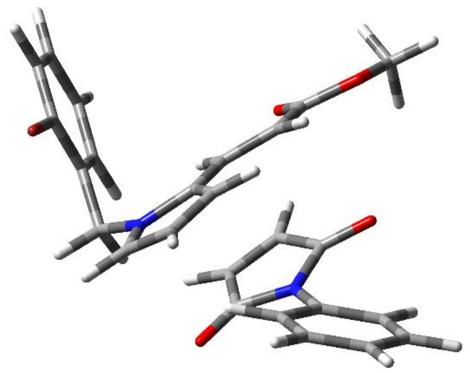

C	0.42247100	-0.89008700	-0.76970900	H	-2.01561300	3.26323400	1.76438700
C	0.12561400	-1.88846300	-1.69009200	H	-0.72937400	1.57863500	3.47604100
C	0.64239700	-3.10209800	-1.19506400	H	-1.11526700	0.60434700	-2.55748000
C	1.24926100	-2.81668900	0.01331500	C	-1.13729400	2.50720100	-1.46817200
N	1.12230900	-1.48471800	0.26891500	O	-0.80814100	3.20599000	-0.52569800
H	-0.44800800	-1.74917900	-2.59552700	C	-2.86429800	1.36436000	0.88011000
H	0.58978900	-4.07508200	-1.66098600	C	-1.73981600	-0.05471500	2.28377800
H	1.80955800	-3.44943200	0.68672600	N	-2.65189300	0.01870500	1.22323600
C	1.76035900	-0.81633600	1.39994300	C	-3.21668900	-1.09901100	0.54394100
H	2.35910300	-1.58152800	1.90404800	C	-4.33297100	-3.27613200	-0.78201000
H	0.99532800	-0.48577000	2.10968300	C	-4.54332500	-1.03954300	0.11157800
C	2.63563800	0.36063200	1.02023100	C	-2.44476400	-2.23948200	0.32049300
C	4.18845600	2.62042500	0.38441500	C	-3.01309900	-3.32569900	-0.33967400
C	3.69105500	0.28130400	0.10874600	C	-5.09335100	-2.12949600	-0.55625900
C	2.38272400	1.60590800	1.60470500	H	-5.12928800	-0.14522900	0.28594000
C	3.14411800	2.72853300	1.29869300	H	-1.41559100	-2.28267400	0.65766000
C	4.46667700	1.39281900	-0.20843600	H	-2.40689200	-4.20910500	-0.51420500
H	1.55349500	1.69263800	2.30237400	H	-6.12249500	-2.08047800	-0.89704400
H	2.91305600	3.68244300	1.76009200	H	-4.76759000	-4.12507000	-1.29995800
H	5.28081400	1.29035800	-0.91718500	O	-1.30743500	-1.06278600	2.79163600
H	4.78852600	3.48732900	0.12835600	O	-3.57799900	1.77100100	-0.00357900
C	-1.39692300	1.35759300	2.65355000	O	-1.95230100	2.93439800	-2.44213600
C	0.01851400	0.49544100	-0.74370800	C	-2.51914800	4.22803000	-2.23931100
C	-0.73675200	1.10652300	-1.67397900	H	-1.73272600	4.97998900	-2.14667800
C	-2.03744700	2.18470900	1.83078700	H	-3.13599800	4.41952800	-3.11550300
H	0.32231300	1.10514400	0.10569500	H	-3.12900100	4.22040800	-1.33267400
Br	4.15718600	-1.37147400	-0.68222400				

Transition State (**TS**) **8c/7c-*exo***.

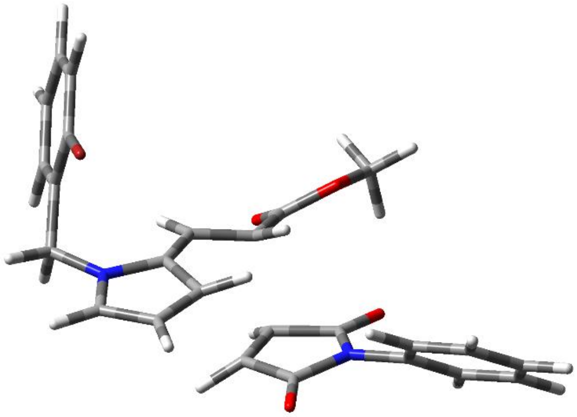

C	-1.09084200	-0.57067300	-0.61804000	H	1.17145000	1.53632300	-2.23351300
C	-0.03909300	-1.50702800	-0.36618500	H	0.87704200	-1.07593800	-2.63217800
C	-0.49296200	-2.78228500	-0.83007900	H	0.80591200	0.77928500	0.72983200
C	-1.72297200	-2.57969500	-1.39189500	C	0.42123800	2.79322600	-0.00490900
N	-2.09082400	-1.26153000	-1.28424900	O	-0.03668600	3.54478200	-0.83861200
H	0.69606400	-1.37327500	0.41717400	C	2.83180500	0.97431100	-0.87378000
H	0.04303100	-3.71682200	-0.76850600	C	2.58448100	-1.29793600	-1.22594900
H	-2.39699700	-3.28706300	-1.85693900	N	3.34928600	-0.29843000	-0.57659100
C	-3.38047200	-0.73092100	-1.69289900	C	4.53494300	-0.55213900	0.16559300
H	-4.02256200	-1.59689900	-1.88795400	C	6.84658200	-1.07206300	1.63145700
H	-3.27831400	-0.17366600	-2.63145500	C	5.64882100	0.27613400	0.01814800
C	-4.03634200	0.17259700	-0.66839400	C	4.57324600	-1.64048200	1.03993000
C	-5.28758800	1.93222700	1.13483400	C	5.73218900	-1.89882700	1.76503000
C	-4.21596100	-0.17416700	0.67279500	C	6.79809500	0.01394500	0.76007500
C	-4.50241100	1.42408600	-1.08113200	H	5.60586200	1.12084500	-0.65806700
C	-5.12613700	2.29990600	-0.19825600	H	3.70631000	-2.28424800	1.13503800
C	-4.83726000	0.68940600	1.57016300	H	5.76079400	-2.74887100	2.43914500
H	-4.36010300	1.71777100	-2.11816700	H	7.66130100	0.66233600	0.64889500
H	-5.47189800	3.26651700	-0.54832200	H	7.74708600	-1.27403700	2.20240700
H	-4.96246700	0.38536300	2.60345100	O	2.86952200	-2.47275600	-1.25800500
H	-5.76479400	2.60601500	1.83868100	O	3.34786900	2.03177600	-0.59386800
C	1.39662400	-0.61910800	-1.80136100	O	1.17269400	3.18164000	1.02729200
C	-0.97801600	0.80526300	-0.47393100	C	1.56694900	4.55529300	1.01496200
C	0.24471200	1.31557300	-0.03148300	H	0.68849400	5.20353900	1.01536900
C	1.52386900	0.75828800	-1.56712600	H	2.15741500	4.69881300	1.91750300
H	-1.70099600	1.48717400	-0.91164900	H	2.16976200	4.75071500	0.12554400
Br	-3.67799500	-1.87306500	1.30913600				

Adduct (**AD**) **8c/7c-*exo***.

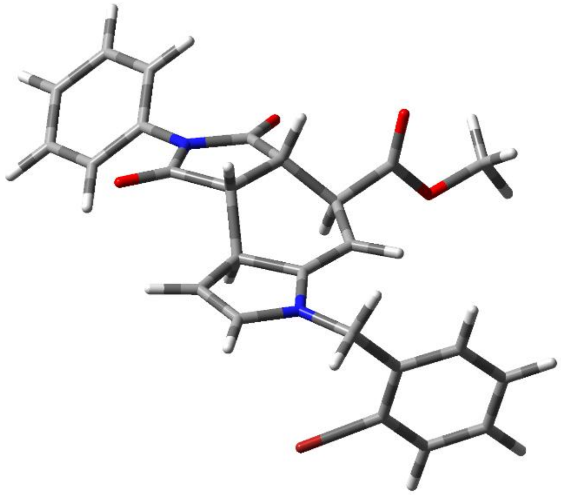

C	1.00571100	-0.31485700	0.90728300	H	-1.69371200	1.61128800	1.89140200
C	-0.21877500	-1.19296800	0.73627500	H	-1.27836500	-0.61930600	2.51928300
C	0.23960400	-2.51997000	1.28902000	H	-0.51247400	1.31122300	-0.85784600
C	1.53282800	-2.40564500	1.61663000	C	-0.38163300	3.06610800	0.31742900
N	2.02984500	-1.11898300	1.37688000	O	-0.76950200	3.69018000	1.27610900
H	-0.46124900	-1.27778400	-0.33712700	C	-2.82984400	1.01293400	0.20559600
H	-0.37471900	-3.40513100	1.35854000	C	-2.71580400	-1.15862200	1.03563000
H	2.21094500	-3.15851500	1.99965900	N	-3.47334700	-0.22922400	0.30995600
C	3.35881100	-0.66255000	1.69472900	C	-4.73707700	-0.51879500	-0.28745100
H	3.93810300	-1.54289800	1.99425000	C	-7.20236100	-1.07211300	-1.44761600
H	3.33043400	0.02710100	2.55117300	C	-5.76469300	0.42072900	-0.20417400
C	4.05272300	0.04105200	0.54368400	C	-4.93132500	-1.73238100	-0.94654500
C	5.35567600	1.42496900	-1.53119100	C	-6.16971700	-2.00489300	-1.52149800
C	4.20924200	-0.52958700	-0.72051400	C	-6.99510000	0.13961900	-0.79083400
C	4.56980900	1.32385200	0.74034400	H	-5.59381200	1.36450600	0.30038800
C	5.21859200	2.01587200	-0.27796900	H	-4.12726000	-2.45705500	-0.99301500
C	4.85818100	0.14351000	-1.75097300	H	-6.32425700	-2.95094000	-2.03001000
H	4.45124700	1.78762900	1.71689600	H	-7.79370700	0.87180700	-0.73107300
H	5.60800600	3.01168300	-0.09441600	H	-8.16468300	-1.28828300	-1.90026400
H	4.96857900	-0.33561900	-2.71752000	O	-3.04354900	-2.29940100	1.25852500
H	5.85485200	1.95160500	-2.33785500	O	-3.22951300	1.94988200	-0.44206400
C	-1.40674300	-0.50349600	1.43993500	O	0.15495900	3.63495800	-0.76858100
C	0.95728400	1.00794600	0.68602500	C	0.21377700	5.06351000	-0.74280300
C	-0.37313900	1.55251500	0.20566900	H	0.82691800	5.40214100	0.09503400
C	-1.54890200	0.96573500	1.01838500	H	0.65909400	5.35625300	-1.69159300
H	1.80799300	1.66919700	0.80095700	H	-0.79159700	5.47664300	-0.64172900
Br	3.62520800	-2.30136800	-1.04529400				

Supramolecular complex (**SC**) **8g/7c-*endo***.

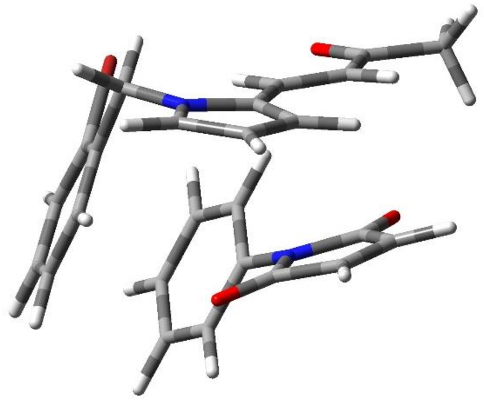

C	-2.05396700	1.63511700	0.50713100	H	-4.04661800	-1.50357900	-1.75310000
C	-3.18891400	2.09109000	-0.16250200	H	-0.77325400	0.18351300	1.51732300
C	-2.94868600	3.41606900	-0.57419400	C	-1.86441000	-1.91262600	-1.33044600
C	-1.67628200	3.74454600	-0.14004000	C	-1.08850000	0.02010000	-2.29737000
N	-1.14171000	2.67490600	0.50832800	N	-0.72323400	-1.14457300	-1.61049900
H	-4.08978700	1.51479200	-0.32084200	O	-1.87972800	-2.99370900	-0.79398500
H	-3.61902200	4.06586200	-1.11686100	O	-0.33609900	0.85679800	-2.73393400
H	-1.11886200	4.66635000	-0.23007900	C	0.60662400	-1.60398100	-1.38678600
C	0.17053200	2.66458900	1.14148500	C	3.13566900	-2.67798700	-0.99105400
H	0.09517600	2.15327700	2.10100500	C	0.94984900	-2.10015600	-0.13054100
H	0.42355900	3.70754800	1.35446500	C	1.52200800	-1.61156600	-2.43954600
C	1.25841800	2.05305100	0.28371600	C	2.79002700	-2.14787300	-2.23403500
C	3.37051500	1.11407300	-1.31353500	C	2.21763800	-2.64705300	0.05728500
C	2.10568100	1.03776600	0.72972600	H	0.23331500	-2.07681500	0.68587400
C	1.47345900	2.55741700	-1.00296200	H	1.23764500	-1.20312700	-3.40352900
C	2.51051900	2.09510300	-1.80159000	H	3.50438000	-2.16092700	-3.05153800
C	3.17236200	0.58759600	-0.04337900	H	2.48223300	-3.04169600	1.03332100
H	0.79655000	3.31569400	-1.38752200	H	4.12089800	-3.10888200	-0.83979700
H	2.63926400	2.49278500	-2.80240700	Br	1.91301800	0.27961500	2.45794600
H	3.82348600	-0.18677300	0.34433600	H	-3.71927800	-0.51072700	1.03502700
H	4.18963100	0.74438300	-1.92212200	C	-2.27438800	-1.87348700	2.01718600
C	-2.58655300	-0.00975600	-2.40822100	C	-3.33082100	-2.94174100	2.15126900
H	-3.12811100	0.79689900	-2.88309500	H	-4.26256100	-2.52377300	2.54445500
C	-1.78353200	0.37341900	1.15042400	H	-2.96853000	-3.73954100	2.79901500
C	-2.68114500	-0.61405900	1.34376300	H	-3.53172300	-3.34922700	1.15384600
C	-3.03424400	-1.13436400	-1.85227500	O	-1.13674200	-2.04141200	2.42645600

Transition State (**TS**) **8g/7c-*endo***.

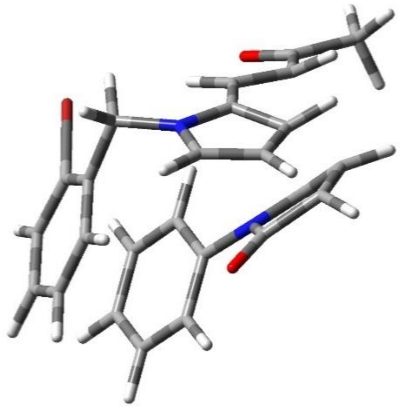

C	-1.77409200	1.73803300	0.63832200	H	-4.01916900	-0.71721500	-1.51198200
C	-2.67550300	2.28752000	-0.34311900	H	-1.04780700	-0.02154400	1.67542100
C	-2.12552600	3.56338100	-0.72496800	C	-1.91035200	-1.36332900	-1.36991000
C	-0.93224400	3.68882700	-0.07944900	C	-0.99943300	0.68509100	-1.93440000
N	-0.70484300	2.60416000	0.73449700	N	-0.71155100	-0.62575200	-1.52968800
H	-3.74202300	2.10326000	-0.29963400	O	-1.97694600	-2.56792300	-1.27367600
H	-2.56073200	4.28101300	-1.40350700	O	-0.19055500	1.50429500	-2.31215600
H	-0.21083000	4.49418800	-0.11577600	C	0.55910800	-1.26471000	-1.53886600
C	0.50364900	2.37300700	1.50788900	C	2.93786100	-2.70131500	-1.62194300
H	0.23609000	1.78219900	2.38483800	C	0.83122300	-2.20671300	-0.54338000
H	0.84588700	3.34928500	1.86682800	C	1.47712600	-1.01588800	-2.55925800
C	1.62728800	1.71409000	0.73229900	C	2.66718600	-1.73996900	-2.59347600
C	3.84601800	0.68315200	-0.65610400	C	2.01919400	-2.92864500	-0.59692800
C	2.19497200	0.48869800	1.08131500	H	0.10550600	-2.38173100	0.24620200
C	2.17559700	2.38623500	-0.36638200	H	1.25657700	-0.26711700	-3.31122300
C	3.26662100	1.88404500	-1.06105800	H	3.38127600	-1.55288500	-3.38986900
C	3.31338500	-0.01315300	0.42011100	H	2.22449100	-3.67020800	0.16903600
H	1.72984100	3.32366400	-0.68537400	H	3.86197800	-3.27033000	-1.66119000
H	3.66232200	2.42728100	-1.91251800	Br	1.54908700	-0.51664200	2.55453700
H	3.74144100	-0.95710800	0.73619400	H	-3.97096900	0.18471100	0.73155100
H	4.70457100	0.27787600	-1.18180900	C	-3.02763100	-1.72854500	1.30382000
C	-2.48741100	0.85746500	-1.79923900	C	-4.28035300	-2.52395300	1.03693200
H	-2.98875000	1.49308700	-2.51792200	H	-5.18323500	-1.91311200	1.11879800
C	-1.88436700	0.46724400	1.18198400	H	-4.32745000	-3.37224500	1.72016400
C	-3.00617500	-0.29668100	0.86277300	H	-4.20464400	-2.90889800	0.01308500
C	-3.00209500	-0.37845200	-1.35777100	O	-2.04274000	-2.23426900	1.80721800

Adduct (**AD**) **8g/7c-*endo***.

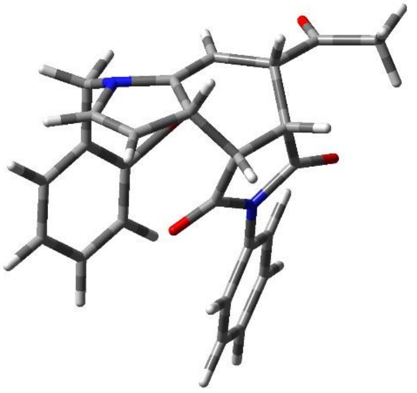

C	-1.84007200	1.82133600	-0.13061200	H	-3.00871900	-1.27475700	-2.04677300
C	-1.99338300	1.80629900	-1.63697300	H	-2.28039000	0.75148100	1.68364900
C	-1.18288300	3.00319900	-2.05913000	C	-1.23978100	-1.69347700	-0.96223200
C	-0.69579700	3.58842600	-0.96079000	C	-0.10514700	0.10442900	-1.89116900
N	-1.06893100	2.93423400	0.22962800	N	0.01066900	-1.14935100	-1.26057400
H	-3.05207300	1.93257800	-1.91298000	O	-1.43236600	-2.77520400	-0.45677000
H	-1.06269600	3.35258400	-3.07432400	O	0.82078000	0.80155400	-2.22242000
H	-0.10003900	4.48993500	-0.89266900	C	1.24823000	-1.82203200	-1.01328700
C	-0.14367300	2.88232600	1.36049500	C	3.67965900	-3.10047900	-0.51636600
H	-0.63922000	2.35979900	2.18040000	C	1.36666100	-2.69383800	0.07332200
H	0.04118900	3.91061400	1.68763100	C	2.33528700	-1.60817800	-1.86558400
C	1.18363100	2.23254800	1.00625800	C	3.54646900	-2.24327100	-1.60708400
C	3.65056100	1.15312200	0.16557200	C	2.58284300	-3.32858400	0.31225300
C	1.51327500	0.89518800	1.24742800	H	0.52259000	-2.87130500	0.72409700
C	2.14098900	3.01205700	0.34534500	H	2.24606300	-0.92873000	-2.70240500
C	3.35641400	2.49071200	-0.08115300	H	4.38930500	-2.06338300	-2.26701700
C	2.73748500	0.36065200	0.85103700	H	2.66843800	-4.00065300	1.16003300
H	1.92536400	4.06302300	0.17189100	H	4.62664400	-3.59273000	-0.31914500
H	4.06872100	3.12640900	-0.59648600	Br	0.38703100	-0.22777100	2.28550200
H	2.96915700	-0.67590500	1.07311500	H	-4.05120500	0.23707000	-0.57529100
H	4.59302500	0.72181800	-0.15636200	C	-3.62647000	-1.35204700	0.74435800
C	-1.58405200	0.39917000	-2.13416100	C	-4.51272100	-2.39546900	0.10286700
H	-1.73970900	0.32846000	-3.21654600	H	-5.17292000	-1.95789800	-0.65186300
C	-2.36681400	0.83805600	0.60854000	H	-5.09984100	-2.89135400	0.87627900
C	-3.13459300	-0.20689300	-0.14826400	H	-3.86952800	-3.13535700	-0.38312700
C	-2.33111600	-0.72284700	-1.38794300	O	-3.34121700	-1.39213900	1.91988600

Supramolecular Complex (**SC**) **8g/7c-*exo***.

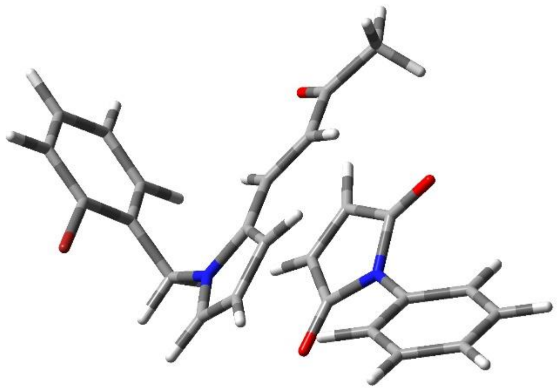

C	-0.25983600	-0.86013400	0.70141600	Br	-3.86900100	-1.67938800	0.54630400
C	0.14555200	-1.91063400	1.51827700	H	1.82625300	3.79172700	-0.98135100
C	-0.21242900	-3.11669800	0.88600500	H	0.74897100	2.33521100	-3.01117200
C	-0.83551400	-2.77724800	-0.30029500	H	1.01367200	0.57859300	2.69831100
N	-0.86814100	-1.42040000	-0.41185400	C	0.74300800	2.62436300	1.91229100
H	0.68042000	-1.80215000	2.45116000	O	0.30625300	3.34250900	1.02258300
H	-0.04589600	-4.12060600	1.24786200	C	2.80908200	1.84098000	-0.39536800
H	-1.30458300	-3.39686400	-1.05124100	C	1.86180000	0.60477000	-2.07419300
C	-1.55899700	-0.72363200	-1.49406300	N	2.73174900	0.56513900	-0.97681500
H	-2.06871600	-1.50026900	-2.07328700	C	3.38986400	-0.59940000	-0.48763700
H	-0.82352000	-0.25504600	-2.15563600	C	4.69250500	-2.86830000	0.46066300
C	-2.56026800	0.31793400	-1.03904000	C	4.69741600	-0.49658100	-0.00743200
C	-4.35745100	2.34345800	-0.27151500	C	2.73095400	-1.82850200	-0.50161100
C	-3.59547600	0.06314000	-0.13664400	C	3.39184100	-2.95939600	-0.02922400
C	-2.45362900	1.61664300	-1.54665500	C	5.34035800	-1.63360200	0.47231800
C	-3.33689700	2.62455200	-1.17575500	H	5.19613500	0.46521300	0.00065200
C	-4.49318000	1.05745700	0.24221500	H	1.71686000	-1.90348100	-0.87631300
H	-1.64532600	1.83709200	-2.23917000	H	2.87306700	-3.91284400	-0.03837000
H	-3.21799300	3.62470400	-1.57792600	H	6.35425200	-1.55205200	0.85059500
H	-5.28875800	0.81993700	0.93956800	H	5.19960100	-3.75328000	0.83151500
H	-5.05119800	3.11918700	0.03517900	O	1.53433000	-0.33182000	-2.76497000
C	1.40911200	2.02719000	-2.21139000	O	3.44730700	2.13887400	0.58466300
C	-0.05964800	0.56026200	0.85750000	C	1.51539400	3.19318800	3.07612100
C	0.58124400	1.15398000	1.88380700	H	1.12815400	2.80848900	4.02451400
C	1.94816500	2.74698300	-1.23035500	H	1.46531200	4.28162900	3.05927900
H	-0.44703500	1.22656000	0.08782200	H	2.55916300	2.87249200	2.98322800

Transition State (**TS**) **8g/7c-*exo***.

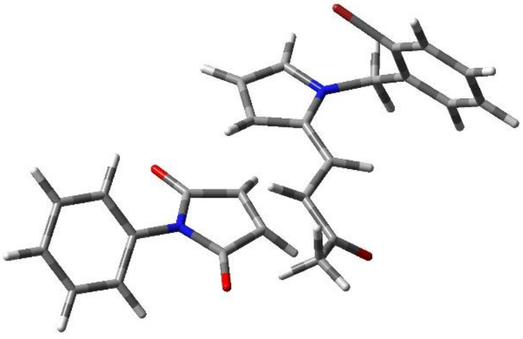

C	0.98830300	-0.36498700	0.63889700	Br	3.45989400	-1.97285700	-1.19008700
C	-0.12306500	-1.24696200	0.45686800	H	-1.23534400	2.03046600	1.99805800
C	0.24801800	-2.51122000	1.01548900	H	-0.95768300	-0.52113900	2.69769100
C	1.49045100	-2.34725100	1.56269700	H	-0.78427300	1.03496800	-0.84554300
N	1.94356200	-1.06847400	1.35641700	C	-0.21693200	3.07686900	-0.22135800
H	-0.84973100	-1.12246100	-0.33668700	O	0.31129400	3.75282100	0.64116400
H	-0.34920600	-3.40987600	1.02755700	C	-2.84988400	1.31533500	0.64624400
H	2.11706600	-3.06001300	2.08256100	C	-2.67843900	-0.88171500	1.34163000
C	3.26754300	-0.59472800	1.72292300	N	-3.40323600	0.03167000	0.53572400
H	3.85143100	-1.48375500	1.98552500	C	-4.58040100	-0.29707800	-0.19080900
H	3.20694100	0.03952900	2.61497400	C	-6.87752300	-0.96449400	-1.61866600
C	3.97872600	0.18069300	0.63211000	C	-5.67032900	0.57513300	-0.19532500
C	5.34311200	1.70729000	-1.29745200	C	-4.63568800	-1.50273900	-0.89320700
C	4.12458200	-0.27581400	-0.67997700	C	-5.78760200	-1.83347000	-1.60023800
C	4.53637000	1.42227300	0.95046300	C	-6.81226500	0.23786100	-0.91776000
C	5.21630300	2.18312400	0.00482700	H	-5.61418500	1.50990200	0.34866800
C	4.80103300	0.47195400	-1.63925400	H	-3.78855600	-2.17883100	-0.86831300
H	4.42119000	1.80169200	1.96270300	H	-5.83029300	-2.77404900	-2.13992700
H	5.63286200	3.14534700	0.28229400	H	-7.65660700	0.91967500	-0.92529200
H	4.89789300	0.08397700	-2.64713400	H	-7.77249900	-1.22417900	-2.17475700
H	5.86413600	2.29057900	-2.04940400	O	-3.00378500	-2.02783200	1.54578900
C	-1.47595600	-0.15906900	1.82028800	O	-3.31898800	2.32432200	0.16622000
C	0.96655000	1.00025600	0.39732900	C	-0.88759800	3.67305500	-1.42958000
C	-0.20768900	1.57922600	-0.09823000	H	-0.48274500	3.22677300	-2.34367800
C	-1.57307900	1.18183500	1.41512100	H	-0.73939700	4.75269100	-1.43720400
H	1.73206500	1.65778000	0.79905000	H	-1.95706900	3.44176600	-1.38665800

Adduct (**AD**) **8g/7c-*exo***.

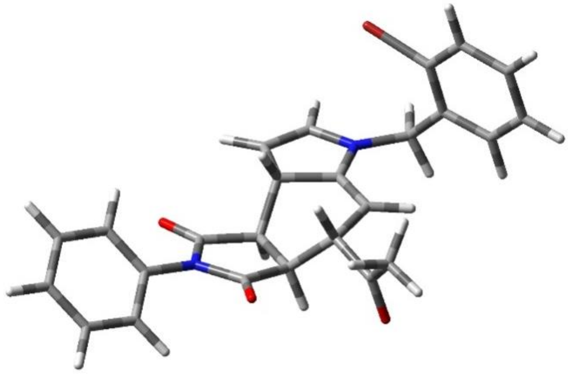

C	-1.02353600	-0.11789500	-0.88510600	Br	-3.68684300	-2.13651700	0.97855400
C	0.18689000	-1.02134800	-0.74442800	H	1.65074100	1.79962500	-1.84799500
C	-0.29391700	-2.32158600	-1.33993400	H	1.25649200	-0.42322100	-2.51515900
C	-1.58316600	-2.17288400	-1.67025900	H	0.54213600	1.36900500	0.91714000
N	-2.05789000	-0.88618800	-1.39096300	C	0.42196000	3.22736700	-0.14963800
H	0.43409600	-1.14465600	0.32382900	O	0.54555200	3.81932500	-1.19918000
H	0.30397800	-3.21558400	-1.43507800	C	2.83203200	1.16188800	-0.19657300
H	-2.27253500	-2.90020200	-2.08157200	C	2.68802300	-1.00093300	-1.04105000
C	-3.37891600	-0.39523100	-1.69124400	N	3.46305100	-0.08276700	-0.31562600
H	-3.97390200	-1.25337900	-2.02264200	C	4.72718800	-0.39010300	0.27239800
H	-3.33838300	0.32500200	-2.52137000	C	7.19236500	-0.97578600	1.41616400
C	-4.05860700	0.27762900	-0.51342500	C	5.76430300	0.53875500	0.18812600
C	-5.32711700	1.60749700	1.61764600	C	4.91175300	-1.60927100	0.92393000
C	-4.22985100	-0.34025400	0.72647200	C	6.15026800	-1.89792900	1.49072600
C	-4.54284400	1.58015600	-0.65679800	C	6.99474200	0.24152900	0.76673000
C	-5.17435800	2.24594400	0.38962100	H	5.60098000	1.48670000	-0.31119400
C	-4.86190300	0.30595900	1.78434400	H	4.10036100	-2.32571300	0.97067700
H	-4.41167300	2.08101000	-1.61319700	H	6.29754600	-2.84829700	1.99328200
H	-5.53821400	3.25800300	0.24717300	H	7.80093300	0.96527100	0.70620100
H	-4.98529100	-0.20986000	2.73026500	H	8.15473500	-1.20463200	1.86239600
H	-5.81428100	2.11260200	2.44521600	O	3.00247200	-2.14376800	-1.27007700
C	1.38430300	-0.32818400	-1.43366200	O	3.24237600	2.09161300	0.45826300
C	-0.95216400	1.19639700	-0.62058200	C	0.26780100	3.92917100	1.17258600
C	0.38683600	1.69682500	-0.12265300	H	-0.59679300	3.53030200	1.71392600
C	1.53883700	1.13201300	-0.98659400	H	0.16487000	5.00347900	1.02241700
H	-1.78125900	1.88187600	-0.75299900	H	1.16008500	3.71774400	1.77257500

Supramolecular complex (**SC**) **8j/7c-*endo***.

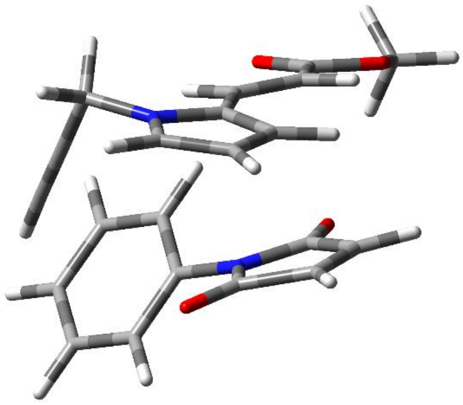

C	-2.45613100	0.39206400	-0.51325900	C	2.01640500	-2.46923600	1.13236500
C	-3.33756000	0.79138500	0.48956600	C	2.90839900	-3.29571800	0.45290300
C	-4.34523400	-0.18760400	0.59805700	C	3.24298500	-1.57910200	-1.21030400
C	-4.06257400	-1.16005800	-0.34348300	H	2.09898900	0.23409800	-0.94608700
N	-2.93137600	-0.80622100	-1.01122900	H	1.52655400	-2.80749700	2.03824200
H	-3.24366300	1.69570900	1.07457100	H	3.12447200	-4.28474300	0.84425300
H	-5.18074400	-0.19467200	1.28191800	H	3.71525700	-1.22914200	-2.12245000
H	-4.58182900	-2.07516100	-0.58913900	H	4.22637400	-3.49669800	-1.24022900
C	-0.97679900	0.43061000	2.55704700	H	-1.40173200	2.89310800	-0.00570700
H	-1.92236300	0.37836000	3.07899600	C	0.50760500	2.70791200	-1.07234400
C	-1.23857300	1.00208300	-0.98908000	O	1.25779800	2.11670100	-1.82305700
C	-0.81844600	2.23225700	-0.63883300	O	0.81077900	3.88529000	-0.50586400
C	-0.14668400	1.45850600	2.38359400	C	2.14583000	4.34086500	-0.73692500
H	-0.22408800	2.48167100	2.72704400	H	2.22463600	5.29422600	-0.21754700
H	-0.58357300	0.41582500	-1.63561200	H	2.85233900	3.61554700	-0.32741600
C	1.03395500	0.99608100	1.58313400	H	2.32444800	4.46604900	-1.80674100
C	-0.40333700	-0.77662400	1.87219000	C	-2.36771700	-1.55040600	-2.13108700
N	0.81385200	-0.36089800	1.30419700	H	-3.11886000	-2.28010200	-2.44387300
O	1.99891500	1.64224700	1.25243500	H	-2.21451100	-0.86441800	-2.97094300
O	-0.86759400	-1.88830700	1.82656700	C	-1.11134900	-2.23036500	-1.80597600
C	1.73358000	-1.20206000	0.62075300	C	-0.06656500	-2.77722900	-1.55787200
C	3.52637100	-2.85277400	-0.71727500	H	0.86317700	-3.25061700	-1.31802100
C	2.33894000	-0.74878100	-0.55195000				

Transition State (**TS**) **8j/7c-*endo***.

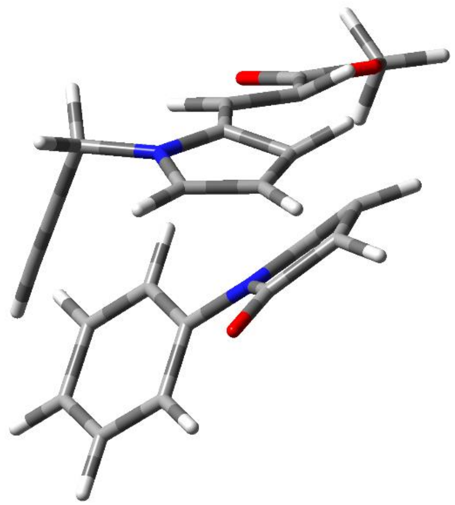

C	-0.67612400	2.19380300	0.61242100	C	2.76077000	-1.36659600	-1.23336300
C	-1.02641500	2.83433700	-0.62711400	C	3.78454400	-2.18567100	-0.75968300
C	-0.03488300	3.84788800	-0.85595100	C	2.30590000	-3.08861800	0.91823500
C	0.88542000	3.73874900	0.14585800	H	0.30530200	-2.27710100	0.94535200
N	0.50325500	2.77018000	1.04061100	H	2.93139400	-0.68646000	-2.05842500
H	-2.05243000	2.91340600	-0.96321500	H	4.75813600	-2.14960400	-1.23824700
H	0.00326000	4.54477500	-1.67897800	H	2.12370600	-3.75551900	1.75479000
H	1.80505200	4.28499000	0.30704800	H	4.36172200	-3.69125700	0.67019700
C	-0.80592900	1.18059700	-1.92839100	H	-3.11045900	1.18182300	-0.01918100
H	-0.94571500	1.83840300	-2.77548000	C	-2.87730800	-0.80714900	0.86514100
C	-1.27795700	1.05252300	1.11870200	O	-2.35426400	-1.49665100	1.71090400
C	-2.37372500	0.52496700	0.43153500	O	-3.95780700	-1.17446500	0.16393300
C	-1.68904600	0.14679500	-1.56666800	C	-4.37669300	-2.52730000	0.37285400
H	-2.66602700	-0.02220700	-2.00195100	H	-5.24017000	-2.67051300	-0.27401700
H	-0.79251800	0.44063600	1.87566400	H	-3.56345800	-3.20149900	0.09607900
C	-0.87735200	-1.03090300	-1.18798000	H	-4.64617700	-2.68257500	1.41928000
C	0.57902700	0.71719800	-1.60841200	C	1.26566800	2.34968500	2.20240500
N	0.45216200	-0.57676800	-1.06244600	H	1.85262000	3.20808300	2.54187700
O	-1.24798400	-2.16956500	-1.01104900	H	0.56062100	2.10505500	3.00393700
O	1.61880100	1.31429300	-1.77405600	C	2.14782200	1.20848600	1.93762000
C	1.51212600	-1.40653700	-0.61134100	C	2.87433400	0.27268100	1.71656800
C	3.56274300	-3.04973100	0.31206500	H	3.50018300	-0.56768400	1.49620700
C	1.27992300	-2.26445900	0.46784100				

Adduct (**AD**) **8j/7c-*endo***.

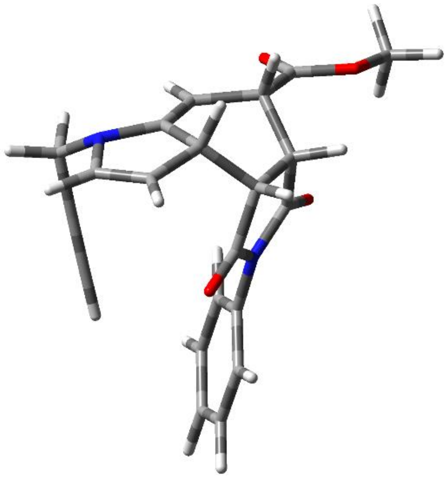

C	-1.18402200	2.05568400	0.34074800
C	-1.10329800	2.24860400	-1.16064000
C	-0.13008700	3.39346500	-1.28532900
C	0.21632600	3.79169000	-0.05859200
N	-0.44373300	3.06917500	0.95845000
H	-2.09231200	2.51883900	-1.56112700
H	0.21302100	3.82199800	-2.21544100
H	0.89452400	4.58518900	0.23070400
C	-0.72139800	0.89591400	-1.82980200
H	-0.84458700	0.99116100	-2.91263600
C	-1.82804700	1.00684200	0.86826400
C	-2.50515700	0.10298000	-0.12397200
C	-1.51773700	-0.29271700	-1.26016500
H	-2.06431900	-0.83952900	-2.03266500
H	-1.87422700	0.76486200	1.92380000
C	-0.46465600	-1.23008200	-0.68488900
C	0.74095000	0.54181100	-1.57790000
N	0.79910600	-0.67368900	-0.87940600
O	-0.68738600	-2.28509000	-0.13887300
O	1.69879100	1.18065500	-1.93567900
C	2.00406100	-1.26492200	-0.39586300
C	4.35995200	-2.36805800	0.59339600
C	2.00934000	-1.86760300	0.86418500
C	3.16563400	-1.21534400	-1.16704200
C	4.34301100	-1.76355700	-0.66202200
C	3.18934100	-2.42224800	1.34996900
H	1.10192700	-1.89026000	1.45522200
H	3.15188800	-0.73521200	-2.13716900
H	5.24878100	-1.71761600	-1.25786600
H	3.19279400	-2.88990200	2.32920600
H	5.27926000	-2.79644300	0.97968500
H	-3.34616800	0.61847900	-0.61416700
C	-3.10335900	-1.12992700	0.53371100
O	-3.10829600	-1.35112500	1.71878200
O	-3.66684200	-1.93316000	-0.37941300
C	-4.12719900	-3.18959500	0.12512800
H	-4.54506800	-3.71626500	-0.73075300
H	-3.28374900	-3.74064600	0.54664500
H	-4.88599800	-3.03540000	0.89460100
C	0.23798200	2.80917500	2.21706400
H	0.55389200	3.76654000	2.64285100
H	-0.47625000	2.36777400	2.91715900
C	1.40716000	1.92929300	2.04068200
C	2.37388700	1.24242600	1.81592600
H	3.22276700	0.62479400	1.61298200

Supramolecular complex (**SC**) **8j/7c-*exo*** M06-2X/6-31+G(d,p).

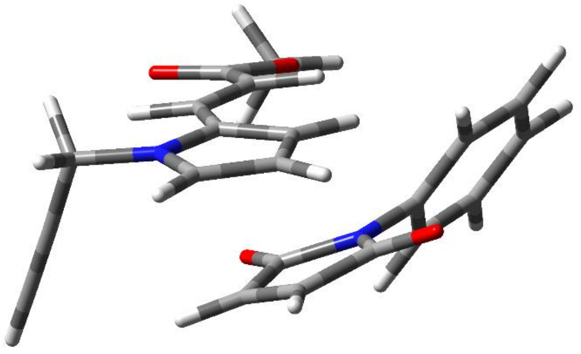

C	2.68003700	-0.49911600	-0.98127700	C	-3.73166100	-0.06597800	1.20433800
C	2.45389800	-1.86392800	-1.11626700	C	-2.94048100	-1.36271600	-0.68372200
C	3.66293300	-2.53370500	-0.82800100	C	-4.23787800	-1.41153000	-1.18368900
C	4.60067800	-1.56647500	-0.52247800	C	-5.02424500	-0.11223500	0.68712100
N	4.00636900	-0.34232200	-0.62143400	H	-3.52627800	0.45985500	2.12912200
H	1.50872400	-2.32240600	-1.37337000	H	-2.12638100	-1.84197000	-1.21349900
H	3.83530300	-3.59952000	-0.84365000	H	-4.42960400	-1.93755600	-2.11328900
H	5.64278100	-1.65156000	-0.24951700	H	-5.83046700	0.37598500	1.22519500
C	0.80687900	-1.11669600	1.69324700	H	-6.29158200	-0.82130200	-0.90387000
C	1.77787100	0.62507800	-1.08548900	O	-0.56267200	-2.73299800	0.49696600
C	0.48105000	0.53519200	-1.42048100	O	-1.34261200	1.60070400	1.74994200
C	0.58276100	0.14678200	2.04644900	O	-1.67384800	1.37438000	-1.44842800
H	2.14450600	1.61617500	-0.81797400	C	-2.63450000	2.40588200	-1.21313300
H	1.24235000	0.85765200	2.52445000	H	-2.53194100	3.19579500	-1.96070900
H	1.69746500	-1.72131500	1.80482700	H	-3.60540100	1.91625100	-1.29005100
H	0.00542100	-0.40641100	-1.68135800	H	-2.48957100	2.81932900	-0.21248100
C	-0.38516800	1.72982000	-1.33617900	C	4.66595000	0.92006800	-0.32958000
O	-0.01008600	2.86729200	-1.15197300	H	5.74424300	0.74378800	-0.36031000
C	-0.80331000	0.52881400	1.62178900	H	4.43508300	1.64198100	-1.11906500
C	-0.41602000	-1.64454400	0.99859700	C	4.29178800	1.47762500	0.97772100
N	-1.36835000	-0.61338000	1.02856200	C	3.99203800	1.96022800	2.04115000
C	-2.69137100	-0.68641400	0.51119500	H	3.73172700	2.39685400	2.98040200
C	-5.28271200	-0.78394100	-0.50577700				

Transition State (**TS**) **8j/7c-*exo***.

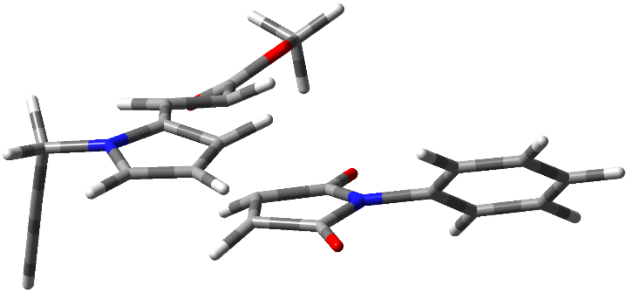

C	2.44368300	-0.66474300	-0.93401500	C	-4.08617800	0.14984700	0.91120900
C	1.34786400	-1.58091100	-0.97413600	C	-3.51402600	-1.54899500	-0.72018700
C	1.89874600	-2.89926300	-0.85920200	C	-4.86310400	-1.70792400	-1.02131900
C	3.24407800	-2.75054000	-0.66926700	C	-5.43205700	-0.00952900	0.59020900
N	3.58787000	-1.42002900	-0.73132100	H	-3.77548100	0.87144500	1.65650400
H	0.40467100	-1.35844700	-1.45692600	H	-2.76174400	-2.15067600	-1.21727100
H	1.35056700	-3.82816800	-0.89217900	H	-5.16018300	-2.43553300	-1.76974600
H	4.01043200	-3.49557800	-0.50085600	H	-6.17508800	0.59365200	1.10200300
C	0.49085200	-0.94842700	0.97269800	H	-6.87692300	-1.06060900	-0.61276200
C	2.33892500	0.71400800	-0.81786900	O	-1.14974000	-2.71526300	0.67304600
C	1.05500100	1.26098100	-0.75237500	O	-1.64888500	1.82865600	0.93602900
C	0.34649100	0.44655200	1.01220100	O	-0.12369600	3.23952600	-1.14800400
H	3.18406700	1.32651400	-0.51377700	C	-0.45576200	4.58598500	-0.80090100
H	0.93607600	1.11028000	1.63339500	H	0.38945800	5.24866100	-0.99648300
H	1.24354600	-1.51954700	1.49865600	H	-1.30837300	4.84744600	-1.42414600
H	0.26054600	0.83364100	-1.35938400	H	-0.72344700	4.63067800	0.25688000
C	0.91766700	2.72138600	-0.49380100	C	4.87383600	-0.86678900	-0.35223400
O	1.64532400	3.34988200	0.24329500	H	5.62920100	-1.64615900	-0.48421400
C	-1.11079500	0.74892700	0.85236100	H	5.12564400	-0.05135100	-1.03866500
C	-0.84782500	-1.54592700	0.73554000	C	4.88756600	-0.37501400	1.03395900
N	-1.74889800	-0.46824400	0.55736800	C	4.87425400	0.03896700	2.16616000
C	-3.12861400	-0.61971700	0.24829700	H	4.86295000	0.41003900	3.16741600
C	-5.82605900	-0.93733800	-0.37161400				

Adduct (**AD**) **8j/7c-*exo***.

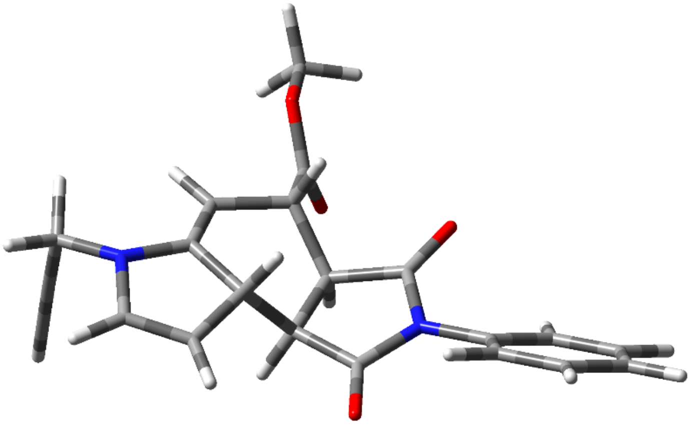

C	2.24089300	-0.91670700	-0.71013800	C	-4.25829500	0.83606400	0.63115300
C	0.88788900	-1.58071300	-0.55770000	C	-4.08592600	-1.25979000	-0.57662200
C	1.25706600	-3.03556800	-0.39339100	C	-5.46757800	-1.26558900	-0.74839900
C	2.57917300	-3.15383200	-0.55709200	C	-5.63742100	0.82281200	0.44467700
N	3.19571100	-1.92436200	-0.85982700	H	-3.77777300	1.65343600	1.15613700
H	0.28386600	-1.42290200	-1.46748600	H	-3.47672400	-2.06945000	-0.96026800
H	0.54861800	-3.83206200	-0.21798500	H	-5.93447500	-2.08677100	-1.28238000
H	3.19281200	-4.04611100	-0.52672500	H	-6.23649300	1.63649800	0.84023000
C	0.15649400	-0.92223200	0.63144400	H	-7.32203900	-0.23460800	-0.38102100
C	2.40632100	0.40664900	-0.60653100	O	-1.74950300	-2.42999000	0.81004400
C	1.15818300	1.25114200	-0.44119600	O	-1.67807500	2.02827800	-0.23684400
C	0.14683600	0.60827700	0.53102100	O	2.11147000	3.34781800	-0.93475900
H	3.37646900	0.88975300	-0.65228000	C	2.52685700	4.66834700	-0.57302900
H	0.31861400	1.05205500	1.51834300	H	3.26458200	4.62520500	0.23081600
H	0.61884800	-1.28049700	1.55433500	H	2.96065600	5.09683900	-1.47433100
H	0.67806100	1.39265500	-1.42002100	H	1.66706700	5.25272700	-0.24015500
C	1.53368900	2.64119700	0.04530600	C	4.59703500	-1.67494200	-0.59540200
O	1.37786000	3.06113800	1.16634300	H	5.16777300	-2.55085600	-0.91763700
C	-1.28014400	0.95567400	0.14666700	H	4.92472500	-0.83500400	-1.21707500
C	-1.30895500	-1.32080400	0.62449600	C	4.88188900	-1.38756800	0.82259700
N	-2.07361400	-0.19138700	0.30370900	C	5.08832100	-1.16056000	1.98913900
C	-3.48865000	-0.20723400	0.11644200	H	5.26981000	-0.95620800	3.02110400
C	-6.24585700	-0.22669200	-0.24161500				

Supramolecular complex (**SC**) **16a/7c-*endo***.

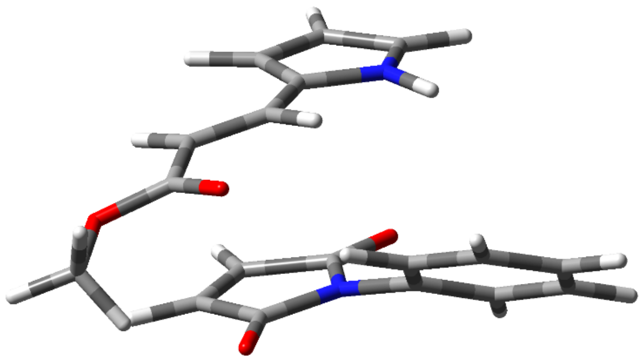

C	0.53306300	-1.79621500	-1.22916000	C	1.10947800	1.49998600	0.53739600
C	0.67707000	-3.11396600	-0.80114200	C	2.25618100	3.58995400	-0.91183500
C	2.05145200	-3.42941500	-0.83984400	C	0.33629000	2.20698400	-0.39083700
C	2.71899400	-2.29813600	-1.27648200	C	2.44849300	1.84104200	0.75045800
N	1.79669700	-1.33391400	-1.52696100	C	3.01576500	2.88043800	0.01645200
H	-0.13556600	-3.77029500	-0.52168500	C	0.91742700	3.25264300	-1.10449500
H	2.51237400	-4.36900400	-0.57425300	H	-0.70832300	1.95661100	-0.54638800
H	3.77364400	-2.11071000	-1.40743400	H	3.03738000	1.29041600	1.47289900
C	0.16722600	-1.69166300	2.17229200	H	4.05675300	3.13923700	0.18209800
H	0.42313600	-2.69132000	2.49641600	H	0.31165500	3.80123100	-1.81858800
C	-0.60817100	-0.92955000	-1.34777000	H	2.70176500	4.40331900	-1.47534200
C	-1.84129900	-1.16659800	-0.86446700	H	-2.11457300	-2.08744600	-0.35992600
C	-1.00096400	-1.05136800	2.20525600	C	-2.83785700	-0.08101100	-0.90959800
H	-1.96628300	-1.38172300	2.56542300	O	-2.69138600	0.98411600	-1.47401200
H	-0.44206900	0.03960600	-1.81799200	O	-3.93065300	-0.38875900	-0.19214600
C	-0.81767900	0.32166100	1.63335200	C	-4.87475500	0.67489100	-0.04826600
C	1.20071800	-0.78280900	1.58174700	H	-5.68623400	0.26872400	0.55292000
N	0.53359300	0.41580900	1.25473100	H	-4.40049800	1.51941600	0.45650000
O	-1.65003600	1.19072600	1.53493600	H	-5.23961700	0.99358200	-1.02672500
O	2.37793600	-1.00654800	1.43706200	H	2.00895000	-0.37239500	-1.75736900

Transition State (**TS**) **16a/7c-*endo***.

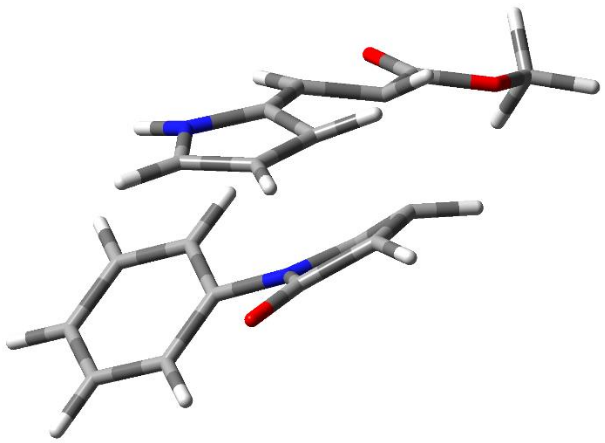

C	0.23235500	1.88500400	-1.17366200	C	-1.75346100	-1.14232700	0.39740100
C	0.41410600	2.95610900	-0.23809200	C	-3.74592200	-2.74194700	-0.72076300
C	-0.76750100	3.74799700	-0.27262800	C	-1.43419700	-2.03700100	-0.63055700
C	-1.65028400	3.11261200	-1.11001800	C	-3.06400900	-1.04975500	0.87180100
N	-1.05440900	2.01967200	-1.67178900	C	-4.05415800	-1.84814200	0.30229200
H	1.38700400	3.29740200	0.08704200	C	-2.43236200	-2.83572000	-1.18005200
H	-0.96816700	4.64528300	0.29160900	H	-0.40782600	-2.12142200	-0.97357200
H	-2.67470500	3.36075600	-1.34779400	H	-3.30000700	-0.35235900	1.66509300
C	0.45839500	1.56033200	1.58072400	H	-5.07241300	-1.77242000	0.67052300
H	0.61002100	2.41547400	2.22342000	H	-2.17830400	-3.53522400	-1.97010200
C	1.06647200	0.80359800	-1.34675000	H	-4.52147500	-3.36558400	-1.15361300
C	2.15634400	0.64946900	-0.45951800	H	2.75338700	1.51930100	-0.19555300
C	1.39292300	0.52364800	1.34114800	C	2.95535100	-0.60668400	-0.63278500
H	2.32401000	0.42616800	1.88882500	O	2.71406500	-1.46959200	-1.44289400
H	0.77358000	-0.04366900	-1.96136900	O	3.96090500	-0.67240000	0.25108700
C	0.59782700	-0.72596500	1.11365300	C	4.67616500	-1.91250300	0.25557000
C	-0.89182800	1.03139300	1.35629700	H	5.43255600	-1.81298400	1.03167500
N	-0.72924700	-0.31919500	0.93358400	H	3.98711000	-2.72866000	0.48206200
O	1.01183800	-1.86114100	1.05056900	H	5.13933400	-2.08473500	-0.71784900
O	-1.96166400	1.59399700	1.46801700	H	-1.53695400	1.30171300	-2.19156200

Adduct (**AD**) **16a/7c-*endo***.

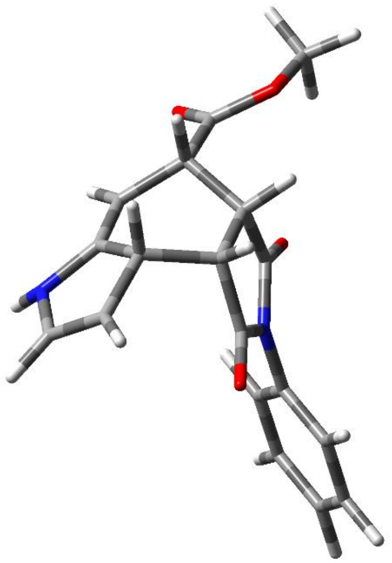

C	-1.41537800	1.99986300	0.86733600	C	2.32669400	-0.69653900	-0.10527700
C	-1.12075500	2.46686000	-0.54788400	C	4.76234100	-1.69165500	0.78877600
C	-0.22176500	3.65868500	-0.31682500	C	2.40192800	-1.31087400	1.14481200
C	-0.13329000	3.87153700	1.00124800	C	3.45725300	-0.57442600	-0.91239100
N	-0.90294200	2.95762600	1.73854100	C	4.67533800	-1.07144400	-0.45611100
H	-2.06463600	2.77733900	-1.02278700	C	3.62418400	-1.81121400	1.58453300
H	0.25068500	4.24352200	-1.09147200	H	1.50975300	-1.40534100	1.75281500
H	0.40667000	4.64950500	1.52458600	H	3.38308700	-0.08341300	-1.87520400
C	-0.57698000	1.29396100	-1.41663200	H	5.55795900	-0.97356600	-1.07973100
H	-0.68817200	1.56247900	-2.47043500	H	3.68446900	-2.29484600	2.55405700
C	-2.04533300	0.84462700	1.11303100	H	5.71354700	-2.08055700	1.13766800
C	-2.43284900	0.03942400	-0.09573400	H	-3.25879500	0.52101600	-0.64496100
C	-1.24240400	-0.05461100	-1.08284400	C	-2.94759300	-1.34088200	0.28265900
H	-1.56340100	-0.57922200	-1.98742600	O	-3.18451600	-1.70831600	1.40594700
H	-2.26941200	0.46240800	2.10223600	O	-3.15914300	-2.09243400	-0.80791300
C	-0.13169800	-0.88632500	-0.45169600	C	-3.53465800	-3.44644600	-0.54311900
C	0.90799300	1.06095300	-1.17258100	H	-3.65720500	-3.91499700	-1.51781100
N	1.07220800	-0.18695300	-0.55809400	H	-2.74358100	-3.93771300	0.02739200
O	-0.26216500	-1.97686200	0.05071600	H	-4.46735700	-3.47855700	0.02326500
O	1.80530700	1.81448600	-1.45901400	H	-0.69251700	2.73327800	2.69897400

Supramolecular complex (**SC**) **16a/7c-*exo***.

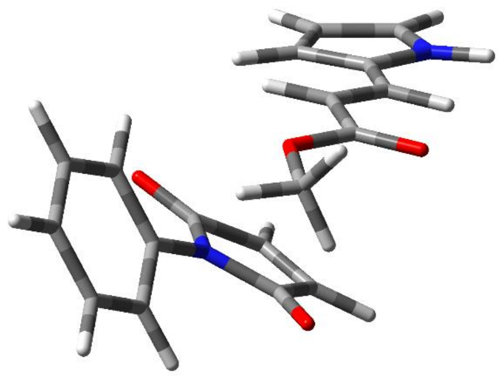

C	3.38450400	0.31268000	-0.52381500	N	-0.99514800	-0.88695700	0.83899300
C	3.36624700	-1.01152900	-0.94437200	C	-2.23137700	-0.89481600	0.13438200
C	4.68243200	-1.51630800	-0.83083400	C	-4.65790500	-0.86125400	-1.23116600
C	5.47556600	-0.49434300	-0.34355300	C	-3.41627000	-0.66263300	0.83365500
N	4.68238600	0.59731200	-0.15702300	C	-2.25246900	-1.11890600	-1.24268200
H	2.49186600	-1.55502000	-1.27806900	C	-3.46876300	-1.10575000	-1.91792700
H	5.01753700	-2.51351400	-1.07474100	C	-4.62638700	-0.63965400	0.14388900
H	6.53130900	-0.46458500	-0.12046800	H	-3.38717000	-0.48545300	1.90223200
C	1.14632000	-1.38064800	1.61009400	H	-1.32657200	-1.29572200	-1.77609700
C	2.33681300	1.29715200	-0.39353000	H	-3.48375500	-1.27927900	-2.98900700
C	1.07007200	1.13269900	-0.80531200	H	-5.54614200	-0.45243600	0.68862300
C	0.71252600	-0.34710000	2.32742800	H	-5.60265200	-0.84623100	-1.76489000
H	2.57770800	2.23529900	0.10814000	O	0.15606600	-2.59448500	-0.24946800
H	1.20290200	0.20419800	3.11819400	O	-1.35191500	0.93352300	2.24726000
H	2.09117900	-1.90619400	1.64855100	O	-1.16544900	1.72116900	-0.85990000
H	0.73737400	0.23807700	-1.32518000	C	-2.27193800	2.54578600	-0.48864300
C	0.05039200	2.15625500	-0.49706000	H	-2.19756100	3.51991500	-0.97720000
O	0.25559400	3.22569100	0.03564500	H	-3.15710600	2.00462400	-0.82416300
C	-0.66021300	0.02750900	1.85226900	H	-2.28743300	2.67262900	0.59607000
C	0.08970600	-1.74456500	0.60593200	H	4.99793900	1.49658700	0.17340000

Transition State (**TS**) **16a/7c-*exo***.

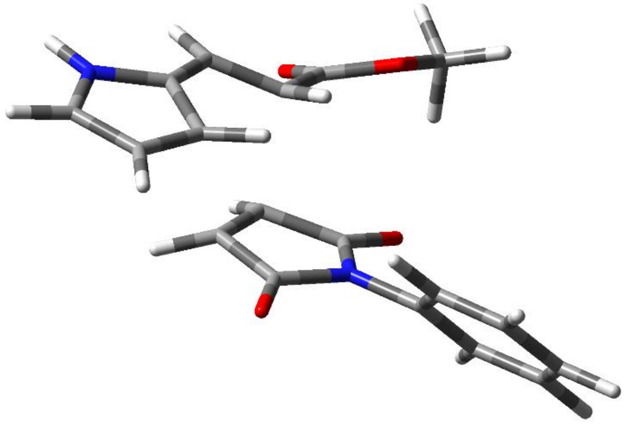

C	3.00982800	-0.97974600	-0.53026600	N	-1.26628600	-0.39841700	0.56548600
C	1.84585500	-1.79956600	-0.65972000	C	-2.61920600	-0.43626300	0.12878600
C	2.26992700	-3.15981600	-0.50361100	C	-5.26376200	-0.53105200	-0.74184000
C	3.60618100	-3.13043500	-0.21144300	C	-3.56290600	0.42730000	0.68763600
N	4.05511300	-1.83332300	-0.22486900	C	-2.99249200	-1.34869400	-0.86021500
H	0.96883100	-1.49867800	-1.21838400	C	-4.31599500	-1.39587500	-1.28685200
H	1.64539300	-4.03703200	-0.57351400	C	-4.88144000	0.37880000	0.24156900
H	4.28652600	-3.94200200	0.00447200	H	-3.26096600	1.13475900	1.44988600
C	0.87398200	-1.06235500	1.19545200	H	-2.25317500	-2.02403300	-1.27540800
C	3.01495300	0.40299200	-0.44159700	H	-4.60465300	-2.11040600	-2.05099200
C	1.78131800	1.06040600	-0.48191200	H	-5.61289800	1.05492900	0.67247200
C	0.85036000	0.34183400	1.20550500	H	-6.29400600	-0.56752400	-1.08081800
H	3.89147300	0.94501600	-0.09542800	O	-0.87946300	-2.68621500	0.76275800
H	1.42749800	0.96269500	1.88049300	O	-1.00232400	1.88744700	0.93193000
H	1.51734200	-1.68855700	1.79807400	O	0.83525900	3.13522800	-0.99898400
H	1.00870400	0.69874700	-1.15633000	C	0.60720200	4.51396400	-0.69674500
C	1.76114100	2.53228400	-0.25184500	H	1.52643100	5.08853400	-0.82562600
O	2.48044900	3.10141200	0.54009900	H	-0.15974100	4.84368900	-1.39440600
C	-0.55548000	0.76414300	0.90839200	H	0.25606900	4.60469200	0.33330100
C	-0.48352400	-1.54620200	0.84033400	H	4.98834800	-1.53592200	0.01169900

Adduct (**AD**) **16a/7c-*exo***.

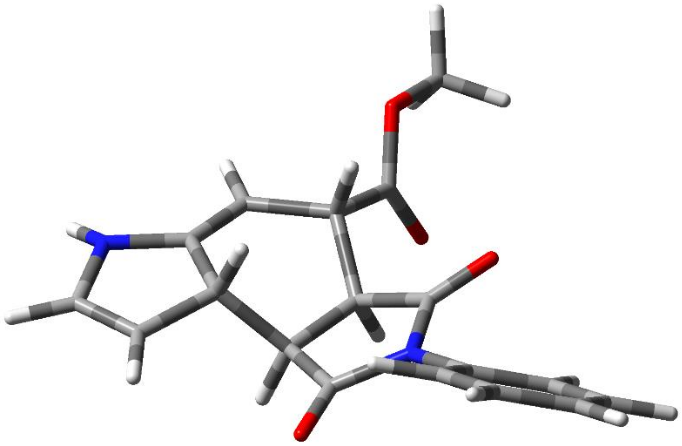

C	-2.61401900	1.64277400	-0.35643600	N	1.54401100	0.04554700	0.31539100
C	-1.14756400	2.02729900	-0.29761300	C	2.91889800	-0.21222000	0.03077700
C	-1.20508800	3.52089600	-0.08426300	C	5.59564500	-0.72449100	-0.51796400
C	-2.48673300	3.90331100	-0.14266100	C	3.50462300	-1.38970300	0.49524000
N	-3.34939900	2.82518000	-0.40172000	C	3.65974900	0.71105900	-0.70650300
H	-0.66200500	1.78155000	-1.25778200	C	5.00097100	0.45034700	-0.97396100
H	-0.34147900	4.15588300	0.04818400	C	4.84395100	-1.64243900	0.21348200
H	-2.90096400	4.89954600	-0.05892900	H	2.91179600	-2.10294900	1.05602300
C	-0.47005800	1.19292100	0.80971900	H	3.19398600	1.62688300	-1.05039700
C	-3.01441000	0.36724900	-0.31116800	H	5.58045900	1.17019800	-1.54271600
C	-1.93214800	-0.69367700	-0.27099900	H	5.29958000	-2.56041600	0.56999900
C	-0.77476300	-0.30481900	0.67406600	H	6.64061900	-0.92426400	-0.73191800
H	-4.05802300	0.07241600	-0.34190800	O	1.70308000	2.29454400	0.85158700
H	-0.99651500	-0.74014400	1.65489800	O	0.68760200	-2.04643400	-0.19839700
H	-0.77645500	1.59788000	1.77734900	O	-3.19741800	-2.59791700	-0.84193800
H	-1.53428100	-0.85132400	-1.28378100	C	-3.81929300	-3.84722300	-0.52636300
C	-2.52720500	-2.02225100	0.16399900	H	-4.55198700	-3.71389300	0.27221000
O	-2.45681000	-2.49281600	1.27378000	H	-4.30253700	-4.17476800	-1.44467300
C	0.53236800	-0.92101900	0.20837900	H	-3.06575700	-4.56876600	-0.20528200
C	1.04080200	1.29696400	0.69388100	H	-4.32471200	2.84819900	-0.14690500

Supramolecular complex (**SC**) **18a/7c-*endo***.

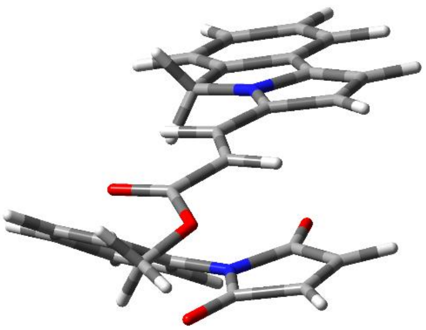

H	-4.96638600	-2.43346200	0.51162300
C	-4.76343000	-1.46571800	0.06423100
C	-4.23047100	1.05899100	-1.09015100
C	-3.48205300	-1.13307400	-0.36359000
C	-5.77982600	-0.52276600	-0.09143700
C	-5.51899500	0.72549000	-0.66105400
C	-3.22045600	0.12315800	-0.93894000
H	-6.78651800	-0.76149000	0.23639700
H	-6.32418000	1.44479600	-0.77012900
H	-4.02773300	2.03234100	-1.52794500
C	-2.22489400	-1.86856300	-0.33515600
C	-1.75662200	0.23249000	-1.30966600
H	-1.25255900	1.03769600	-0.76538600
H	-1.60494500	0.37576700	-2.38478500
C	-1.63323100	-3.05970000	0.07240200
H	-2.13025400	-3.89519700	0.54215700
N	-1.26410800	-1.07624900	-0.88668900
C	-0.26420700	-2.94583700	-0.23280000
H	0.50013100	-3.69180800	-0.06050100
C	-0.03982300	-1.69423500	-0.82779700
C	1.15936900	-1.03183400	-1.25366700
H	1.04481100	-0.05082600	-1.71602300
C	2.41835000	-1.47339700	-1.06148700
H	2.64536400	-2.42691300	-0.59626800
C	2.39777200	-1.23315700	2.12220600
C	1.14143200	-1.61198800	2.35783600
C	3.54616800	-0.58640300	-1.39040800
O	4.71014700	-1.08921200	-0.94645900
O	3.45806300	0.48942600	-1.94868400
C	5.83271900	-0.21439200	-1.07566400
H	5.65427700	0.69738200	-0.50137100
H	5.99689000	0.04120000	-2.12440600
H	6.68212200	-0.76478700	-0.67496100
H	0.75722200	-2.54368500	2.75033400
H	3.32542800	-1.76975800	2.27092900
C	2.38941700	0.16115600	1.57216000
C	0.21908000	-0.49601400	1.97282000
N	1.04123000	0.55143900	1.51061000
C	0.55865200	1.75283500	0.92546700
C	-0.49050800	4.02374900	-0.30068700
C	1.19012300	2.28069600	-0.20534600
C	-0.57676600	2.36740300	1.46013300
C	-1.10261500	3.49563600	0.83516200
C	0.66007300	3.41976600	-0.80735800
H	2.07870200	1.80909600	-0.61343800
H	-1.05744800	1.94688800	2.33466100
H	-1.99391200	3.96169300	1.24300200
H	1.15091300	3.82724300	-1.68537700
H	-0.90179400	4.90500400	-0.78257300
O	3.34151300	0.83543600	1.26057700
O	-0.98541400	-0.47230900	2.04998600

Transition State (**TS**) **18a/7c-*endo***.

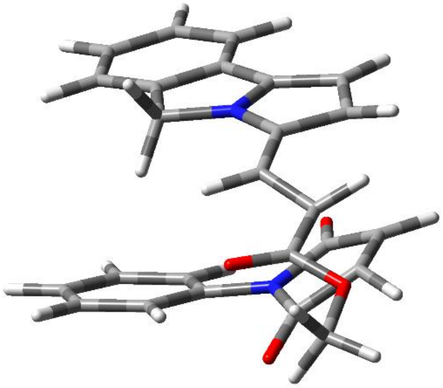

H	-4.41910200	-2.64640900	0.79389400	C	3.85766600	-0.32241500	-1.07854100
C	-4.36427800	-1.74563500	0.19131800	O	5.00039800	-0.73504100	-0.50983700
C	-4.20100200	0.61942200	-1.35417800	O	3.78241200	0.63027800	-1.81695100
C	-3.15714700	-1.33203000	-0.36420400	C	6.13312200	0.10401100	-0.75758400
C	-5.49539700	-0.96499100	-0.03808500	H	5.93104400	1.11074600	-0.38636200
C	-5.41480200	0.20403400	-0.80129400	H	6.34453300	0.14658000	-1.82778700
C	-3.07465000	-0.15811400	-1.13170200	H	6.96078600	-0.35194300	-0.21772700
H	-6.44900700	-1.26333000	0.38496600	H	1.39304200	-2.49996600	2.30666000
H	-6.30765400	0.79981900	-0.96192800	H	3.54581500	-1.16649000	1.51227300
H	-4.14079000	1.53549900	-1.93448800	C	2.18966300	0.55268400	1.20364500
C	-1.81628400	-1.89698600	-0.28596500	C	0.31053300	-0.61899600	1.88585800
C	-1.65196100	0.06806800	-1.59738800	N	0.80505500	0.63886800	1.38935700
H	-1.24063300	1.01473400	-1.23149200	C	-0.00174800	1.72331700	0.95770800
H	-1.56033800	0.03520000	-2.68902000	C	-1.65774300	3.72514800	-0.09484500
C	-1.09538500	-2.94117000	0.27309800	C	0.48708700	2.59996400	-0.02468000
H	-1.49115000	-3.73866500	0.88215200	C	-1.31026100	1.87434200	1.43407400
N	-0.98389800	-1.07814200	-0.99181600	C	-2.12895200	2.86587300	0.89385200
C	0.25761600	-2.69181400	-0.03717600	C	-0.34107900	3.59224800	-0.53859500
H	1.06828900	-3.40119700	0.04970500	H	1.50352600	2.50056100	-0.38604800
C	0.32289900	-1.51386000	-0.87032200	H	-1.68897200	1.20415100	2.19241500
C	1.44944400	-0.82004000	-1.23931100	H	-3.14803300	2.95812700	1.25697100
H	1.37244500	0.13736400	-1.74969000	H	0.04882400	4.26021200	-1.30035400
C	2.69691900	-1.17346000	-0.65080000	H	-2.30085000	4.49661500	-0.50625700
H	2.95040900	-2.22919300	-0.57086900	O	2.95316800	1.47353300	1.01690500
C	2.53287600	-0.90992600	1.21695300	O	-0.81944900	-0.79861400	2.29903600
C	1.39744500	-1.57113000	1.75599500				

Adduct (**AD**) **18a/7c-*endo***.

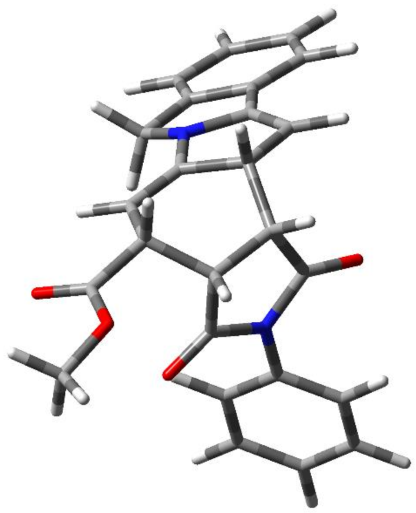

H	-4.96051500	-0.01297200	1.93324300	C	3.16348200	-2.33250900	-0.64724500
C	-4.92401800	-0.21093300	0.86669100	O	4.31025300	-2.19867300	0.03620100
C	-4.81368600	-0.72525800	-1.90856100	O	3.11162400	-2.59393400	-1.82277300
C	-3.77573100	-0.73511100	0.28131800	C	5.49457200	-2.21922400	-0.76432300
C	-6.02287900	0.05221000	0.05087200	H	5.46118800	-1.39802800	-1.48351000
C	-5.96869900	-0.20321100	-1.32230800	H	5.57704100	-3.16831300	-1.29749200
C	-3.71759300	-0.98825800	-1.09857300	H	6.32291300	-2.09205700	-0.06981200
H	-6.93020300	0.45936200	0.48507100	H	1.06559100	-0.67058100	3.08429700
H	-6.83548100	0.00712400	-1.94070900	H	2.99019600	-0.96346100	1.74646500
H	-4.77768400	-0.91839300	-2.97682400	C	2.22287100	0.31553600	0.23752300
C	-2.47788000	-1.08834200	0.85684700	C	0.53391800	0.86939200	1.73157400
C	-2.35448100	-1.53178800	-1.48652200	N	1.31493700	1.29321800	0.64905200
H	-1.79361200	-0.82245000	-2.11461700	C	1.21147400	2.58182000	0.04334900
H	-2.41576100	-2.48864000	-2.01775100	C	1.00705100	5.08229900	-1.15098100
C	-1.75791400	-0.97016800	1.98333400	C	1.20276800	2.68121000	-1.34805300
H	-2.07609900	-0.54001500	2.92072400	C	1.11879000	3.72007800	0.84332400
N	-1.73795700	-1.68007500	-0.18360000	C	1.01217300	4.96955000	0.23796300
C	-0.37059200	-1.51152600	1.69341000	C	1.10461200	3.93716200	-1.93982000
H	-0.18980900	-2.44875400	2.24295400	H	1.28619700	1.78490100	-1.95180500
C	-0.42466300	-1.82792000	0.20232500	H	1.11498200	3.62290500	1.92227500
C	0.66831600	-2.15311700	-0.50279600	H	0.93477300	5.85704300	0.85754800
H	0.67093300	-2.38170600	-1.56216800	H	1.10291700	4.01818200	-3.02193200
C	1.95886500	-2.17420200	0.26765600	H	0.92725600	6.05897700	-1.61734100
H	1.99871100	-3.03551700	0.95516200	O	3.00069200	0.42912300	-0.67941900
C	2.07960900	-0.90240500	1.14316300	O	-0.24964000	1.56249900	2.33230900
C	0.84699500	-0.59390700	2.01608900				

Supramolecular complex (**SC**) **18a/7c-*exo***.

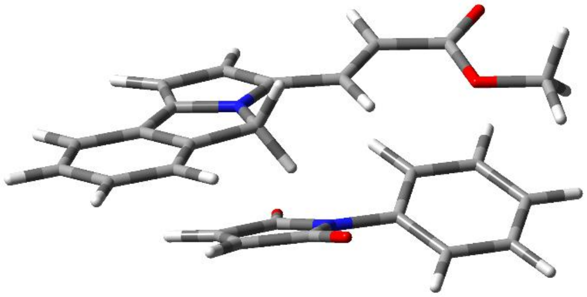

H	-5.62284400	-0.72497900	-1.47351100	C	-0.99470900	-2.64928200	-0.37231700
C	-5.36003000	-0.17257800	-0.57684900	H	-1.62597600	-3.24586000	-1.01624900
C	-4.67576600	1.26686400	1.75700100	C	0.48679100	-2.56124600	-0.59254000
C	-4.06653200	0.30745000	-0.38972400	C	-0.07181200	-1.24159800	1.19451200
C	-6.31043500	0.07409400	0.41442800	O	0.00594100	-0.44410300	2.10297000
C	-5.97499400	0.78423100	1.56918900	O	1.13460700	-3.11438700	-1.44449700
C	-3.72951900	1.02571900	0.77376100	N	0.98884700	-1.70754000	0.41057600
H	-7.32425900	-0.29157600	0.28664000	C	2.35653500	-1.34882900	0.57919500
H	-6.72923100	0.96350000	2.32828500	C	5.03083900	-0.65277700	0.91778200
H	-4.41778900	1.81851100	2.65642200	C	3.17204900	-1.16933900	-0.53961000
C	-2.86513300	0.20860100	-1.20771900	C	2.87013900	-1.18098500	1.86605800
C	-2.27167300	1.44017000	0.72824400	C	4.20705600	-0.82704100	2.02784100
H	-1.68745600	1.05196800	1.57017200	C	4.50871500	-0.82304100	-0.36335100
H	-2.15403700	2.52825200	0.68262400	H	2.76299600	-1.29929300	-1.53415600
C	-2.35337700	-0.29007100	-2.39539700	H	2.22757500	-1.31615700	2.72757500
H	-2.89530900	-0.84131700	-3.14980100	H	4.60454300	-0.69570200	3.02936900
N	-1.84911500	0.82618500	-0.52864600	H	5.13625800	-0.66992900	-1.23515700
C	-0.98121300	0.05529900	-2.41543900	H	6.07411800	-0.38236900	1.04875200
H	-0.26491700	-0.19974800	-3.18476600	C	2.92197500	2.04052800	-0.95938200
C	-0.68226700	0.76165400	-1.24801100	O	2.90901900	2.18542400	0.37788600
C	0.55692000	1.31880700	-0.74951300	O	3.88932300	2.29830000	-1.64448800
H	0.61004100	1.53907200	0.31705600	C	4.10771200	2.71067100	0.94549500
C	1.64460300	1.54362400	-1.50472100	H	4.25087700	3.74725600	0.62941200
H	1.65271300	1.37037600	-2.57640100	H	4.96708000	2.11615800	0.63106400
C	-1.31599800	-1.89568300	0.67616000	H	3.97274300	2.65054700	2.02439600
H	-2.28563400	-1.70257300	1.11922400				

Transition State (**TS**) **18a/7c-*exo***.

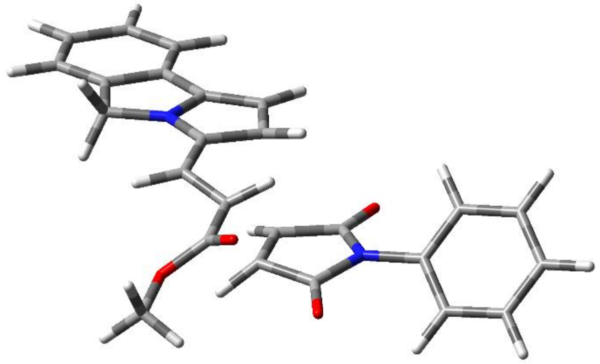

H	4.69310900	-3.33933900	0.04067500	C	-0.32416100	-0.31814500	-1.07850200
C	5.15944400	-2.36381400	-0.05209200	H	0.56441200	-0.54738700	-1.64991000
C	6.36477100	0.18236500	-0.29620700	C	-1.31199800	-1.38852500	-0.80681300
C	4.41582600	-1.20049900	0.12160100	C	-2.44070500	0.62962700	-0.80176900
C	6.51643000	-2.24287900	-0.34837100	O	-3.35162700	1.42336000	-0.81654600
C	7.11328500	-0.98534500	-0.46880600	O	-1.13947300	-2.58606000	-0.78938100
C	5.01533000	0.06512500	-0.00146900	N	-2.54474500	-0.74586900	-0.53295700
H	7.11678900	-3.13591400	-0.48767400	C	-3.74058700	-1.42520500	-0.17109000
H	8.17107400	-0.91405400	-0.69997200	C	-6.06936900	-2.77283700	0.55315700
H	6.83499100	1.15663800	-0.39276300	C	-3.67974400	-2.47019100	0.75341600
C	3.00483500	-0.99409600	0.43217900	C	-4.96083900	-1.05209700	-0.73742600
C	3.98748700	1.15821400	0.22582700	C	-6.12131800	-1.72593900	-0.36446600
H	3.84960600	1.78922400	-0.66025500	C	-4.84491400	-3.14365000	1.10661700
H	4.23851300	1.79886700	1.07859100	H	-2.72444900	-2.75933300	1.17607600
C	1.82298000	-1.65719600	0.66525800	H	-4.99837800	-0.23447400	-1.44627600
H	1.66133900	-2.72388300	0.68519600	H	-7.07013400	-1.42950200	-0.80002200
N	2.80028100	0.36521100	0.49838400	H	-4.79337800	-3.95893200	1.82111100
C	0.82768900	-0.64518200	0.83668900	H	-6.97680600	-3.29670100	0.83571800
H	-0.11678900	-0.80447300	1.34110800	C	-1.27805200	3.18362000	0.63888800
C	1.47909700	0.63410100	0.77456800	O	-0.76538200	3.99041000	-0.31045100
C	0.84994700	1.86785100	0.71981400	O	-2.21497500	3.48282000	1.33671700
H	1.40308500	2.75971500	0.43769000	C	-1.44766200	5.23830000	-0.46559000
C	-0.55128300	1.88450800	0.71281200	H	-1.39876700	5.81398000	0.46095700
H	-1.08176400	1.17119000	1.33958100	H	-2.49358500	5.05862600	-0.72199300
C	-0.98926000	0.91708300	-1.03855000	H	-0.93312900	5.75826300	-1.27161000
H	-0.74620400	1.76670100	-1.66497200				

Adduct (**AD**) **18a/7c-*exo***.

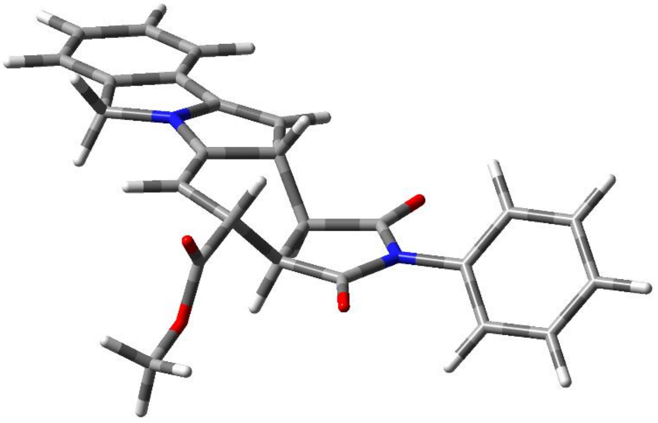

H	-4.64791600	-3.37349100	0.30597900	C	0.30416200	-0.37776600	0.61444700
C	-5.16574200	-2.42073400	0.25811800	H	-0.15742300	-0.52400800	1.59540800
C	-6.50195000	0.06456800	0.12692600	C	1.39910800	-1.41847600	0.45645300
C	-4.46706500	-1.24549400	-0.00073600	C	2.48442400	0.63643800	0.30668000
C	-6.54394300	-2.34385700	0.44986500	O	3.38234800	1.42772200	0.14810200
C	-7.20527900	-1.11407200	0.38447700	O	1.24883600	-2.61672700	0.45043800
C	-5.12904000	-0.00887100	-0.06388000	N	2.62380800	-0.75878500	0.28656300
H	-7.11065100	-3.24726100	0.65027900	C	3.86998000	-1.42868600	0.09236300
H	-8.27943200	-1.07519700	0.53468300	C	6.29854200	-2.72735900	-0.28229200
H	-7.02262500	1.01656500	0.07815900	C	3.94222000	-2.51554300	-0.77833600
C	-3.04113300	-1.00473800	-0.22346300	C	5.00112200	-0.98473400	0.77660700
C	-4.14422200	1.11286500	-0.34009800	C	6.21537500	-1.63659600	0.58127700
H	-4.05565800	1.80085400	0.51473200	C	5.16076000	-3.16442400	-0.95789000
H	-4.41055300	1.69872400	-1.22775000	H	3.05208700	-2.85405100	-1.29503600
C	-1.84517000	-1.61364200	-0.16801100	H	4.93032800	-0.13009000	1.43915500
H	-1.63334200	-2.65401600	0.02937900	H	7.09794400	-1.28918300	1.10831300
N	-2.92268300	0.36133000	-0.54078000	H	5.21830400	-4.01328500	-1.63133600
C	-0.79200900	-0.56297300	-0.45876200	H	7.24682100	-3.23423100	-0.42885200
H	-0.29376600	-0.75436600	-1.42415900	C	1.07564200	3.31333100	-0.49315000
C	-1.59646200	0.72734000	-0.55988200	O	1.13760000	3.74912500	0.77416400
C	-1.00391400	1.92939600	-0.60461100	O	1.38364200	3.98785600	-1.44442000
H	-1.54800500	2.86476900	-0.67548600	C	1.68487200	5.05982600	0.93802900
C	0.51113600	1.90853700	-0.59810400	H	1.07727700	5.79345400	0.40443300
H	0.88064200	1.53425900	-1.56392300	H	2.70489300	5.08535800	0.54974900
C	1.02261400	0.97820400	0.52879100	H	1.67322900	5.25254500	2.00912900
H	0.94637700	1.52577600	1.47149200				

Appendix 6

Calculated [M06-2X/6-31+G(d,p)] NCIs, including distances, angles and contact type from the ZPE-corrected geometries of the *endo* TSs of the Diels–Alder reactions of dienes **8b**, **8c**, **8g**, **8j** and**18a** and dienophiles **7b**,**c**.

Transition state **8b/7c-*endo***

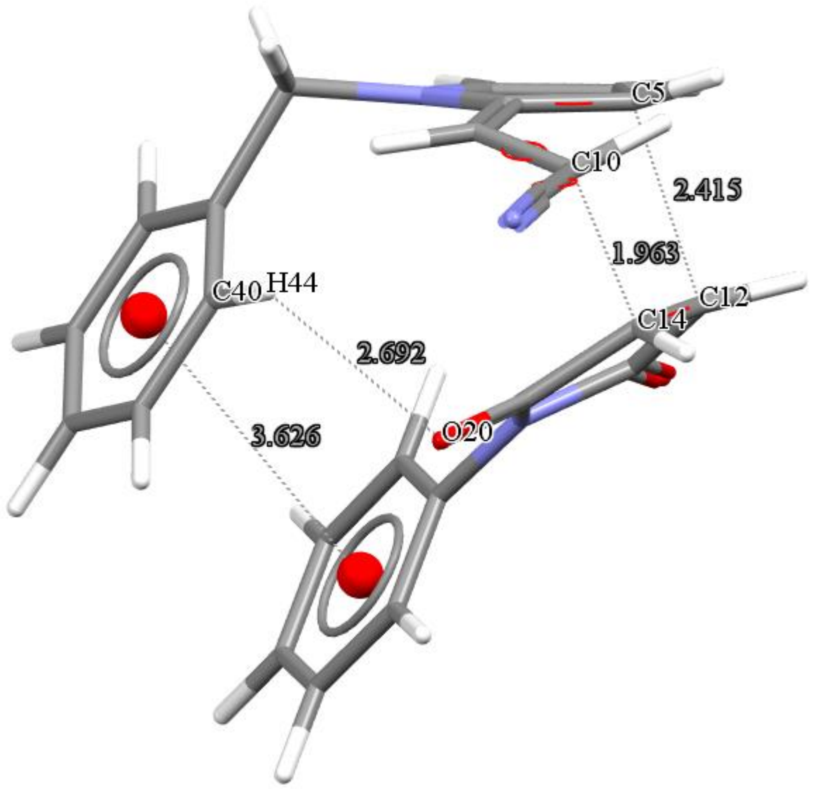

Atom	Distance (Å)	Angle (°)	Contact Type
C10 ··· C14	1.963	*a*	
C5 ··· C12	2.415	*a*	
C40-H44 ··· O20	2.962	131.89	D-H ··· A
π···π	Centroid-centroid 3.626	Plane-plane torsion angle 4.24	Offset π-stacked

[a] No determined.

Transition state **8c/7c-*endo***.

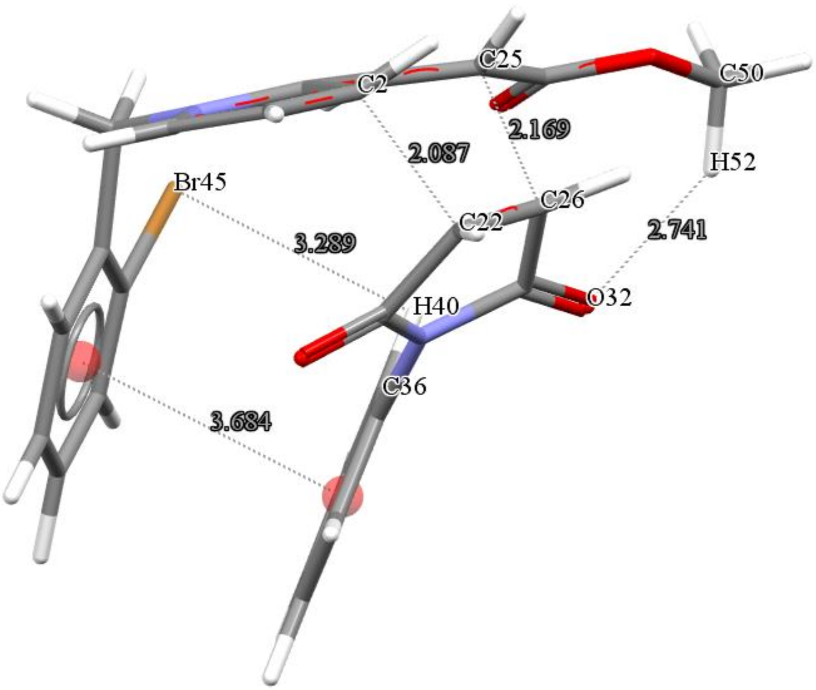

Atom	Distance (Å)	Angle (°)	Contact Type
C2 ··· C22	2.087	*a*	
C25 ··· C26	2.169	*a*	
C50-H52 ··· O32	2.741	119.78	D-H ··· A
C36-H40 ··· Br45	3.289	98.02	D-H ··· A
π···π	Centroid-Centroid 3.684	Plane-plane torsion angle 16.02	Offset π-stacked

[a] No determined

Transition state **8g/7c-*endo***.

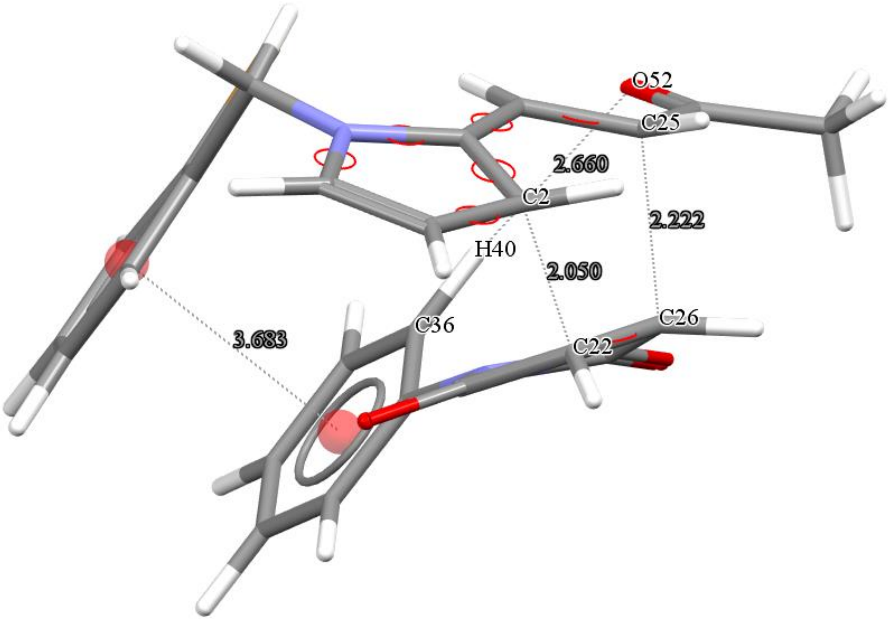

Atom	Distance (Å)	Angle (°)	Contact Type
C2··· C22	2.050	*a*	
C25··· C26	2.222	*a*	
C36-H40··· O52	2.660	163.14	D-H ··· A
π···π	Centroid-Centroid 3.683	Plane-plane torsion angle 15.90	Offset π-stacked

[a] No determined

Transition state **16a/7c-*endo***.

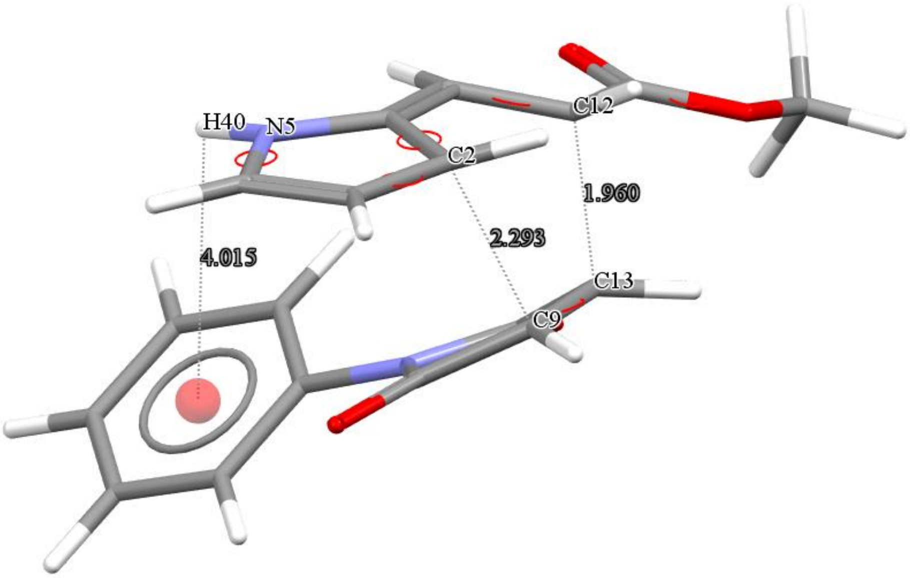

Atom	Distance (Å)	Angle (°)
C2··· C9	2.293	*a*
C12··· C13	1.960	*a*
N5-H40 ···π	4.015	117.28

[a] No determined

Transition state **8c/7b-*endo***.

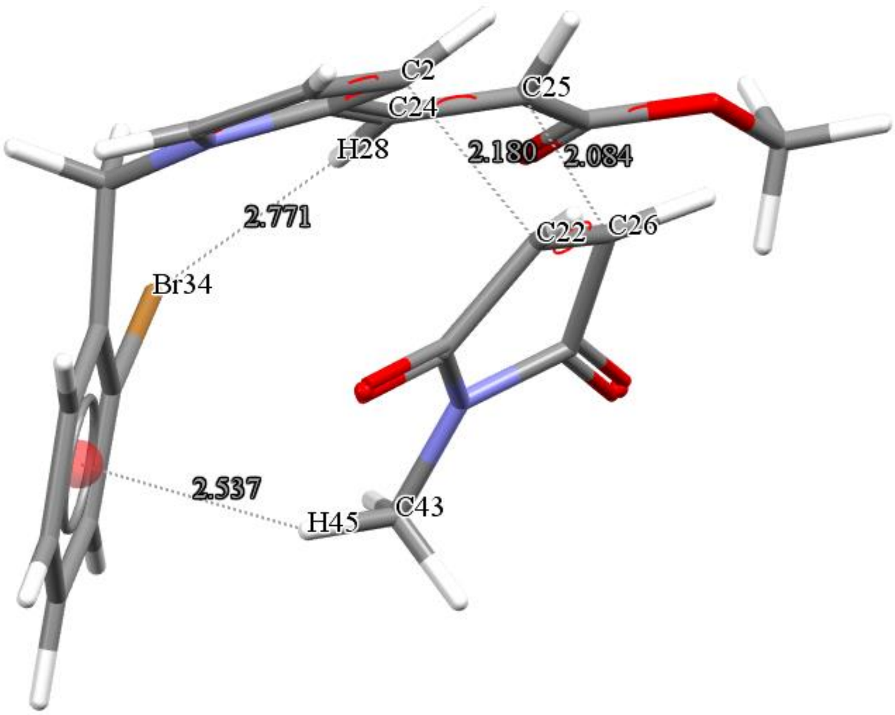

Atom	Distance (Å)	Angle (°)	Contact Type
C2··· C22	2.180	*a*	
C25··· C26	2.084	*a*	
C43-H45···π	2.537	126.61	D-H ··· A
C24-H28···Br34	2.771	160.54	D-H ··· A

[a] No determined

Transition state **8j/7c-*endo***.

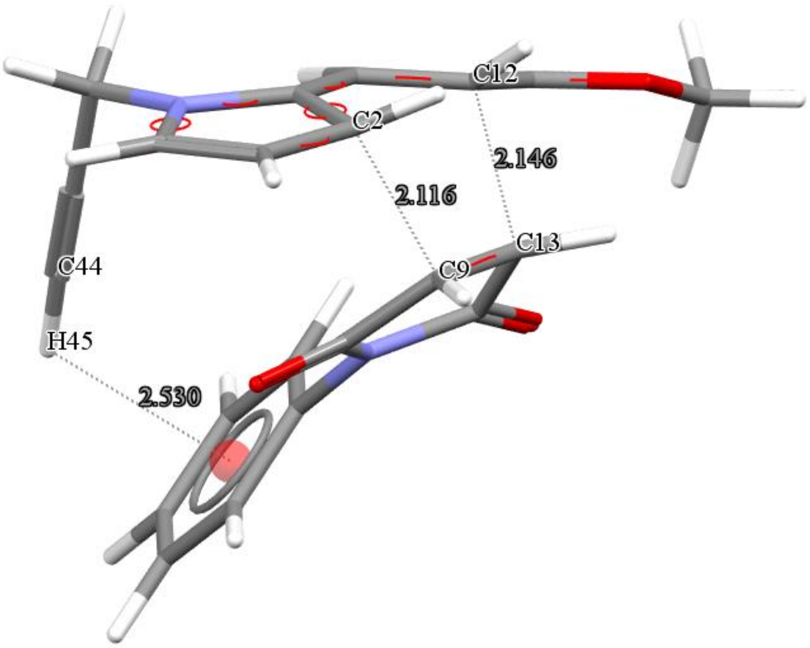

Atom	Distance (Å)	Angle (°)	Contact Type
C2··· C9	2.116	*a*	
C12··· C13	2.146	*a*	
C44-H45···π	2.530	115.19	D-H ··· A

[a] No determined

Transition state **18a/7c-*endo***.

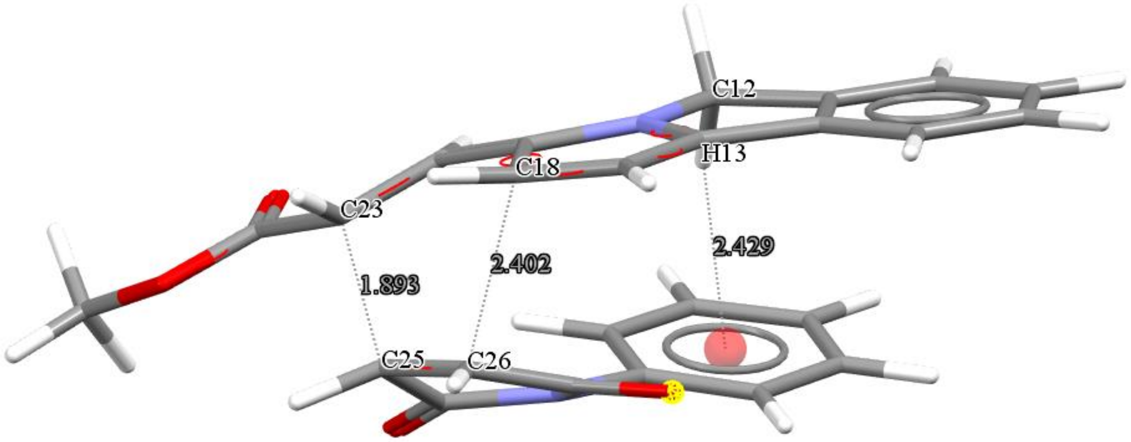

Atom	Distance (Å)	Angle (°)	Contact Type
C23··· C25	1.893	*a*	
C18··· C26	2.402	*a*	
C12-H13···π	2.429	154.71	D-H ··· A

[a] No determined

Appendix 7.

Experimental section

Melting points were determined on a Krüss KSP 1N capillary melting point apparatus. IR spectra were recorded on a Perkin-Elmer 2000 spectrophotometer. ^{1}H and ^{13}C NMR spectra were captured on Varian Mercury (300 MHz), Varian VNMR (500 MHz) and Bruker 600AVANCE III (600 MHz) instruments, with $CDCl_3$ as the solvent and TMS as internal standard. Signal assignments were based on 2D NMR spectra (HMQC, HMBC and ROESY). Mass spectra (MS) were recorded on Thermo Polaris Q-Trace GC Ultra and Hewlett-Packard 5971A spectrometers. High-resolution mass spectra (HRMS) were obtained (in electron impact mode) on a Jeol JSM-GCMateII spectrometer. Elemental analyses were performed on a CE-440 Exeter Analytical instrument. Analytical thin-layer chromatography was carried out using E. Merck silica gel 60 F_{254} coated 0.25 plates, visualized by using a long- and short-wavelength UV lamp. Flash column chromatography was done over Natland International Co. silica gel (230–400 mesh). All air moisture sensitive reactions were carried out under N_2 using oven-dried glassware. THF and toluene was freshly distilled over sodium, as were DMF, DMA, MeCN and CH_2Cl_2 over CaH_2, prior to use. DMSO and acetone were dried by distillation following treatment with 4Å molecular sieves. EtOH and MeOH were distilled over sodium. Et_3N was freshly distilled from NaOH. K_2CO_3 and Li_2CO_3 were dried overnight at 200 °C prior to use. All other reagents were employed without further purification. Compounds **16a-c** and **8i-j** were prepared as described [S1,S2,S3,S4].

1-Benzyl-1*H*-pyrrole-2-carbaldehyde (13b) [S5]**.** To a solution of **13a** (0.250 g, 2.63 mmol) in anhydrous DMF (3.0 mL), at 0 °C and under N_2, NaH (60%, 0.158 g, 3.95 mmol) was added. The mixture was stirred at 0 °C for 15 min and **14a** (0.674 g, 3.94 mmol) was added dropwise. After stirring at 0 °C for 4 h, EtOAc (40 mL) was added and washed with H_2O (2 × 20 mL). The organic layer was dried (Na_2SO_4) and the solvent removed under vacuum. The residue was purified by column chromatography over silica gel (20 g/g crude, hexane/EtOAc, 98:2) to give **13b** (0.480 g, 99%) as an amber oil. R*f* 0.68 (hexane/EtOAc, 7:3); IR (film): ῡ 1656, 1476, 1402, 1368, 1315, 1217, 1072, 1027, 758, 709 cm^{-1}; ^{1}H NMR

(500 MHz, $CDCl_3$) δ 5.51 (s, 2H, CH_2Ph), 6.23 (t, *J* = 3.5 Hz, 1H, H-4), 6.93 (d, *J* = 3.5 Hz, 2H, H-3, H-5), 7.10–7.13 (m, 2H, H-2'), 7.20–7.24 (m, 1H, H.4'), 7.25–7.29 (m, 2H, H-3'), 9.53 (s, 1H, C*H*O); ^{13}C NMR (125 MHz, $CDCl_3$) δ 51.9 (CH_2Ph), 110.2 (C-4), 124.8 (C-3), 127.3 (C-2'), 127.7 (C-4'), 128.7 (C-3'), 131.4 (C-5), 131.5 (C-2), 137.6 (C-1'), 179.5 (*C*HO); MS (70 eV): *m/z* 185 (M^+, 100), 184 (26), 168 (17), 167 (30), 156 (14), 91 (54), 65 (19); HRMS (EI): *m/z* [M^+] calcd for $C_{12}H_{11}NO$: 185.0841; found: 185.0842.

1-(2-Bromobenzyl)-1*H*-pyrrole-2-carbaldehyde (13c) [S6]**.** Following the method of preparation for **13b**, a mixture of **13a (**0.500 g, 5.26 mmol), NaH (60%, 0.274 g, 6.84 mmol) and **14b** (1.447 g, 5.79 mmol) in anhydrous DMF (5.0 mL) afforded **13c** (1.335 g, 96%) as a white solid. R*f* 0.68 (hexane/EtOAc, 7:3); mp 93–94 °C [Lit [89] 90–92 °C]; IR (KBr): ῡ 3103, 2801, 1656, 1476, 1400, 1363, 1319, 1224, 1078, 1024, 766, 746 cm^{-1}; ^{1}H NNR (500 MHz, $CDCl_3$) δ 5.65 (s, 2H, CH_2Ar), 6.30 (dd, *J* = 4.0, 2.5 Hz, 1H, H-4), 6.68 (d, *J* = 7.5 Hz, 1H, H-6'), 6.96 (br s, 1H, H-5), 7.01 (dd, *J* = 4.0, 2.0 Hz, 1H, H-3), 7.13 (t, *J* = 7.5 Hz, 1H, H-4'), 7.19 (t, *J* = 7.5 Hz, 1H, H-5'), 7.57 (d, *J* = 7.5 Hz, 1H, H-3'), 9.58 (s, 1H, C*H*O); ^{13}C NMR (125 MHz, $CDCl_3$) δ 25.0 (*C*H_2Ar), 110.3 (C-4), 122.6 (C-2'), 124.7 (C-3), 127.8 (C-5'), 128.2 (C-6'), 129.1 (C-4'), 131.4 (C-5), 131.7 (C-2), 132.8 (C-3'), 137.1 (C-1'), 179.4 (*C*HO); MS (70 eV): *m/z* 265 (M^++1, 1), 185 (29), 184 (100), 171 (35), 169 (35), 156 (31), 129 (23), 91 (19), 90 (22); HRMS (EI): *m/z* [M^+] calcd for $C_{12}H_{10}BrNO$: 262.9946; found: 262.9942.

1-(3-Methoxybenzyl)-1*H*-pyrrole-2-carbaldehyde (13d) [S7]**.** Following the method of preparation for **13b**, a mixture of **13a (**0.500 g, 5.26 mmol), NaH (60%, 0.274 g, 6.84 mmol) and **14c** (1.164 g, 5.79 mmol) in anhydrous DMF (6.0 mL) provided **13d** (1.020 g, 90%) as a reddish solid. R*f* 0.70 (hexane/EtOAc, 7:3); mp 40–41 °C; IR (KBr): ῡ 3127, 2808, 1662, 1608, 1583, 1488, 1423, 1402, 1370, 1320, 1287, 1261, 1143, 1049, 862, 787, 758, 689 cm^{-1}; ^{1}H NMR (500 MHz, $CDCl_3$) δ 3.76 (s, 3H, CH_3O), 5.54 (s, 2H, CH_2Ar), 6.26–6.28 (m, 1H, H-4), 6.67 (br s, 1H, H-2'), 6.73 (dm, *J* = 8.0 Hz, 1H, H-6'), 6.79 (dd, *J* = 8.0, 3.0 Hz, 1H, H-4'), 6.97 (d, *J* = 4.0 Hz, 2H, H-3, H-5), 7.22 (t, *J* = 8.0 Hz, 1H, H-5'), 9.56 (d, *J* = 0.5 Hz, 1H, C*H*O); ^{13}C NMR (125 MHz, $CDCl_3$) δ 51.8 (*C*H_2Ar), 55.1 (*C*H_3O), 110.1 (C-4), 112.97 (C-2' or C-4'), 112.99 (C-4' or C-2'), 119.5 (C-6'), 124.8 (C-3), 129.7

(C-5'), 131.4 (C-5), 131.6 (C-2), 139.1 (C-1'), 159.9 (C-3'), 179.5 (*C*HO); MS (70 eV): *m/z* 215 (M^+, 83), 214 (51), 198 (51), 197 (100), 154 (14), 122 (34), 121 (64), 91 (58), 77 (27); HRMS (EI): *m/z* [M^+] calcd for $C_{13}H_{13}NO_2$: 215.0946; found: 215.0937.

1-(2-Bromo-4,5-dimethoxybenzyl)-1*H*-pyrrole-2-carbaldehyde (13e). Following the method of preparation for **13b**, a mixture of **13a (**0.181 g, 1.91 mmol), NaH (60%, 0.090 g, 2.25 mmol), and **14d** (0.536 g, 1.73 mmol) in anhydrous DMF (6.0 mL), after stirring at 0 °C for 12 h, produced **13e** (0.543 g, 97%) as a white solid. R*f* 0.58 (hexane/EtOAc, 7:3); mp 111–112 °C; IR (KBr): ῡ 2937, 1665, 1505, 1424, 1402, 1372, 1321, 1262, 1206, 1157, 1081, 1028, 844, 761 cm^{-1}; ^1H NMR (300 MHz, $CDCl_3$) δ 3.71 (s, 3H, CH_3O-5'), 3.85 (s, 3H, CH_3O-4'), 5.59 (s, 2H, CH_2Ar), 6.26 (dd, *J* = 3.9, 2.7 Hz, 1H, H-4), 6.55 (s, 1H, H-6'), 6.99 (dd, *J* = 3.9, 1.8 Hz, 1H, H-3), 7.03 (s, 1H, H-3'), 7.02–7.08 (m, 1H, H-5), 9.58 (d, *J* = 0.9 Hz, 1H, C*H*O); ^{13}C NMR (75.4 MHz, $CDCl_3$) δ 51.3 (CH_2Ar), 55.8 (CH_3O-5'), 56.1 (CH_3O-4'), 110.2 (C-4), 111.9 (C-6'), 113.2 (C-2'), 115.2 (C-3'), 125.0 (C-3), 128.7 (C-1'), 131.4 (C-2, C-5), 148.6 (C-5'), 149.0 (C-4'), 179.5 (*C*HO); MS (70 eV): *m/z* 323 (M^+-1,1), 244 (100), 229 (30), 216 (20), 185 (15), 151 (10), 107 (8). HRMS (EI): *m/z* [M^+] calcd for $C_{14}H_{14}NO_3Br$: 323.0157; found: 323.0163.

Ethyl (*E*)-3-(1-benzyl-1*H*-pyrrol-2-yl)acrylate (8a) [S8]**.** Analogous procedure as described in [S1]. To a solution of **13b** (0.500 g, 2.70 mmol) in anhydrous THF (3.0 mL) at 0 °C and under N_2, NaH (60%, 0.162 g, 4.05 mmol) was added. The mixture was stirred at 0 °C for 30 min before triethyl phosphonoacetate (**15b**) (0.726 g, 3.24 mmol) in anhydrous THF (5.0 mL) was added dropwise. After stirring at room temperature for 12 h, EtOAc (50 mL) was added, and the mixture was washed with water (2 × 25 mL). The organic layer was dried (Na_2SO_4) and the solvent removed under vacuum. The residue was purified by column chromatography over silica gel (20 g/g crude, hexane/EtOAc, 9:1) to furnish **8a** (0.675 g, 98%) as a colorless oil. R*f* 0.66 (hexane/EtOAc, 9:1 x 3); IR (film): ῡ 2979, 1697, 1620, 1471, 1453, 1398, 1365, 1325, 1275, 1159, 1077, 1031, 967, 850, 721, 610 cm^{-1}; ^1H NMR (500 MHz, $CDCl_3$) δ 1.28 (t, *J* = 7.0 Hz, 3H, $CO_2CH_2CH_3$), 4.18 (q, *J* = 7.0 Hz, 2H, $CO_2CH_2CH_3$), 5.20 (s, 2H, CH_2Ph), 6.13 (d, *J* = 16.0 Hz, 1H, H-2), 6.24 (t, *J* = 3.5 Hz, 1H, H-4'), 6.72 (d, *J* = 3.5 Hz, 1H, H-3'), 6.82 (br s, 1H, H-5'), 7.01–7.03 (m, 2H, H-2''), 7.22–

7.28 (m, 1H, H-4”), 7.31 (t, *J* = 7.5 Hz, 2H, H-3”), 7.55 (d, *J* = 16.0 Hz, 1H, H-3); ^{13}C NMR (125 MHz, $CDCl_3$) δ 14.3 ($CO_2CH_2\textit{C}H_3$), 50.7 (*C*H_2Ph), 60.1 ($CO_2\textit{C}H_2CH_3$), 109.9 (C-4’), 111.8 (C-3’), 113.4 (C-2), 126.3 (C-5’), 126.4 (C-2”), 127.8 (C-4”), 128.9 (C-3”), 129.1 (C-2’), 132.1 (C-3), 137.3 (C-1”), 167.6 (*C*O_2Et); MS (70 eV): *m/z* 255 (M^+, 26), 203 (8), 182 (23), 168 (100), 167 (32), 115 (6), 91 (44); HRMS (EI): *m/z* [M^+] calcd for $C_{16}H_{17}NO_2$: 255.1259; found: 255.1253.

(*E*)-3-(1-Benzyl-1*H*-pyrrol-2-yl)acrylonitrile ((*E*)-8b). (*Z*)-3-(1-Benzyl-1*H*-pyrrol-2-yl)acrylonitrile ((*Z*)-8b). Following the method of preparation for **8a**, a mixture of **13b** (0.500 g, 2.70 mmol), NaH (60%, 0.162 g, 4.05 mmol), and diethyl (cyanomethyl)phophonate (**15c**) (0.574 g, 3.24 mmol) in anhydrous THF (10.0 mL) generated a mixture of (***E***)**-8b**/**(*Z*)-8b** (95:5, 0.556 g, 99%) as a colorless oil. R*f* 0.75 (hexane/EtOAc, 7:3); IR (film): ῡ 2206, 1607, 1472, 1443, 1413, 1324, 1298, 1081, 948, 730, 706 cm^{-1}; ^{1}H NMR (500 MHz, $CDCl_3$) δ 5.16 (s, 2H, C$\textit{H}_2$Ph), 5.45 (d, *J* = 16.0 Hz, 1H, H-2), 6.26 (dd, *J* = 4.0, 2.5 Hz, 1H, H-4’), 6.70 (dd, *J* = 4.0, 1.5 Hz, 1H, H-3’), 6.88 (dd, *J* = 2.5, 1.5 Hz, 1H, H-5’), 6.97–7.01 (m, 2H, H-2”), 7.10 (d, *J* = 16.5 Hz, 1H, H-3), 7.27–7.37 (m, 3H, H-3”, H-4”); signals attributed to the minor isomer **(*Z*)-8b**: δ 4.94 (d, *J* = 11.5 Hz, H-2), 5.18 (s, C$\textit{H}_2$Ph), 6.34 (dd, *J* = 4.0, 2.5 Hz, H-4’), 6.82 (d, *J* = 12.0 Hz, H-3), 7.44 (dd, *J* = 4.0, 1.5 Hz, H-5); ^{13}C NMR (125 MHz, $CDCl_3$) δ 50.9 (C$\textit{H}_2$Ph), 90.1 (C-2), 110.2 (C-4’), 112.2 (C-3’), 119.3 (CN), 126.0 (C-2”), 127.4 (C-5’), 128.0 (C-4”), 128.3 (C-2’), 129.4 (C-3”), 136.7 (C-1”), 137.4 (C-3); signals attributed to the minor isomer **(*Z*)-8b**: δ 50.7, 87.8, 114.7, 118.5, 126.0, 126.5, 127.9, 129.0, 134.8, 137.0; MS (70 eV): *m/z* 208 (M^+, 65), 168 (52), 167 (28), 91 (100), 65 (17); HRMS (EI): *m/z* [M^+] calcd for $C_{14}H_{12}N_2$: 208.1001; found: 208.1002.

Methyl (*E*)-3-(1-(2-bromobenzyl)-1*H*-pyrrol-2-yl)acrylate (8c). Following the method of preparation for **8a**, a mixture of **13c** (0.500 g, 1.89 mmol), NaH (60%, 0.113 g, 2.84 mmol), and trimethyl phophonoacetate (**15a**) (0.413 g, 2.27 mmol) in anhydrous THF (10.0 mL) gave **8c** (0.600 g, 99%) as a colorless solid. R*f* 0.73 (hexane/EtOAc, 7:3); mp 85-86 °C; IR (KBr): ῡ 3122, 2943, 1705, 1620, 1463, 1431, 1328, 1284, 1204, 1169, 1126, 1078, 1023, 965, 848, 756, 739 cm^{-1}; RMN ^{1}H (300 MHz, $CDCl_3$) δ 3.72 (s, 3H, $CO_2C\textit{H}_3$), 5.24 (s, 2H,

CH_2N), 6.13 (d, *J* = 15.6 Hz, 1H, H-2), 6.29 (ddd, *J* = 3.8, 2.7, 0.6 Hz, 1H, H-4'), 6.47 (dm, *J* = 7.4 Hz, 1H, H-6"), 6.76 (dd, *J* = 3.8, 1.8 Hz, 1H, H-3'), 6.81 (dd, *J* = 2.7, 1.8 Hz, 1H, H-5'), 7.13 (tm, *J* = 7.4 Hz, 1H, H-4"), 7.19 (td, *J* = 7.4, 1.5 Hz, 1H, H-5"), 7.47 (d, *J* = 15.6 Hz, 1H, H-3), 7.58 (dd, *J* = 7.4, 1.8 Hz, 1H, H-3"); RMN ^{13}C (75.4 MHz, $CDCl_3$) δ 50.8 (*C*H_2N), 51.4 (CO_2*C*H_3), 110.3 (C-4'), 112.2 (C-3'), 113.2 (C-2), 121.7 (C-2"), 126.5 (C-5'), 127.6 (C-6"), 128.0 (C-5"), 129.1 (C-2'), 129.2 (C-4"), 131.9 (C-3), 132.7 (C-3"), 136.6 (C-1"), 167.9 (*C*O_2CH_3); EM (70 eV): *m/z* 321 (M^++1, 51), 320 (M^+, 8), 319 (M^+-1, 55), 262 (14), 248 (51), 240 (100), 208 (76), 181 (61), 180 (89), 171 (55), 169 (55), 152 (16), 90 (40); HRMS (EI): *m/z* [M^+] calcd for $C_{15}H_{14}BrNO_2$: 319.0208; found: 319.0201; Anal calcd for $C_{15}H_{14}BrNO_2$: C, 56.27; H, 4.41; N, 4.37; found: C, 56.27; H, 4.39; N, 4.36.

Ethyl (*E*)-3-(1-(2-bromobenzyl)-1*H*-pyrrol-2-yl)acrylate (8d). Following the method of preparation for **8a**, a mixture of **13c** (0.500 g, 1.89 mmol), NaH (60%, 0.091 g, 2.27 mmol), and **15b** (0.467 g, 2.08 mmol) in anhydrous THF (10.0 mL) afforded **8d** (0.587 g, 93%) as an orange solid. R*f* 0.73 (hexane/EtOAc, 7:3); mp 58–59 °C; IR (KBr): ῡ 2978, 1699, 1626, 1462, 1437, 1327, 1288, 1202, 1172, 1026, 960, 757, 733 cm^{-1}; ^{1}H NMR (500 MHz, $CDCl_3$) δ 1.27 (t, *J* = 7.0 Hz, 3H, CO_2CH_2C*H_3*), 4.18 (q, *J* = 7.0 Hz, 2H, CO_2C*H_2*CH_3), 5.25 (s, 2H, C*H_2*Ar), 6.14 (d, *J* = 15.5 Hz, 1H, H-2), 6.28 (t, *J* = 3.5 Hz, 1H, H-4'), 6.49 (d, *J* = 7.5 Hz, 6"), 6.75 (d, *J* = 3.5 Hz, 1H, H-3'), 6.81 (br s, 1H, H-5'), 7.14 (t, *J* = 7.5 Hz, 1H, H-4"), 7.20 (t, *J* = 7.5 Hz, 1H, H-5"), 7.46 (d, *J* = 15.5 Hz, 1H, H-3), 7.58 (d, *J* = 7.5 Hz, 1H, H-3"); ^{13}C NMR (125 MHz, $CDCl_3$) δ 14.3 (CO_2CH_2*C*H_3), 50.8 (*C*H_2Ar), 60.2 (CO_2*C*H_2CH_3), 110.2 (C-4'), 112.0 (C-3'), 113.7 (C-2), 121.8 (C-2"), 126.4 (C-5'), 127.6 (C-6"), 128.0 (C-5"), 129.2 (C-4"), 129.3 (C-2'), 131.7 (C-3), 132.7 (C-3"), 136.7 (C-1"), 167.5 (*C*O_2Et); MS (70 eV): *m/z* 335 (M^++1), 333 (M^+-1, 49), 254 (100), 248 (76), 246 (58), 226 (23), 208 (86), 181 (61), 180 (83), 169 (64), 90 (27); HRMS (EI): *m/z* [M^+] calcd for $C_{16}H_{16}BrNO_2$: 333.0364; found: 333.0358.

Ethyl (*E*)-3-(1-(3-methoxybenzyl)-1*H*-pyrrol-2-yl)acrylate (8e). Following the method of preparation for **8a**, a mixture of **13d** (0.500 g, 2.33 mmol), NaH (60%, 0.112 g, 2.79 mmol) and **15b** (0.574 g, 2.56 mmol) in anhydrous THF (10.0 mL) provided **8e** (0.598 g, 90%) as a colorless oil. R*f* 0.66 (hexane/EtOAc, 7:3); IR (film): ῡ 2932, 1699, 1620, 1469,

1324, 1260, 1165, 1038, 966, 778, 727 cm^{-1}; ^{1}H NMR (500 MHz, $CDCl_3$) δ 1.28 (t, *J* = 7.0 Hz, 3H, $CO_2CH_2CH_3$), 3.75 (s, 3H, CH_3O), 4.18 (q, *J* = 7.0 Hz, 2H, $CO_2CH_2CH_3$), 5.17 (s, 2H, CH_2Ar), 6.12 (d, *J* = 15.5 Hz, 1H, H-2), 6.24 (br s, 1H, H-4'), 6.56 (br s, 1H, H-2"), 6.63 (d, *J* = 7.5 Hz, 1H, H-6"), 6.71 (d, *J* = 4.0 Hz, 1H, H-3'), 6.79 (d, *J* = 7.5 Hz, 1H, H-4"), 6.82 (br s, 1H, H-5'), 7.22 (t, *J* = 7.5 Hz, 1H, H-5"), 7.54 (d, *J* = 15.5 Hz, 1H, H-3); ^{13}C NMR (125 MHz, $CDCl_3$) δ 14.3 ($CO_2CH_2CH_3$), 50.6 (CH_2Ar), 55.2 (CH_3O), 60.1 ($CO_2CH_2CH_3$), 109.9 (C-4'), 111.9 (C-3'), 112.2 (C-2"), 113.1 (C-4"), 113.4 (C-2), 118.7 (C-6"), 126.4 (C-5'), 129.2 (C-2'), 129.9 (C-5"), 132.1 (C-3), 138.9 (C-1"), 160.1 (C-3"), 167.6 (CO_2Et); EM (70 eV): *m/z* 285 (M^+, 54), 212 (76), 198 (100), 197 (58), 145 (14), 121 (46), 91 (30), 77 (13); HRMS (EI): *m/z* [M^+] calcd for $C_{17}H_{19}NO_3$: 285.1365; found: 285.1370.

Methyl (*E*)-3-(1-(2-bromo-4,5-dimethoxybenzyl)-1*H*-pyrrol-2-yl)acrylate (8f). Following the method of preparation for **8a**, a mixture of **13e** (0.500 g, 1.54 mmol), NaH (60%, 0.093 g, 2.31 mmol) and **15a** (0.337 g, 1.85 mmol) in anhydrous THF (10.0 mL) produced **8f** (0.573 g, 98%) as a white solid. R*f* 0.55 (hexane/EtOAc, 7:3); mp 71–72 °C; IR (KBr): ῡ 2945, 2837, 1710, 1622, 1510, 1470, 1437, 1327, 1283, 1258, 1219, 1191, 1162, 1029, 967, 851, 804, 717 cm^{-1}; ^{1}H NMR (300 MHz, $CDCl_3$) δ 3.61 (s, 3H, CH_3O-5"), 3.71 (s, 3H, CO_2CH_3), 3.83 (s, 3H, CH_3O-4"), 5.15 (s, 2H, CH_2N), 6.06 (s, 1H, H-6"), 6.12 (d, *J* = 15.5 Hz, 1H, H-2), 6.25 (dd, *J* = 3.9, 3.2 Hz, 1H, H-4'), 6.74 (dd, *J* = 3.9, 1.7 Hz, 1H, H-3'), 6.82 (dd, *J* = 2.6, 1.7 Hz, 1H, H-5'), 7.02 (s, 1H, H-3"), 7.52 (d, *J* = 15.5 Hz, 1H, H-3); ^{13}C NMR (75.4 MHz, $CDCl_3$) δ 50.2 (CH_2N), 51.2 (CO_2CH_3), 55.5 (CH_3O-5"), 55.9 (CH_3O-4"), 109.9 (C-4'), 110.3 (C-6"), 111.7 (C-2"), 112.0 (C-3'), 112.6 (C-2), 115.1 (C-3"), 126.3 (C-5'), 128.1 (C-1"), 128.7 (C-2'), 131.8 (C-3), 148.6 (C-5"), 148.8 (C-4"), 167.6 (CO_2CH_3); MS (70 eV): *m/z* 381 (M^++1, 8), 380 (M^+, 2), 379 (M^+-1, 10), 300 (100), 268 (15), 231 (76), 229 (78), 107 (10); HRMS (EI): *m/z* [M^+] calcd for $C_{17}H_{18}BrNO_4$: 379.0419; found: 379.0426.

(*E*)-4-(1-(2-Bromobenzyl)-1*H*-pyrrol-2-yl)but-3-en-2-one (8g). In a threaded ACE glass pressure tube equipped with sealed Teflon screw cap and a magnetic stirring bar, a solution of KOH (0.096 g, 1.71 mmol), acetone (0.086 g, 1.48 mmol) and **13c** (0.300 g, 1.14 mmol)

in MeOH (3 mL) was heated at 100 °C for 4 h. The mixture was diluted with EtOAc (50 mL) and washed with water (2 x 25 mL), then the organic layer was dried (Na_2SO_4) and the solvent removed under vacuum. The residue was purified by column chromatography over silica gel (20 g/g crude, hexane/EtOAc, 95:5) to obtain **8g** (0.281 g, 81%) as a yellow oil. R*f* 0.62 (hexane/EtOAc, 7:3); IR (KBr): ῡ 3107, 1658, 1615, 1469, 1439, 1416, 1357, 1325, 1279, 1252, 1198, 1080, 1025, 971, 820, 752, 730 cm^{-1}; ^1H NMR (500 MHz, $CDCl_3$) δ 2.21 (s, 3H, CH_3CO), 5.26 (s, 2H, CH_2Ar), 6.30 (t, *J* = 3.5 Hz, 1H, H-4’), 6.45 (d, *J* = 16.0 Hz, 1H, H-3), 6.52 (dm, *J* = 7.5 Hz, 1H, H-6”), 6.79 (dd, *J* = 4.0, 1.5 Hz, 1H, H-3’), 6.87 (dd, *J* = 2.5, 1.5 Hz, 1H, H-5’), 7.15 (td, *J* = 7.5, 2.0 Hz, 1H, H-4”), 7.21 (td, *J* = 7.5, 1.5 Hz, 1H, H-5”), 7.29 (d, *J* = 16.0 Hz, 1H, H-4), 7.59 (dd, *J* = 7.5, 1.0 Hz, 1H, H-3”); ^{13}C NMR (125 MHz, $CDCl_3$) δ 27.8 (CH_3CO), 50.9 (CH_2Ar), 110.5 (C-4’), 112.8 (C-3’), 121.7 (C-2”), 122.6 (C-3), 127.3 (C-5’), 127.7 (C-6”), 128.1 (C-5”), 129.1 (C-2’), 129.3 (C-4”), 130.3 (C-4), 132.7 (C-3”), 136.5 (C-1”), 197.5 (CH_3CO); MS (70 eV): *m/z* 305 (M^++1, 46), 304 (M^+, 44), 303 (M^+-1, 44), 262 (31), 260 (33), 247 (18), 224 (100), 181 (78), 180 (72), 171 (91), 169 (95), 90 (37); HRMS (EI): *m/z* [M^+] calcd for $C_{15}H_{14}BrNO$: 303.0259; found: 303.0267.

(*E*)-4-(1-(2-Bromo-4,5-dimethoxybenzyl)-1*H*-pyrrol-2-yl)but-3-en-2-one (8h). Following the method of preparation for **8g**, a mixture of **13e** (0.500 g, 1.54 mmol), KOH (0.130 g, 2.31 mmol) and acetone (0.116 g, 2.00 mmol) in MeOH (3.0 mL), furnished **8h** (0.405 g, 72%) as a yellow solid. R*f* 0.36 (hexane/EtOAc, 7:3); mp 107–108 °C; IR (KBr): ῡ 2934, 1660, 1588, 1508, 1470, 1258, 1211, 1161, 1029, 962, 730 cm^{-1}; ^1H NMR (300 MHz, $CDCl_3$) δ 2.25 (s, 3H, CH_3CO), 3.64 (s, 3H, CH_3O), 3.87 (s, 3H, CH_3O), 5.20 (s, 2H, CH_2Ar), 6.11 (s, 1H, H-6”), 6.29 (ddd, *J* = 3.9, 2.7, 0.6 Hz, 1H, H-4’), 6.46 (d, *J* = 15.8 Hz, 1H, H-3), 6.79 (dd, *J* = 3.9, 1.5 Hz, 1H, H-3’), 6.88 (dd, *J* = 2.7, 1.5 Hz, 1H, H-5’), 7.04 (s, 1H, H-3”), 7.36 (d, *J* = 15.8 Hz, 1H, H-4); ^{13}C NMR (74.5 MHz, $CDCl_3$) δ 27.8 (CH_3CO), 50.6 (CH_2Ar), 55.8 (CH_3O), 56.2 (CH_3O), 110.3 (C-4’), 110.6 (C-6”), 111.9 (C-2”), 112.8 (C-3’), 115.3 (C-3”), 122.4 (C-3), 127.2 (C-5’), 128.2 (C-1”), 129.0 (C-2’), 130.5 (C-4), 148.9 (C-4” or C-5”), 149.1 (C-5” or C-4”), 197.7 (CH_3CO); HRMS (EI): *m/z* [M^+] calcd for $C_{17}H_{18}BrNO_3$: 363.0470; found: 363.0477.

Dimethyl 2-((1-(2-bromobenzyl)-1*H*-pyrrol-2-yl)methylene)malonate (8k). Following the method of preparation for **13b**, a mixture of **16c** (0.350 g, 1.67 mmol), NaH (60%, 0.100 g, 2.51 mmol) and **14b** (0.500 g, 2.00 mmol) in anhydrous DMF (5.0 mL), after stirring at rt for 1 h, resulted in **8k** (0.392 g, 62%) as a yellow solid. R*f* 0.40 (hexane/EtOAc, 8:2); mp 95–96 °C; IR (film): ῡ 2950, 1716, 1609, 1472, 1436, 1416, 1363, 1313, 1205, 1069, 1026, 735 cm^{-1}; ^{1}H NMR (500 MHz, $CDCl_3$) δ 3.75 (s, 3H, CO_2CH_3), 3.89 (s, 3H, CO_2CH_3), 5.26 (s, 2H, CH_2Ar), 6.30 (ddd, *J* = 4.0, 2.5, 0.5 Hz, 1H, H-4”), 6.54 (dm, *J* = 7.5 Hz, 1H, H-6’”), 6.69 (ddd, *J* = 4.0, 1.5, 0.5 Hz, 1H, H-3”), 6.88 (dd, *J* = 2.5, 1.5 Hz, 1H, H-5”), 7.14 (td, *J* = 7.5, 2.0 Hz, 1H, H-4’”), 7.20 (td, *J* = 7.5, 1.5 Hz, 1H, H-5’”), 7.50 (s, 1H, H-1’), 7.58 (dd, *J* = 8.0, 1.0 Hz, 1H, H-3’”); ^{13}C NMR (125 MHz, $CDCl_3$) δ 50.7 (CH_2Ar), 52.2 (CO_2CH_3), 52.6 (CO_2CH_3), 111.0 (C-4”), 115.1 (C-3”), 118.9 (C-2), 121.8 (C-2’”), 126.3 (C-2”), 127.6 (C-5”), 127.8 (C-6’”), 128.1 (C-5’”), 129.1 (C-1’), 129.4 (C-4’”), 132.7 (C-3’”), 136.2 (C-1’”), 164.9 (CO_2CH_3), 167.5 (CO_2CH_3); HRMS (EI): *m/z* [M^+] calcd for $C_{17}H_{16}BrNO_4$: 377.0263; found: 377.0267.

Dimethyl 2-((1-(2-bromo-4,5-dimethoxybenzyl)-1*H*-pyrrol-2-yl)methylene)malonate (8l). Following the method of preparation for **13b**, a mixture of **16c** (0.200 g, 0.96 mmol), NaH (60%, 0.058 g, 1.45 mmol) and **14d** (0.329 g, 1.06 mmol) in anhydrous DMF (5.0 mL), after stirring at rt for 1 h, led to **8l** (0.126 g, 30%) as a red solid. R*f* 0.33 (hexane/EtOAc, 7:3); mp 86–67 °C; IR (film): ῡ 3417, 2955, 2841, 1732, 1615, 1435, 1207, 740 cm^{-1}; ^{1}H NMR (500 MHz, $CDCl_3$) δ 3.67 (s, 3H, CH_3O), 3.78 (s, 3H, CO_2CH_3), 3.87 (s, 3H, CH_3O), 3.89 (s, 3H, CO_2CH_3), 5.21 (s, 2H, CH_2Ar), 6.11 (s, 1H, H-6’”), 6.29 (dd, *J* = 4.0, 2.5, 0.5 Hz, 1H, H-3”), 6.66 (ddd, *J* = 4.0, 1.5, 0.5 Hz, 1H, H-4”), 6.89 (dd, *J* = 2.5, 1.5 Hz, 1H, H-5”), 7.04 (s, 1H, 3’”), 7.58 (s, 1H, H-1’); ^{13}C NMR (125 MHz, $CDCl_3$) δ 50.5 (CH_2Ar), 52.4 (CH_3O), 52.7 (CO_2CH_3), 55.8 (CH_3O), 56.2 (CO_2CH_3), 110.7 (C-6’”), 110.9 (C-4”), 112.1 (C-2’”), 115.1 (C-3”), 115.4 (C-4’”), 118.9 (C-2), 126.3 (C-2”), 127.6 (C-5”), 127.9 (C-1’”), 129.4 (C-1’), 148.9 (C-5’”), 149.2 (C-4’”), 164.9 (CO_2CH_3), 167.6 (CO_2CH_3); HRMS (EI): *m/z* [M^+] calcd for $C_{19}H_{20}BrNO_6$: 437.0474; found: 437.0476.

Methyl (3a*R,4*S**,8b*S**)-2-methyl-1,3-dioxo-1,2,3,3a,4,5,6,8b-octahydropyrrolo[3,4-*e*]indole-4-carboxylate (9a). Methyl (3a*R**,4*R**,8b*S**)-2-methyl-1,3-dioxo-**

1,2,3,3a,4,5,6,8b-octahydropyrrolo[3,4-*e*]indole-4-carboxylate (10a). In a threaded ACE glass pressure tube equipped with a sealed Teflon screw cap and a magnetic stirring bar, a solution of **16a** (0.500 g, 3.31 mmol) and *N*-methylmaleimide (**7b**) (0.441 g, 3.97 mmol) in toluene (3.0 mL) was heated at 150 °C for 7 days. The solvent was removed under vacuum, to generate a mixture of **9a**/**10a** (70:30) as an amber oil, which was purified by column chromatography over silica gel (20 g/g crude, hexane/EtOAc, 7:3) to provide **9a** (0.170 g, 15%) as a yellow solid, and a mixture of **9a**/**10a** (0.637 g, 57%) as a brown oil. Data of **9a**: R*f* 0.33 (hexane/EtOAc, 1:1); mp 182–184 °C; IR (film): ῡ 3383, 1732, 1698, 1436, 1385, 1279, 1247, 1064, 722 cm^{-1}; ^{1}H NMR (600 MHz, $CDCl_3$) δ 2.78 (dd, *J* = 16.5, 13.2 Hz, 1H, H-5), 2.88 (s, 3H, NCH_3), 2.91–3.00 (m, 2H, H-4, H-5), 3.82 (s, 3H, CO_2CH_3), 3.92 (dd, *J* = 7.8, 4.8 Hz, 1H, H-3a), 4.10 (d, *J* = 7.8 Hz, 1H, H-8b), 6.29 (t, *J* = 2.7 Hz, 1H, H-8), 6.67 (t, *J* = 2.7 Hz, 1H, H-7), 8.32 (br s, 1H, NH); signals attributed to the minor isomer **10a**: 2.96 (s, NCH_3), 3.81 (s, CO_2CH_3), 3.87 (dd, *J* = 8.3, 4.0 Hz, H-3a), 4.12 (d, *J* = 7.1 Hz, H-8b); ^{13}C NMR (150 MHz, $CDCl_3$) δ 21.2 (C-5), 24.6 (NCH_3), 38.6 (C-4), 40.8 (C-8b), 42.4 (C-3a), 52.3 (CO_2CH_3), 107.0 (C-8), 110.7 (C-8a), 117.8 (C-7), 125.4 (C-5a), 172.5 (CO_2CH_3), 177.1 (C-3), 177.6 (C-1); HRMS (EI): m/z [M^+] calcd for $C_{13}H_{14}N_2O_4$: 262.0954; found: 262.0953.

Ethyl (3a*R,4*S**,8b*S**)-2-methyl-1,3-dioxo-1,2,3,3a,4,5,6,8b-octahydropyrrolo[3,4-*e*]indole-4-carboxylate (9b). Ethyl (3a*R**,4*R**,8b*S**)-2-methyl-1,3-dioxo-1,2,3,3a,4,5,6,8b-octahydropyrrolo[3,4-*e*]indole-4-carboxylate (10b).** Following the method of preparation for **9a**/**10a**, a mixture of **16b** (0.500 g, 3.03 mmol) and **7b** (0.403 g, 3.63 mmol) in toluene (3.0 mL) gave a mixture of **9b**/**10b** (75:25) as an amber oil. Purification afforded **9b** (0.157 g, 19%) as a yellow oil and a mixture of **9b**/**10b** (0.461 g, 55%) as an amber oil. Data of **9b**: R*f* 0.29 (hexane/EtOAc, 1:1); IR (film): ῡ 3374, 2983, 2932, 1774, 1704, 1438, 1385, 1282, 1247, 1029, 724 cm^{-1}; ^{1}H NMR (500 MHz, $CDCl_3$) δ 1.33 (t, *J* = 7.3 Hz, 3H, $CO_2CH_2CH_3$), 2.79 (ddd, *J* = 16.5, 13.0, 1.5 Hz, 1H, H-5), 2.89 (s, 3H, NCH_3), 2.92–2.98 (m, 2H, H-4, H-5), 3.92 (dd, *J* = 8.0, 4.0 Hz, 1H, H-3a), 4.09 (d, *J* = 8.0 Hz, 1H, H-8b), 4.24–4.34 (m, 2H, $CO_2CH_2CH_3$), 6.30 (t, *J* = 2.7 Hz, 1H, H-8), 6.67 (t, *J* = 2.7 Hz, 1H, H-7), 8.20 (br s, 1H, NH); signals attributed to the minor isomer **10b**: δ 1.20 (t, *J* = 7.5 Hz, OCH_2CH_3), 2.88 (s, NCH_3), 3.70 (dd, *J* = 8.1, 5.6 Hz), 3.99 (d, *J* = 8.4 Hz);

^{13}C NMR (125 MHz, $CDCl_3$) δ 14.1 ($CO_2CH_2CH_3$), 21.2 (C-5), 24.7 (N-CH_3), 38.8 (C-4), 40.9 (C-8b), 42.5 (C-3a), 61.3 ($CO_2CH_2CH_3$), 107.1 (C-8), 110.9 (C-8a), 117.8 (C-7), 125.6 (C-5a), 172.0 ($CO_2CH_2CH_3$), 177.0 (C-3), 177.6 (C-1); HRMS (EI): m/z [M^+] calcd for $C_{14}H_{16}N_2O_4$: 276.1110, found: 276.1111.

Methyl (3a*R,4*S**,8b*S**)-1,3-dioxo-2-phenyl-1,2,3,3a,4,5,6,8b-octahydropyrrolo[3,4-*e*]indole-4-carboxylate (9c). Methyl (3a*R**,4*R**,8b*S**)-1,3-dioxo-2-phenyl-1,2,3,3a,4,5,6,8b-octahydropyrrolo[3,4-*e*]indole-4-carboxylate (10c).** Following the method of preparation for **9a**/**10a**, a mixture of **16a** (0.300 g, 1.98 mmol) and *N*-phenylmaleimide (**7c**) (0.412 g, 2.38 mmol) in toluene (2.0 mL) produced a mixture of **9c**/**10c** (94:6) as an amber oil. Purification led to the formation of **9c** (0.170 g, 15%) as a pale yellow solid and a mixture of **9c**/**10c** (0.648 g, 58%) as an amber oil. Data of **9c**: R*f* 0.36 (hexane/EtOAc, 7:3); mp 209–211 ºC; IR (film): ῡ 3369, 1710, 1498, 1385, 1246, 1196, 1128, 719 cm^{-1}; ^1H NMR (600 MHz, $CDCl_3$) δ 2.90 (dd, *J* = 15.6, 11.6 Hz, 1H, H-5), 2.99 (dd, *J* = 15.6, 4.8 Hz, 1H, H-5), 3.05 (dt, *J* = 11.6, 4.8 Hz, 1H, H-4), 3.82 (s, 3H, CO_2CH_3), 4.11 (br dd, *J* = 7.8, 4.2 Hz, 1H, H-3a), 4.23 (d, *J* = 7.8 Hz, 1H, H-8b), 6.34 (t, *J* = 2.6 Hz, 1H, H-8), 6.67 (t, *J* = 2.6 Hz, 1H, H-7), 7.21 (d, *J* = 7.7 Hz, 2H, H-2'), 7.31 (t, *J* = 7.7 Hz, 1H, H-4'), 7.39 (t, *J* = 7.7 Hz, 2H, H-3'), 8.09 (br s, 1H, N*H*); signals attributed to the minor isomer **10c**: δ 6.25 (t, *J* = 2.7 Hz, H-8), 6.53 (t, *J* = 2.7 Hz, H-7); ^{13}C NMR (150 MHz, $CDCl_3$) δ 21.2 (C-5), 38.6 (C-4), 41.1 (C-8b), 42.7 (C-3a), 52.4 (CO_2CH_3), 107.3 (C-8), 110.7 (C-8a), 118.0 (C-7), 125.6 (C-5a), 126.1 (C-2'), 128.3 (C-4'), 128.9 (C-3'), 131.7 (C-1'), 172.4 (CO_2CH_3), 176.0 (C-3), 176.3 (C-1); HRMS (EI): m/z [M^+] calcd for $C_{18}H_{16}N_2O_4$: 324.1110, found: 324.1108.

Ethyl (3a*R,4*S**,8b*S**)-1,3-dioxo-2-phenyl-1,2,3,3a,4,5,6,8b-octahydropyrrolo[3,4-*e*]indole-4-carboxylate (9d). Ethyl (3a*R**,4*R**,8b*S**)-1,3-dioxo-2-phenyl-1,2,3,3a,4,5,6,8b-octahydropyrrolo[3,4-*e*]indole-4-carboxylate (10d).** Following the method of preparation for **9a**/**10a**, a mixture of **16b** (0.280 g, 1.70 mmol) and **7c** (0.353 g, 2.04 mmol) in toluene (3.0 mL) generated a mixture of **9d**/**10d** (97:3) as a yellow oil. Purification furnished **9d** (0.409 g, 71%) as an amber oil. R*f* 0.51 (hexane/EtOAc, 1:1); IR (film): ῡ 3377, 1712, 1498, 1384, 1319, 1245, 1193, 1153, 1128, 1032, 718, 690 cm^{-1}; ^1H

NMR (500 MHz, $CDCl_3$) δ 1.31 (t, *J* = 7.0 Hz, 3H, $CO_2CH_2CH_3$), 2.88-2.99 (m, 2H, H-5), 3.03 (ddd, *J* = 9.0, 4.5, 2.5 Hz 1H, H-4), 4.12 (dd, *J* = 8.0, 4.5 Hz, 1H, H-3a), 4.22 (d, *J* = 8.0 Hz, 1H, H-8b), 4.29 (q, *J* = 7.0 Hz, 2H, $CO_2CH_2CH_3$), 6.35 (t, *J* = 2.5 Hz, 1H, H-8), 6.68 (t, *J* = 2.5 Hz, 1H, H-7), 7.19–7.23 (m, 2H, H-2'), 7.29–7.33 (m, 1H, H-4'), 7.37–7.42 (m, 2H, H-3'), 8.00 (br s, 1H, NH); ^{13}C NMR (125 MHz, $CDCl_3$) δ 14.1 ($CO_2CH_2CH_3$), 21.3 (C-5), 38.8 (C-4), 41.2 (C-8b), 42.7 (C-3a), 61.3 ($CO_2CH_2CH_3$), 107.4 (C-8), 110.8 (C-8a), 117.9 (C-7), 125.7 (C-5a), 126.1 (C-2'), 128.3 (C-4'), 128.9 (C-3'), 131.8 (C-1'), 171.8 (CO_2Et), 175.9 (C-3), 176.3 (C-1); MS (70 eV): m/z 338 (M^+, 44), 265 (20), 264 (82), 144 (13), 118 (50), 117 (100), 91 (12); HRMS (EI): m/z [M^+] calcd for $C_{19}H_{18}N_2O_4$: 338.1267; found: 338.1274.

Ethyl (3a*R,4*S**,8b*S**)-6-benzyl-1,3-dioxo-2-phenyl-1,2,3,3a,4,5,6,8b-octahydropyrrolo[3,4-*e*]indole-4-carboxylate (9e). Ethyl (3a*R**,4*R**,8b*S**)-6-benzyl-1,3-dioxo-2-phenyl-1,2,3,3a,4,5,6,8b-octahydropyrrolo[3,4-*e*]indole-4-carboxylate (10e).**

Following the method of preparation for **9a**/**10a**, a mixture of **8a** (0.250 g, 0.98 mmol) and **7c** (0.204 g, 1.18 mmol) in toluene (6.0 mL) gave a mixture of **9e**/**10e** (95:5) as an amber oil. Purification resulted in **9e** (0.386 g, 92%) as an amber oil and **10e** (0.013 g, 3%) as a dark oil, *Rf* 0.45 (hexane/EtOAc, 7:3 x 2). Data of **9e**: R*f* 0.55 (hexane/EtOAc, 7:3 x 2); IR (film): ῡ 1711, 1496, 1453, 1384, 1263, 1240, 1194, 1023, 710 cm^{-1}; ^1H NMR (500 MHz, $CDCl_3$) δ 1.28 (t, *J* = 7.5 Hz, 3H, $CO_2CH_2CH_3$), 2.73 (dd, *J* = 16.0, 12.0 Hz, 1H, H-5), 2.88 (dd, *J* = 16.0, 5.0 Hz, 1H, H-5), 2.99 (dt, *J* = 12.0, 5.0 Hz, 1H, H-4), 4.09 (dd, *J* = 8.0, 5.0 Hz, 1H, H-3a), 4.20–4.30 (m, 3H, H-8b, $CO_2CH_2CH_3$), 5.01 (s, 2H, CH_2Ph), 6.35 (d, *J* = 3.0 Hz, 1H, H-8), 6.64 (d, *J* = 3.0 Hz, 1H, H-7), 6.95 (br d, *J* = 7.0 Hz, 2H, H-2"), 7.19–7.22 (m, 2H, H-2'), 7.24–7-34 (m, 4H, H-4", H-3", H-4'), 7.28–7.42 (m, 2H, H-3'); signals attributed to the minor isomer **10e**: δ 1.09 (t, *J* = 7.0 Hz, OCH_2CH_3), 2.63 (dd, *J* = 16.0, 5.5 Hz, H-5), 4.97 (s, CH_2Ph), 6.32 (d, *J* = 3.0 Hz, H-8), 6.61 (d, *J* = 3.0 Hz, H-7); ^{13}C NMR (125 MHz, $CDCl_3$) δ 14.1 ($CO_2CH_2CH_3$), 20.2 (C-5), 38.8 (C-4), 41.4 (C-8b), 42.5 (C-3a), 50.1 (CH_2Ph), 61.3 ($CO_2CH_2CH_3$), 106.7 (C-8), 111.5 (C-8a), 122.2 (C-7), 126.2 (C-2'), 126.3 (C-2"), 126.7 (C-5a), 127.7 (C-4"), 128.3 (C-4'), 128.8 (C-3"), 128.9 (C-3'), 131.8 (C-1'), 137.6 (C-1"), 171.8 (CO_2Et), 175.9 (C-1), 176.4 (C-3); MS (70 eV): m/z 429

(M^++1, 21), 428 (M^+, 75), 355 (54), 354 (100), 234 (21), 208 (31), 207 (61), 91 (69); HRMS (EI): m/z [M^+] calcd for $C_{26}H_{24}N_2O_4$: 428.1736; found: 428.1749; Anal calcd for $C_{26}H_{24}N_2O_4$: C, 72.88; H, 5.65; N, 6.54; found: C, 72.89; H, 5.63; N, 6.58.

(3a*S,4*S**,8b*S**)-6-Benzyl-1,3-dioxo-2-phenyl-1,2,3,3a,4,5,6,8b-octahydropyrrolo[3,4-*e*]indole-4-carbonitrile (9f). (3a*S**,4*R**,8b*S**)-6-Benzyl-1,3-dioxo-2-phenyl-1,2,3,3a,4,5,6,8b-octahydropyrrolo[3,4-*e*]indole-4-carbonitrile (10f).** Following the method for the preparation of **9a**/**10a**, a mixture of **8b**(*E*)/**8b**(*Z*) (95:5, 0.510 g, 2.45 mmol) and **7c** (0.509 g, 2.94 mmol) in toluene (8.0 mL) afforded a mixture of **9f**/**10f** (76:24) as an amber oil. Purification, led to the formation of **9f** (0.682 g, 73%) as a yellow solid and **10f** (0.195 g, 21%) as an orange solid. Data of **9f**: R*f* 0.25 (hexane/EtOAc, 7:3): mp 163–164 ºC**;** IR (KBr): ῡ 2923, 2241, 1778, 1713, 1496, 1454, 1387, 1196, 734, 696 cm^{-1}; ^{1}H NMR (500 MHz, $CDCl_3$) δ 2.79–2.88 (m, 2H, H-5), 3.32–3.37 (m, 1H, H-4), 3.61 (ddd, *J* = 8.0, 4.3, 1.0 Hz, 1H, H-3a), 4.16 (d, *J* = 8.0 Hz, 1H, H-8b), 5.00 (s, 2H, CH_2Ph), 6.40 (d, *J* = 2.5 Hz, 1H, H-8), 6.70 (d, *J* = 2.5 Hz, 1H, H-7), 6.96 (br d, *J* = 8.0 Hz, 2H, H-2”), 7.23–7.26 (m, 2H, H-2’), 7.26–7.33 (m, 3H, H-3”, H-4”), 7.34–7.38 (m, 1H, H-4’), 7.40–7.45 (m, 2H, H-3’); ^{13}C NMR (125 MHz, $CDCl_3$) δ 22.8 (C-5), 25.9 (C-4), 40.1 (C-8b), 41.6 (C-3a), 50.5 (*C*H_2Ph), 107.0 (C-8), 110.9 (C-8a), 118.7 (CN), 123.0 (C-7), 123.9 (C-5a), 126.2 (C-2”), 126.3 (C-2’), 127.9 (C-4”), 128.6 (C-4’), 129.0 (C-3”), 129.1 (C-3’), 131.5 (C-1’), 137.0 (C-1”), 174.2 (C-3), 174.8 (C-1); MS (70 eV): m/z 382 (M^++1, 19), 381 (M^+, 68), 354 (61), 234 (19), 207 (41), 91 (100), 65 (12); HRMS (EI): m/z [M^+] calcd for $C_{24}H_{19}N_3O_2$: 381.1477; found: 381.1499; Anal calcd for $C_{24}H_{19}N_3O_2$: C, 75.57; H, 5.02; N, 11.02; found: C, 75.58; H, 5.00; N, 11.06. Data of **10f**: R*f* 0.50 (hexane/EtOAc, 7:3); mp 185–186 °C; IR (KBr): ῡ 2944, 2245, 1777, 1709, 1498, 1453, 1387, 1192, 743, 694 cm^{-1}; ^{1}H NMR (500 MHz, $CDCl_3$) δ 2.67 (dd, *J* = 16.0, 5.5 Hz, 1H, H-5), 2.81 (br d, *J* = 16.0 Hz, 1H, H-5), 3.70 (dd, *J* = 8.0, 2.5 Hz, 1H, H-3a), 3.82–3.87 (m, 1H, H-4), 4.33 (d, *J* = 8.0 Hz, 1H, H-8b), 5.01 (s, 2H. C*H*$_2$Ph), 6.38 (d, *J* = 3.0 Hz, 1H, H-8), 6.70 (d, *J* = 3.0 Hz, 1H, H-7), 6.93 (br d, *J* = 7.5 Hz, 2H, H-2”), 7.17–7.20 (m, 2H, H-2’), 7.24–7.28 (m, 1H, H-4”), 7.29–7.33 (m, 2H, H-3”), 7.34–7.38 (m, 1H, H-4’), 7.40–7.44 (m, 2H, H-3’); ^{13}C NMR (125 MHz, $CDCl_3$) δ 21.7 (C-5), 24.4 (C-4), 39.4 (C-8b), 42.6 (C-3a), 50.4 (*C*H_2Ph), 107.2 (C-8), 111.1 (C-8a), 119.9 (CN), 123.0 (C-5a), 123.2 (C-7), 126.0 (C-2”), 126.1 (C-2’),

127.8 (C-4”), 128.6 (C-4’), 129.0 (C-3”), 129.1 (C-3’), 131.4 (C-1’), 137.1 (C-1”), 174.1 (C-3), 175.3 (C-1); HRMS (EI): m/z [M$^+$] calcd for $C_{24}H_{19}N_3O_2$: 381.1477; found: 381.1462; Anal calcd for $C_{24}H_{19}N_3O_2$: C, 75.57; H, 5.02; N, 11.02; found: C, 75.56; H, 5.00; N, 11.04.

Methyl (3a*R,4*S**,8b*S**)-6-(2-bromobenzyl)-2-methyl-1,3-dioxo-1,2,3,3a,4,5,6,8b-octahydropyrrolo[3,4-*e*]indole-4-carboxylate (9g). Methyl (3a*R**,4*R**,8b*S**)-6-(2-bromobenzyl)-2-methyl-1,3-dioxo-1,2,3,3a,4,5,6,8b-octahydropyrrolo[3,4-*e*]indole-4-carboxylate (10g).** Following the method of preparation for **9a**/**10a**, a mixture of **8c** (0.432 g, 1.35 mmol) and **7b** (0.180 g, 1.62 mmol) in toluene (3.0 mL) generated a mixture of **9g**/**10g** (99:1) as a yellow oil. After purification, **9g** (0.506 g, 94%) was isolated as an orange oil. R*f* 0.24 (hexane/EtOAc, 7:3); IR (KBr): ῡ 2951, 2926, 1737, 1702, 1436, 1384, 1276, 1203, 1073, 1028, 752, 735 cm^{-1}; ^{1}H NMR (600 MHz, $CDCl_3$) δ 2.60 (dd, *J* = 15.9, 12.0 Hz, 1H, H-5), 2.77 (dd, *J* = 15.9, 4.8 Hz, 1H, H-5), 2.86 (s, 3H, NCH_3), 2.91 (dt, *J* = 12.0, 4.8 Hz, 1H, H-4), 3.75 (s, 3H, CO_2CH_3), 3.88 (dd, *J* = 7.8, 4.8 Hz, 1H, H-3a), 4.09 (d, *J* = 7.8 Hz, 1H, H-8b), 4.98 (br s, 2H, CH_2Ar), 6.31 (d, *J* = 2.4 Hz, 1H, H-8), 6.36 (br d, *J* = 7.2 Hz, 1H, H-6’), 6.56 (d, *J* = 2.4 Hz, 1H, H-7), 7.10 (dd, *J* = 7.8, 7.2 Hz, 1H, H-4’), 7.15 (dd, *J* = 7.8, 7.2 Hz, 1H, H-5’), 7.52 (d, *J* = 7.8 Hz, 1H, H-3’); ^{13}C NMR (150 MHz, $CDCl_3$) δ 19.8 (C-5), 24.5 (C-4), 38.5 (NCH_3), 40.8 (C-8b), 42.1 (C-3a), 50.1 (CH_2Ar), 52.1 (CO_2CH_3), 106.8 (C-8), 111.6 (C-8a), 121.7 (C-2’), 121.8 (C-7), 126.3 (C-5a), 127.3 (C-6’), 127.7 (C-5’), 129.0 (C-4’), 132.6 (C-3’), 136.7 (C-1’), 172.2 (CO_2CH_3), 176.8 (C-3), 177.3 (C-1); HRMS (EI): *m/z* [M$^+$] calcd for $C_{20}H_{19}N_2O_4Br$: 430.0528; found: 430.0530.

Methyl (3a*R,4*S**,8b*S**)-6-(2-bromobenzyl)-1,3-dioxo-2-phenyl-1,2,3,3a,4,5,6,8b-octahydropyrrolo[3,4-*e*]indole-4-carboxylate (9h). Methyl (3a*R**,4*R**,8b*S**)-6-(2-bromobenzyl)-1,3-dioxo-2-phenyl-1,2,3,3a,4,5,6,8b-octahydropyrrolo[3,4-*e*]indole-4-carboxylate (10h).** Following the method of preparation for **9a**/**10a**, a mixture of **8c** (0.540 g, 1.69 mmol) and **7c** (0.321 g, 1.86 mmol) in toluene (6.0 mL) provided a mixture of **9h**/**10h** (99:1) as a yellow oil. After purification, **9h** (0.819 g, 98%) was furnished as a yellow solid. R*f* 0.35 (hexane/EtOAc, 7:3); mp 198–199 °C; IR (KBr): ῡ 2942, 1739, 1713,

1597, 1497, 1445, 1387, 1357, 1325, 1229, 1194, 1161, 1129, 1028, 762, 714 cm^{-1}; ^{1}H NMR (300 MHz, $CDCl_3$) δ 2.72 (dd, *J* = 16.0, 12.0 Hz, 1H, H-5), 2.86 (dd, *J* = 16.0, 5.0 Hz, 1H, H-5), 3.03 (ddd, *J* = 12.0, 5.0, 4.4 Hz, 1H, H-4), 3.77 (s, 3H, CO_2CH_3), 4.11 (dd, *J* = 8.0, 4.4 Hz, 1H, H-3a), 4.26 (d, *J* = 8.0 Hz, 1H, H-8b), 5.04 (s, 2H, CH_2Ar), 6.27–6.34 (m, 1H, H-6''), 6.39 (d, *J* = 2.9 Hz, 1H, H-8), 6.62 (d, *J* = 2.9 Hz, 1H, H-7), 7.09–7.17 (m, 2H, H-4'', H-5''), 7.17–7.25 (m, 2H, H-2'), 7.30–7.36 (m, 1H, H-4'), 7.37–7.48 (m, 2H, H-3'), 7.54–7.58 (m, 1H, H-3''); ^{13}C NMR (75.4 MHz, $CDCl_3$) δ 19.9 (C-5), 38.5 (C-4), 41.3 (C-8b), 42.4 (C-3a), 50.3 (CH_2Ar), 52.4 (CO_2CH_3), 107.1 (C-8), 111.5 (C-8a), 121.8 (C-2''), 122.2 (C-7), 126.1 (C-2'), 126.6 (C-5a), 127.1 (C-6''), 127.9 (C-5''), 128.3 (C-4'), 128.9 (C-3'), 129.1 (C-4''), 131.6 (C-1'), 132.7 (C-3''), 136.9 (C-1''), 172.2 (CO_2CH_3), 175.8 (C-1), 176.2 (C-3); MS (70 eV): m/z 494 (M^++1, 23), 492 (M^+-1, 28), 434 (20), 413 (44), 353 (100), 314 (11), 288 (15), 206 (54), 169 (31); HRMS (EI): m/z [M^+] calcd for $C_{25}H_{21}BrN_2O_4$: 492.0685; found: 492.0685.

Ethyl (3a*R,4*S**,8b*S**)-6-(2-bromobenzyl)-2-methyl-1,3-dioxo-1,2,3,3a,4,5,6,8b-octahydropyrrolo[3,4-*e*]indole-4-carboxylate (9i). Ethyl (3a*R**,4*R**,8b*S**)-6-(2-bromobenzyl)-2-methyl-1,3-dioxo-1,2,3,3a,4,5,6,8b-octahydropyrrolo[3,4-*e*]indole-4-carboxylate (10i).** Following the method of preparation for **9a**/**10a**, a mixture of **8d** (0.451 g, 1.35 mmol) and **7b** (0.180 g, 1.62 mmol) in toluene (3.0 mL) produced a mixture of **9i**/**10i** (99:1) as a yellow oil. After purification, **9i** (0.580 g, 97%) was yielded as an orange resin. R*f* 0.24 (hexane/EtOAc, 7:3); IR (film): ῡ 2982, 2936, 1732, 1705, 1437, 1383, 1277, 1199, 1072, 1028, 752, 735, 713 cm^{-1}; ^{1}H NMR (500 MHz, $CDCl_3$) δ 1.30 (t, *J* = 7.3 Hz, 3H, $CO_2CH_2CH_3$), 2.63 (dd, *J* = 15.8, 12.0 Hz, 1H, H-5), 2.79 (dd, *J* = 15.8, 5.0 Hz, 1H, H-5), 2.90 (s, 3H, NCH_3), 2.91–2.95 (m, 1H, H-4), 3.91 (dd, *J* = 7.8, 4.5 Hz, 1H, H-3a), 4.12 (d, *J* = 7.8 Hz, 1H, H-8b), 4.21–4.32 (m, 2H, $CO_2CH_2CH_3$), 4.98 (d, *J* = 17.0 Hz, 1H, CH_2Ar), 5.02 (d, *J* = 17.0 Hz, 1H, CH_2Ar), 6.34 (d, *J* = 2.8 Hz, 1H, H-8), 6.39 (br d, *J* = 7.5 Hz, 1H, H-6'), 6.59 (d, *J* = 2.8 Hz, 1H, H-7), 7.13 (td, *J* = 7.5, 1.5 Hz, 1H, H-5'), 7.18 (br t, *J* = 7.5 Hz, 1H, H-4'), 7.56 (dd, *J* = 7.5, 0.8 Hz, 1H, H-3'); ^{13}C NMR (125 MHz, $CDCl_3$) δ 14.1 ($CO_2CH_2CH_3$), 20.0 (C-5), 24.7 (NCH_3), 38.9 (C-4), 41.0 (C-8b), 42.3 (C-3a), 50.2 (CH_2Ar), 61.2 ($CO_2CH_2CH_3$), 107.0 (C-8), 111.8 (C-8a), 121.9 (C-2'), 122.0 (C-7), 126.6 (C-5a), 127.5 (C-6'), 127.9 (C-4'), 129.1 (C-5'), 132.7 (C-3'), 136.9 (C-1''), 171.8

(*C*$O_2CH_2CH_3$), 176.9 (C-1 or C-3), 177.5 (C-3 or C-1); HRMS (EI): *m/z* [M^+] calcd for $C_{21}H_{21}N_2O_4Br$: 444.0685; found: 444.0685.

Ethyl (3a*R,4*S**,8b*S**)-6-(2-bromobenzyl)-1,3-dioxo-2-phenyl-1,2,3,3a,4,5,6,8b-octahydropyrrolo[3,4-*e*]indole-4-carboxylate (9j). Ethyl (3a*R**,4*R**,8b*S**)-6-(2-bromobenzyl)-1,3-dioxo-2-phenyl-1,2,3,3a,4,5,6,8b-octahydropyrrolo[3,4-*e*]indole-4-carboxylate (10j).** Following the method of preparation for **9a**/**10a**, a mixture of **8d** (0.300 g, 0.90 mmol) and **7c** (0.186 g, 1.08 mmol) in toluene (4.0 mL) gave a mixture of **9j**/**10j** (97:3) as a yellow oil. After purification, **9j** (0.429 g, 94%) was furnished as a white solid and **10j** (0.008 g, 2%) as a yellow oil; *Rf* 0.41 (hexane/EtOAc, 7:3). Data of **9j**: *Rf* 0.38 (hexane/EtOAc, 7:3); mp 178–179 °C; IR (KBr): ῡ 2940, 1713, 1596, 1496, 1444, 1384, 1324, 1290, 1230, 1191, 1161, 1129, 1030, 763, 712 cm^{-1}; ^{1}H NMR (500 MHz, $CDCl_3$) δ 1.28 (t, *J* = 7.0 Hz, 3H, $CO_2CH_2CH_3$), 2.73 (dd, *J* = 16.5, 11.5 Hz, 1H, H-5), 2.85 (dd, *J* = 16.5, 5.0 Hz, 1H, H-5), 3.01 (dt, *J* = 11.5, 5.0 Hz, 1H, H-4), 4.12 (dd, *J* = 8.0, 5.0 Hz, 1H, H-3a), 4.21–4.29 (m, 3H, H-8b, $CO_2CH_2CH_3$), 5.05 (s, 2H, CH_2Ar), 6.33 (dd, *J* = 6.5, 2.0 Hz, 1H, H-6”), 6.39 (d, *J* = 3.0 Hz, 1H, H-8), 6.61 (d, *J* = 3.0 Hz, 1H, H-7), 7.11–7.18 (m, 2H, H-4”, H-5”), 7.21 (d, *J* = 7.5 Hz, 2H, H-2’), 7.33 (t, *J* = 7.5 Hz, 1H, H-4’), 7.41 (t, *J* = 7.5 Hz, 2H, H-3’), 7.57 (dd, *J* = 7.0, 1.5 Hz, 1H, H-3”); signals attributed to the minor isomer **10j**: δ 1.12 (t, *J* = 7.5 Hz, $CO_2CH_2CH_3$), 2.66 (dd, *J* = 16.0, 6.0 Hz, H-5), 3.04 (dm, *J* = 16.0 Hz, H-5), 3.67 (dt, *J* = 6.0, 3.0 Hz, H-4), 3.93–4.00 (m, H-3a), 5.01 (d, *J* = 17.0 Hz, CH_2Ar), 6.36 (d, *J* = 2.5 Hz, H-8), 6.60 (d, *J* = 3.0 Hz, H-7); ^{13}C NMR (125 MHz, $CDCl_3$) δ 14.1 ($CO_2CH_2CH_3$), 20.0 (C-5), 38.8 (C-4), 41.4 (C-8b), 42.5 (C-3a), 50.3 (CH_2Ar), 61.3 ($CO_2CH_2CH_3$), 107.2 (C-8), 111.7 (C-8a), 121.9 (C-2”), 122.2 (C-7), 126.1 (C-2’), 126.8 (C-5a), 127.3 (C-6”), 127.9 (C-5”), 128.3 (C-4’), 128.9 (C-3’), 129.1 (C-4”), 131.8 (C-1’), 132.8 (C-3”), 137.0 (C-1”), 171.7 ($CO_2CH_2CH_3$), 175.8 (C-1), 176.3 (C-3); signals attributed to the minor isomer **10j**: δ 14.0 ($CO_2CH_2CH_3$), 20.6 (C-5), 38.1 (C-4), 39.9 (C-8b), 42.1 (C-3a), 50.4 (CH_2Ar), 107.1 (C-8), 111.4 (C-8a), 172.3 ($CO_2CH_2CH_3$), 176.7 (C-1), 176.8 (C-3); MS (70 eV): *m/z* 508 (M^++1, 26), 506 (M^+-1, 23), 434 (31), 427 (43), 381 (11), 353 (100), 287 (16), 232 (14), 206 (38), 171 (26); Anal calcd for $C_{26}H_{23}BrN_2O_4$: C, 61.55; H, 4.57; N, 5.52; found: C, 61.53; H, 4.56; N, 5.52.

Ethyl (3a*R,4*S**,8b*S**)-6-(3-methoxybenzyl)-1,3-dioxo-2-phenyl-1,2,3,3a,4,5,6,8b-octahydropyrrolo[3,4-*e*]indole-4-carboxylate (9k). Ethyl (3a*R**,4*R**,8b*S**)-6-(3-methoxybenzyl)-1,3-dioxo-2-phenyl-1,2,3,3a,4,5,6,8b-octahydropyrrolo[3,4-*e*]indole-4-carboxylate (10k).** Following the method of preparation for **9a**/**10a**, a mixture of **8e** (0.300 g, 1.05 mmol) and **7c** (0.219 g, 1.26 mmol) in toluene (5.0 mL) afforded a mixture of **9k**/**10k** (98:2) as a yellow oil. After purification, **9k** (0.463 g, 96%) was yielded as a yellow solid and **10k** (0.004 g, 1%) as a yellow oil; R_f 0.45 (hexane/EtOAc, 7:3). Data of **9k**: R*f* 0.31 (hexane/EtOAc, 7:3); mp 124–125 °C; IR (KBr): ῡ 2936, 1715, 1599, 1494, 1456, 1380, 1321, 1262, 1193, 1035, 760, 711 cm^{-1}; ^1H NMR (500 MHz, $CDCl_3$) δ 1.28 (t, *J* = 7.0 Hz, 3H, $CO_2CH_2CH_3$), 2.72 (dd, *J* = 16.0, 12.0 Hz, 1H, H-5), 2.87 (dd, *J* =16.0, 5.0 Hz, 1H, H-5), 2.98 (dt, *J* = 12.0, 5.0 Hz, 1H, H-4), 3.71 (s, 3H, C*H*3O), 4.09 (dd, *J* = 8.0, 5.0 Hz, 1H, H-3a), 4.20–4.30 (m, 3H, H-8b, $CO_2CH_2CH_3$), 4.98 (s, 2H, C*H*2Ar), 6.34 (d, *J* = 2.5 Hz, 1H, H-8), 6.44 (br s, 1H, H-2”), 6.54 (dd, *J* = 8.0, 1.0 Hz, 1H, H-6”), 6.63 (d, *J* = 2.5 Hz, 1H, H-7), 6.77 (dd, *J* = 8.0, 2.0 Hz, 1H, H-4”), 7.16–7.22 (m, 3H, H-2’, H-5”), 7.29–7.34 (m, 1H, H-4’), 7.35–7.41 (m, 2H, H-3’); signals attributed to the minor isomer **10k**: δ 1.12 (t, *J* = 7.0 Hz, $CO_2CH_2CH_3$), 2.63 (dd, *J* = 16.0, 6.0 Hz, H-5), 3.10 (d, *J* = 16.0 Hz, H-5), 3.66 (dt, *J* = 6.0, 2.5 Hz, H-4), 3.72 (s, C*H*3O), 3.93–4.09 (m, H-3a, $CO_2CH_2CH_3$), 4.12 (dq, *J* = 10.5, 7.0 Hz, $CO_2CH_2CH_3$), 4.96 (s, C*H*2Ar), 6.32 (d, *J* = 2.5 Hz, H-8), 6.45 (br s, H-2”), 6.56 (d, *J* = 7.5 Hz, H-6”), 6.62 (d, *J* = 3.0 Hz, H-7), 6.78 (dd, *J* = 8.5, 2.5 Hz, H-4”); ^{13}C NMR (125 MHz, $CDCl_3$) δ 14.1 ($CO_2CH_2CH_3$), 20.1 (C-5), 38.7 (C-4), 41.4 (C-8b), 42.5 (C-3a), 50.0 (*C*H2Ar), 55.1 (*C*H3O), 61.3 ($CO_2CH_2CH_3$), 106.7 (C-8), 111.5 (C-8a), 112.1 (C-2”), 112.7 (C-4”), 118.4 (C-6”), 122.2 (C-7), 126.1 (C-2’), 126.6 (C-5a), 128.2 (C-4’), 128.9 (C-3’), 129.8 (C-5”), 131.8 (C-1’), 139.2 (C-1”), 160.0 (C-3”), 171.8 ($CO_2CH_2CH_3$), 175.8 (C-1), 176.3 (C-3); MS (70 eV): *m/z* 459 (M^++1, 28), 458 (M^+, 86), 385 (51), 384 (100), 264 (15), 238 (25), 237 (48), 121 (54), 91 (24); Anal calcd for $C_{27}H_{26}N_2O_5$: C, 70.73; H, 5.72; N, 6.11; found: C, 70.72; H, 5.74; N, 6.09.

Methyl (3a*R,4*S**,8b*S**)-6-(2-bromo-4,5-dimethoxybenzyl)-1,3-dioxo-2-phenyl-1,2,3,3a,4,5,6,8b-octahydropyrrolo[3,4-*e*]indole-4-carboxylate (9l). Methyl**

(3a*R,4*R**,8b*S**)-6-(2-bromo-4,5-dimethoxybenzyl)-1,3-dioxo-2-phenyl-1,2,3,3a,4,5,6,8b-octahydropyrrolo[3,4-*e*]indole-4-carboxylate (10l).** Following the method of preparation for **9a**/**10a**, a mixture of **8f** (0.500 g, 1.32 mmol) and **7c** (0.274 g, 1.58 mmol) in toluene (6.0 mL) provided a mixture of **9l**/**10l** (93:7) as a yellow oil. After purification, **9l** (0.641 g, 88%) was produced as a yellow solid and **10l** (0.051 g, 7%) as a yellow oil. Data of **9l**: R*f* 0.18 (hexane/EtOAc, 7:3); mp 138–139 °C; IR (KBr): ῡ 2950, 1714, 1600, 1506, 1437, 1382, 1262, 1160, 1028, 804, 712 cm^{-1}; ^{1}H NMR (500 MHz, $CDCl_3$) δ 2.73 (br dd, *J* = 16.0, 11.5 Hz, 1H, H-5), 2.91 (dd, *J* = 16.0, 5.5 Hz, 1H, H-5), 3.01 (dt, *J* = 11.0, 5.5 Hz, 1H, H-4), 3.47 (s, 3H, CH_3O), 3.79 (s, 3H, CO_2CH_3), 3.85 (s, 3H, CH_3O), 4.11 (dd, *J* = 8.0, 4.5 Hz, 1H, H-3a), 4.25 (br d, *J* = 8.0 Hz, 1H, H-8b), 4.98 (s, 2H, CH_2Ar), 5.93 (s, 1H, H-6"), 6.38 (d, *J* = 3.0 Hz, 1H, H-8), 6.62 (d, *J* = 3.0 Hz, 1H, H-7), 7.02 (s, 1H, H-3"), 7.18–7.21 (m, 2H, H-2'), 7.31 (tt, *J* = 7.5, 2.0 Hz, 1H, H-4'), 7.37–7.41 (m, 2H, H-3'); ^{13}C NMR (125 MHz, $CDCl_3$) δ 20.1 (C-5), 38.5 (C-4), 41.4 (C-8b), 42.5 (C-3a), 50.0 (CH_2Ar), 52.4 (CO_2CH_3), 55.8 (CH_3O), 56.2 (CH_3O), 107.0 (C-8), 110.5 (C-6"), 111.7 (C-8a), 112.0 (C-2"), 115.6 (C-3"), 122.2 (C-7), 125.8 (C-2'), 126.6 (C-5a), 128.2 (C-4'), 128.6 (C-1"), 128.9 (C-3') , 131.7 (C-1'), 148.9 (C-4" or C-5"), 149.1 (C-5" or C-4"), 172.2 (CO_2CH_3), 175.8 (C-1 or C-3), 176.2 (C-3 or C-1); HRMS (EI): *m/z* [M^+] calcd for $C_{27}H_{25}N_2O_6Br$: 552.0896; found: 552.0885. Data of **10l**: R_f 0.25 (hexane/EtOAc, 7:3); IR (film): ῡ 2934, 1715, 1600, 1506, 1437, 1382, 1261, 1209, 1181, 1160, 1029, 804, 721 cm^{-1}; ^{1}H NMR (500 MHz, $CDCl_3$) δ 2.67 (dd, *J* = 16.0, 6.0 Hz, 1H, H-5), 3.04 (br d, *J* = 16.0 Hz, 1H, H-5), 3.54 (s, 3H, CH_3O), 3.56 (s, 3H, CO_2CH_3), 3.68–3.72 (m, 1H, H-4), 3.86 (s, 3H, CH_3O), 3.96 (ddd, *J* = 8.0, 3.0, 1.0 Hz, 1H, H-3a), 4.26 (d, *J* = 8.0 Hz, 1H, H-8b), 4.96 (s, 2H, CH_2Ar), 5.88 (s, 1H, H-6"), 6.36 (d, *J* = 3.0 Hz, 1H, H-8), 6.63 (d, *J* = 3.0 Hz, 1H, H-7), 7.02 (s, 1H, H-3"), 7.21–7.23 (m, 2H, H-2'), 7.34 (tt, *J* = 7.5, 2.0 Hz, 1H, H-4'), 7.42 (tm, *J* = 7.5 Hz, 2H, H-3'); ^{13}C NMR (125 MHz, $CDCl_3$) δ 20.4 (C-5), 37.8 (C-4), 39.8 (C-8b), 42.1 (C-3a), 50.0 (CH_2Ar), 52.4 (CO_2CH_3), 55.8 (CH_3O), 56.2 (CH_3O), 107.3 (C-8), 110.6 (C-6"), 111.4 (C-8a), 111.7 (C-2"), 115.4 (C-3"), 122.1 (C-7), 125.3 (C-5a), 126.1 (C-2'), 128.3 (C-4'), 128.9 (C-3'), 129.0 (C-1"), 131.8 (C-1'), 148.9 (C-4" or C-5"), 149.0 (C-5" or C-4"), 172.9 (CO_2CH_3), 176.5 (C-1 or C-3), 176.6 (C-3 or C-1); HRMS (EI): *m/z* [M^+] calcd for $C_{27}H_{25}BrN_2O_6$: 552.0896; found: 552.0878.

(3a*S,4*S**,8b*S**)-4-Acetyl-6-(2-bromobenzyl)-2-phenyl-4,5,6,8b-tetrahydropyrrolo[3,4-*e*]indole-1,3(2*H*,3a*H*)-dione (9m). (3a*S**,4*R**,8b*S**)-4-Acetyl-6-(2-bromobenzyl)-2-phenyl-4,5,6,8b-tetrahydropyrrolo[3,4-*e*]indole-1,3(2*H*,3a*H*)-dione (10m).** Following the method of preparation for **9a**/**10a**, a mixture of **8g** (0.440 g, 1.45 mmol) and **7c** (0.276 g, 1.59 mmol) in toluene (6.0 mL) furnished a mixture of **9m/10m** (91:9) as a yellow oil. After purification, **9m** (0.597 g, 86%) was afforded as a yellow solid and **10m** (0.055 g, 8%) as a yellow solid. Data of **9m**: R*f* 0.18 (hexane/EtOAc, 7:3); mp 165–166 °C; IR (KBr): ʋ 1712, 1495, 1440, 1382, 1192, 1027, 753, 692 cm^{-1}; ^{1}H NMR (500 MHz, $CDCl_3$): δ 2.32 (s, 3H, $COCH_3$), 2.68 (dd, *J* = 16.0, 11.0 Hz, 1H, H-5), 2.85 (dd, *J* = 16.0, 4.5 Hz, 1H, H-5), 2.90 (dt, *J* = 11.0, 4.5 Hz, 1H, H-4), 4.08 (dd, *J* = 8.0, 4.5 Hz, 1H, H-3a), 4.28 (d, *J* = 8.0 Hz, 1H, H-8b), 5.05 (s, 2H, CH_2Ar), 6.32 (dd, *J* = 7.5, 2.0 Hz, 2H, H-6”), 6.40 (d, *J* = 3.0 Hz, 1H, H-8), 6.63 (d, *J* = 2.5 Hz, 1H, H-7), 7.11–7.18 (m, 2H, H-4”, H-5”), 7.19–7.22 (m, 2H, H-2’), 7.34 (tm, *J* = 7.5 Hz, 1H, H-4’), 7.41 (tm, *J* = 7.5 Hz, 2H, H-3’), 7.57 (dd, *J* = 7.0, 2.0 Hz, 1H, H-3”); ^{13}C NMR (125 MHz, $CDCl_3$) δ 19.9 (C-5), 27.9 ($COCH_3$), 41.5 (C-8b), 42.6 (C-3a), 46.7 (C-4), 50.4 (CH_2Ar), 107.1 (C-8), 111.7 (C-8a), 121.9 (C-2”), 122.3 (C-7), 126.1 (C-2’), 126.6 (C-5a), 127.3 (C-6”), 127.9 (C-5”), 128.4 (C-4’), 129.0 (C-3’), 129.2 (C-4”), 131.7 (C-1’), 132.8 (C-3”), 137.0 (C-1”), 176.0 (C-3), 176.2 (C-1), 206.3 ($COCH_3$); MS (70 eV): *m/z* 478 (M^++1, 35), 476 (M^+-1, 42), 435 (62), 433 (64), 397 (38), 314 (47), 312 (43), 288 (36), 286 (40), 206 (30), 184 (24), 171 (100), 169 (94), 144 (16), 118 (19), 91 (31), 90 (30); HRMS (EI): *m/z* [M^+] calcd for $C_{25}H_{21}BrN_2O_3$: 476.0736; found: 476.0744; Anal calcd for $C_{25}H_{21}BrN_2O_3$: C, 62.90; H, 4.43; N, 5.87; found: C, 62.89; H, 4.41; N, 5.90. Data of **10m**: R*f* 0.30 (hexane/EtOAc, 7:3); mp 179–180 °C; IR (KBr): ʋ 1706, 1499, 1439, 1384, 1355, 1194, 1172, 1027, 762, 710 cm^{-1}; ^{1}H NMR (500 MHz, $CDCl_3$) δ 1.94 (s, 3H, $COCH_3$), 2.78 (dd, *J* = 16.0, 6.0 Hz, 1H, H-5), 2.86 (d, *J* = 16.0 Hz, 1H, H-5), 3.56–3.62 (m, 1H, H-4), 3.87 (dd, *J* = 8.0, 3.0 Hz, 1H, H-3a), 4.28 (d, *J* = 8.0 Hz, 1H, H-8b), 5.01 (d, *J* = 17.0 Hz, 1H, CH_2Ar), 5.09 (d, *J* = 17.0 Hz, 1H, CH_2Ar), 6.33 (br d, *J* = 7.5 Hz, 2H, H-6”), 6.36 (d, *J* = 3.0 Hz, 1H, H-8), 6.64 (d, *J* = 3.0 Hz, 1H, H-7), 7.14 (br t, *J* = 7.5 Hz, 1H, H-4”), 7.19 (br t, *J* = 7.5 Hz, 1H, H-5”), 7.22 (br d, *J* = 7.5 Hz, 2H, H-2’), 7.35 (br t, *J* = 7.5 Hz, 1H, H-4’), 7.42 (br t, *J* = 7.5 Hz, 2H, H-3’), 7.57 (d, *J* = 8.0 Hz, 1H, H-3”); ^{13}C NMR (125 MHz, $CDCl_3$) δ 20.4 (C-5), 27.3 ($COCH_3$), 39.8 (C-8b), 41.3 (C-3a), 45.9 (C-4), 50.4 (CH_2Ar), 107.5 (C-8), 112.3 (C-8a),

121.7 (C-2”), 122.4 (C-7), 124.3 (C-5a), 126.3 (C-2’), 127.5 (C-6”), 128.0 (C-5”), 128.4 (C-4’), 129.0 (C-3’), 129.3 (C-4”), 131.9 (C-1’), 132.7 (C-3”), 137.1 (C-1”), 176.8 (C-3), 177.4 (C-1), 207.0 (*C*OCH3); HRMS (EI): *m/z* [M+] calcd for $C_{25}H_{21}BrN_2O_3$: 476.0736; found: 476.0720; Anal calcd for $C_{25}H_{21}BrN_2O_3$: C, 62.90; H, 4.43; N, 5.87; found: C, 62.90; H, 4.44; N, 5.84.

(3a*S,4*S**,8b*S**)-4-Acetyl-6-(2-bromo-4,5-dimethoxybenzyl)-2-phenyl-4,5,6,8b-tetrahydropyrrolo[3,4-*e*]indole-1,3(2*H*,3a*H*)-dione (9n). (3a*S**,4*R**,8b*S**)-4-Acetyl-6-(2-bromo-4,5-dimethoxybenzyl)-2-phenyl-4,5,6,8b-tetrahydropyrrolo[3,4-*e*]indole-1,3(2*H*,3a*H*)-dione (10n).** Following the method of preparation for **9a**/**10a**, a mixture of **8h** (0.300 g, 0.82 mmol) and **7c** (0.170 g, 0.98 mmol) in toluene (5.0 mL) gave a mixture of **9n**/**10n** (90:10) as a yellow oil. After purification, **9n** (0.371 g, 84%) was provided as a yellow solid and **10n** (0.041 g, 9%) as a yellow solid. Data of **9n**: R*f* 0.18 (hexane/EtOAc, 7:3); mp 170–172 °C; IR (film): ῡ 2933, 1716, 1506, 1438, 1383, 1261, 1210, 1160, 1030, 734, 701 cm^{-1}; ^{1}H NMR (500 MHz, $CDCl_3$) δ 2.31 (s, 3H, COC*H*3), 2.68 (dd, *J* = 16.5, 12.3 Hz, 1H, H-5), 2.87–2.94 (m, 2H, H-4, H-5), 3.49 (s, 3H, C*H*3O), 3.85 (s, 3H, C*H*3O), 4.07 (dd, *J* = 8.0, 4.0 Hz, 1H, H-3a), 4.26 (d, *J* = 8.0 Hz, 1H, H-8b), 4.96 (d, *J* = 16.5 Hz, 1H, C*H*2Ar), 5.00 (d, *J* = 16.5 Hz, 1H, C*H*2Ar), 5.92 (s, 1H, H-6”), 6.35 (d, *J* = 2.7 Hz, 1H, H-8), 6.64 (d, *J* = 2.7 Hz, 1H, H-7), 7.02 (s, 1H, H-3”), 7.17–7.21 (m, 2H, H-2’), 7.30–7.34 (m, 1H, H-4’), 7.37–7.42 (m, 1H, H-3’); ^{13}C NMR (125 MHz, $CDCl_3$) δ 20.0 (C-5), 27.9 (CO*C*H3), 41.4 (C-8b), 42.6 (C-3a), 46.6 (C-4), 50.0 (*C*H2Ar), 55.8 (*C*H3O), 56.2 (*C*H3O), 106.9 (C-8), 110.4 (C-6”), 111.7 (C-8a), 111.9 (C-2”), 115.4 (C-3”), 122.2 (C-7), 125.8 (C-2’), 126.4 (C-5a), 128.3 (C-4’), 128.5 (C-1”), 128.9 (C-3’), 131.6 (C-1’), 148.9 (C-4” or C-5”), 149.0 (C-5” or C-4”), 176.0 (C-3), 176.2 (C-1), 206.3 (*C*OCH3); HRMS (EI): *m/z* [M+] calcd for $C_{27}H_{25}BrN_2O_5$: 536.0947; found: 536.0948. Data of **10n**: R*f* 0.28 (hexane/EtOAc, 7:3); IR (film): ῡ 2934, 1712, 1505, 1383, 1261, 1210, 1161, 1029, 721 cm^{-1}; ^{1}H NMR (500 MHz, $CDCl_3$) δ 1.93 (s, 3H, COC*H*3), 2.78 (dd, *J* = 16.5, 6.0 Hz, 1H, H-5), 2.88 (br d, *J* = 16.5 Hz, 1H, H-5), 3.53 (s, 3H, C*H*3O), 3.64 (dt, *J* = 6.3, 2.5 Hz, 1H, H-4), 3.82–3.86 (m, 1H, H-3a), 3.85 (s, 3H, C*H*3O), 4.29 (d, *J* = 8.0 Hz, 1H, H-8b), 4.93 (d, *J* = 16.9 Hz, 1H, C*H*2Ar), 5.02 (d, *J* = 16.9 Hz, 1H, C*H*2Ar), 5.78 (s, 1H, H-6’), 6.35 (d, *J* = 2.7 Hz, 1H, H-8), 6.64 (d, *J* = 2.7 Hz, 1H, H-7), 7.01 (s, 1H, H-3’), 7.20–7.23 (m, 1H, H-2”), 7.32–7.36

(m, 1H, H-4”), 7.39–7.44 (m, 1H, H-3”); ^{13}C NMR (125 MHz, $CDCl_3$) δ 20.4 (C-5), 27.3 (CO*C*H_3), 39.9 (C-8b), 41.3 (C-3a), 45.8 (C-4), 50.1 (*C*H_2Ar), 55.8 (*C*H_3O), 56.3 (*C*H_3O), 107.4 (C-8), 109.9 (C-6”), 111.4 (C-2”), 112.4 (C-8a), 115.3 (C-3”), 122.3 (C-7), 124.2 (C-5a), 126.1 (C-2’), 128.4 (C-4’), 128.9 (C-1”), 129.0 (C-3’), 131.8 (C-1’), 149.0 (C-4” or C-5”), 149.1 (C-5” or C-4”), 176.8 (C-3), 177.4 (C-1), 207.1 (*C*OCH_3); HRMS (EI): *m/z* [M^+] calcd for $C_{27}H_{25}BrN_2O_5$: 536.0947; found: 536.0953.

Methyl (3a*R,4*S**,8b*S**)-6-allyl-1,3-dioxo-2-phenyl-1,2,3,3a,4,5,6,8b-octahydropyrrolo[3,4-*e*]indole-4-carboxylate (9o). Methyl (3a*R**,4*R**,8b*S**)-6-allyl-1,3-dioxo-2-phenyl-1,2,3,3a,4,5,6,8b-octahydropyrrolo[3,4-*e*]indole-4-carboxylate (10o).** Following the method of preparation for **9a**/**10a**, a mixture of **8i** (0.500 g, 2.62 mmol) and **7c** (0.498 g, 2.88 mmol) in toluene (6.0 mL) afforded a mixture of **9o**/**10o** (98:2) as a yellow oil. After purification, **9o** (0.877 g, 92%) was afforded as an orange solid. R*f* 0.38 (hexane/EtOAc, 7:3); mp 214–215 °C; IR (KBr): ῡ 2915, 1736, 1713, 1494, 1429, 1379, 1330, 1259, 1194, 990, 932, 728, 714 cm^{-1}; ^{1}H NMR (300 MHz, $CDCl_3$) δ 2.76 (ddd, *J* = 15.9, 11.7, 1.2 Hz, 1H, H-5), 2.94 (dd, *J* = 15.9, 5.1 Hz, 1H, H-5), 3.01 (ddd, *J* = 11.7, 5.1, 4.2 Hz, 1H, H-4), 3.82 (s, 3H, CO_2C*H_3*), 4.10 (dd, *J* = 7.8, 4.2 Hz, 1H, H-3a), 4.23 (d, *J* = 7.8 Hz, 1H, H-8b), 4.40 (ddd, *J* = 4.8, 1.8, 1.5 Hz, 2H, H-1”), 4.86 (ddd, *J* = 17.1, 2.9, 1.8 Hz, 1H, H-3a”), 5.14 (ddd, *J* = 10.2, 2.9, 1.5 Hz, 1H, H-3b”), 5.90 (ddt, *J* = 17.1, 10.2, 4.8 Hz, 1H, H-2”), 6.31 (d, *J* = 2.9 Hz, 1H, H-8), 6.58 (d, *J* = 2.9 Hz, 1H, H-7), 7.17–7.25 (m, 2H, H-2’), 7.28–7.34 (m, 1H, H-4’), 7.35–7.45 (m, 2H, H-3’); signals attributed to the minor isomer **10o**: δ 6.28 (d, *J* = 3.0 Hz, H-8), 6.55 (d, *J* = 3.0 Hz, H-7); ^{13}C NMR (75.4 MHz, $CDCl_3$) δ 19.9 (C-5), 38.4 (C-4), 41.3 (C-8b), 42.5 (C-3a), 48.8 (C-1”), 52.3 (CO_2*C*H_3), 106.3 (C-8), 110.9 (C-8a), 116.8 (C-3”), 121.5 (C-7), 126.0 (C-2’), 126.2 (C-5a), 128.2 (C-4’), 128.8 (C-3’), 131.6 (C-1’), 133.8 (C-2”), 172.4 (*C*O_2CH_3), 175.9 (C-1 or C-3), 176.3 (C-3 or C-1); EM (70 eV): *m/z* 364 (M^+, 68), 305 (46), 304 (81), 184 (40), 158 (62), 157 (100), 156 (42), 130 (19), 117 (20); HRMS (EI): *m/z* [M^+] calcd for $C_{21}H_{20}N_2O_4$: 364.1423; found: 364.1421; Anal calcd for $C_{21}H_{20}N_2O_4$: C, 69.22; H, 5.53; N, 7.69; found: C, 69.24; H, 5.50; N, 7.69.

Methyl (3a*R,4*S**,8b*S**)-1,3-dioxo-2-phenyl-6-(prop-2-yn-1-yl)-1,2,3,3a,4,5,6,8b-octahydropyrrolo[3,4-*e*]indole-4-carboxylate (9p). Methyl (3a*R**,4*R**,8b*S**)-1,3-dioxo-2-phenyl-6-(prop-2-yn-1-yl)-1,2,3,3a,4,5,6,8b-octahydropyrrolo[3,4-*e*]indole-4-carboxylate (10p).** Following the method of preparation for **9a**/**10a**, a mixture of **8j** (0.600 g, 3.17 mmol) and **7c** (0.604 g, 3.49 mmol) in toluene (6.0 mL) generated a mixture of **9p**/**10p** (99:1) as a yellow oil. After purification, **9p** (1.096 g, 95%) was isolated as an orange solid. R*f* 0.38 (hexane/EtOAc, 7:3 x 2); mp 187–188 °C; IR (KBr): ῡ 3291, 2912, 2126, 1710, 1596, 1495, 1454, 1434, 1374, 1325, 1269, 1227, 1204, 1159, 1126, 1086, 1026, 912, 884, 813, 762, 715, 643 cm^{-1}; ^1H NMR (300 MHz, $CDCl_3$) δ 2.40 (t, *J* = 2.6 Hz, 1H, H-3”), 2.84 (dd, *J* = 17.4, 13.2 Hz, 1H, H-5), 3.00–3.11 (m, 2H, H-4, H-5), 3.83 (s, 3H, CO_2CH_3), 4.11 (dd, *J* = 7.8, 4.0 Hz, 1H, H-3a), 4.20 (d, *J* = 7.8 Hz, 1H, H-8b), 4.56 (d, *J* = 2.6 Hz, 2H, H-1”), 6.32 (d, *J* = 3.0 Hz, 1H, H-8), 6.71 (d, *J* = 3.0 Hz, 1H, H-7), 7.18–7.24 (m, 2H, H-2’), 7.28–7.34 (m, 1H, H-4’), 7.35–7.43 (m, 2H, H-3’); ^{13}C NMR (75.4 MHz, $CDCl_3$) δ 19.9 (C-5), 35.9 (C-1”), 38.3 (C-4), 41.2 (C-8b), 42.4 (C-3a), 52.4 (CO_2CH_3), 73.8 (C-3”), 77.5 (C-2”), 106.8 (C-8), 111.7 (C-8a), 121.2 (C-7), 126.0 (C-2’), 126.1 (C-5a), 128.2 (C-4’), 128.8 (C-3’), 131.5 (C-1’), 172.3 (CO_2CH_3), 175.8 (C-1 or C-3), 176.1 (C-3 or C-1); HRMS (EI): *m/z* [M^+] calcd for $C_{21}H_{18}N_2O_4$: 362.1267; found: 361.1253; Anal calcd for $C_{21}H_{18}N_2O_4$: C, 69.60; H, 5.01; N, 7.73; found: C, 69.61; H, 4.96; N, 7.73.

Methyl (*E*)-3-(5*H*-pyrrolo[2,1-*a*]isoindol-3-yl)acrylate (18a). In a threaded ACE glass pressure tube equipped with a sealed Teflon screw cap and a magnetic stirring bar, a mixture of **8c** (0.100 g, 0.31 mmol) and $Pd(PPh_3)_4$ (0.072 g, 0.062 mmol) and KOAc (0.061 g, 0.63 mmol) in dry DMA (1.0 mL) was heated at 100 °C for 12 h. Water (50 mL) was added and the resulting solution extracted with EtOAc (2 × 50 mL). The organic layer was dried (Na_2SO_4) and the solvent removed under vacuum. The residue was purified by column chromatography over silica gel (20 g/g crude, hexane/EtOAc, 95:5) to give **18a** (0.065 g, 88%) as a yellow solid. R*f* 0.80 (hexano/EtOAc, 7:3); m.p. 140–141 °C; IR (KBr): ῡ 2948, 1692, 1618, 1474, 1436, 1261, 1171, 1033, 968, 858, 838, 747 cm^{-1}; ^1H NMR (300 MHz, $CDCl_3$) δ 3.80 (s, 3H, CO_2CH_3), 4.97 (s, 2H, H-5’), 6.00 (d, *J* = 15.9 Hz, 1H, H-2), 6.41 (d, *J* = 3.9 Hz, 1H, H-1’), 6.69 (d, *J* = 3.9 Hz, 1H, H-2’), 7.25 (ddd, *J* = 7.5, 6.3, 1.2 Hz, 1H, H-7’), 7.37 (tm, *J* = 7.5 Hz, 1H, H-8’), 7.43 (dt, *J* = 7.5, 0.9 Hz, 1H, H-6’),

7.56 (br d, *J* = 7.5 Hz, 1H, H-9'), 7.64 (d, *J* = 15.9 Hz, 1H, H-3); ^{13}C NMR (75.4 MHz, $CDCl_3$) δ 51.1 (C-5'), 51.5 ($CO_2\mathit{C}H_3$), 101.0 (C-1'), 110.0 (C-2), 119.5 (C-9'), 120.3 (C-2'), 123.1 (C-6'), 126.1 (C-8'), 126.2 (C-3'), 128.1 (C-7'), 132.1 (C-5a'), 133.3 (C-3), 140.7 (C-9a'), 143.2 (C-9b'), 168.2 ($\mathit{C}O_2CH_3$); MS (70 eV): *m/z* 240 (M^++1, 20), 239 (M^+, 100), 224 (14), 208 (30), 207 (60), 196 (13), 180 (69), 179 (36), 152 (25); HRMS (EI): *m/z* [M^+] calcd for $C_{25}H_{13}NO_2$: 239.0946; found: 239.0958.

Ethyl (*E*)-3-(5*H*-pyrrolo[2,1-*a*]isoindol-3-yl)acrylate (18b). Following the method of preparation for **18a**, a mixture of **8d** (0.200 g, 0.60 mmol), KOAc (0.118 g, 1.2 mmol) and $Pd(PPh_3)_4$ (0.139 g, 0.12 mmol) in dry DMA (1.0 mL), after stirring at 110 °C for 12 h, produced **18b** (0.127 g, 83%) was obtained as a reddish solid. R*f* 0.79 (hexane/EtOAc, 7:3); mp 98–100 °C; IR (film): ῡ 2980, 1702, 1618, 1447, 1315, 1289, 1260, 1208, 1180, 1033, 969, 750 cm^{-1}; ^{1}H NMR (600 MHz, $CDCl_3$) δ 1.34 (t, *J* = 7.0 Hz, 3H, $CO_2CH_2C\mathit{H}_3$), 4.26 (q, *J* = 7.0 Hz, 2H, $CO_2C\mathit{H}_2CH_3$), 4.95 (s, 2H, H-5'), 5.99 (d, *J* = 16.0 Hz, 1H, H-2), 6.40 (d, *J* = 3.9 Hz, 1H, H-1'), 6.68 (d, *J* = 3.9 Hz, 1H, H-2'), 7.23 (td, *J* = 7.4, 1.2 Hz, 1H, H-7'), 7.35 (br t, *J* = 7.4 Hz, 1H, H-8'), 7.41 (br d, *J* = 7.4 Hz, 1H, H-6'), 7.54 (br d, *J* = 7.4 Hz, 1H, H-9'), 7.62 (d, *J* = 16.0 Hz, 1H, H-3); ^{13}C NMR (150 MHz, $CDCl_3$) δ 14.4 ($CO_2CH_2\mathit{C}H_3$), 51.1 (C-5'), 60.2 ($CO_2\mathit{C}H_2CH_3$), 101.0 (C-1'), 110.6 (C-2), 119.6 (C-9'), 120.2 (C-2'), 123.1 (C-6'), 126.1 (C-8'), 126.4 (C-3'), 128.2 (C-7'), 132.2 (C-9a'), 133.1 (C-3), 140.7 (C-5a'), 143.2 (C-9b'), 167.8 ($\mathit{C}O_2CH_2CH_3$); HRMS (EI): m/z [M^+] calcd for $C_{16}H_{15}NO_2$: 253.1103; found: 253.1109.

(*E*)-4-(5*H*-Pyrrolo[2,1-*a*]isoindol-3-yl)but-3-en-2-one (18c). Following the method of preparation for **18a**, a mixture of **8g** (0.100 g, 0.33 mmol), AcOK (0.143 g, 1.46 mmol) and $Pd(PPh_3)_4$ (0.081 g, 0.07 mmol) in anhydrous MeCN (1.0 mL), after stirring at 100 °C for 40 h, furnished **18c** (0.054 g, 74%) as a yellow solid. R*f* 0.43 (hexane/EtOAc, 8:2); mp 100–101 °C; IR (KBr): ῡ 1607, 1476, 1446, 1359, 1260, 1172, 1040, 963, 743, 525 cm^{-1}; ^{1}H NMR (300 MHz, $CDCl_3$) δ 2.36 (s, 3H, $C\mathit{H}_3CO$), 5.02 (s, 2H, H-5'), 6.34 (d, *J* = 16.2 Hz, 1H, H-3), 6.46 (d, *J* = 3.9 Hz, 1H, H-1'), 6.75 (d, *J* = 3.9 Hz, 1H, H-2'), 7.28 (td, *J* = 7.5, 1.2 Hz, 1H, H-7'), 7.39 (td, *J* = 7.5, 0.9 Hz, 1H, H-8'), 7.46 (d, *J* = 7.5 Hz, 1H, H-6'), 7.48 (d, *J* = 16.2 Hz, 1H, H-4), 7.59 (d, *J* = 7.5 Hz, 1H, H-9'); ^{13}C NMR (75.4 MHz,

CDCl3) δ 27.2 (*C*H_3CO), 51.4 (C-5'), 101.6 (C-1'), 119.7 (C-9'), 120.2 (C-3), 121.4 (C-2'), 123.2 (C-6'), 126.2 (C-3'), 126.4 (C-7'), 128.3 (C-8'), 132.0 (C-9a'), 132.2 (C-4), 140.8 (C-5a'), 144.0 (C-9b'), 198.1 (*C*OCH_3); HRMS (EI): m/z [M^+] calcd for $C_{15}H_{13}NO$: 223.0997; found: 223.0998.

Dimethyl 2-((5*H*-pyrrolo[2,1-*a*]isoindol-3-yl)methylene)malonate (18d). Following the method of preparation for **18a**, a mixture of **8k** (0.100 g, 0.26 mmol), KOAc (0.051 g, 0.52 mmol) and Pd(PPh_3)$_4$ (0.035 g, 0.03 mmol) in anhydrous MeCN (3.0 mL), after stirring at 140 °C for 40 h, gave **18d** (0.056 g, 72%) as a yellow solid. R*f* 0.29 (hexane/EtOAc, 8:2); mp 136–137 °C; IR (film): ῡ 2951, 2922, 1728, 1605, 1432, 1408, 1243, 1212, 1156, 1067, 751 cm^{-1}; ^{1}H NMR (300 MHz, $CDCl_3$) δ 3.83 (s, 3H, CO_2CH_3), 3.93 (s, 3H, CO_2CH_3), 5.01 (s, 2H, H-5"), 6.42 (dd, *J* = 4.2, 0.3 Hz, 1H, H-1"), 6.77 (d, *J* = 4.2 Hz, 1H, H-2"), 7.26 (td, *J* = 7.5, 1.2 Hz, 1H, H-7"), 7.37 (td, *J* = 7.5, 1.2 Hz, 1H, H-8"), 7.42 (br d, *J* = 7.5 Hz, 1H, H-6"), 7.54 (br d, *J* = 7.5 Hz, 1H, H-9"), 7.64 (br s, 1H, H-1'); ^{13}C NMR (75.4 MHz, $CDCl_3$) δ 49.5 (C-5"), 52.4 (CO_2CH_3), 52.7 (CO_2CH_3), 102.6 (C-1"), 116.7 (C-2), 118.9 (C-2"), 119.8 (C-9"), 123.3 (C-6"), 123.7 (C-3"), 126.5 (C-7"), 128.3 (C-8"), 129.9 (C-1'), 132.5 (C-9a"), 140.3 (C-5a"), 143.2 (C-9b"), 165.3 (CO_2CH_3), 167.7 (CO_2CH_3); HRMS (EI): m/z [M^+] calcd for $C_{17}H_{15}NO_4$: 297.1001; found: 297.1010.

Dimethyl 2-((7,8-dimethoxy-5*H*-pyrrolo[2,1-*a*]isoindol-3-yl)methylene)malonate (18e). Following the method of preparation for **18a**, a mixture of **8l** (0.100 g, 0.23 mmol), KOAc (0.045 g, 0.46 mmol) and Pd(PPh_3)$_4$ (0.023 g, 0.02 mmol) in anhydrous MeCN (3.0 mL), after stirring at 140 °C for 40 h, led to the formation of **18e** (0.063 g, 78%) as a yellow solid. R*f* 0.12 (hexane/EtOAc, 7:3); mp 162–163 °C; IR (film): ῡ 2924, 1692, 1578, 1417, 1270, 1154, 1076, 783 cm^{-1}; ^{1}H NMR (300 MHz, $CDCl_3$) δ 3.83 (s, 3H, CO_2CH_3), 3.93 (s, 6H, CO_2CH_3, CH_3O), 3.94 (s, 3H, CH_3O), 4.90 (s, 2H, H-5"), 6.30 (d, *J* = 2.6 Hz, 1H, H-1"), 6.75 (d, *J* = 2.6 Hz, 1H, H-2"), 6.97 (s, 1H, H-9"), 7.05 (s, 1H, H-9"), 7.61 (s, 1H, H-1'); ^{13}C NMR (74.5 MHz, $CDCl_3$) δ 49.4 (C-5"), 52.3 (CO_2CH_3), 52.6 (CO_2CH_3), 56.1 (CH_3O), 56.2 (CH_3O), 102.9 (C-6"), 106.7 (C-9"), 115.8 (C-2), 119.0 (C-2"), 123.8 (C-3"), 125.3 (C-5a"), 129.9 (C-1'), 132.9 (C-9b"), 132.9 (C-9b"), 143.9 (C-9a"), 148.5 (C-7"),

149.5 (C-8”), 165.3 (CO_2CH_3), 167.7 (CO_2CH_3); HRMS (EI): m/z [M^+] calcd for $C_{19}H_{19}NO_6$: 357.1212; found: 357.1215.

Ethyl (3a*R,4*S**,12b*S**)-1,3-dioxo-2-phenyl-1,2,3,3a,4,5,7,12b-octahydroisoindolo[2,1-*a*]pyrrolo[3,4-*e*]indole-4-carboxylate (11a).** Following the method of preparation for **18a**, a mixture of **9j** (0.200 g, 0.39 mmol), KOAc (0.076 g, 0.78 mmol) and $Pd(PPh_3)_4$ (0.090 g, 0.078 mmol) in anhydrous MeCN (1.0 mL), after stirring at 100 °C for 48 h, afforded **11a** (0.126 g, 75%) as a reddish solid. R*f* 0.28 (hexane/EtOAc, 7:3); mp 236–238 °C; IR (film): ʋ 2929, 1714, 1499, 1373, 1192, 1128, 1044, 751 cm^{-1}; ^{1}H NMR (600 MHz, $CDCl_3$) δ 1.34 (t, *J* = 7.2 Hz, 3H, $CO_2CH_2CH_3$), 2.92 (dd, *J* = 15.8, 12.0 Hz, 1H, H-5), 3.01 (dd, *J* = 15.8, 4.8 Hz, 1H, H-5), 3.08 (dt, *J* = 12.0, 4.8 Hz, 1H, H-4), 4.17 (dd, *J* = 7.8, 4.2 Hz, 1H, H-3a), 4.30 (d, *J* = 7.8 Hz, 1H, H-12b), 4.32 (q, *J* = 7.2 Hz, 2H, $CO_2CH_2CH_3$), 4.75 (d, *J* = 16.2 Hz, 1H, H-7), 4.79 (d, *J* = 16.2 Hz, 1H, H-7), 6.46 (s, 1H, H-12), 7.17 (ddd, *J* = 7.8, 7.2, 0.6 Hz, 1H, H-10), 7.21–7.24 (m, 2H, H-2’), 7.28–7.31 (m, 1H, H-4’), 7.32 (br t, *J* = 7.2 Hz, 1H, H-9), 7.36–7.39 (m, 3H, H-8, H-3’), 7.48 (br d, *J* = 7.8 Hz, 1H, H-11); ^{13}C NMR (150 MHz, $CDCl_3$) δ 14.1 ($CO_2CH_2CH_3$), 20.6 (C-5), 38.4 (C-4), 41.8 (C-12b), 42.6 (C-3a), 48.5 (C-7), 61.4 ($CO_2CH_2CH_3$), 96.8 (C-12), 114.5 (C-12a), 118.9 (C-11), 123.1 (C-8), 123.9 (C-5a), 125.1 (C-9), 126.1 (C-2’), 128.1 (C-10), 128.2 (C-4’), 128.9 (C-3’), 131.7 (C-1’), 133.3 (C-11a), 138.1 (C-11b), 139.5 (C-7a), 171.8 ($CO_2CH_2CH_3$), 175.9 (C-3), 176.4 (C-1); HRMS (EI): m/z [M^+] calcd for $C_{26}H_{22}N_2O_4$: 426.1580; found: 426.1584.

Methyl (3a*R,4*S**,12b*S**)-1,3-dioxo-2-phenyl-1,2,3,3a,4,5,7,12b-octahydroisoindolo[2,1-*a*]pyrrolo[3,4-*e*]indole-4-carboxylate (11b).** Method A. Following the method of preparation for **18a**, a mixture of **9h** (0.200 g, 0.41 mmol), KOAc (0.080 g, 0.82 mmol) and $Pd(PPh_3)_4$ (0.095 g, 0.082 mmol) in anhydrous MeCN (2.0 mL), after stirring at 100 °C for 48 h, provided **11b** (0.128 g, 77%) as a reddish solid. R*f* 0.38 (hexane/EtOAc, 7:3 x 2); mp 298–299 °C. Method B. Following the method of preparation for **9a**/**10a**, a mixture of **18a** (0.060 g, 0.25 mmol) and **7c** (0.041 g, 0.30 mmol) in toluene (2.0 mL), after stirring at 150 °C for 10 d, generated a mixture of **11b**/**20** (91:9) as an amber oil. After purification, **11b** (0.093 g, 90%) was obtained as a reddish solid; R*f* 0.38 (hexane/EtOAc, 7:3 x 2); mp 298–299 °C; IR (KBr): ʋ 2917, 1768, 1710, 1616, 1499,

1452, 1380, 1273, 1209, 1174, 1131, 757, 693 cm^{-1}; ^{1}H NMR (500 MHz, $CDCl_3$) δ 2.93 (dd, *J* = 16.0, 12.0 Hz, 1H, H-5), 3.01 (dd, *J* = 16.0, 5.0 Hz, 1H, H-5), 3.09 (dt, *J* = 12.0, 5.0 Hz, 1H, H-4), 3.85 ($CO_2C\mathit{H}_3$), 4.15 (dd, *J* = 8.0, 4.0 Hz, 1H, H-3a), 4.29 (d, *J* = 8.0 Hz, 1H, H-12b), 4.74 (d, J = 16.5 Hz, 1H, H-7), 4.78 (d, *J* = 16.5 Hz, 1H, H-7), 6.46 (s, 1H, H-12), 7.16 (br t, *J* = 7.5 Hz, 1H, H-9), 7.22–7.24 (m, 2H, H-2'), 7.26–7.34 (m, 2H, H-10, H-4'), 7.35–7.39 (m, 3H, H-8, H-3'), 7.48 (br d, *J* = 8.0 Hz, 1H, H-11); signals attributed to the minor isomer **20**: δ 2.85 (dd, *J* = 16.2, 5.4 Hz, H-5), 4.03 (dd, *J* = 7.8, 3.0 Hz, H-3a), 6.44 (s, H-12); ^{13}C NMR (125 MHz, $CDCl_3$) δ 20.6 (C-5), 38.3 (C-4), 41.8 (C-12b), 42.7 (C-3a), 48.5 (C-7), 52.4 ($CO_2\mathit{C}H_3$), 96.9 (C-12), 114.5 (C-12a), 118.9 (C-11), 123.1 (C-8), 123.8 (C-5a), 125.1 (C-9), 126.1 (C-2'), 128.1 (C-10), 128.2 (C-4'), 128.9 (C-3'), 131.7 (C-1'), 133.3 (C-11a), 138.1 (C-11b), 139.5 (C-7a), 172.3 ($\mathit{C}O_2CH_3$), 175.9 (C-3), 176.3 (C-1); HRMS (EI): m/z [M^+] calcd for $C_{25}H_{20}N_2O_4$: 412.1423; found: 412.1416.

Ethyl (3a*R,4*S**,12b*S**)-2-methyl-1,3-dioxo-1,2,3,3a,4,5,7,12b-octahydroisoindolo[2,1-*a*]pyrrolo[3,4-*e*]indole-4-carboxylate (11c).** Following the method of preparation for **18a**, a mixture of **9i** (0.100 g, 0.22 mmol), KOAc (0.043 g, 0.44 mmol) and $Pd(PPh_3)_4$ (0.046 g, 0.04 mmol) in anhydrous MeCN (1.0 mL), after stirring at 100 °C for 48 h, produced **11c** (0.065 g, 81%) as a reddish solid. R*f* 0.26 (hexane/EtOAc, 7:3); mp 187 °C–decomp; IR (film): ῡ 2928, 1731, 1702, 1435, 1380, 1275, 1096, 1051, 1029, 948, 758 cm^{-1}; ^{1}H NMR (600 MHz, $CDCl_3$) δ 1.35 (t, *J* = 7.2 Hz, 3H, $CO_2CH_2C\mathit{H}_3$), 2.80 (dd, *J* = 15.5, 12.3 Hz, 1H, H-5), 2.90 (s, 3H, $NC\mathit{H}_3$), 2.94–3.02 (m, 2H, H-4, H-5), 3.96 (dd, *J* = 7.8, 4.2 Hz, 1H, H-3a), 4.16 (d, *J* = 7.8 Hz, 1H, H-12b), 4.30–4.36 (m, 2H, $CO_2C\mathit{H}_2CH_3$), 4.72 (d, *J* = 16.5 Hz, 1H, H-7), 4.77 (d, *J* = 16.5 Hz, 1H, H-7), 6.41 (s, 1H, H-12), 7.15 (t, *J* = 7.8 Hz, 1H, H-9), 7.31 (t, *J* = 7.8 Hz, 1H, H-10), 7.35 (d, *J* = 7.8 Hz, 1H, H-8), 7.46 (d, *J* = 7.8 Hz, 1H, H-11); ^{13}C NMR (150 MHz, $CDCl_3$) δ 14.1 ($CO_2CH_2\mathit{C}H_3$), 20.6 (C-5), 24.7 ($N\mathit{C}H_3$), 38.5 (C-4), 41.5 (C-12b), 42.4 (C-3a), 48.4 (C-7), 61.4 ($CO_2\mathit{C}H_2CH_3$), 96.7 (C-12), 114.8 (C-12a), 118.9 (C-11), 123.1 (C-8), 123.9 (C-5a), 125.1 (C-9), 128.1 (C-10), 133.3 (C-11a), 138.0 (C-11b), 139.5 (C-7a), 171.9 ($\mathit{C}O_2CH_2CH_3$), 176.9 (C-3), 177.64 (C-1); HRMS (EI): m/z [M^+] calcd for $C_{21}H_{20}N_2O_4$: 364.1423; found: 364.1421.

(3a*S,4*S**,12b*S**)-4-Acetyl-2-phenyl-3a,5,7,12b-tetrahydroisoindolo[2,1-*a*]pyrrolo[3,4-*e*]indole-1,3(2*H*,4*H*)-dione (11d).** Following the method of preparation for **18a**, a mixture of **9m** (0.150 g, 0.31 mmol), KOAc (0.061 g, 0.62 mmol) and $Pd(PPh_3)_4$ (0.072 g, 0.062 mmol) in anhydrous MeCN (1.0 mL), after stirring at 100 °C for 48 h, delivered **11d** (0.059 g, 48%) as a reddish solid. R*f* 0.28 (hexane/EtOAc, 7:3); mp 253–254 °C; IR (film): ῡ 2918, 1702, 1386, 1163, 1014, 750 cm^{-1}; ^{1}H NMR (600 MHz, $CDCl_3$) δ 2.25 (s, 3H, $COCH_3$), 2.98 (dd, *J* = 16.6, 6.0 Hz, 1H, H-5), 3.12 (br d, *J* = 16.6 Hz, 1H, H-5), 3.68–3.71 (m, 1H, H-4), 3.98 (dd, *J* = 8.0, 3.0 Hz, 1H, H-3a), 4.32 (d, *J* = 8.0 Hz, 1H, H-12b), 4.77 (d, *J* = 16.2 Hz, 1H, H-7), 4.81 (d, *J* = 16.2 Hz, 1H, H-7), 6.45 (s, 1H, H-12), 7.32 (t, *J* = 6.0 Hz, 1H, H-8), 7.25 (d, *J* = 7.6 Hz, 1H, H-2'), 7.32 (t, *J* = 7.5 Hz, 1H, H-10), 7.34–7.38 (m, 2H, H-9, H-4'), 7.42 (t, *J* = 7.6 Hz, 1H, H-3'), 7.47 (d, *J* = 7.6 Hz, 1H, H-11); ^{13}C NMR (150 MHz, $CDCl_3$) δ 21.1 (C-5), 27.6 ($COCH_3$), 40.1 (C-12b), 41.6 (C-3a), 45.8 (C-4), 48.4 (C-7), 97.4 (C-12), 114.8 (C-12a), 119.0 (C-11), 121.8 (C-5a), 123.1 (C-8), 125.1 (C-9), 126.3 (C-2'), 128.2 (C-10), 128.4 (C-4'), 129.0 (C-3'), 131.8 (C-1'), 133.4 (C-11a), 138.1 (C-11b), 139.3 (C-7a), 176.8 (C-1), 177.5 (C-3), 207.0 ($COCH_3$); HRMS (EI): m/z [M^+] calcd for $C_{25}H_{20}N_2O_3$: 396.1474; found: 396.1479.

Methyl (3a*R,4*S**,12b*S**)-2-methyl-1,3-dioxo-1,2,3,3a,4,5,7,12b-octahydroisoindolo[2,1-*a*]pyrrolo[3,4-*e*]indole-4-carboxylate (11e).** Following the method of preparation for **18a**, a mixture of **9g** (0.200 g, 0.47 mmol), KOAc (0.091 g, 0.93 mmol) and $Pd(PPh_3)_4$ (0.107 g, 0.093 mmol) in anhydrous MeCN (2.0 mL), after stirring at 100 °C for 48 h, furnished **11e** (0.140 g, 86%) as a reddish solid. R*f* 0.18 (hexane/EtOAc, 1:1); mp 219–221 °C; IR (film): ῡ 2918, 1740, 1702, 1622, 1386, 1163, 1014, 951, 750 cm^{-1}; ^{1}H NMR (500 MHz, $CDCl_3$) δ 2.74–2.85 (m, 1H, H-5), 2.89 (s, 3H, NCH_3), 2.93–3.04 (m, 2H, H-4, H-5), 3.86 (s, 3H, CO_2CH_3), 3.95 (dd, *J* = 7.7, 4.5 Hz, 1H, H-3a), 4.16 (d, *J* = 7.7 Hz, 1H, H-12b), 4.71 (d, *J* = 16.5 Hz, 1H, H-7), 4.76 (d, *J* = 16.5 Hz, 1H, H-7), 6.41 (s, 1H, H-12), 7.15 (t, *J* = 7.5 Hz, 1H, H-9), 7.31 (t, *J* = 7.5 Hz, 1H, H-10), 7.35 (d, *J* = 7.5 Hz, 1H, H-8), 7.46 (d, *J* = 7.5 Hz, 1H, H-11); ^{13}C NMR (125 MHz, $CDCl_3$) δ 20.6 (C-5), 24.7 (NCH_3), 38.4 (C-4), 41.5 (C-12b), 42.4 (C-3a), 48.4 (C-7), 52.4 (CO_2CH_3), 96.7 (C-12), 114.8 (C-12a), 118.9 (C-11), 123.1 (C-8), 123.7 (C-5a), 125.1 (C-9), 128.1 (C-10), 133.3

(C-11a), 138.0 (C-11b), 139.5 (C-7a), 172.4 (CO_2CH_3), 176.9 (C-3), 177.5 (C-1); HRMS (EI): m/z [M^+] calcd for $C_{20}H_{18}N_2O_4$: 350.1267; found: 350.1267.

Ethyl 6-benzyl-1,3-dioxo-2-phenyl-1,2,3,6-tetrahydropyrrolo[3,4-*e*]indole-4-carboxylate (21a). To a solution of **9e** (0.200 g, 0.47 mmol) in CH_2Cl_2 (20 mL), DDQ (0.268 g, 1.18 mmol) was added. After stirring at rt for 48 h, the reaction mixture was filtered over celite and the solvent removed under vacuum. The residue was purified by column chromatography over silica gel (20 g/g crude, hexane/EtOAc, 7:3), resulting in **21a** (0.175 g, 88%) as a yellow solid. R*f* 0.63 (hexano/AcOEt, 7:3 x 2); mp 165–166 °C; IR (KBr): ῡ 1770, 1710, 1499, 1392, 1369, 1277, 1205, 1114, 1025, 749, 696 cm^{-1}; ^1H NMR (500 MHz, $CDCl_3$) δ 1.42 (t, *J* = 7.5 Hz, 3H, $CO_2CH_2CH_3$), 4.46 (q, *J* = 7.5 Hz, 2H, $CO_2CH_2CH_3$), 5.42 (s, 2H, CH_2Ph), 7.08–7.12 (m, 2H, H-2”), 7.17 (dd, *J* = 3.0, 1.0 Hz, 1H, H-8), 7.29–7.35 (m, 3H, H-3”, H-4”), 7.35–7.40 (m, 1H, H-4’), 7.46–7.51 (m, 5H, H-7, H-2’, H-3’), 7.94 (d, *J* = 1.0 Hz, 1H, H-5); ^{13}C NMR (125 MHz, $CDCl_3$) δ 14.1 ($CO_2CH_2CH_3$), 50.6 (CH_2Ph), 62.0 ($CO_2CH_2CH_3$), 102.2 (C-8), 115.7 (C-5), 121.9 (C-3a), 123.2 (C-4), 124.9 (C-8b), 125.2 (C-8a), 126.7 (C-2’), 126.8 (C-2”), 127.7 (C-4’), 128.3 (C-4”), 128.9 (C-3’), 129.1 (C-3”), 132.0 (C-1’), 135.6 (C-1”), 135.7 (C-7), 139.5 (C-5a), 166.2 (C-1 or C-3), 166.7 ($CO_2CH_2CH_3$), 167.3 (C-3 or C-1); MS (70 eV): m/z 425 (M^++1, 29), 242 (M^+, 100), 379 (11), 352 (34), 351 (31), 336 (14), 308 (8), 266 (6), 205 (6), 91 (80); HRMS (EI): m/z [M^+] calcd for $C_{26}H_{20}N_2O_4$: 424.1423; found: 424.1434.

6-Benzyl-1,3-dioxo-2-phenyl-1,2,3,6-tetrahydropyrrolo[3,4-*e*]indole-4-carbonitrile (21b). Following the method of preparation for **21a**, a mixture of **9f** (0.200 g, 0.52 mmol) and DDQ (0.293 g, 1.3 mmol) in CH_2Cl_2 (20 mL) gave **21b** (0.185 g, 94%) as a pale brown solid. R*f* 0.43 (hexane/EtOAc, 7:3); mp 211–212 °C; IR (KBr): ῡ 2229, 1769, 1712, 1497, 1453, 1389, 1374, 1308, 1165, 1108, 857, 770, 743, 693 cm^{-1}; ^1H NMR (500 MHz, DMSO-d_6) δ 5.67 (s, 2H, CH_2Ph), 7.04 (dd, *J* = 3.0, 1.0 Hz, 1H, H-8), 7.25–7.30 (m, 3H, H-2”, H-4”), 7.34 (tm, *J* = 7.5 Hz, 2H, H-3”), 7.44 (tm, *J* = 7.5 Hz, 1H, H-4’), 7.47 (dm, *J* = 7.5 Hz, 2H, H-2’), 7.53 (tm, *J* = 7.5 Hz, 2H, H-3’), 8.23 (d, *J* = 3.0 Hz, 1H, H-7), 8.65 (d, *J* = 1.0 Hz, 1H, H-5); ^{13}C NMR (125 MHz, DMSO-d_6) δ 49.7 (CH_2Ph), 97.3 (CN), 101.3 (C-8), 116.4 (C-3a), 120.8 (C-5), 124.1 (C-4 or C-8b), 124.2 (C-8b or C-4), 125.2 (C-8a), 127.2

(C-2”), 127.3 (C-2’), 127.9 (C-4”), 128.0 (C-4’), 128.7 (C-3’), 128.8 (C-3”), 131.8 (C-1’), 136.9 (C-1”), 138.9 (C-5a), 139.0 (C-7), 165.8 (C-1 or C-3), 166.4 (C-3 or C-1); MS (70 eV): *m/z* 378 (M^++1, 14), 377 (M^+, 43), 91 (100). Anal calcd for $C_{24}H_{15}N_3O_2$: C, 76.38; H, 4.01; N, 11.13; found: C, 76.39; H, 4.03; N, 11.10.

Methyl 6-(2-bromobenzyl)-1,3-dioxo-2-phenyl-1,2,3,6-tetrahydropyrrolo[3,4-*e*]indole-4-carboxylate (21c). Method A. Following the method of preparation for **21a**, a mixture of **9h** (0.200 g, 0.41 mmol) and DDQ (0.227 g, 1.0 mmol) in CH_2Cl_2 (20 mL) led to the formation of **21c** (0.178 g, 90%) as a yellow solid. Method B. In a threaded ACE glass pressure tube equipped with a sealed Teflon screw cap and a magnetic stirring bar, a solution of **9h** (0.050 g, 0.10 mmol) and MnO_2 (0.035 g, 0.40 mmol) in toluene (1.0 mL) was heated at 140 °C for 24 h. The reaction mixture was filtered under vacuum over celite and the solvent removed under vacuum. The residue was purified by column chromatography over silica gel (20 g/g crude, hexane/EtOAc, 8:2) to give **21c** (0.034 g, 69%) as a yellow solid. R*f* 0.71 (hexane/EtOAc, 7:3); mp 209–211 °C; IR (film): ῡ 2952, 1771, 1713, 1499, 1368, 1274, 1207, 1130, 1027, 745 cm^{-1}; ^{1}H NMR (500 MHz, $CDCl_3$) δ 4.00 (s, 3H, CO_2CH_3), 5.51 (s, 2H, CH_2Ar), 6.63 (dd, *J* = 5.5, 4.0 Hz, 1H, H-6”), 7.20–7.24 (m, 3H, H-8, H-4”, H-5”), 7.37–7.41 (m, 1H, H-4’), 7.46–7.52 (m, 5H, H-7, H-2’, H-3’), 7.61–7.67 (m, 1H, H-3”), 7.96 (s, 1H, H-5); ^{13}C NMR (125 MHz, $CDCl_3$) δ 50.8 (CH_2Ar), 52.9 (CO_2CH_3), 102.6 (C-8), 115.9 (C-5), 122.2 (C-3a), 122.7 (C-2”), 122.9 (C-4), 125.1 (C-8b), 125.3 (C-8a), 126.7 (C-2’), 127.8 (C-4’), 128.0 (C-6”), 128.1 (C-5”), 129.0 (C-3’), 129.9 (C-4”), 132.0 (C-1’), 133.3 (C-3”), 134.9 (C-7), 135.7 (C-1”), 139.6 (C-5a), 166.2 (C-1 or C-3), 167.0 (CO_2CH_3), 167.2 (C-3 or C-1); HRMS (EI): m/z [M^+] calcd for $C_{25}H_{17}BrN_2O_4$: 488.0372; found: 488.0376.

Ethyl 6-(2-bromobenzyl)-2-methyl-1,3-dioxo-1,2,3,6-tetrahydropyrrolo[3,4-*e*]indole-4-carboxylate (21d). Following the method of preparation for **21a**, a mixture of **9i** (0.200 g, 0.45 mmol) and DDQ (0.256 g, 1.13 mmol) in CH_2Cl_2 (20 mL) afforded **21d** (0.171 g, 86%) as a brown solid. R*f* 0.43 (hexane/EtOAc, 7:3); mp 189–191 °C; IR (KBr): ῡ 2930, 1761, 1704, 1502, 1467, 1438, 1369, 1307, 1274, 1143, 1027, 1013, 762 cm^{-1}; ^{1}H NMR (500 MHz, $CDCl_3$) δ 1.45 (t, *J* = 7.5 Hz, 3H, $CO_2CH_2CH_3$), 3.19 (s, 3H, NCH_3), 4.47 (q, *J*

= 7.5 Hz, 2H, $CO_2CH_2CH_3$), 5.48 (s, 2H, C*H*$_2$Ar), 6.62–6.66 (m, 1H, H-6’), 7.16 (dm, *J* = 3.5 Hz, 1H, H-8), 7.17–7.22 (m, 2H, H-4’, H-5’), 7.46 (d, *J* = 3.0 Hz, 1H, H-7), 7.62–7.66 (m, 1H, H-3’), 7.88 (s, 1H, H-5); ^{13}C NMR (125 MHz, $CDCl_3$) δ 14.1 ($CO_2CH_2CH_3$), 23.8 (N*C*H$_3$), 50.7 (*C*H$_2$Ar), 62.0 ($CO_2CH_2CH_3$), 102.3 (C-8), 115.3 (C-5), 122.7 (C-2’), 122.8 (C-3a), 123.0 (C-4), 125.1 (C-8a), 125.6 (C-8b), 128.1 (C-5’), 128.2 (C-6’), 129.9 (C-4’), 133.2 (C-3’), 135.0 (C-1’), 135.4 (C-7), 139.4 (C-5a), 166.5 (*C*O$_2$Et), 167.4 (C-1 or C-3), 168.5 (C-3 or C-1); HRMS (EI): m/z [M^+] calcd for $C_{21}H_{17}N_2O_4Br$: 440.0372; found: 440.0378.

Ethyl 6-(2-bromobenzyl)-1,3-dioxo-2-phenyl-1,2,3,6-tetrahydropyrrolo[3,4-*e*]indole-4-carboxylate (21e). Following the method of preparation for **21a**, a mixture of **9j** (0.200 g, 0.39 mmol) and DDQ (0.222 g, 0.98 mmol) in CH_2Cl_2 (20 mL) provided **21e** (0.176 g, 89%) as a yellow solid. R*f* 0.45 (hexane/EtOAc, 7:3); mp 159–161 °C; IR (film): ῡ 2924, 2854, 1772, 1713, 1500, 1368, 1272, 1205, 1158, 1028, 746 cm^{-1}; ^{1}H NMR (500 MHz, $CDCl_3$) δ 1.43 (t, *J* = 7.2 Hz, 3H, $CO_2CH_2CH_3$), 4.47 (q, *J* = 7.2 Hz, 2H, $CO_2CH_2CH_3$), 5.51 (s, 2H, C*H*$_2$Ar), 6.61–6.65 (m, 1H, H-6”), 7.18–7.23 (m, 3H, H-8, H-4”, H-5”), 7.36–7.40 (m, 1H, H-4’), 7.46–7.52 (m, 5H, H-7, H-2’, H-3’), 7.62–7.66 (m, 1H, H-3”), 7.94 (s, 1H, H-5); ^{13}C NMR (125 MHz, $CDCl_3$) δ 14.1 ($CO_2CH_2CH_3$), 50.8 (Ar*C*H$_2$), 62.1 ($CO_2CH_2CH_3$), 102.5 (C-8), 115.7 (C-5), 122.1 (C-3a), 122.7 (C-2”), 123.5 (C-4), 125.0 (C-8b), 125.2 (C-8a), 126.7 (C-2’), 127.7 (C-4’), 128.1 (C-6”), 128.2 (C-5”), 129.0 (C-3’), 129.9 (C-4”), 132.0 (C-1’), 133.3 (C-3”), 134.9 (C-7), 135.6 (C-1”), 139.5 (C-5a), 166.1 (C-1 or C-3), 166.7 (*C*O$_2$CH$_2$CH$_3$), 167.2 (C-3 or C-1); HRMS (EI): m/z [M^+] calcd for $C_{26}H_{19}BrN_2O_4$: 502.0528; found: 502.0522.

Ethyl 6-(3-methoxybenzyl)-1,3-dioxo-2-phenyl-1,2,3,6-tetrahydropyrrolo[3,4-*e*]indole-4-carboxylate (21f). Following the method of preparation for **21a**, a mixture of **9k** (0.200 g, 0.44 mmol) and DDQ (0.250 g, 1.1 mmol) in CH_2Cl_2 (20 mL) produced **21f** (0.185 g, 90%) as a yellow solid. R*f* 0.63 (hexane/EtOAc, 7:3); mp 128–129 °C; IR (KBr): ῡ 3095, 2973, 2838, 1769, 1727, 1585, 1499, 1458, 1369, 1257, 1204, 1154, 1123, 1051, 1029, 869, 840, 777, 751, 691, 628 cm^{-1}; ^{1}H NMR (300 MHz, $CDCl_3$) δ 1.42 (t, *J* = 7.2 Hz, 3H, $CO_2CH_2CH_3$), 3.75 (s, 3H, C*H*$_3$O), 4.46 (q, *J* = 7.2 Hz, 2H, $CO_2CH_2CH_3$), 5.41 (s, 2H,

CH_2Ar), 6.62 (br t, *J* = 2.0 Hz, 1H, H-2”), 6.69 (ddd, *J* = 7.7, 1.2, 0.9 Hz, 1H, H-6”), 6.84 (ddd, *J* = 8.1, 2.7, 0.9 Hz, 1H, H-4”), 7.18 (dd, *J* = 3.3, 0.9 Hz, 1H, H-8), 7.25 (dd, *J* = 8.1, 7.7 Hz, 1H, H-5”), 7.35–7.41 (m, 1H, H-4’), 7.46–7.54 (m, 5H, H-7, H-2’, H-3’), 7.94 (d, *J* = 0.9 Hz, 1H, H-5); ^{13}C NMR (75.4 MHz, $CDCl_3$) δ 14.1 ($CO_2CH_2CH_3$), 50.6 (ArCH_2), 55.2 (CH_3O), 62.1 ($CO_2CH_2CH_3$), 102.2 (C-8), 112.7 (C-2”), 113.5 (C-4”), 115.8 (C-5), 118.9 (C-6”), 121.9 (C-3a), 123.2 (C-4), 124.9 (C-8a), 125.3 (C-8b), 126.8 (C-2’), 127.7 (C-4’), 129.0 (C-3’), 130.2 (C-5”), 132.0 (C-1’), 135.7 (C-7), 137.3 (C-1”), 139.5 (C-5a), 160.2 (C-3”), 166.2 (C-1 or C-3), 166.7 (CO_2Et), 167.3 (C-3 or C-1); Anal calcd for $C_{27}H_{22}N_2O_5$: C, 71.35; H, 4.88; N, 6.16; found: C, 71.37; H, 4.85; N, 6.15.

4-Acetyl-6-(2-bromobenzyl)-2-phenylpyrrolo[3,4-*e*]indole-1,3(2*H*,6*H*)-dione (21g). Following the method of preparation for **21a**, a mixture of **9m** (0.200 g, 0.42 mmol) and DDQ (0.250 g, 1.10 mmol) in CH_2Cl_2 (20 mL) furnished **21g** (0.178 g, 90%) as a yellow solid. R*f* 0.32 (hexane/EtOAc, 7:3); mp 183–185 °C; IR (film): ῡ 2934, 1661, 1508, 1358, 1272, 1215, 1186, 1063, 745 cm^{-1}; ^{1}H NMR (500 MHz, $CDCl_3$) δ 2.84 (s, 3H, COCH_3), 5.50 (s, 2H, CH_2Ar), 6.65 (dd, *J* = 5.0, 4.0 Hz, 1H, H-6”), 7.18–7.23 (m, 3H, H-8, H-4”, H-5”), 7.37–7.42 (m, 1H, H-4’), 7.46–7.54 (m, 5H, H-7, H-2’, H-3’), 7.64 (dd, *J* = 5.5, 3.5 Hz, 1H, H-3”), 7.77 (s, 1H, H-5); ^{13}C NMR (125 MHz, $CDCl_3$) δ 31.3 (COCH_3), 50.8 (ArCH_2), 102.4 (C-8), 114.5 (C-5), 121.0 (C-3a), 122.7 (C-2”), 124.5 (C-8b), 125.2 (C-8a), 126.6 (C-2’), 127.9 (C-4’), 128.1 (C-5”), 128.2 (C-6”), 129.0 (C-3’), 129.9 (C-4”), 131.8 (C-1’), 132.2 (C-4), 133.3 (C-3”), 134.9 (C-1”), 135.8 (C-7), 140.0 (C-5a), 167.2 (C-1 or C-3), 167.6 (C-3 or C-1), 201.6 (CO_2CH_3); HRMS (EI): m/z [M$^+$] calcd for $C_{25}H_{17}BrN_2O_3$: 472.0423; found: 472.0425.

(3a*S,4*S**,8b*S**)-6-Benzyl-7-formyl-1,3-dioxo-2-phenyl-1,2,3,3a,4,5,6,8b-octahydropyrrolo[3,4-*e*]indole-4-carbonitrile (22a).** Analogous procedure as described in [S2]. After adding phosphorus oxychloride (0.120 g, 0.79 mmol) to anhydrous dimethylformamide (0.058 g, 0.79 mmol) at 0 ºC, the mixture was stirred for 10 min before a solution of **9f** (0.200 g, 0.52 mmol) in anhydrous CH_2Cl_2 (10.0 mL) was added dropwise. The temperature was then slowly raised to reflux and the mixture was maintained at that point for 1 h prior to adding a 1.0 M solution of KOH until reaching neutral. Subsequently,

CH_2Cl_2 (50 mL) was added, the organic layer was dried (Na_2SO_4) and the solvent removed under vacuum. Through purification of the residue by column chromatography over silica gel (20 g/g crude, hexane/EtOAc, 1:1), **22a** (0.203 g, 95%) was obtained as an orange solid. R*f* 0.38 (hexane/EtOAc, 7:3); mp 114–115 °C; IR (KBr): ῡ 2943, 2799, 2244, 1783, 1717, 1660, 1597, 1496, 1384, 1189, 1135, 1095, 911, 835, 784, 732, 693 cm^{-1}; ^{1}H NMR (300 MHz, $CDCl_3$) δ 2.75 (dd, *J* = 16.5, 4.5 Hz, 1H, H-5), 2.88 (dd, *J* = 16.5, 6.9 Hz, 1H, H-5), 3.40 (dt, *J* = 6.9, 4.5 Hz, 1H, H-4), 3.52 (dd, *J* = 8.4, 4.5 Hz, 1H, H-3a), 4.05 (d, *J* = 8.4 Hz, 1H, H-8b), 5.47 (d, *J* = 16.4 Hz, 1H, CH_2Ph), 5.62 (d, *J* = 16.4 Hz, 1H, CH_2Ph), 6.92 (dm, *J* = 7.8 Hz, 2H, H-2”), 7.19 (s, 1H, H-8), 7.20–7.29 (m, 5H, H-2’, H-3”, H-4”), 7.29–7.45 (m, 3H, H-4’, H-3’), 9.49 (s, 1H, C*H*O); ^{13}C NMR (75.4 MHz, $CDCl_3$) δ 22.8 (C-5), 26.0 (C-4), 38.4 (C-8b), 41.1 (C-3a), 48.2 (CH_2Ph), 112.3 (C-8a), 118.0 (CN), 122.8 (C-8), 126.0 (C-2’), 126.3 (C-2”), 127.6 (C-4”), 128.7 (C-3”), 128.8 (C-4’), 129.1 (C-3’), 131.2 (C-1’), 132.4 (C-7), 134.5 (C-5a), 136.5 (C-1”), 174.0 (C-1 or C-3), 174.1 (C-3 or C-1), 179.6 (*C*HO); MS (70 eV): m/z 410 (M^++1, 23), 409 (M^+, 81), 382 (33), 235 (18), 218 (13), 91 (100). HRMS (EI): m/z [M^+] calcd for $C_{25}H_{19}N_3O_3$: 409.1426; found: 409.1422.

Methyl (3aR*,4S*,8bS*)-6-(2-bromobenzyl)-7-formyl-1,3-dioxo-2-phenyl-1,2,3,3a,4,5,6,8b-octahydropyrrolo[3,4-*e*]indole-4-carboxylate (22b). Following the method of preparation for **22a**, a mixture of anhydrous DMF (0.053 g, 0.73 mmol), $POCl_3$ (0.112 g, 0.73 mmol) and **9h** (0.300 g, 0.61 mmol) in anhydrous CH_2Cl_2 (30.0 mL), after stirring at 0 °C for 3 h, delivered **22b** (0.280 g, 88%) as an amber oil. R*f* 0.65 (hexane/EtOAc, 1:1); IR (film): ῡ 2952, 1714, 1661, 1496, 1437, 1383, 1249, 1196, 1126, 1028, 822, 785, 754, 734, 693 cm^{-1}; ^{1}H NMR (500 MHz, $CDCl_3$) δ 2.76 (dd, *J* = 17.0, 11.0 Hz, 1H, H-5), 2.88 (dd, *J* = 17.0, 5.0 Hz, 1H, H-5), 3.10 (dt, *J* = 10.5, 5.0 Hz, 1H, H-4), 3.71 (s, 3H, CO_2CH_3), 4.07 (dd, *J* = 8.0, 5.0 Hz, 1H, H-3a), 4.25 (d, *J* = 8.5 Hz, 1H, H-8b), 5.59 (d, *J* = 17.0 Hz, 1H, CH_2Ar), 5.69 (d, *J* = 17.0 Hz, 1H, CH_2Ar), 6.12-6.16 (m, 1H, H-6”), 7.08–7.13 (m, 2H, H-4”, H-5”), 7.19–7.23 (m, 2H, H-2’), 7.24 (s, 1H, H-8), 7.34–7.38 (m, 1H, H-4’), 7.40–7.45 (m, 2H, H-3’), 7.55–7.59 (m, 1H, H-3”), 9.51 (s, 1H, C*H*O); ^{13}C NMR (125 MHz, $CDCl_3$) δ 20.3 (C-5), 38.3 (C-4), 40.1 (C-8b), 41.9 (C-3a), 48.8 (CH_2Ar), 52.5 (CO_2CH_3), 113.7 (C-8a), 121.7 (C-2”), 122.7 (C-8), 125.8 (C-6”), 126.1 (C-2’), 127.8 (C-5”), 128.6 (C-4’), 128.8 (C-4”), 129.0 (C-3’), 131.5 (C-1’), 132.5 (C-7), 132.8 (C-3”),

136.3 (C-1”), 137.1 (C-5a), 171.4 (CO_2CH_3), 175.3 (C-1 or C-3), 175.4 (C-3 or C-1), 179.3 (*C*HO); HRMS (EI): m/z [M^+] calcd for $C_{26}H_{21}BrN_2O_5$: 520.0634; found: 520.0625.

Methyl (3a*R,4*S**,8b*S**)-6-(2-bromo-4,5-dimethoxybenzyl)-7-formyl-1,3-dioxo-2-phenyl-1,2,3,3a,4,5,6,8b-octahydropyrrolo[3,4-*e*]indole-4-carboxylate (22c).** Following the method of preparation for **22a**, a mixture of anhydrous DMF (0.040 g, 0.54 mmol), $POCl_3$ (0.083 g, 0.54 mmol) and **9l** (0.200 g, 0.36 mmol) in anhydrous CH_2Cl_2 (20.0 mL), after stirring at 0 °C for 2 h, resulted in **22c** (0.187 g, 89%) as a yellow solid. R*f* 0.38 (hexane/EtOAc, 1:1); mp 88–89 °C; IR (film): ῡ 2952, 1715, 1662, 1506, 1436, 1382, 1262, 1209, 1161, 1126, 1031, 804, 785, 733, 714, 691 cm^{-1}; ^1H NMR (500 MHz, $CDCl_3$) δ 2.76 (dd, *J* = 17.0, 10.5 Hz, 1H, H-5), 2.94 (dd, *J* = 17.0, 5.0 Hz, 1H, H-5), 3.06–3.12 (m, 1H, H-4), 3.47 (s, 3H, CH_3O), 3.72 (s, 3H, CO_2CH_3), 3.84 (s, 3H, CH_3O), 4.06 (dd, *J* = 8.5, 4.5 Hz, 1H, H-3a), 4.24 (d, *J* = 8.5 Hz, 1H, H-8b), 5.53 (d, *J* = 16.5 Hz, 1H, CH_2Ar), 5.66 (d, *J* = 16.5 Hz, 1H, CH_2Ar), 5.83 (s, 1H, H-6”), 7.03 (s, 1H, H-3”), 7.18–7.21 (m, 2H, H-2’), 7.24 (s, 1H, H-8), 7.35 (tt, *J* = 7.5, 2.5 Hz, 1H, H-4’), 7.41 (tm, *J* = 7.5 Hz, 2H, H-3’), 9.55 (s, 1H, C*H*O); ^{13}C NMR (125 MHz, $CDCl_3$) δ 20.5 (C-5), 38.3 (C-4), 40.1 (C-8b), 41.9 (C-3a), 48.2 (CH_2Ar), 52.5 (CO_2CH_3), 55.9 (CH_3O), 56.2 (CH_3O), 109.6 (C-6”), 111.9 (C-2”), 113.8 (C-8a), 115.7 (C-3”), 122.8 (C-8), 125.9 (C-2’), 128.0 (C-1”), 128.6 (C-4’), 129.0 (C-3’), 131.4 (C-1’), 132.5 (C-7), 137.4 (C-5a), 148.94 (C-4” or C-5”), 148.96 (C-5” or C-4”), 171.4 (CO_2CH_3), 175.2 (C-1 or C-3), 175.3 (C-3 or C-1), 179.3 (*C*HO); HRMS (EI): m/z [M^+] calcd for $C_{28}N_{25}BrN_2O_7$: 580.0845; found: 580.0821.

Methyl (3a*R,4*S**,8b*S**)-6-allyl-7-formyl-1,3-dioxo-2-phenyl-1,2,3,3a,4,5,6,8b-octahydropyrrolo[3,4-*e*]indole-4-carboxylate (22d).** Following the method of preparation for **22a**, a mixture of anhydrous DMF (0.073 g, 0.99 mmol), $POCl_3$ (0.152 g, 0.99 mmol) and **9o** (0.300 g, 0.82 mmol) in anhydrous CH_2Cl_2 (30.0 mL), after stirring at rt for 2 h, led to the formation to **22d** (0.271 g, 84%) as a yellow solid. R*f* 0.43 (hexane/EtOAc, 1:1); mp 154–155 °C; IR (KBr): ῡ 1714, 1652, 1496, 1439, 1382, 1248, 1196, 1122, 1033, 920, 820, 801, 785, 758, 713, 692 cm^{-1}; ^1H NMR (500 MHz, $CDCl_3$) δ 2.86 (dd, *J* = 16.9, 11.0 Hz, 1H, H-5), 3.02 (dd, *J* = 16.9, 5.0 Hz, 1H, H-5), 3.10 (ddd, *J* = 11.0, 5.0, 4.5 Hz, 1H, H-4), 3.81 (s, 3H, CO_2CH_3), 4.10 (dd, *J* = 8.5, 4.5 Hz, 1H, H-3a), 4.23 (d, *J* = 8.5 Hz, 1H, H-8b),

4.75 (d, *J* = 17.0 Hz, 1H, H-3”), 4.92 (dm, *J* = 16.8 Hz, 1H, H-1”), 5.03 (dm, *J* = 16.8 Hz, 1H, H-1”), 5.11 (d, *J* = 10.0 Hz, 1H, H-3”), 5.88–5.96 (m, 1H, H-2”), 7.13 (s, 1H, H-8), 7.19–7.22 (m, 2H, H-2’), 7.32–7.35 (m, 1H, H-4’), 7.39–7.41 (m, 2H, H-3’). 9.48 (s, 1H, C*H*O); ^{13}C NMR (125 MHz, $CDCl_3$) δ 20.3 (C-5), 38.3 (C-4), 40.3 (C-8b), 42.0 (C-3a), 47.2 (C-1”), 52.6 ($CO_2\mathit{C}H_3$), 113.1 (C-8a), 116.1 (C-3”), 122.5 (C-8), 126.1 (C-2’), 128.6 (C-4’), 129.0 (C-3’), 131.5 (C-1’), 132.1 (C-7), 133.4 (C-2”), 136.8 (C-5a), 171.6 ($\mathit{C}O_2CH_3$), 175.4 (C-1 or C-3), 175.5 (C-3 or C-1), 179.2 (*C*HO); MS (70 eV): m/z 392 (M^+, 100), 375 (54), 363 (23), 333 (65), 332 (75), 315 (69), 304 (20), 212 (15), 185 (70), 168 (41), 158 (43), 130 (24), 117 (22); HRMS (EI): m/z [M^+] calcd for $C_{22}H_{20}N_2O_5$: 392.1372; found: 392.1379; Anal calcd for $C_{22}H_{20}N_2O_5$: C, 67.34; H, 5.14; N, 7.14; found: C, 67.33; H, 5.09; N, 7.13.

Methyl (3a*R,4*S**,8b*S**)-7-formyl-1,3-dioxo-2-phenyl-6-(prop-2-yn-1-yl)-1,2,3,3a,4,5,6,8b-octahydropyrrolo[3,4-*e*]indole-4-carboxylate (22e).** Following the method of preparation for **22a**, a mixture of anhydrous DMF (0.073 g, 0.99 mmol), $POCl_3$ (0.152 g, 0.99 mmol) and **9p** (0.300 g, 0.83 mmol) in anhydrous CH_2Cl_2 (30.0 mL), after stirring at rt for 2 h, afforded **22e** (0.258 g, 80%) as a yellow solid. R*f* 0.40 (hexane/EtOAc, 1:1); mp 268–269 °C; IR (KBr): ῡ 3295, 2950, 2126, 1711, 1658, 1495, 1433, 1388, 1321, 1272, 1201, 1125, 1025, 966, 882, 847, 803, 761, 719, 663 cm^{-1}; ^{1}H NMR (300 MHz, $CDCl_3$) δ 2.35 (t, *J* = 2.6 Hz, 1H, H-3”), 2.93 (dd, *J* = 14.1, 5.4 Hz, 1H, H-5), 3.08–3.23 (m, 2H, H-4, H-5), 3.80 (s, 3H, $CO_2C\mathit{H}_3$), 4.09 (dm, *J* = 8.4, 1H, H-3a), 4.20 (d, *J* = 8.4 Hz, 1H, H-8b), 5.12 (dd, *J* = 17.7, 2.4 Hz, 1H, H-1”), 5.28 (dd, *J* = 17.7, 2.4 Hz, 1H, H-1”), 7.11 (s, 1H, H-8), 7.16–7.25 (m, 2H, H-2’), 7.30–7.36 (m, 1H, H-4’), 7.36–7.44 (m, 2H, H-3’), 9.47 (s, 1H, C*H*O); ^{13}C NMR (75.4 MHz, $CDCl_3$) δ 20.2 (C-5), 34.2 (C-1”), 38.0 (C-4), 39.9 (C-8b), 41.8 (C-3a), 52.4 ($CO_2\mathit{C}H_3$), 73.1 (C-3”), 77.5 (C-2”), 113.6 (C-8a), 122.7 (C-8), 125.9 (C-2’), 128.4 (C-4’), 128.8 (C-3’), 131.2 (C-7 or C-1’), 131.3 (C-1’ or C-7), 136.9 (C-5a), 171.4 ($\mathit{C}O_2CH_3$), 175.2 (C-1 or C-3), 175.3 (C-1 or C-3), 179.4 (*C*HO); HRMS (EI): m/z [M^+] calcd for $C_{22}H_{18}N_2O_5$: 390.1216; found: 390.1220; Anal calcd for $C_{22}H_{18}N_2O_5$: C, 67.69; H, 4.65; N, 7.18; found: C, 67.63; H, 4.69; N, 7.15.

Ethyl 1,3-dioxo-2-phenyl-1,2,3,7-tetrahydroisoindolo[2,1-*a*]pyrrolo[3,4-*e*]indole-4-carboxylate (12). In a threaded ACE glass pressure tube equipped with a sealed Teflon screw cap and a magnetic stirring bar, a solution of **11a** (0.050 g, 0.12 mmol) and MnO_2 (0.081 g, 0.93 mmol) in CH_2Cl_2 (2.0 mL) was heated at 100 °C for 12 h. The reaction mixture was filtered under vacuum over celite and the solvent removed under vacuum. The residue was purified by column chromatography over silica gel (20 g/g crude, hexane/EtOAc, 85:5) to give **12** (0.035 g, 71%) as an orange solid. R*f* 0.38 (hexano/AcOEt, 7:3); mp 224–226 °C; IR (film): ῡ 2928, 1767, 1712, 1506, 1365, 1266, 1095, 1023, 756, 691 cm^{-1}; ^{1}H NMR (500 MHz, CD_2Cl_2/acetone-d_6/$CDCl_3$, 80:13:7) δ 1.44 (t, J = 7.1 Hz, 3H, $CO_2CH_2CH_3$), 4.46 (q, J = 7.1 Hz, 3H, $CO_2CH_2CH_3$), 5.23 (s, 2H, H-7), 7.19 (s, 1H, H-12), 7.40–7.44 (m, 1H, H-4'), 7.44–7.54 (m, 6H, H-9, H-10, H-2', H-3'), 7.59 (br d, J = 7.0 Hz, 1H, H-8), 7.83 (br d, J = 7.0 Hz, 1H, H-11), 7.99 (s, 1H, H-5); ^{13}C NMR (125 MHz, CD_2Cl_2/acetone-d_6/$CDCl_3$, 80:13:7) δ 14.1 ($CO_2CH_2CH_3$), 49.4 (C-7), 62.0 ($CO_2CH_2CH_3$), 91.4 (C-12), 115.0 (C-5), 121.7 (C-3a), 122.3 (C-11), 124.0 (C-4), 124.6 (C-12b), 127.1 (C-2'), 127.8 (C-4'), 128.8 (C-10), 129.0 (C-3'), 129.3 (C-9), 129.5 (C-12a), 131.2 (C-7a), 132.5 (C-1'), 137.1 (C-5a), 142.3 (C-11a), 151.9 (C-11b), 166.3 (C-1 or C-3), 166.6 ($CO_2CH_2CH_3$), 167.5 (C-3 or C-1); HRMS (EI): m/z [M^+] calcd for $C_{26}H_{18}N_2O_4$: 422.1267; found: 422.1265.

Single-Crystal X-ray Crystallography. Adducts **9m** and **10m** were obtained as colorless crystals and crystallized on a mixture of hexane/EtOAc (8:2), which were mounted on glass fibers. Crystallographic measurements were performed by utilizing an area-detector with Mo Kα radiation (λ = 71073 Å; graphite monochromator) at rt. Unit cell parameters were obtained from a least-squares refinement. Intensities were corrected for Lorentz and polarization effects. No absorption correction was applied. Anisotropic temperature factors were introduced for all non-hydrogen atoms. Hydrogen atoms were placed in idealized positions and their atomic coordinates refined by employing unit weights. After the structure was solved using SHELXT [S9], it was visualized and plotted on the MERCURY program [S10]. Data for **9m**: (CCDC **1987245**) Formula: $C_{25}H_{21}BrN_2O_3$; molecular weight: 477.35; cryst. syst.: orthorhombic; space group: P c a 21; unit cell parameters: *a*, 10.8111(4), *b*, 11.5651(7), *c*, 17.8855(7) (Å); *α*, 90º, *β*, 90º, *γ*, 90º; temp. (K): 291(2); Z: 4;

No. of reflections collected: 12660; no. of independent reflections: 6914; no. of reflections observed: 2904; data collection range: 3.442 < θ < 32.624°; *R*: 0.0558; GOF: 0.946. Data for **10m**: (CCDC **1987244**) Formula: $C_{25}H_{21}BrN_2O_3$; molecular weight: 477.35; cryst. syst.: triclinic; space group: P -1; unit cell parameters: *a*, 9.3102(3), *b*, 10.9639(5), *c*, 11.1414(4) (Å); α, 97.296(3)°., β, 103.708(3)°, γ, 98.674(3)°; temp. (K): 291(2); Z: 2; No. of reflections collected: 23063; no. of independent reflections: 7192; no. of reflections observed: 5385; data collection range: 3.246 < θ < 32.547°; *R*: 0.0395; GOF: 1.009.

Theoretical Calculations. All *ab initio* and DFT calculations were carried out on the Gaussian 09 program [S11]. Optimizations of the stationary points were initially made at the HF/6-31G(d,p) level of theory. The optimized geometry was used as the starting points for further optimizations at the M06-2X/6-31+G(d,p) level of theory. For all optimizations, the OPT=TIGHT option was employed. For all DFT calculations, the INT(GRID=ULTRAFINE) option was selected. The TSs were located with the QST2, QST3 or TS optimization options. Additional confirmation of the nature of the TSs was furnished by IRC analyses, which was performed for all the reaction coordinates under study. All stationary points were characterized by frequency calculations. All minima (starting materials and adducts) showed only real vibrational frequencies, while the TSs each displayed a single negative eigenvalue of the Hessian matrix. Through visual inspection of the normal mode associated with the imaginary vibrational frequency, it was confirmed that the TSs corresponded to motion along the reaction coordinate. The short contact analysis (distance and angles measurement) of the *endo*-TSs was conducted on the MERCURY program [S10].

References

S1. Martínez-Mora, E. I.; Caracas, M. A.; Escalante, C. H.; Madrigal, D. A.; Quiroz-Florentino, H.; Delgado, F.; Tamariz, J. *J. Mex. Chem. Soc.* **2016**, *60*, 23–33. doi:10.29356/jmcs.v60/1.67

S2. Martínez-Mora, E. I.; Caracas, M. A.; Escalante, C. H.; Espinoza-Hicks, C.; Quiroz-Florentino, H.; Delgado, F.; Tamariz, J. *Synthesis* **2016**, *48*, 1055–1068. doi:10.1055/s-0035–1561331

S3. Mouloungui, Z.; Murengezi, I.; Delmas, M.; Gaset, A. *Synth. Commun*. **1988**, *18*, 1241–1245. doi:10.1080/0039791880860916

S4. Campbell, S. E.; Comer, M. C.; Derbyshire, P. A.; Despinoy, X. L. M.; McNab, H.; Morrison, R.; Sommerville, C. C.; Thornley, C. *J. Chem. Soc., Perkin Trans. 1* **1997**, 2195–2202. doi:10.1039/A701749l

S5. Shin, J.-L.; Nguyen, T. S.; May, J. A. *Angew. Chem. Int. Ed*. **2015**, *54*, 9931–9935. doi:10.1002/ange.20150528

S6. Antonio, Y.; De la Cruz, M. E.; Galeazzi, Guzman, A.; Bray, B. L.; Greenhouse, R.; Kurz, L. J.; Lustig, D. A.; Maddox, M. L.; Muchowski, J. M. *Can. J. Chem.* **1994**, *72*, 15–22. doi:10.1139/v94-004

S7. Corona, A.; Di Leva, F. S.; Thierry, S.; Pescatori, L.; Crucitti, G. C.; Subra, F.; Delelis, O.; Esposito, F.; Rigogliuso G.; Costi, R.; Cosconati, S.; Novellino, E.; Di Santo, R.; Tramontano, E. *Antimicrob. Agents Chemother.* **2014**, *58*, 6101–6110. doi:10.1128/AAC.03605-14

S8. Murthy, S. N.; Nageswar, Y. V. D. *Tetrahedron Lett.* **2011**, *52*, 4481–4484. doi:10.1016/j.tetlet.2011.06.077

S9. Sheldrick, G. M. *Acta Cryst. A* **2015**, *A71*, 3-8. doi:10.1107/S205327331402630

S10. Macrae, C. F.; Edgington, P. R.; McCabe, P.; Pidcock, E.; Shields, G. P.; Taylor, R.; Towler, M.; van de Streek, J. *J. Appl. Cryst.* **2006**, *39*, 453-457. doi:10.1107/S00188980600731X

S11. Frisch, M. J.; Trucks, G. W.; Schlegel, H. B.; Scuseria, G. E.; Robb, M. A.; Cheeseman, J. R.; Scalmani, G.; Barone, V.; Mennucci, B.; Petersson, G. A.; Nakatsuji, H.; Caricato, M.; Li, X.; Hratchian, H. P.; Izmaylov, A. F.; Bloino, J.; Zheng, G.; Sonnenberg, J. L.; Hada, M.; Ehara, M.; Toyota, K.; Fukuda, R.;

Hasegawa, J.; Ishida, M.; Nakajima, T.; Honda, Y.; Kitao, O.; Nakai, H.; Vreven, T.; Montgomery, J. A.; Peralta, J. E.; Ogliaro, F.; Bearpark, M.; Heyd, J. J.; Brothers, E.; Kudin, K. N.; Staroverov, V. N.; Kobayashi, R.; Normand, J.; Raghavachari, K.; Rendell, A.; Burant, J. C.; Iyengar, S. S.; Tomasi, J.; Cossi, M.; Rega, N.; Millam, J. M.; Klene, M.; Knox, J. E.; Cross, J. B.; Bakken, V.; Adamo, C.; Jaramillo, J.; Gomperts, R.; Stratmann, R. E.; Yazyev, O.; Austin, A. J.; Cammi, R.; Pomelli, C.; Ochterski, J. W.; Martin, R. L.; Morokuma, K.; Zakrzewski, V. G.; Voth, G. A.; Salvador, P.; Dannenberg, J. J.; Dapprich, S.; Daniels, A. D.; Farkas, O.; Foresman, J. B.; Ortiz, J. V.; Cioslowski, J.; Fox, D. J. Gaussian 09, Revision A.1. Wallingford CT: Gaussian, Inc; 2009.

Appendix 8.

^{1}H and ^{13}C NMR spectra of all new compounds.

^{1}H NMR (500 MHz, $CDCl_3$) of compound **13b**.

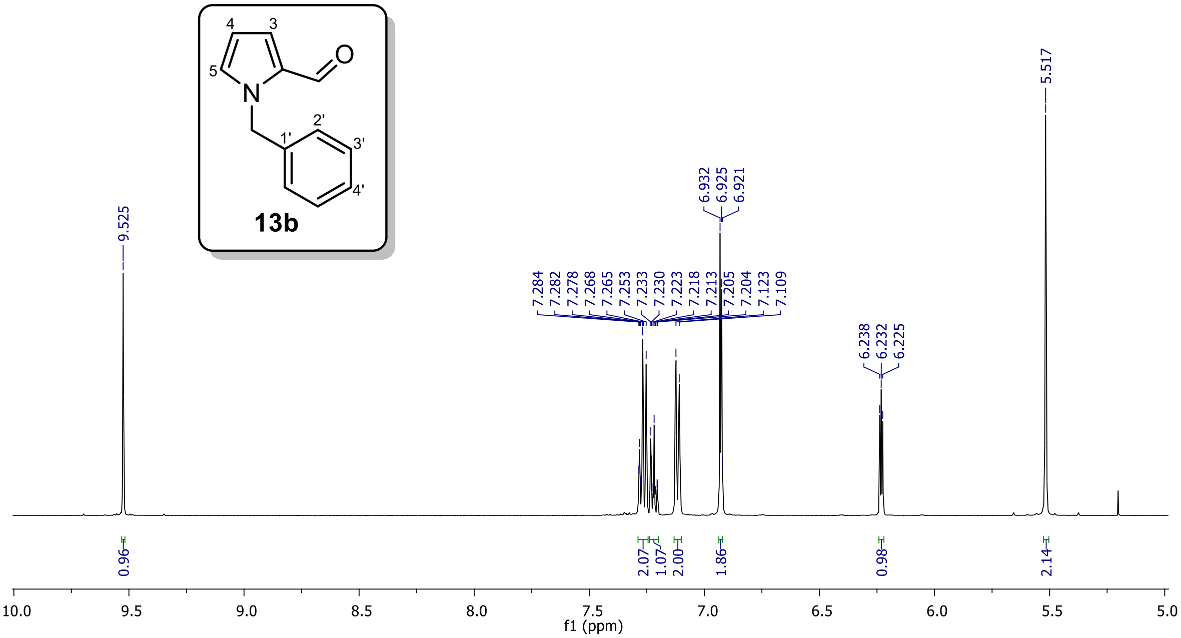

^{13}C NMR (125 MHz, $CDCl_3$) of compound **13b**.

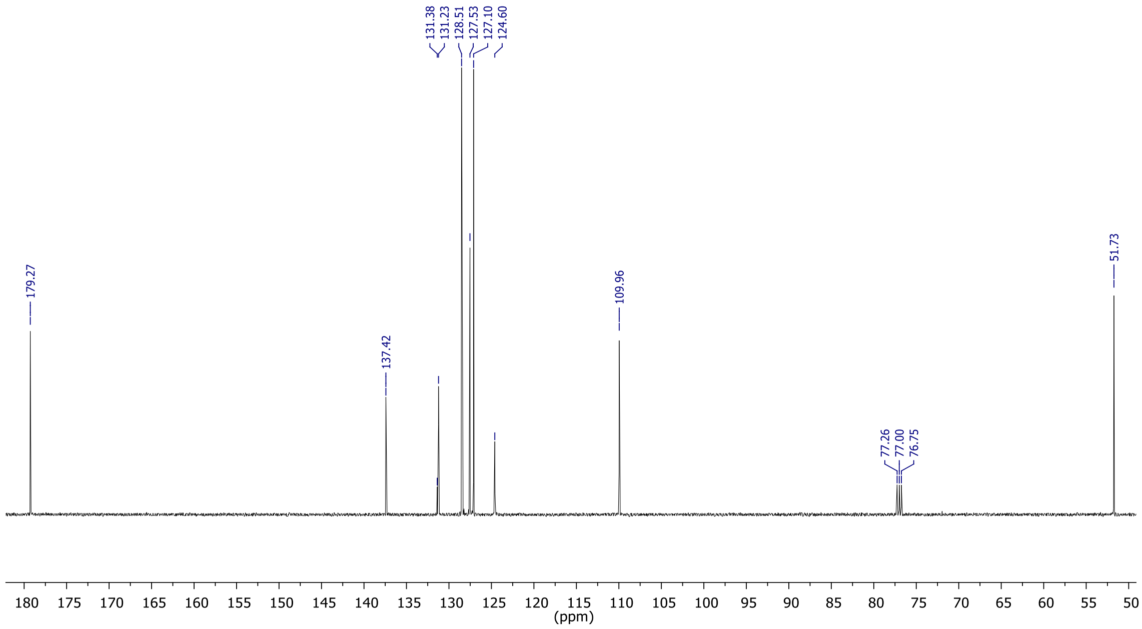

^{1}H NMR (500 MHz, $CDCl_3$) of compound **13c**.

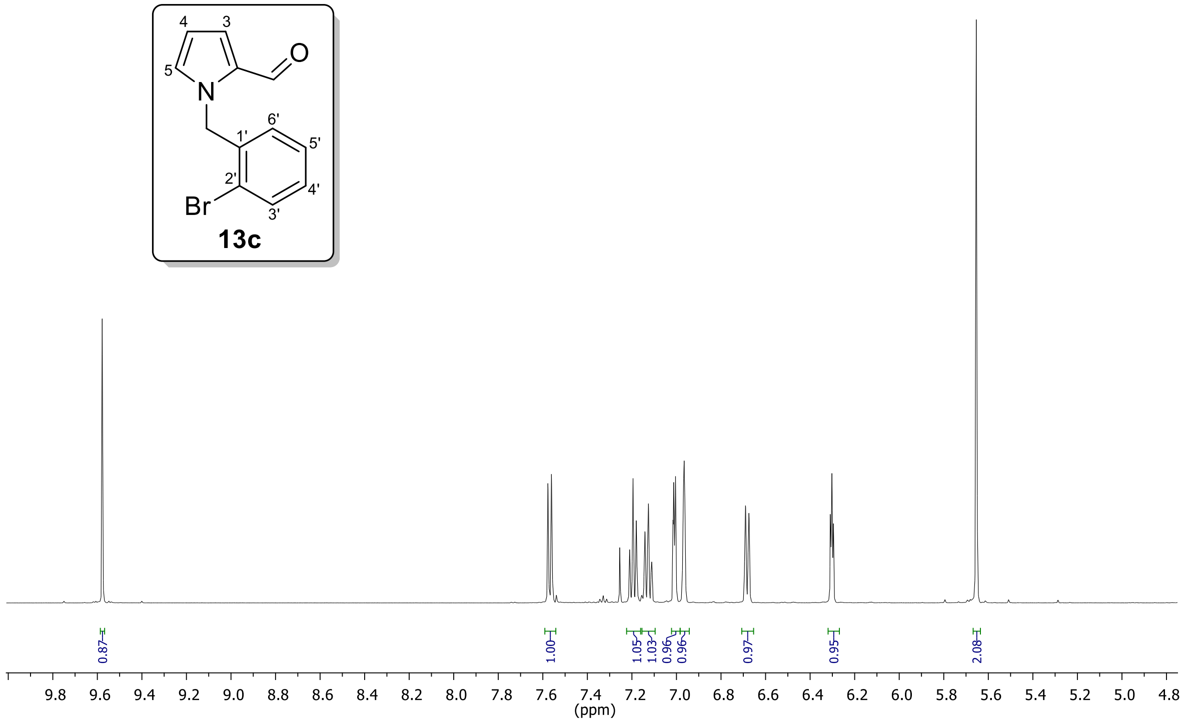

^{13}C NMR (125 MHz, $CDCl_3$) of compound **13c**.

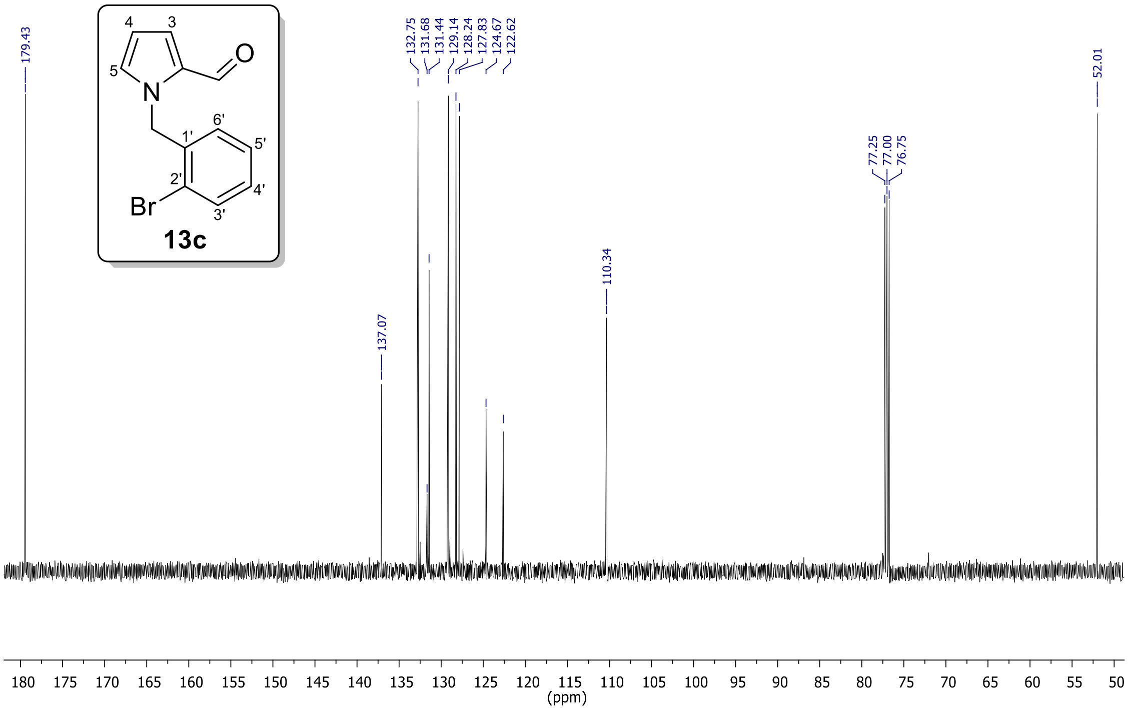

13c
Br
N
O
179.43
137.07
132.75
131.68
131.44
129.14
128.24
127.83
124.67
122.62
110.34
77.25
77.00
76.75
52.01
(ppm)

^{1}H NMR(500 MHz, $CDCl_3$) of compound **13d**.

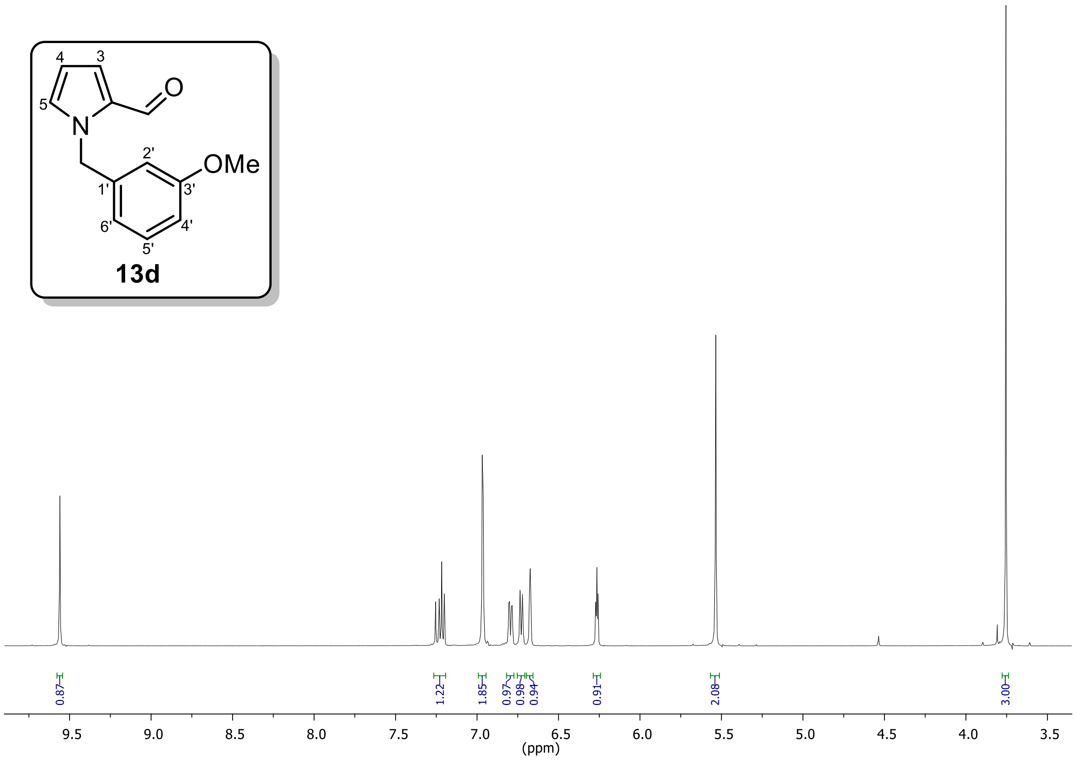

^{13}C NMR (125 MHz, $CDCl_3$) of compound **13d**.

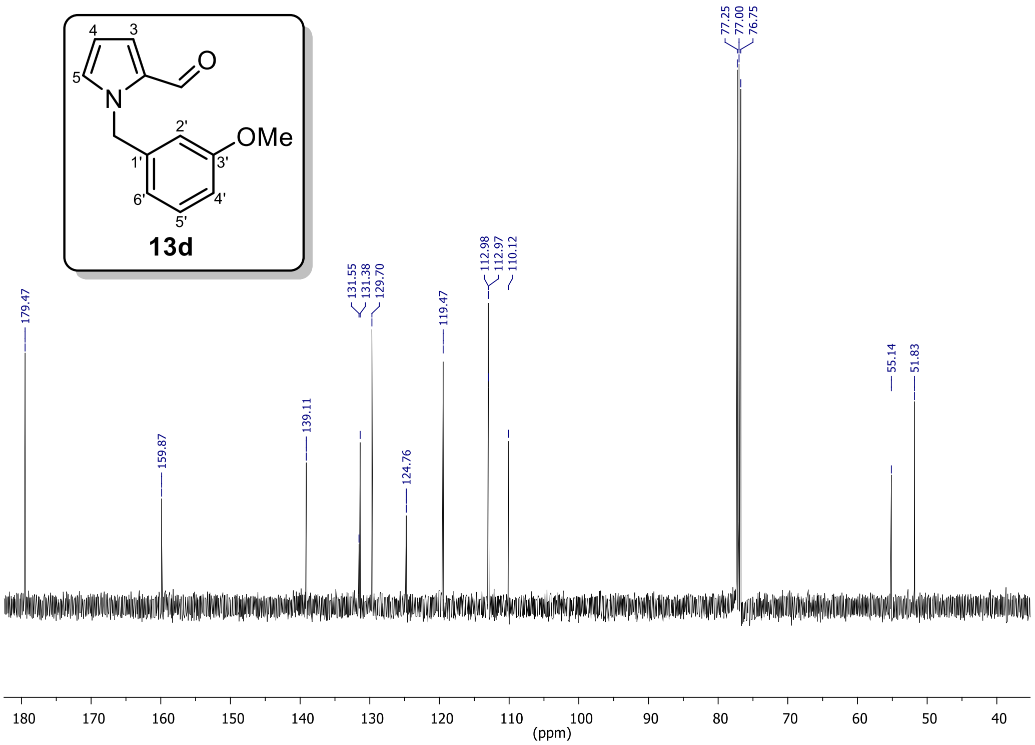

^{1}H NMR (300 MHz, $CDCl_3$) of compound **13e**.

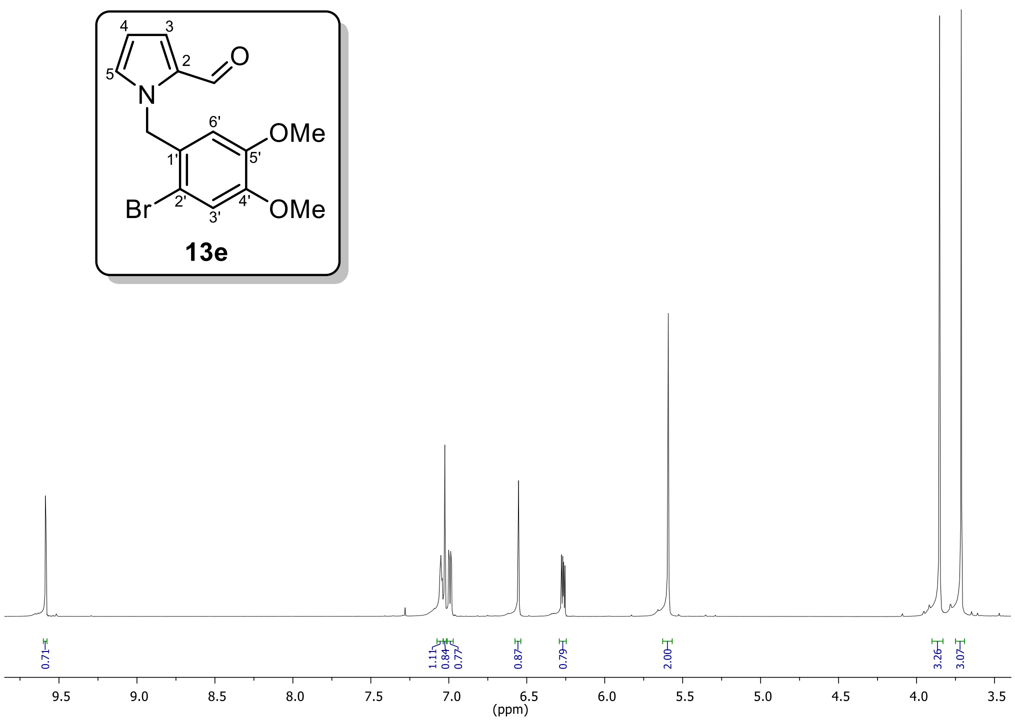

^{13}C NMR (75.4 MHz, $CDCl_3$) of compound **13e**.

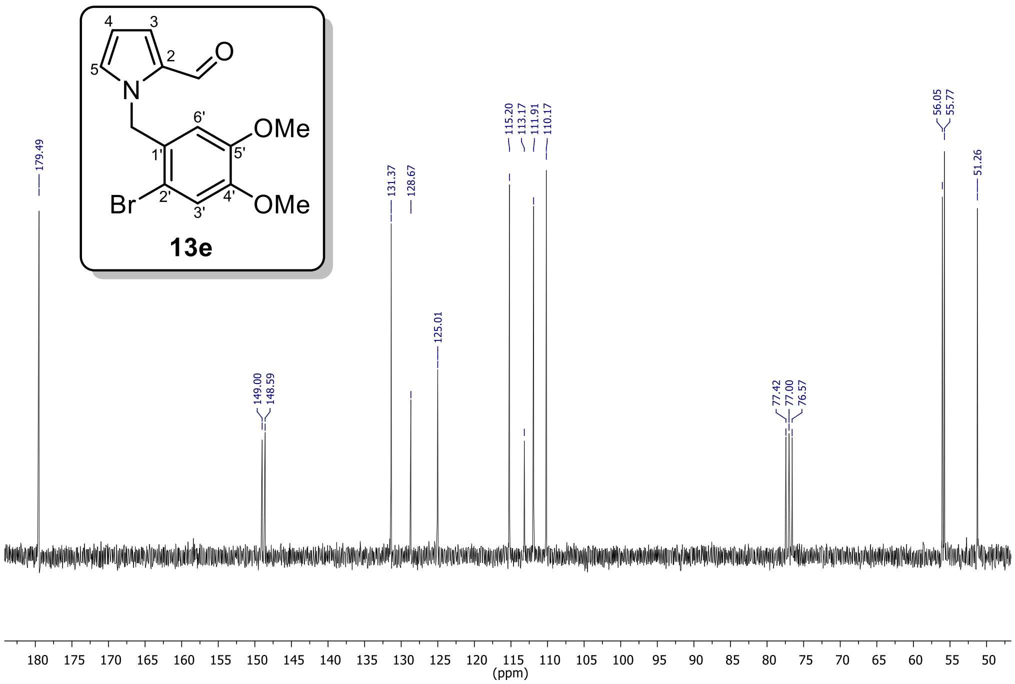

^{1}H NMR (500 MHz, $CDCl_3$) of compound **8a**.

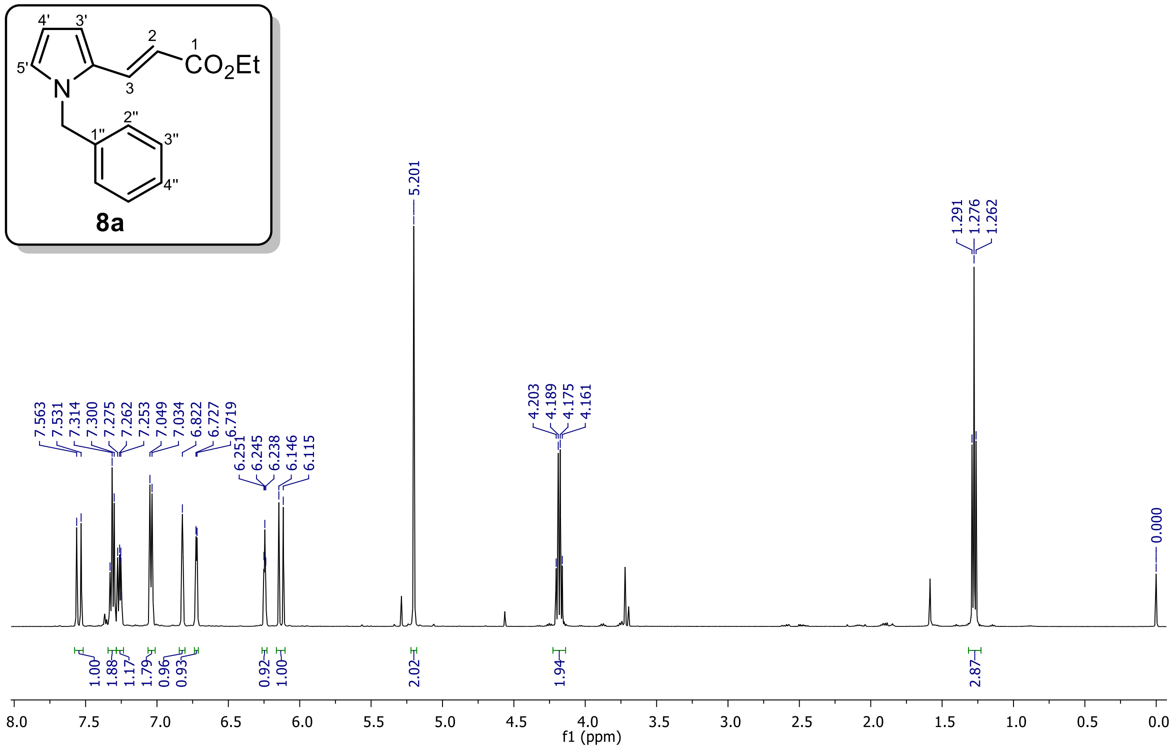

^{13}C NMR (125 MHz, $CDCl_3$) of compound **8a**.

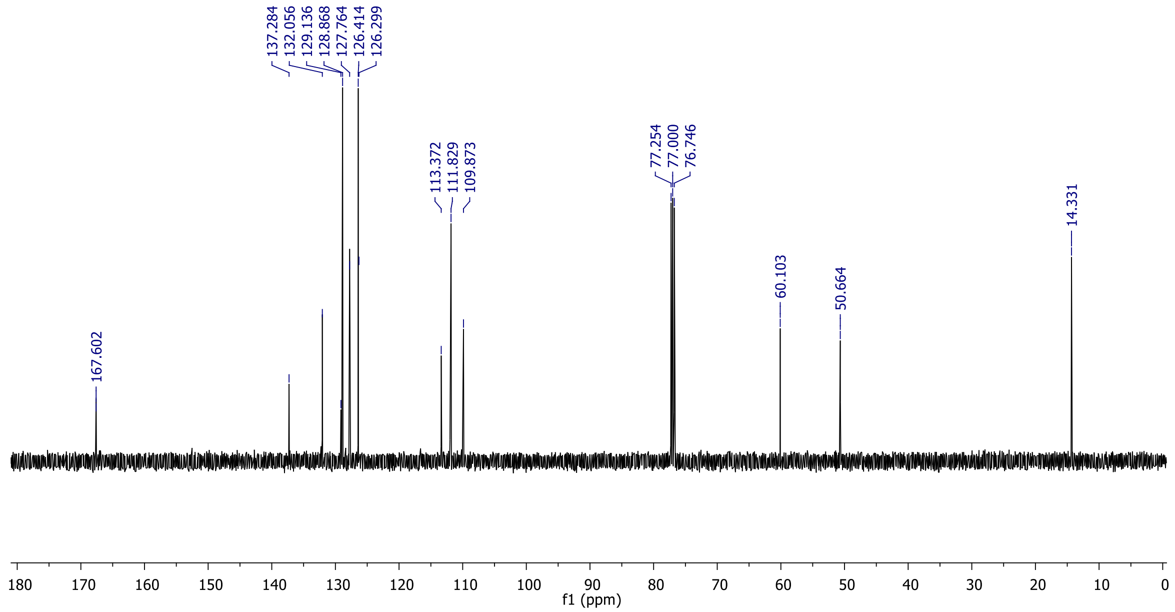

^{1}H NMR (500 MHz, $CDCl_3$) of compounds **(*E*)-8b** and **(*Z*)-8b**.

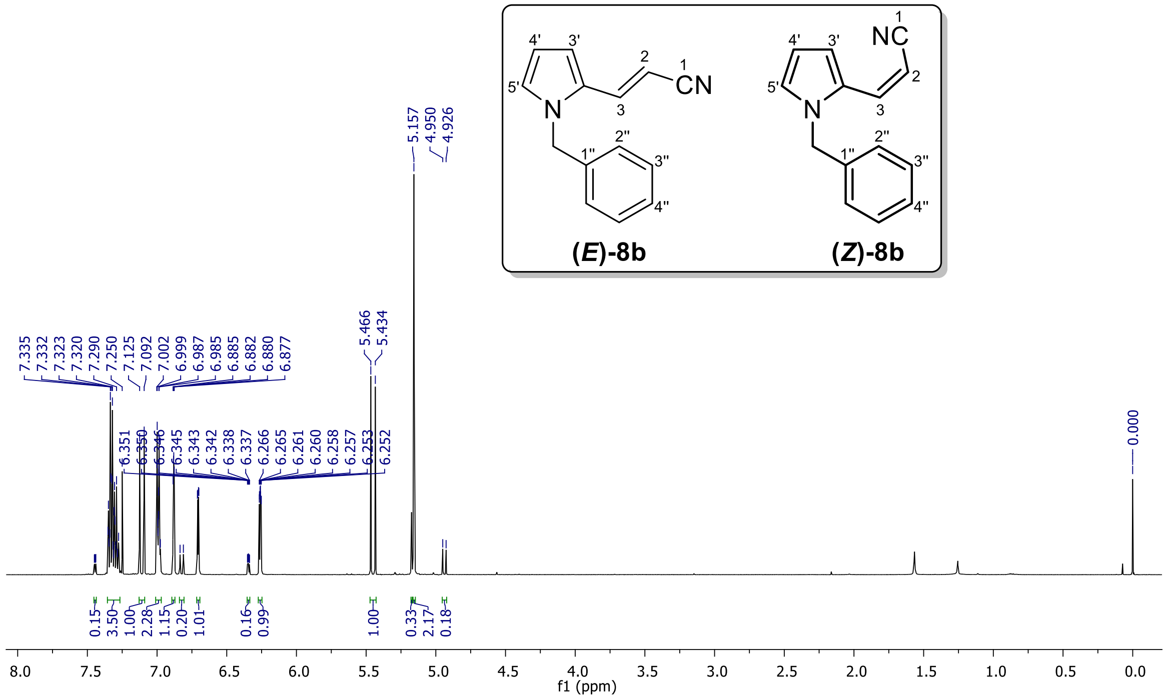

^{13}C NMR (125 MHz, $CDCl_3$) of compounds **(*E*)-8b** and **(*Z*)-8b**.

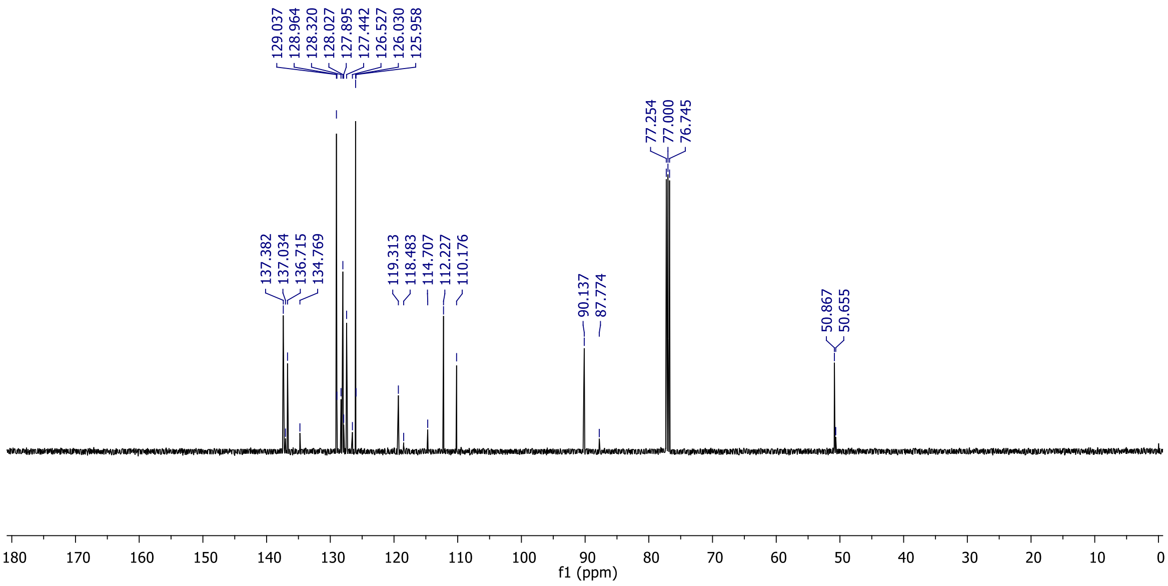

^{1}H NMR (300 MHz, $CDCl_3$)of compound **8c**.

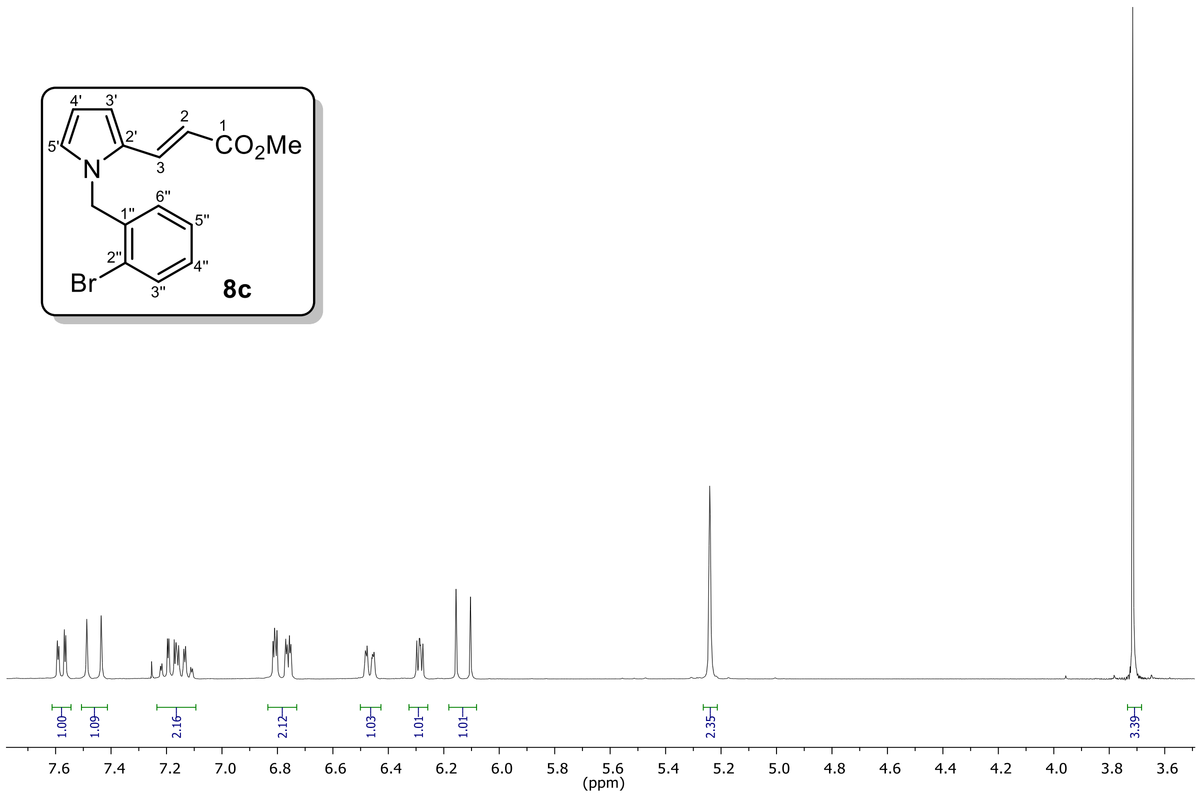

^{13}C NMR (75.4 MHz, $CDCl_3$)of compound **8c**.

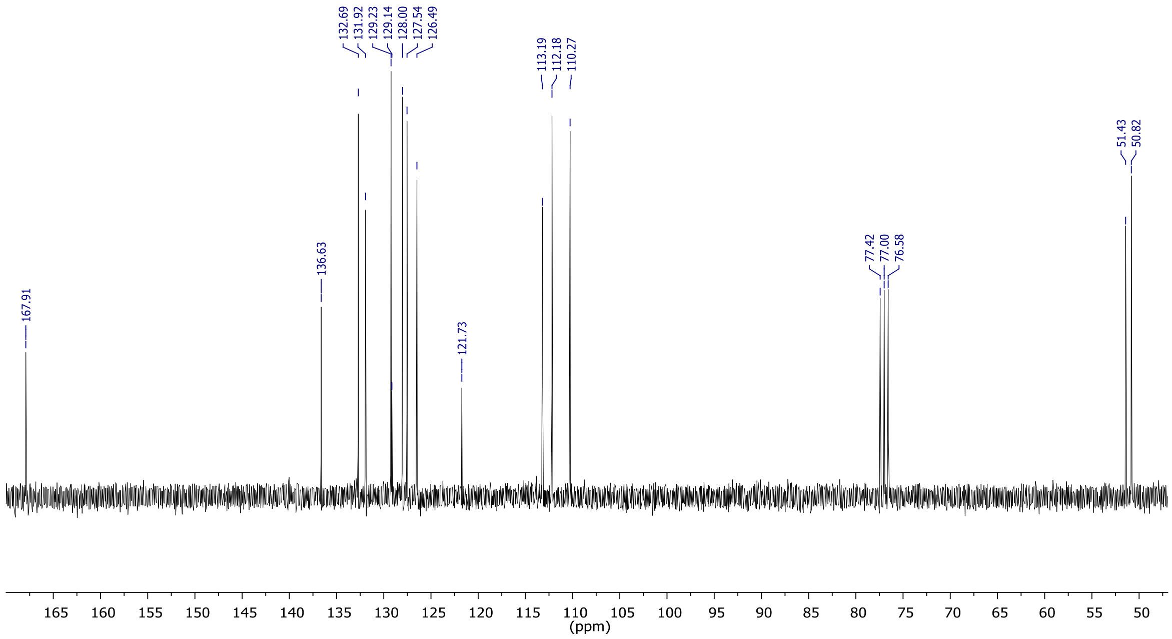

^{1}H NMR (500 MHz, $CDCl_3$) of compound **8d**.

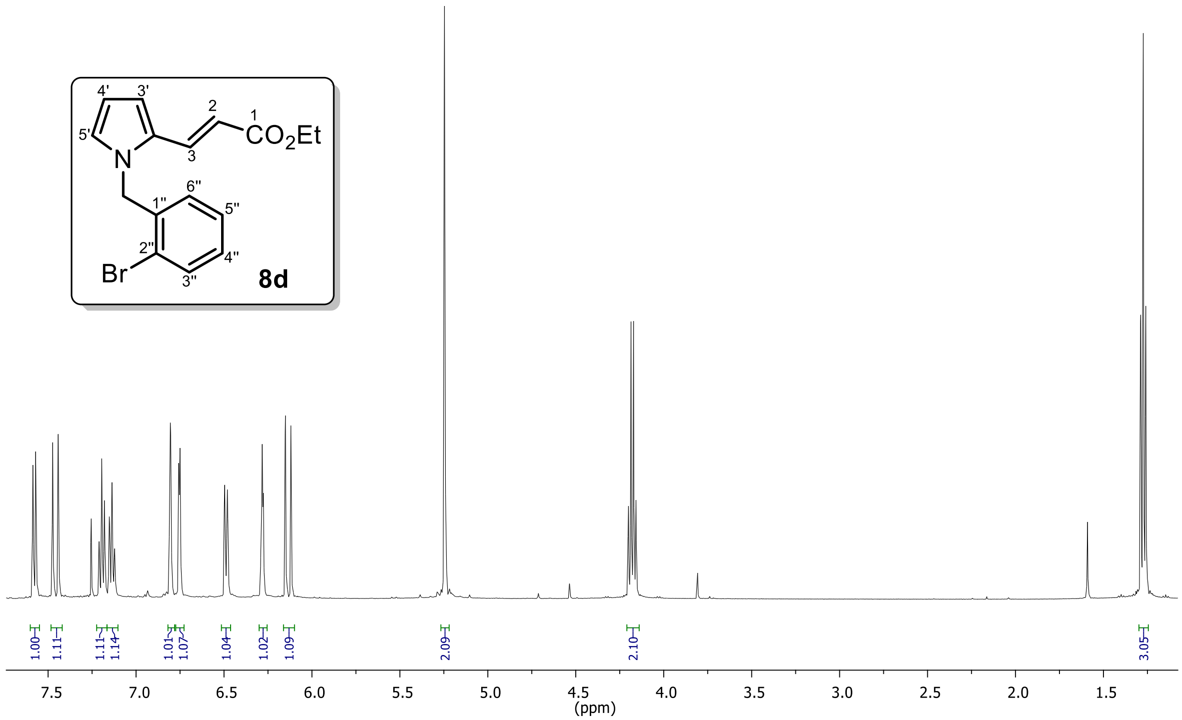

^{13}C NMR (125 MHz, $CDCl_3$) of compound **8d**.

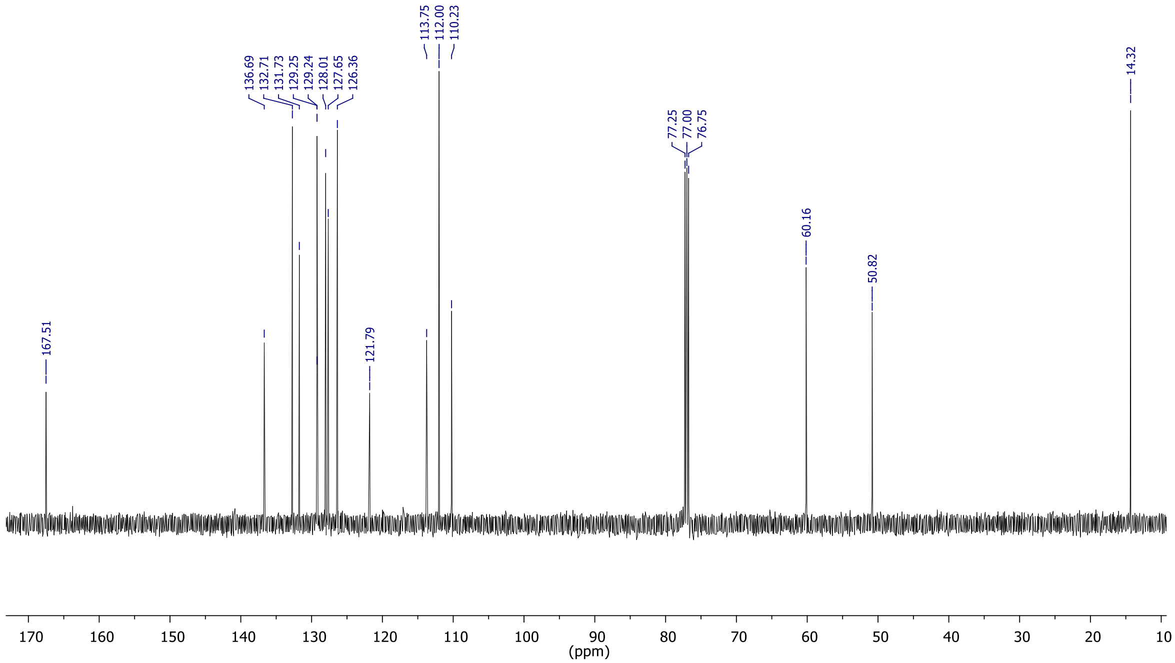

^{1}H NMR (500 MHz, $CDCl_3$) of compound **8e**.

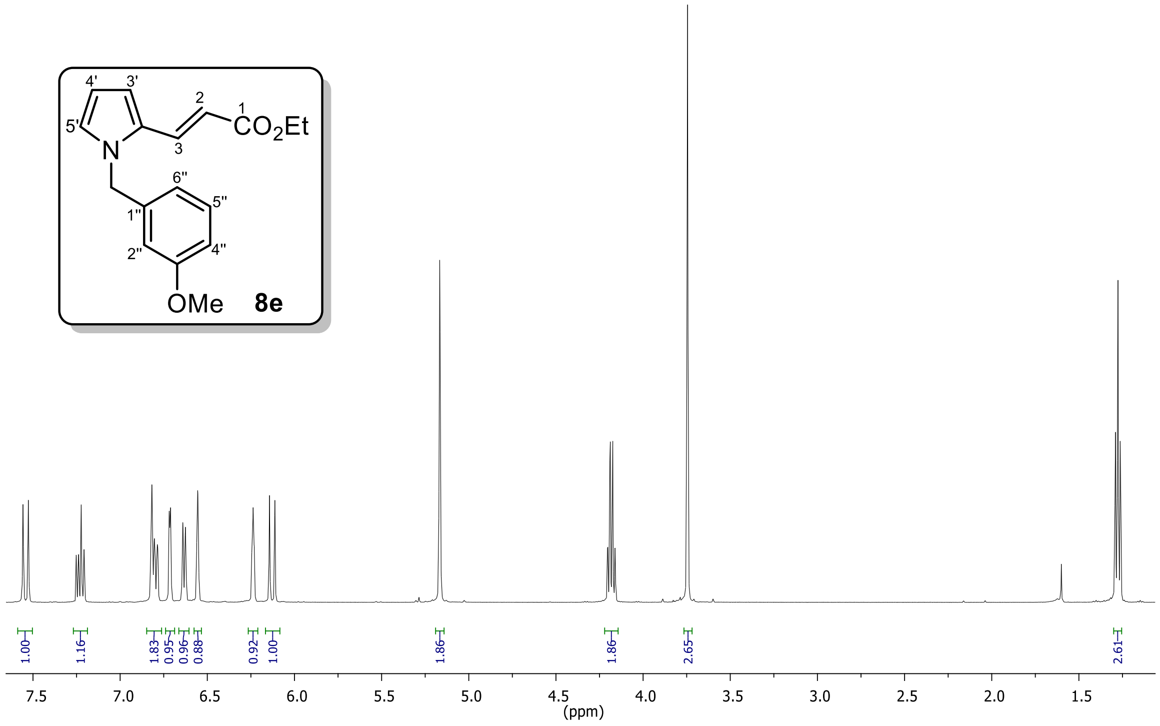

^{13}C NMR (125 MHz, $CDCl_3$) of compound **8e**.

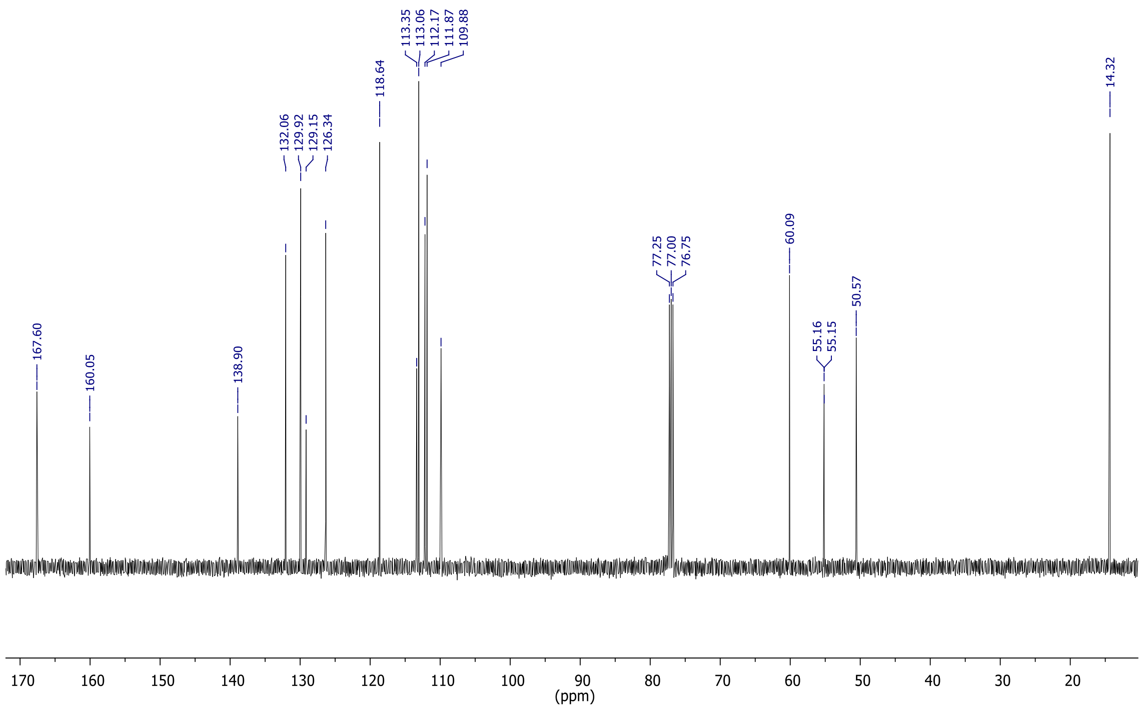

^{1}H NMR (300 MHz, $CDCl_3$) of compound **8f**.

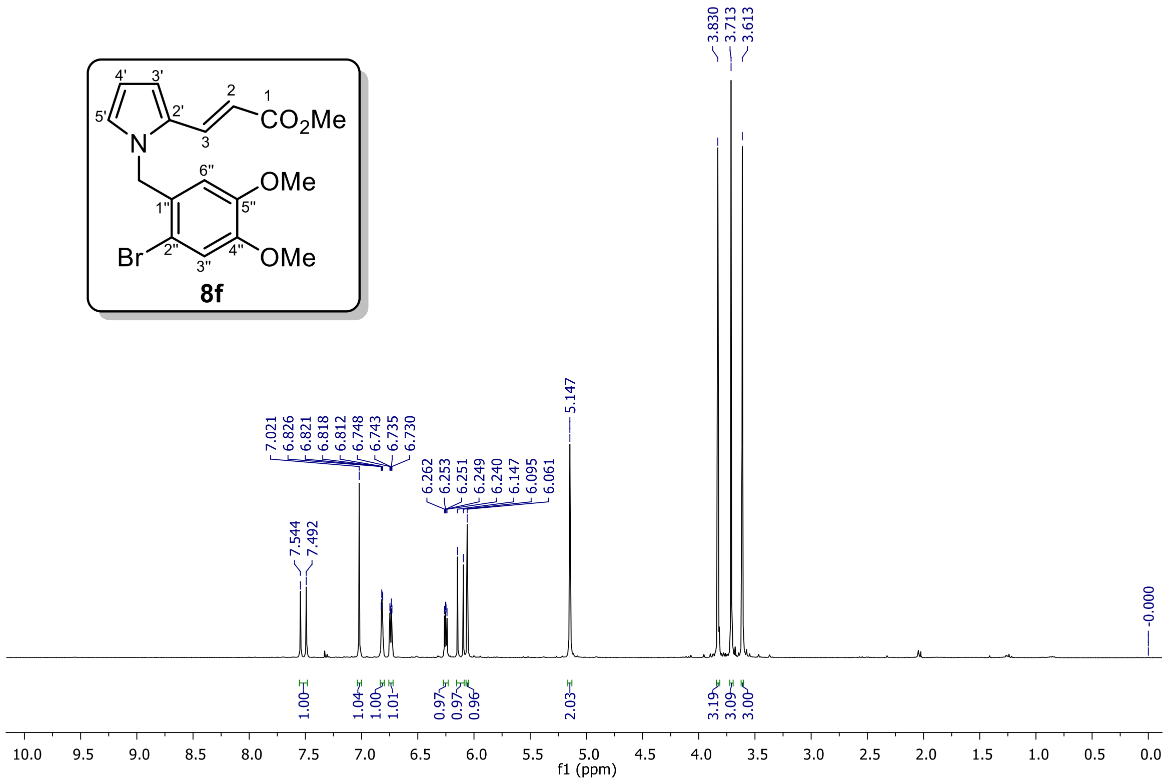

^{13}C NMR (75.4 MHz, $CDCl_3$) of compound **8f**.

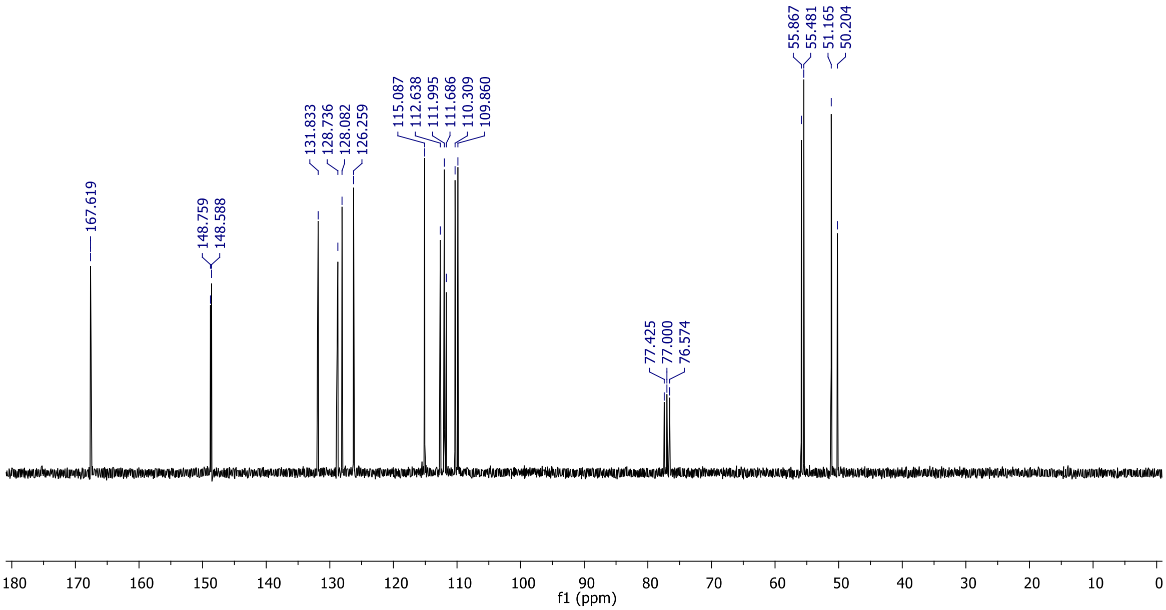

^{1}H NMR (500 MHz, $CDCl_3$) of compound **8g**.

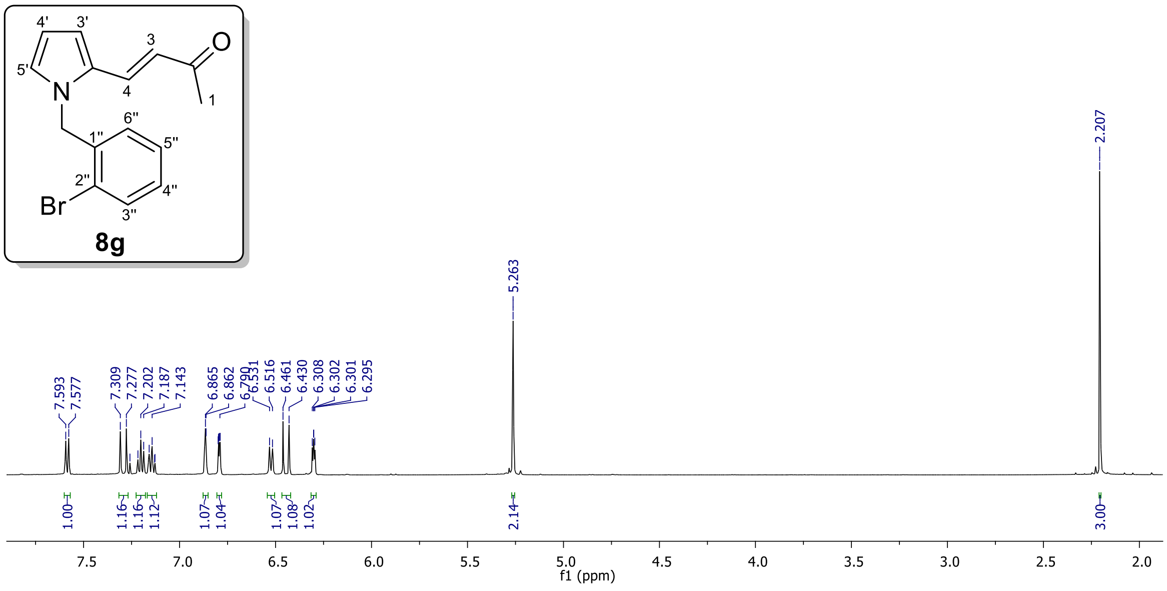

^{13}C NMR (125 MHz, $CDCl_3$) of compound **8g**.

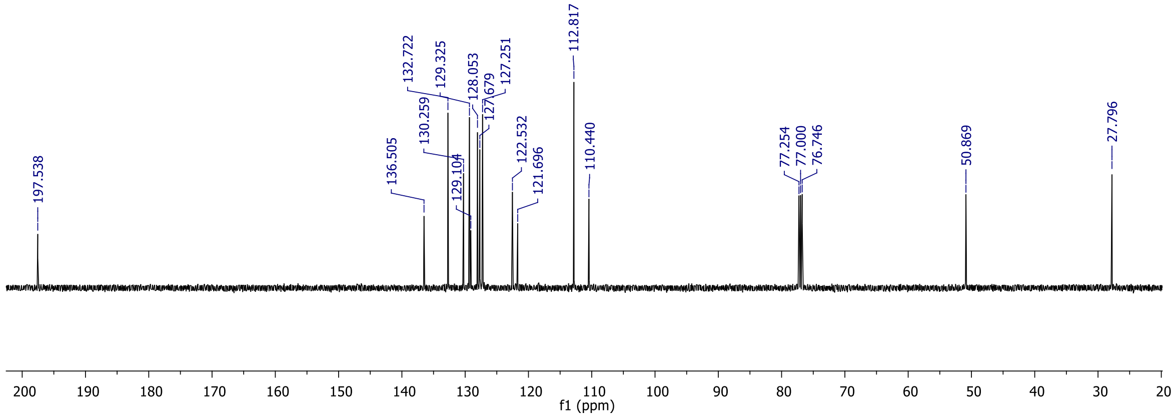

^{1}H NMR (300 MHz, $CDCl_3$) of compound **8h**.

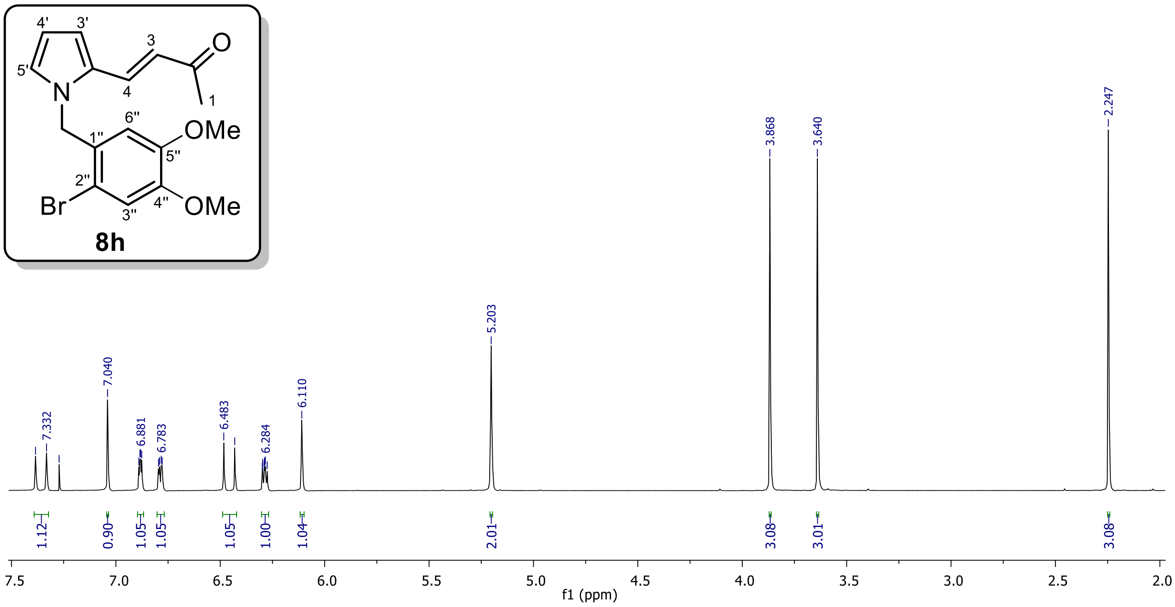

^{13}C NMR (74.5 MHz, $CDCl_3$) of compound **8h**.

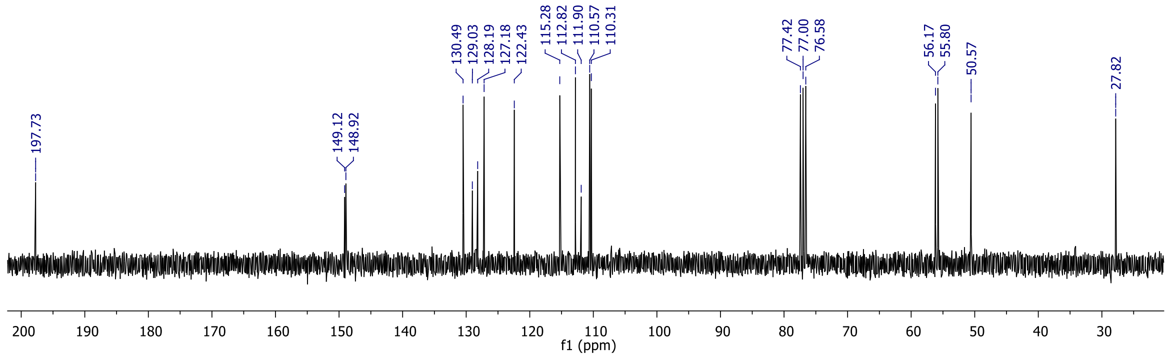

^{1}H NMR(500 MHz, $CDCl_3$) of compound **8k**.

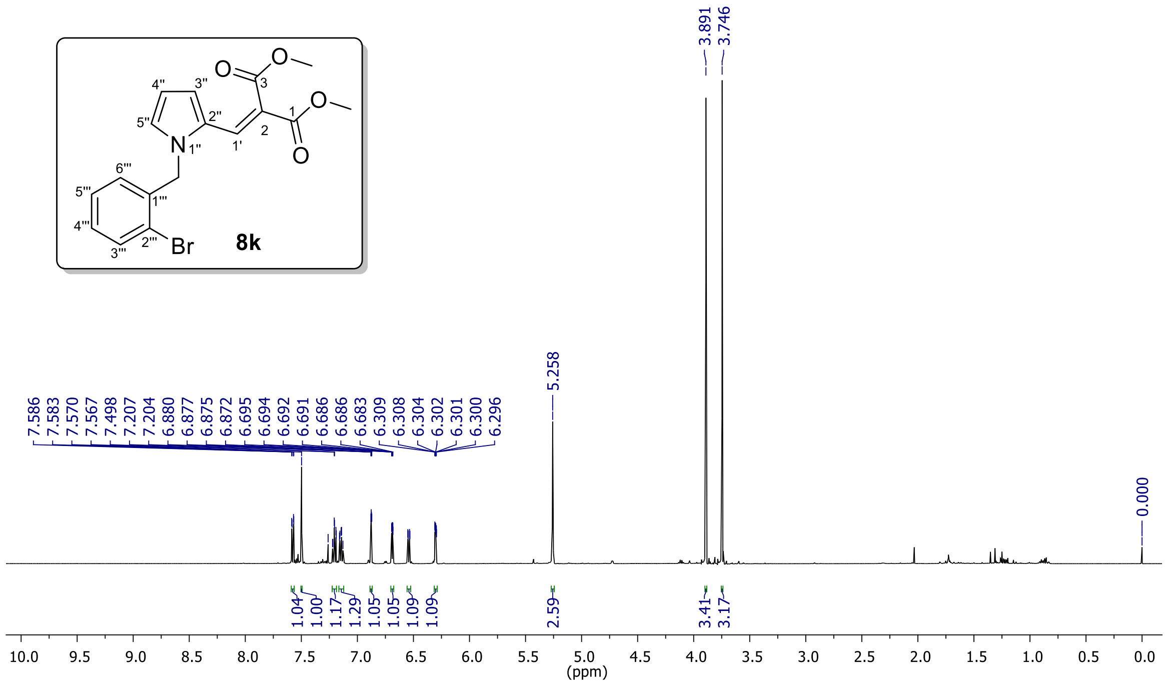

^{13}C NMR (125 MHz, $CDCl_3$) of compound **8k**.

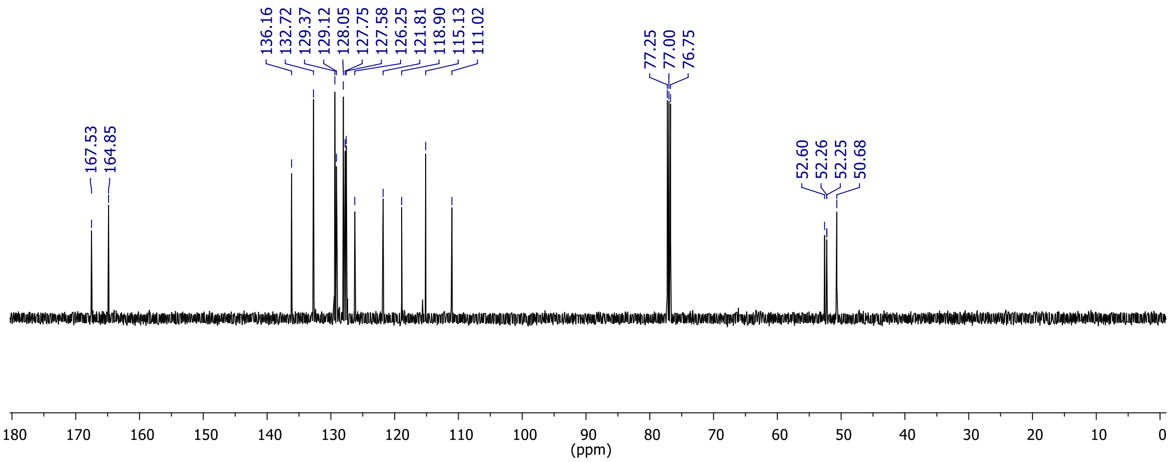

^{1}H NMR (500 MHz, $CDCl_3$) of compound **8l**.

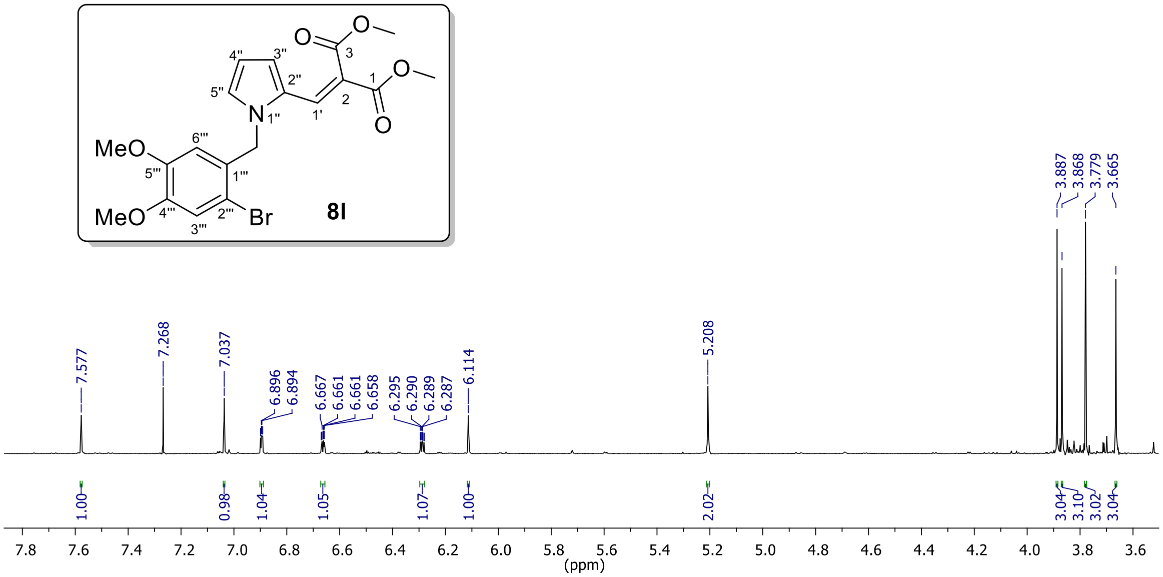

^{13}C NMR (125 MHz, $CDCl_3$) of compound **8l**.

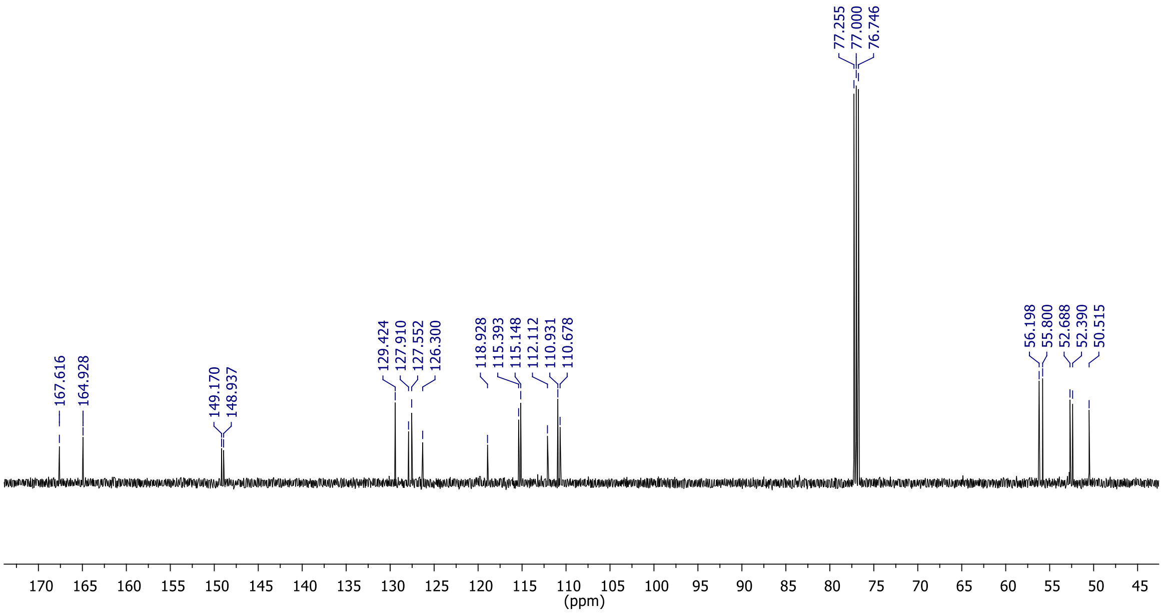

^{1}H NMR (600 MHz, $CDCl_3$) of compound **9a**.

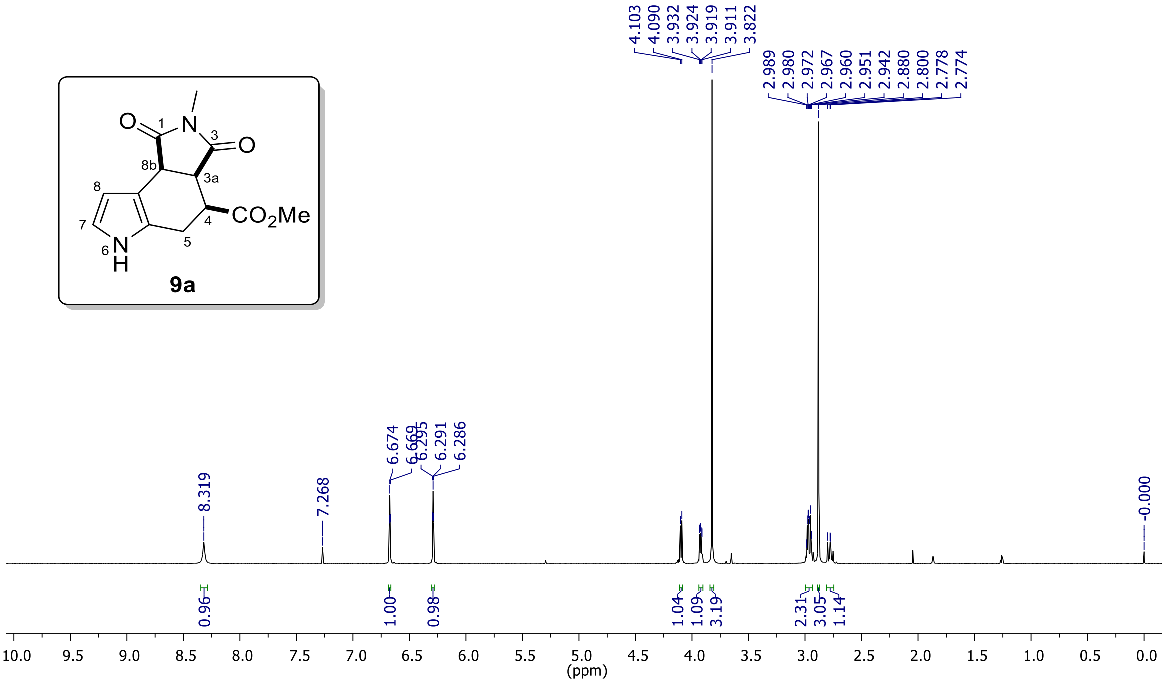

^{13}C NMR (150 MHz, $CDCl_3$) of compound **9a**.

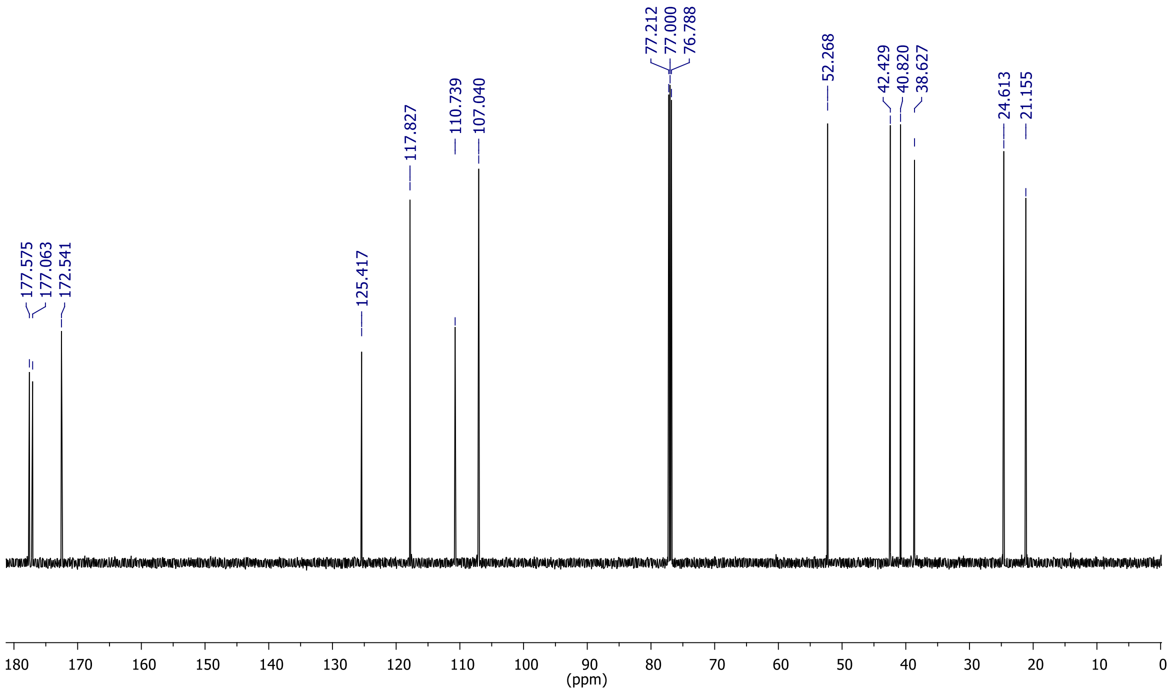

^{1}H NMR (500 MHz, $CDCl_3$) of compound **9b**.

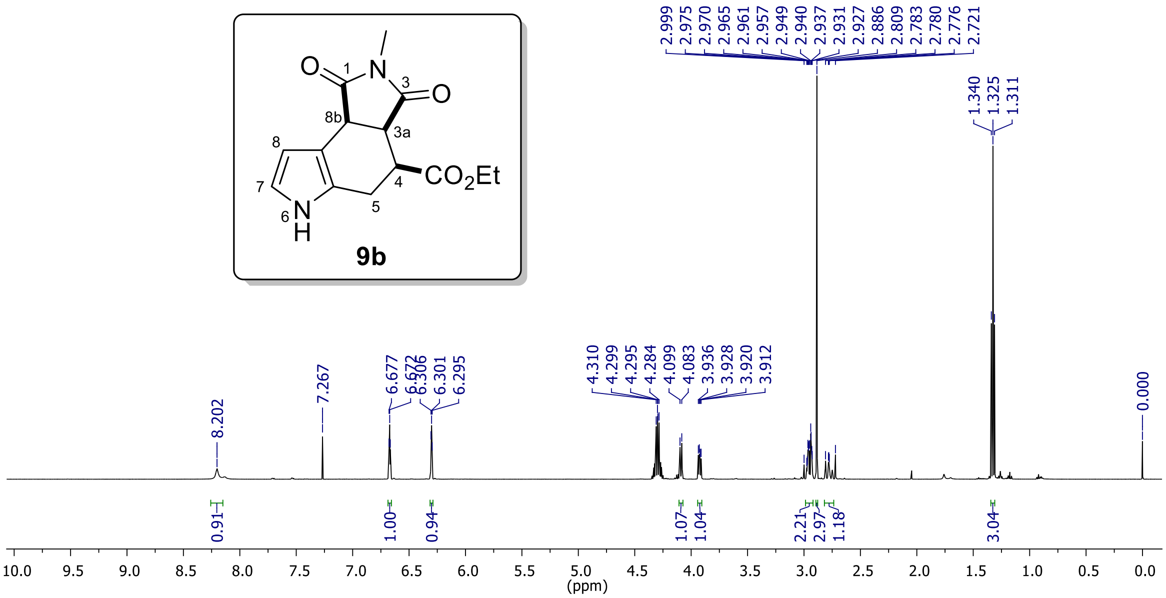

^{13}C NMR (125 MHz, $CDCl_3$) of compound **9b**.

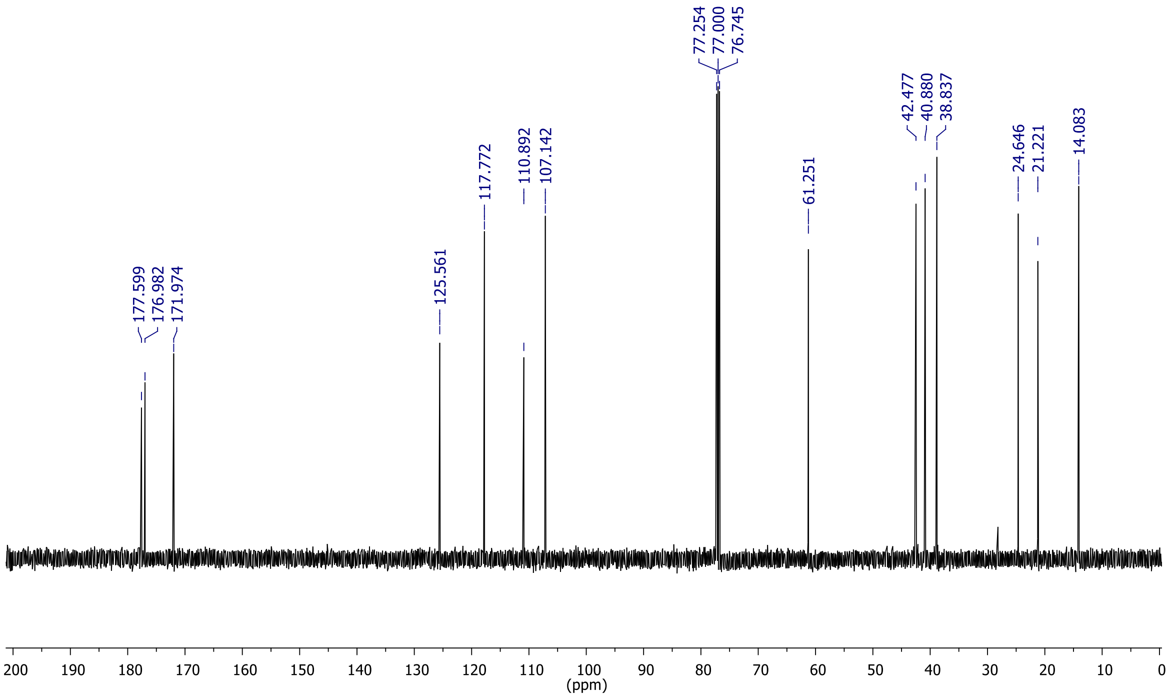

^{1}H NMR (600 MHz, $CDCl_3$) of compound **9c**

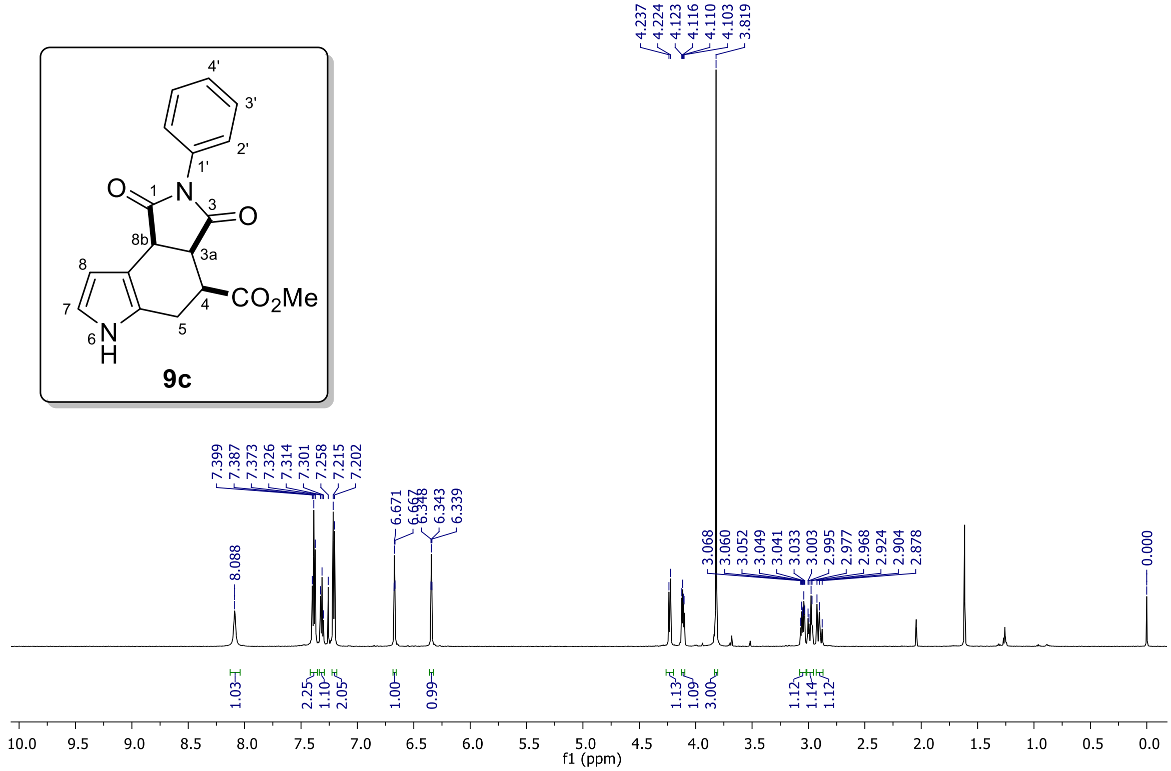

^{13}C NMR (150 MHz, $CDCl_3$) of compound **9c**.

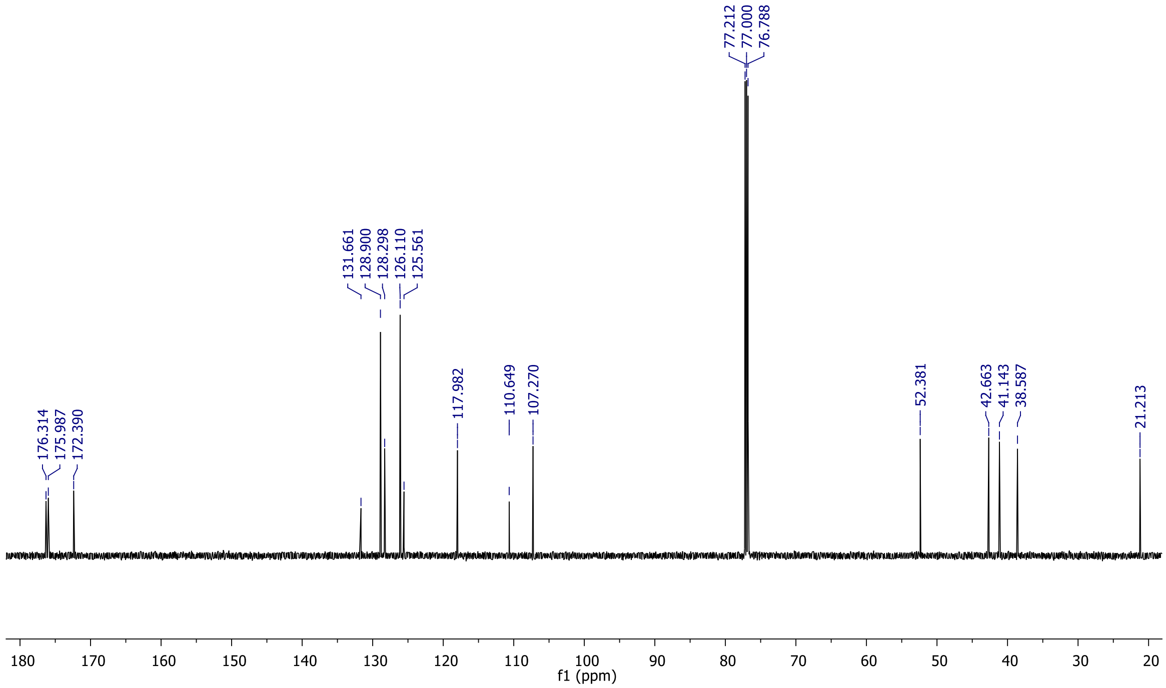

^{1}H NMR (500 MHz, $CDCl_3$) of compound **9d**.

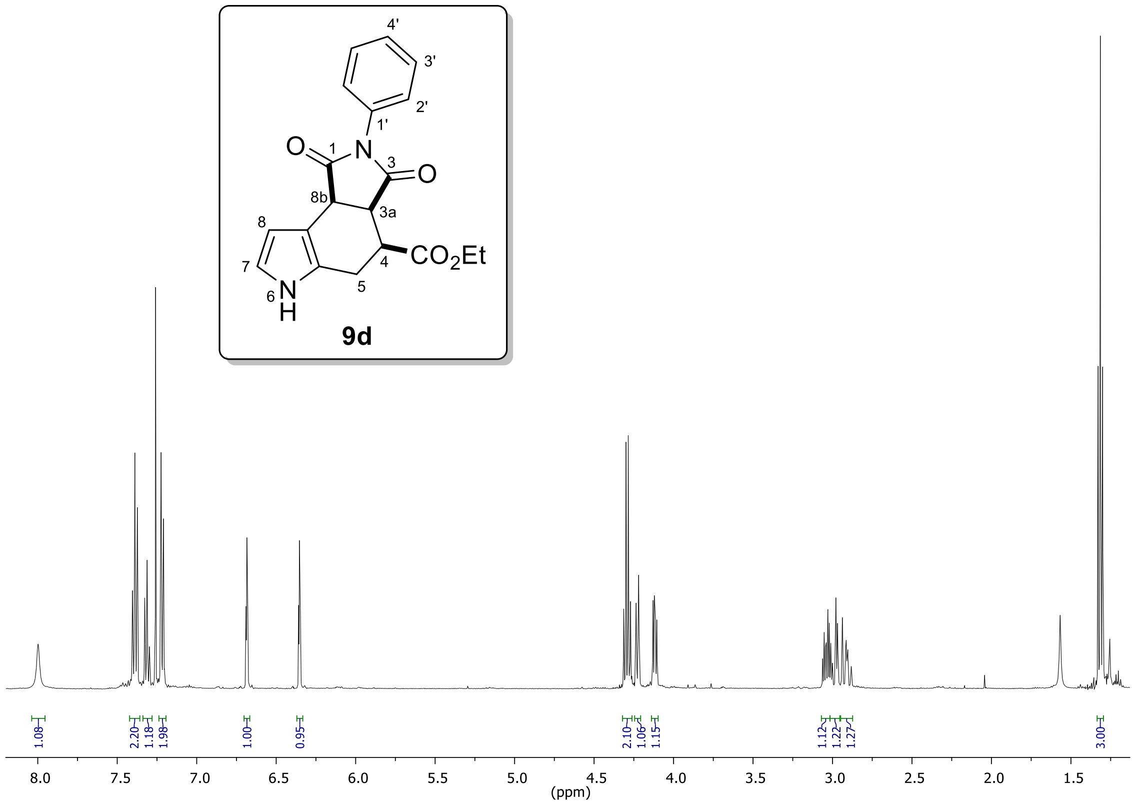

^{13}C NMR (125 MHz, $CDCl_3$) of compound **9d**.

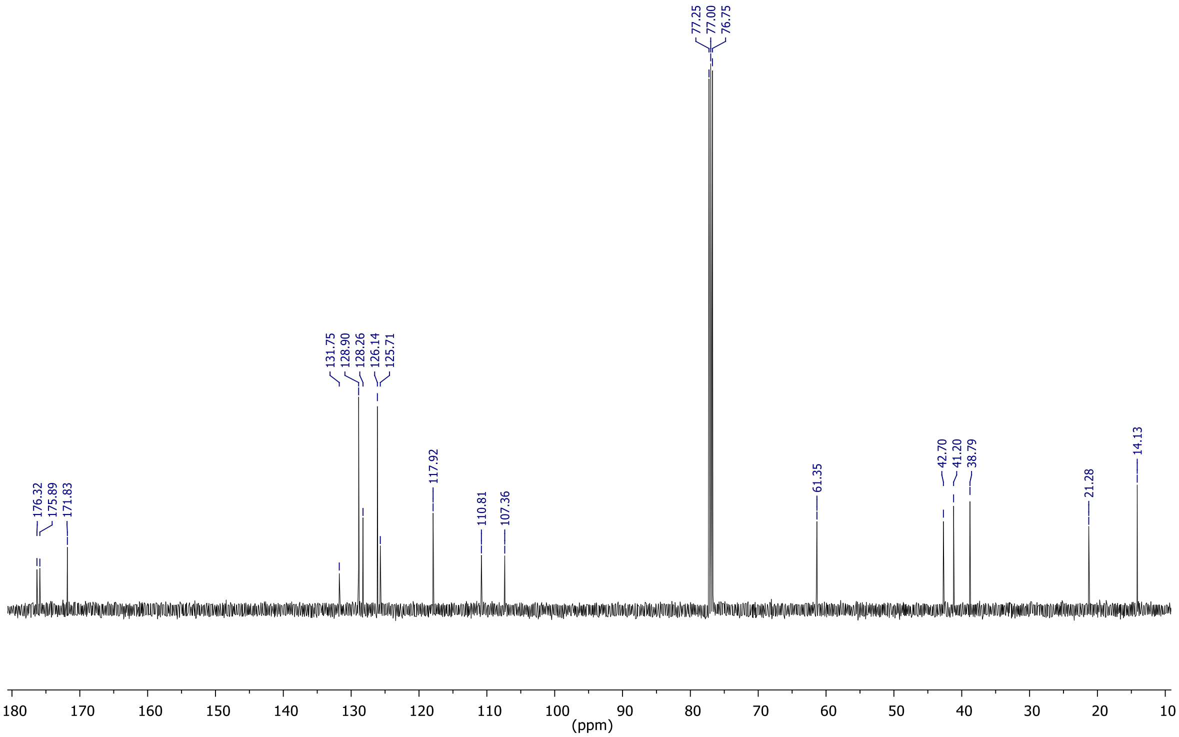

^{1}H NMR (500 MHz, $CDCl_3$) of compound **9e**.

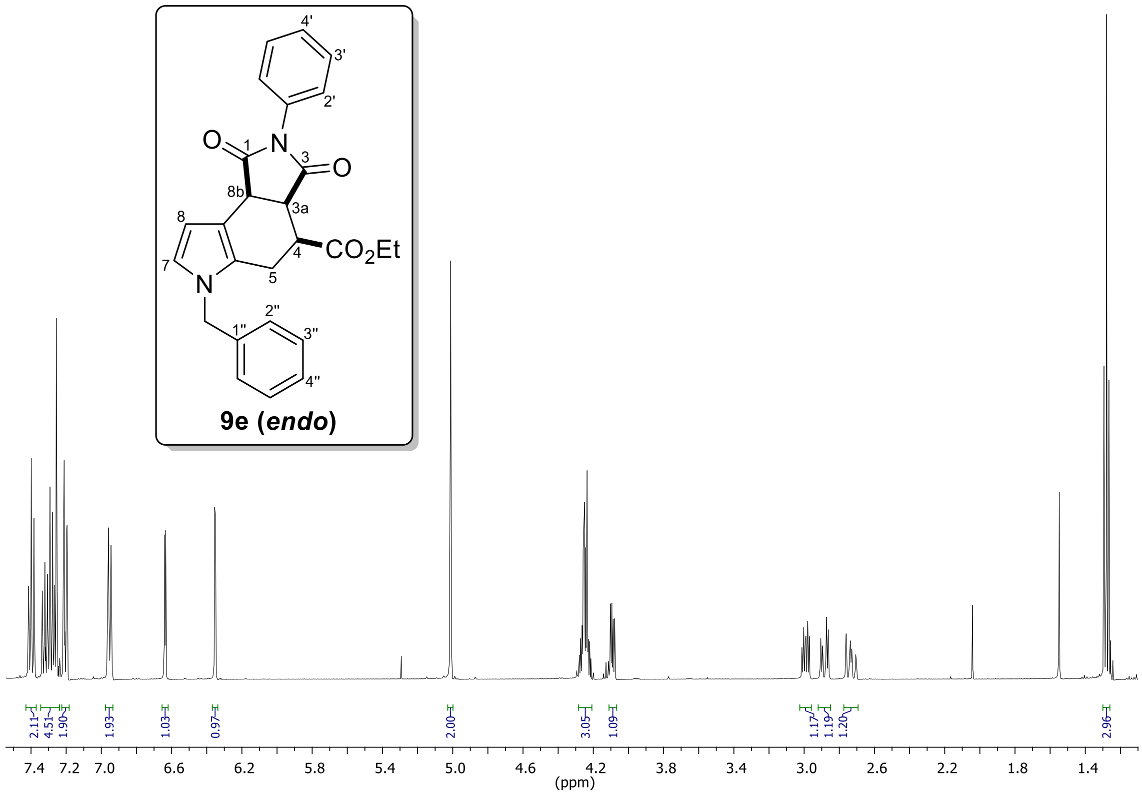

^{13}C NMR (125 MHz, $CDCl_3$) of compound **9e**.

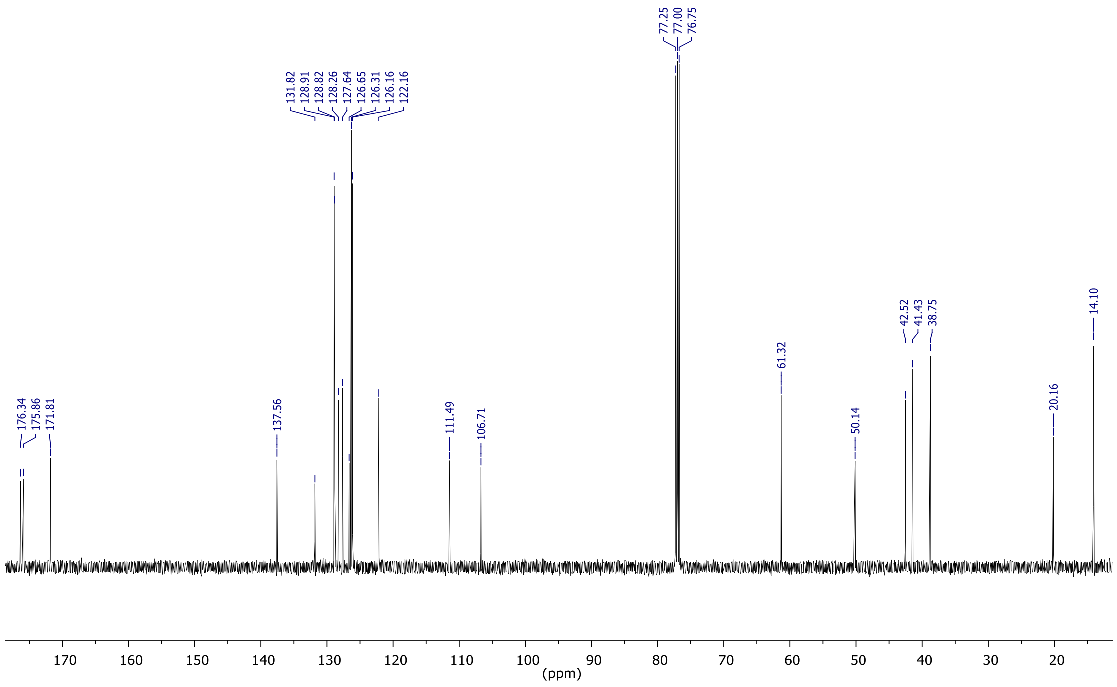

^{1}H NMR (500 MHz, $CDCl_3$) of compound **9f**.

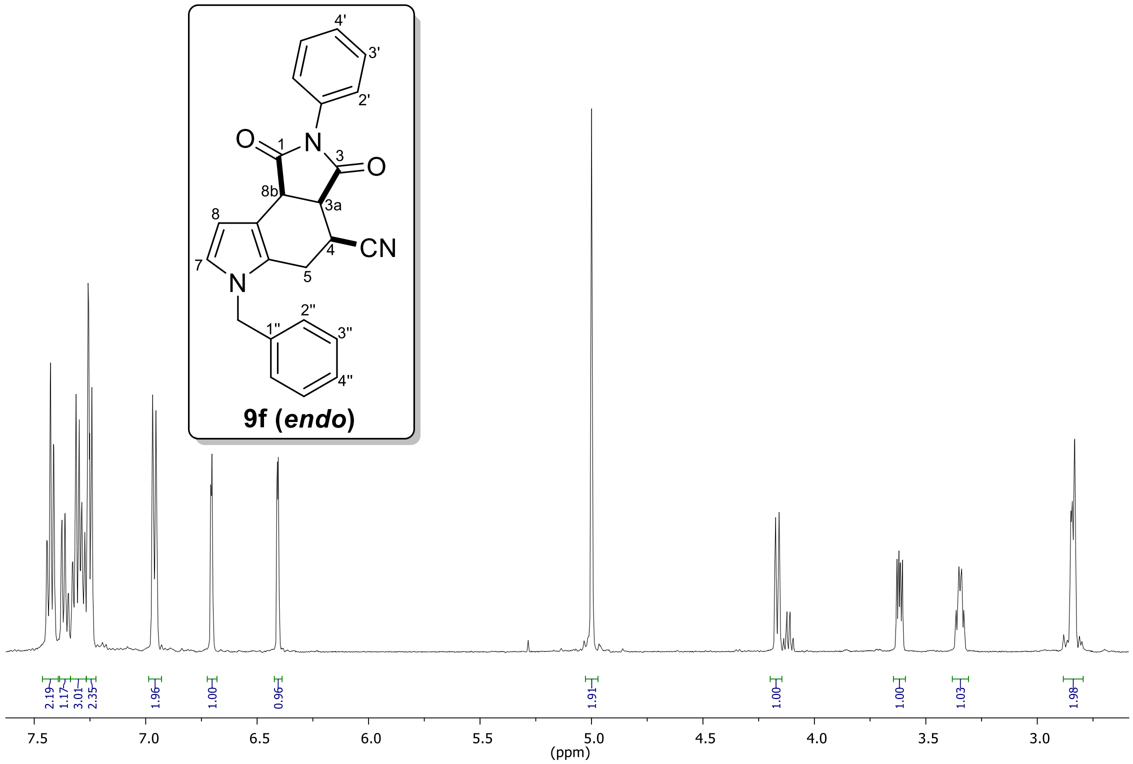

^{13}C NMR (125 MHz, $CDCl_3$) of compound **9f**.

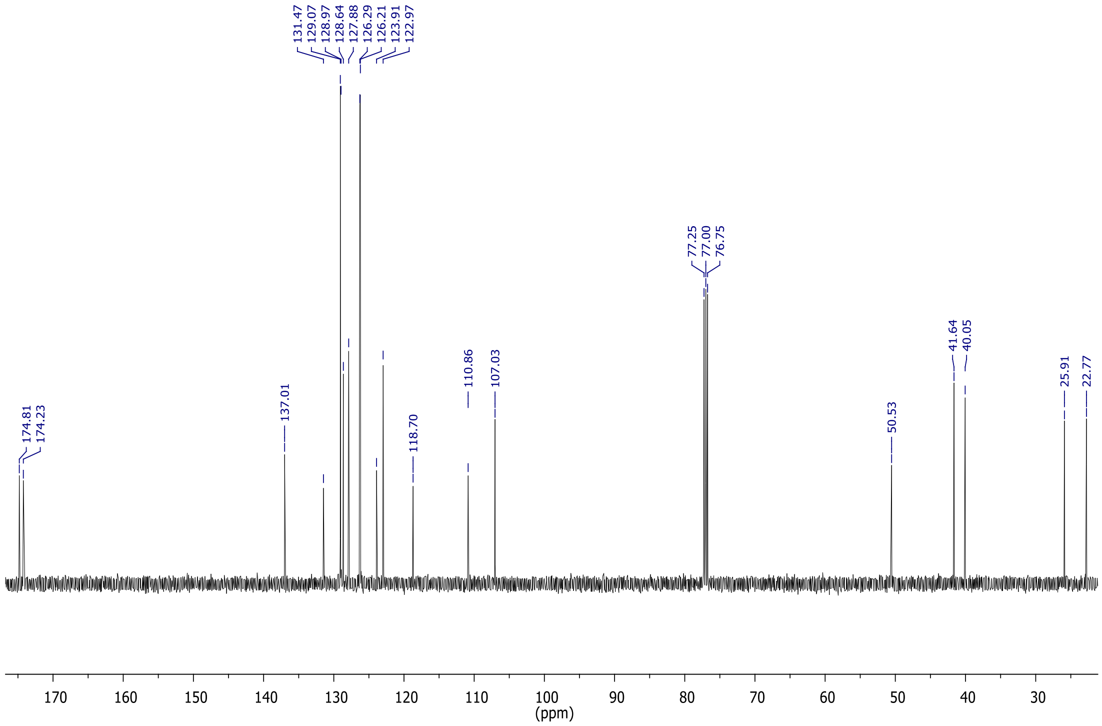

^{1}H NMR (500 MHz, $CDCl_3$) of compound **10f**.

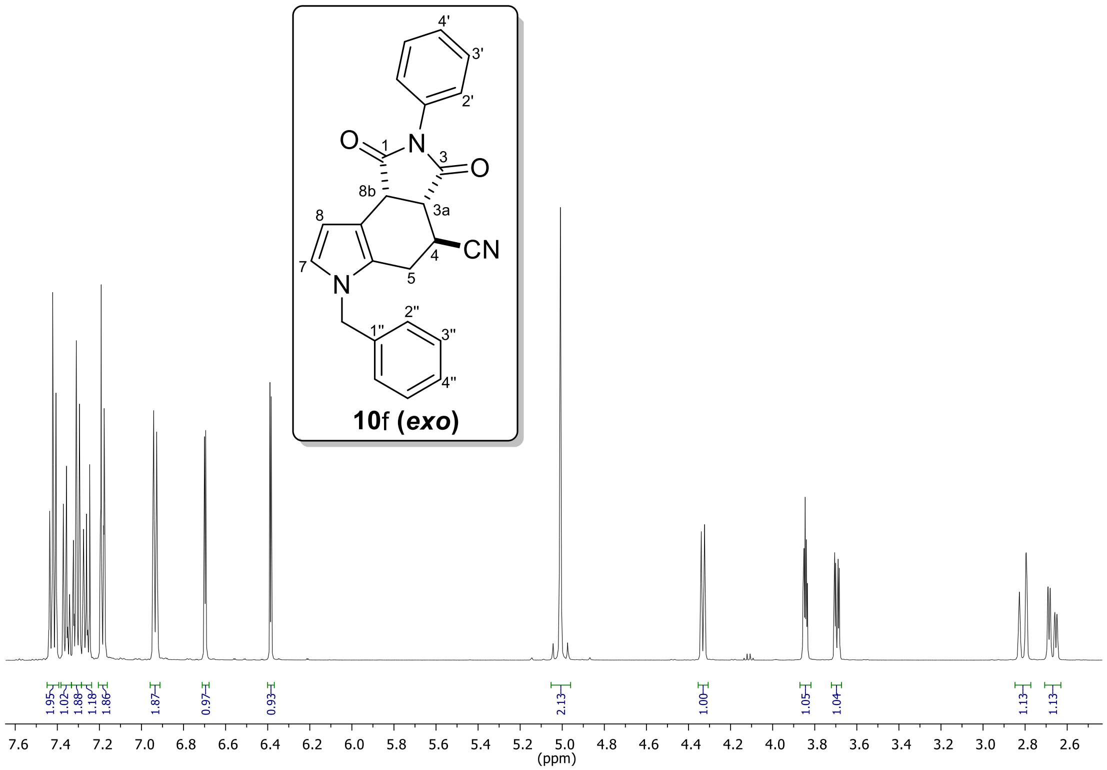

^{13}C NMR (125 MHz, $CDCl_3$) of compound **10f**.

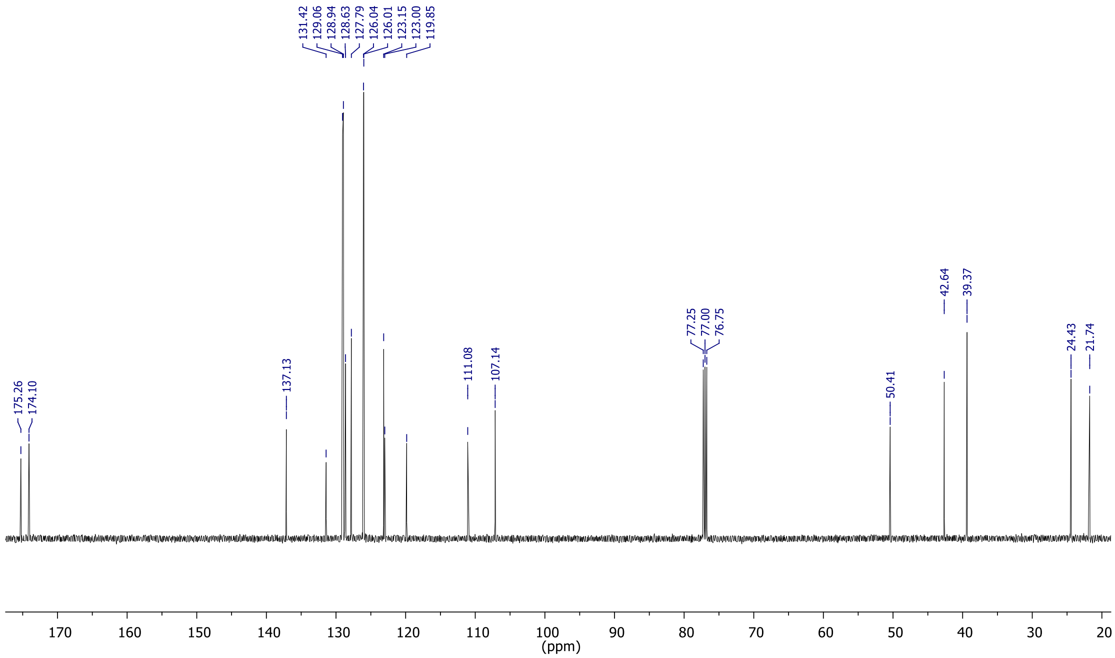

^{1}H NMR (600 MHz, $CDCl_3$) of compound **9g**.

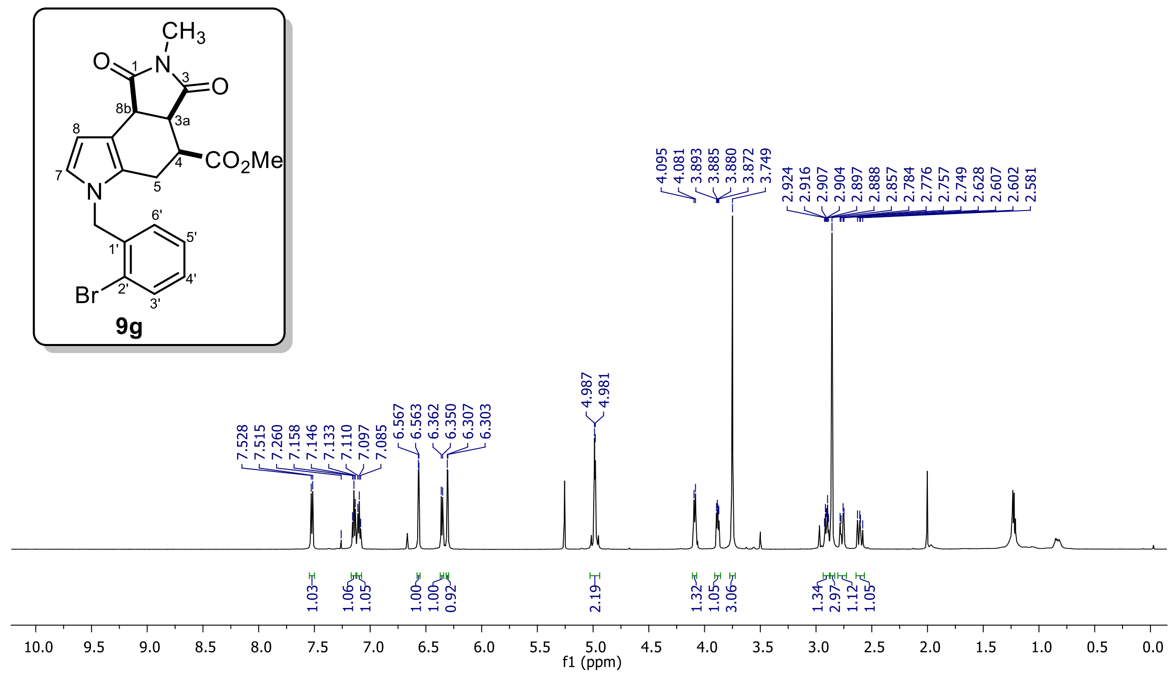

^{13}C NMR (150 MHz, $CDCl_3$) of compound **9g**.

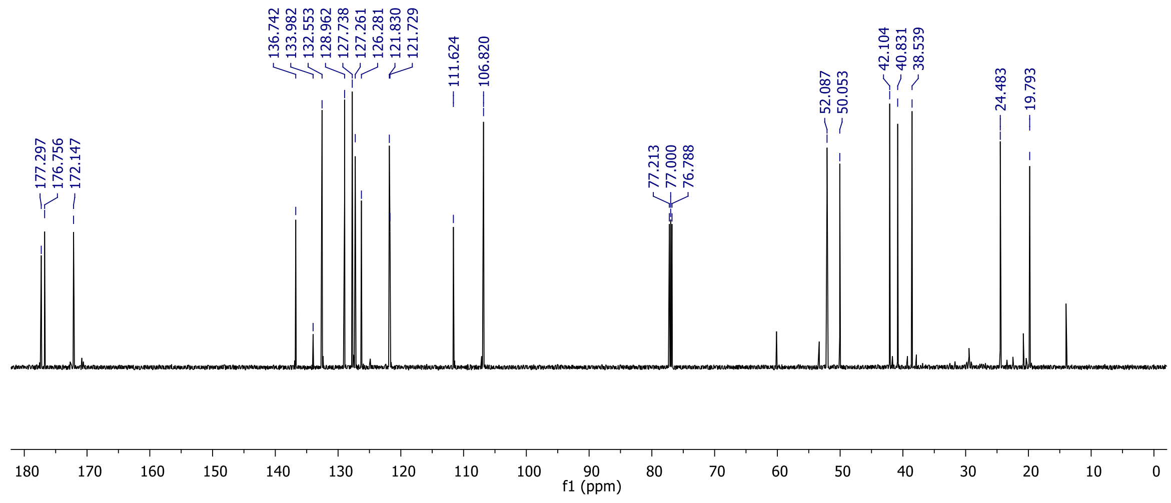

^{1}H NMR (300 MHz, $CDCl_3$) of compound **9h**.

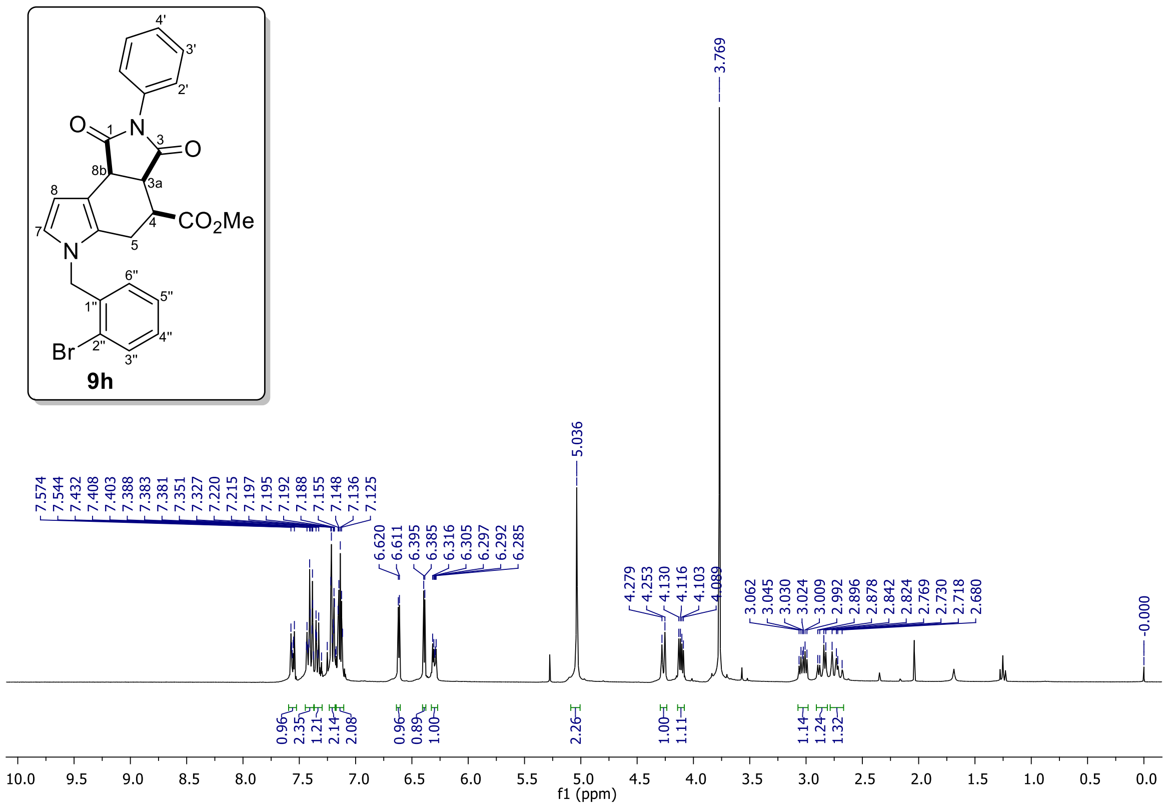

^{13}C NMR (74.5 MHz, $CDCl_3$) of compound **9h**.

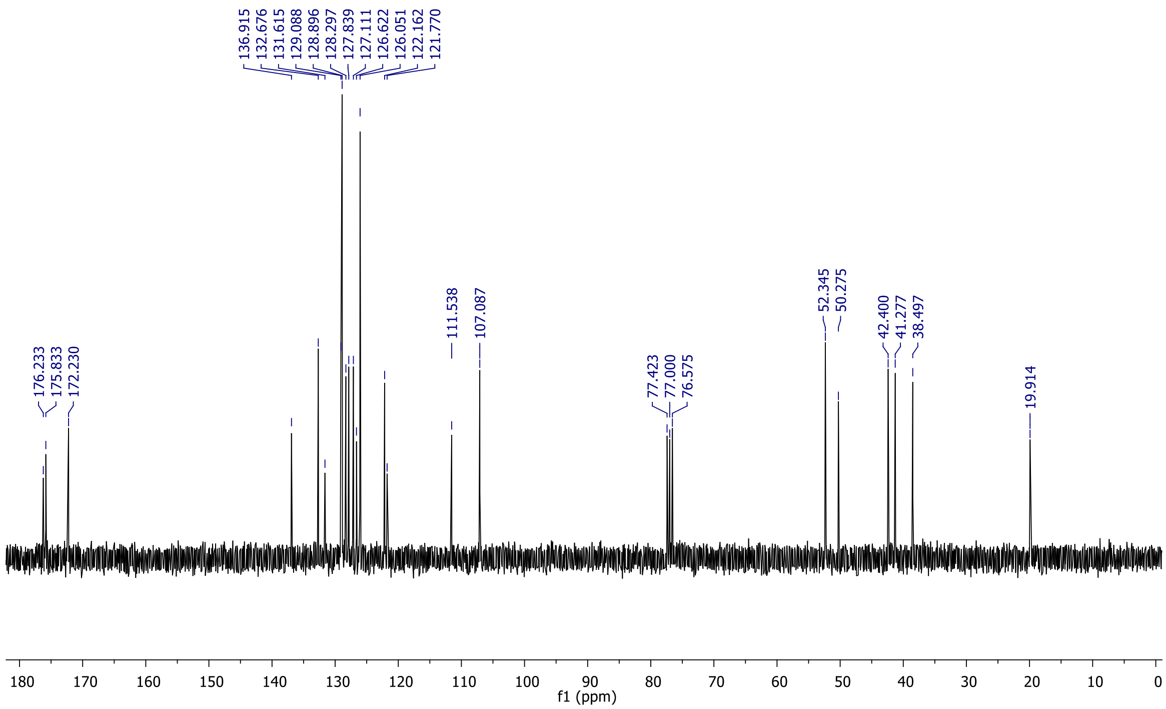

^{1}H NMR (500 MHz, $CDCl_3$) of compound **9i**.

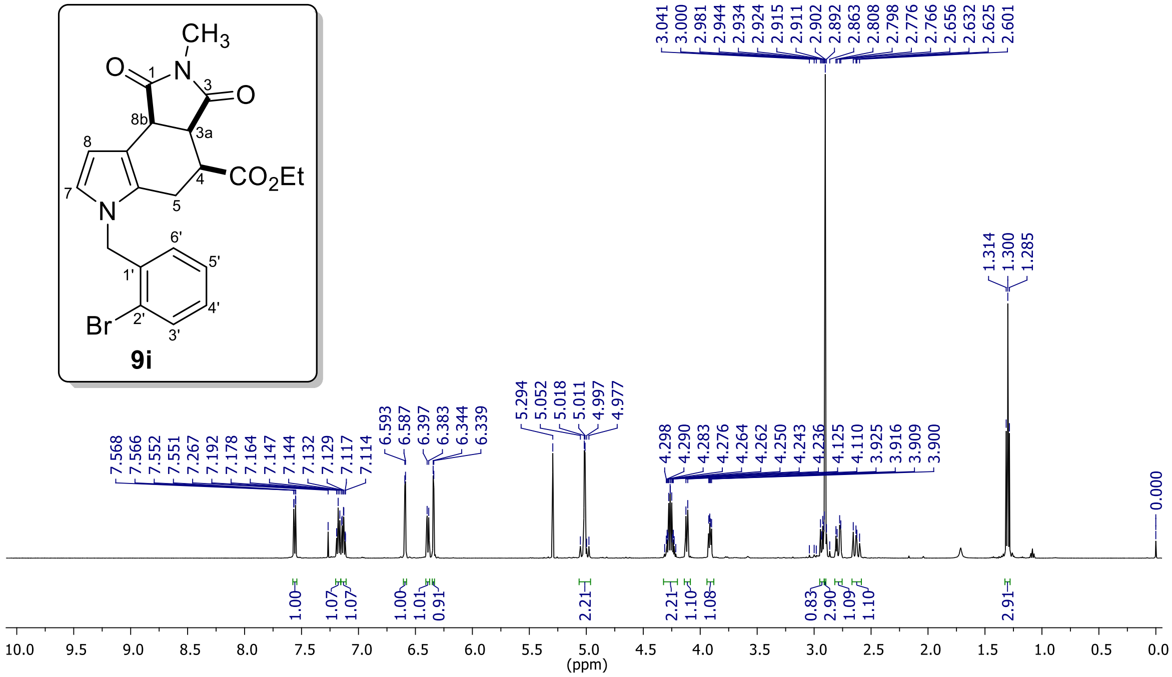

^{13}C NMR (125 MHz, $CDCl_3$) of compound **9i**.

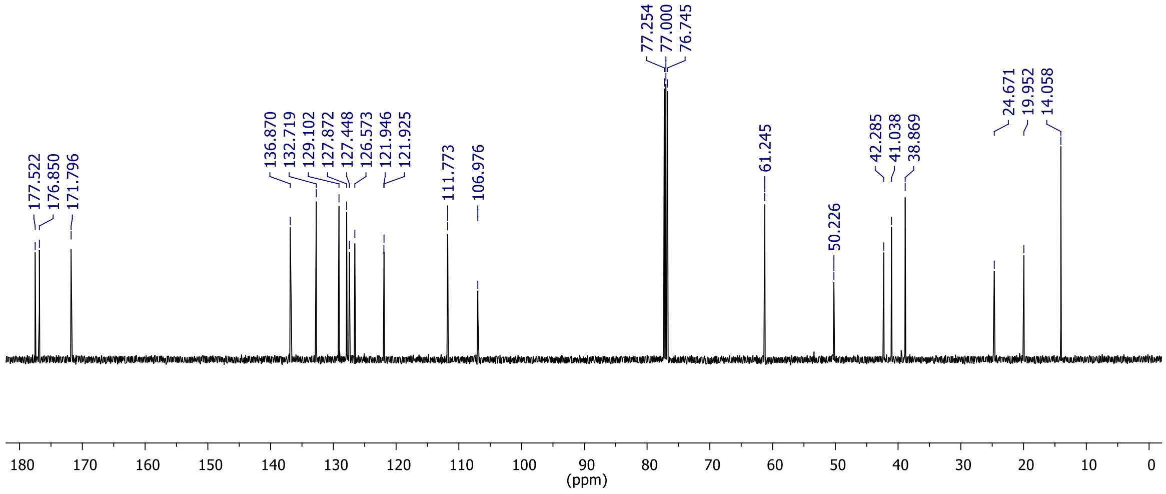

^{1}H NMR (500 MHz, $CDCl_3$) of compound **9j**.

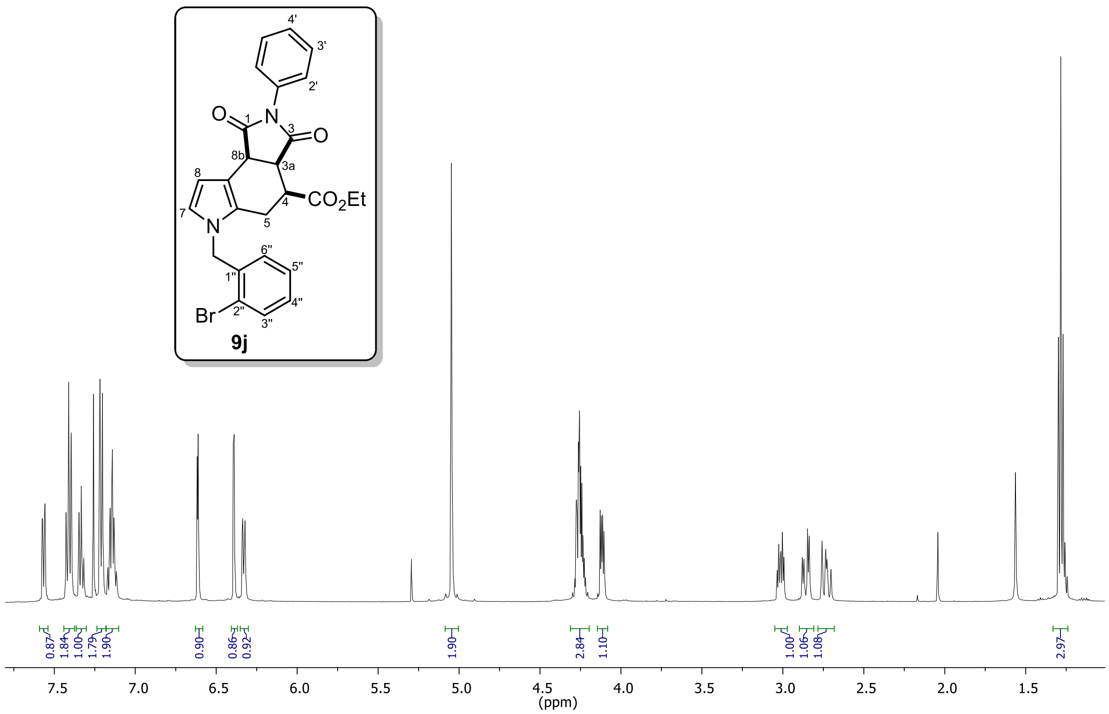

^{13}C NMR (125 MHz, $CDCl_3$) of compound **9j**.

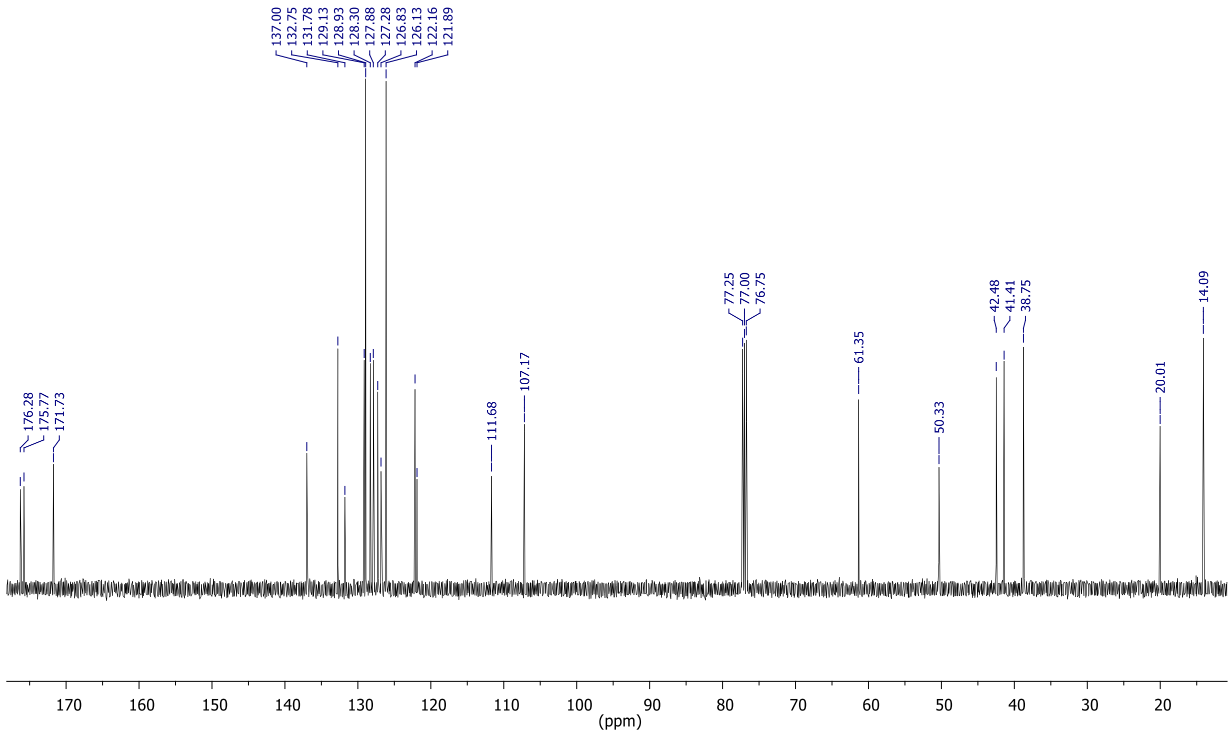

^{1}H NMR (500 MHz, $CDCl_3$) of compound **9k**.

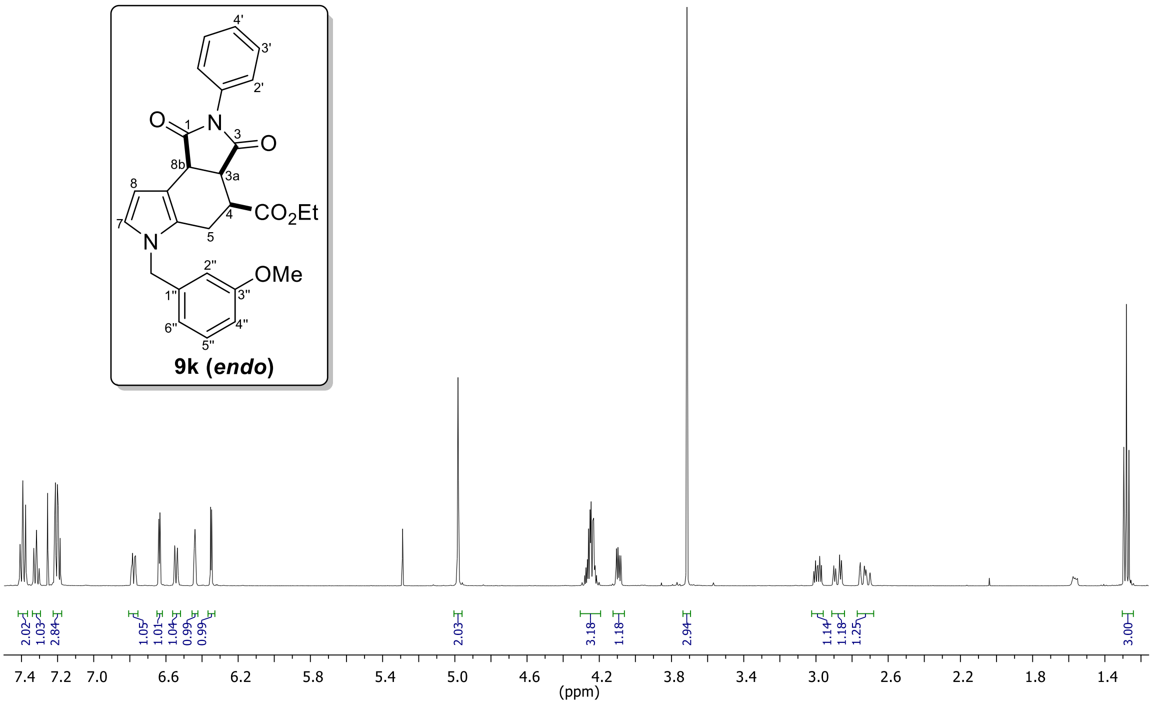

^{13}C NMR (125 MHz, $CDCl_3$) of compound **9k**.

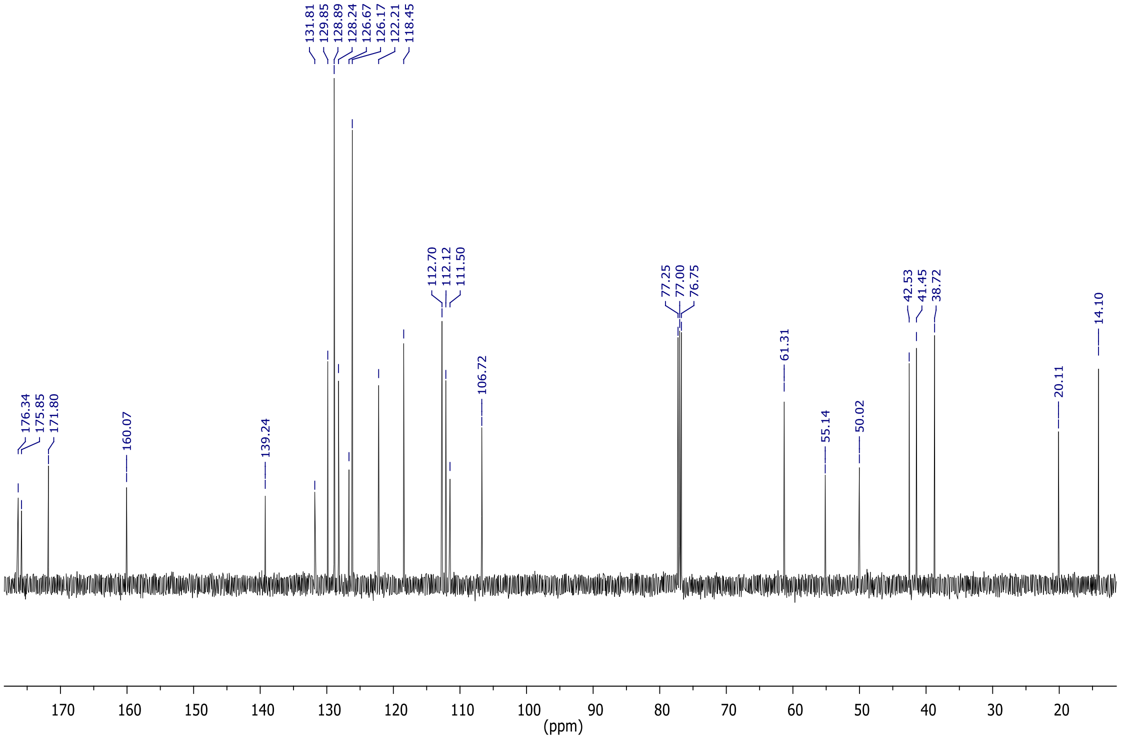

^{1}H NMR (500 MHz, $CDCl_3$) of compound **9l**.

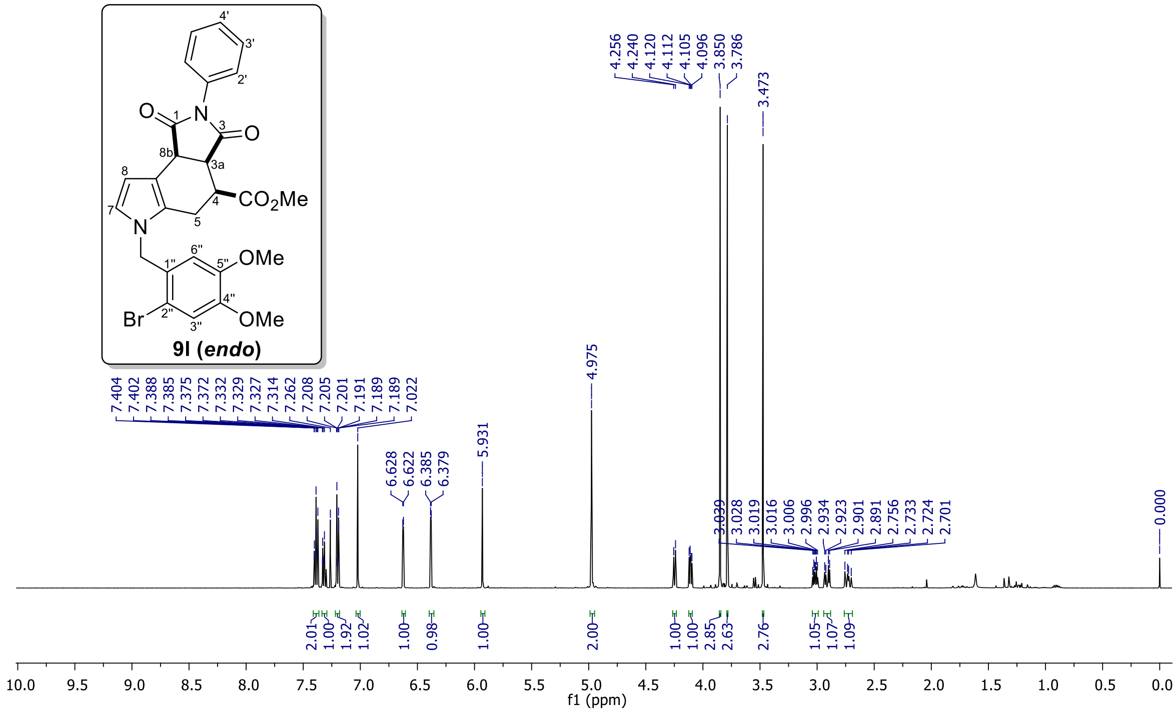

^{13}C NMR (125 MHz, $CDCl_3$) of compound **9l**.

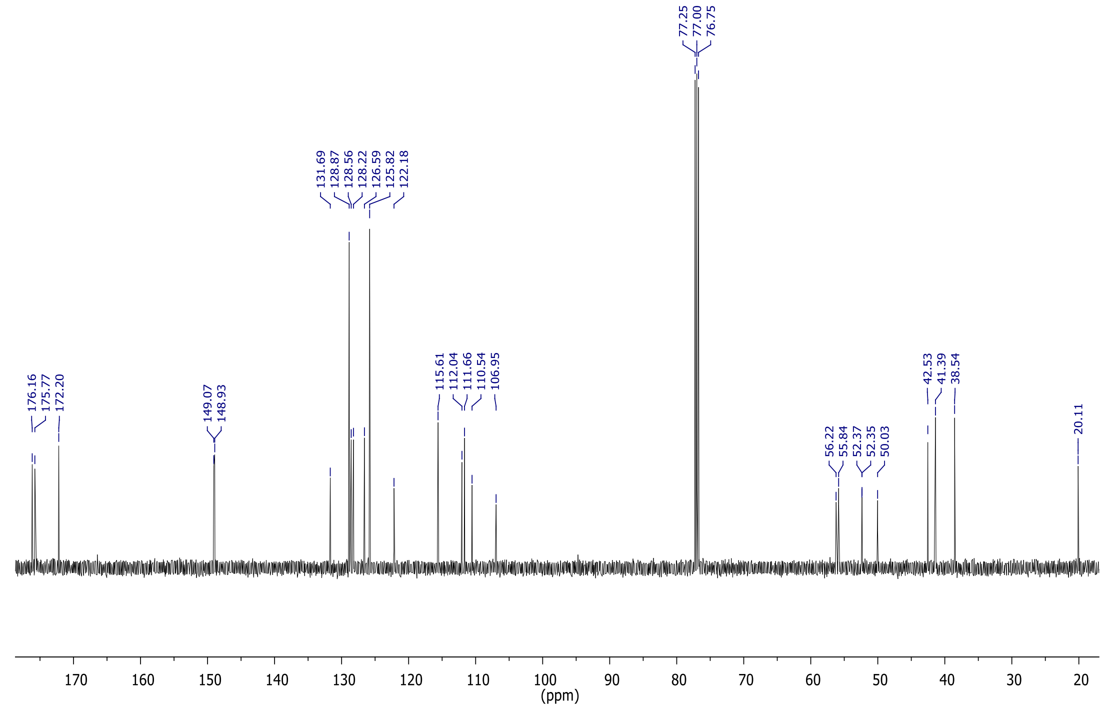

^{1}H NMR (500 MHz, $CDCl_3$) of compound **10l**.

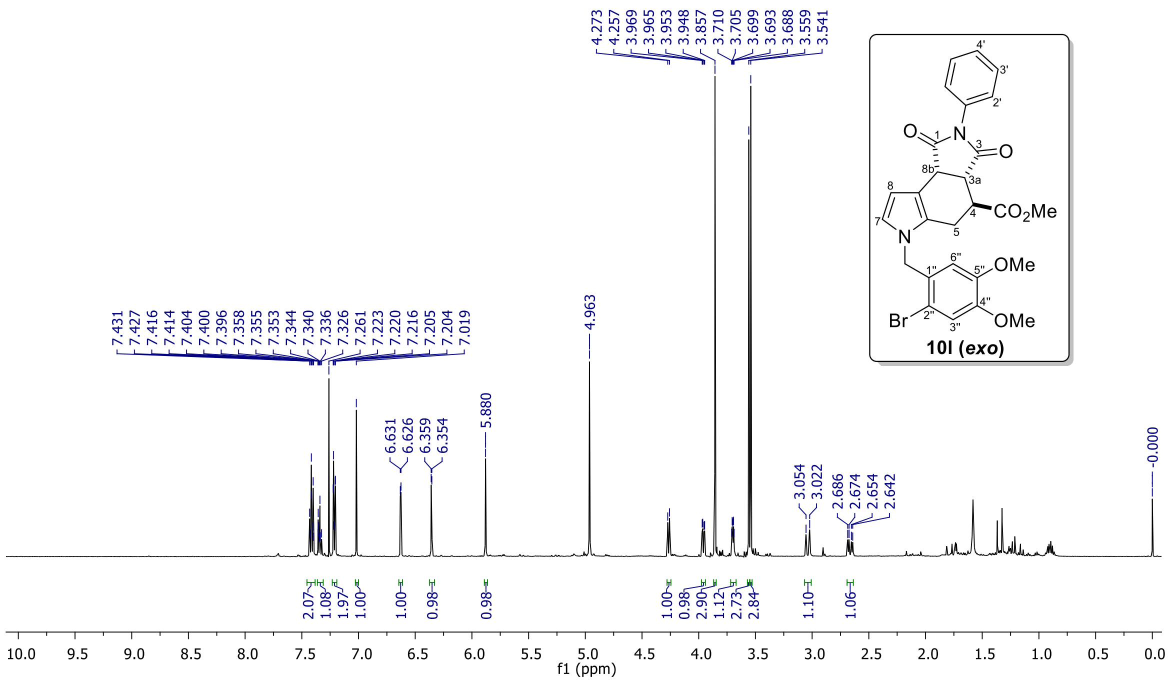

^{13}C NMR (125 MHz, $CDCl_3$) of compound **10l**.

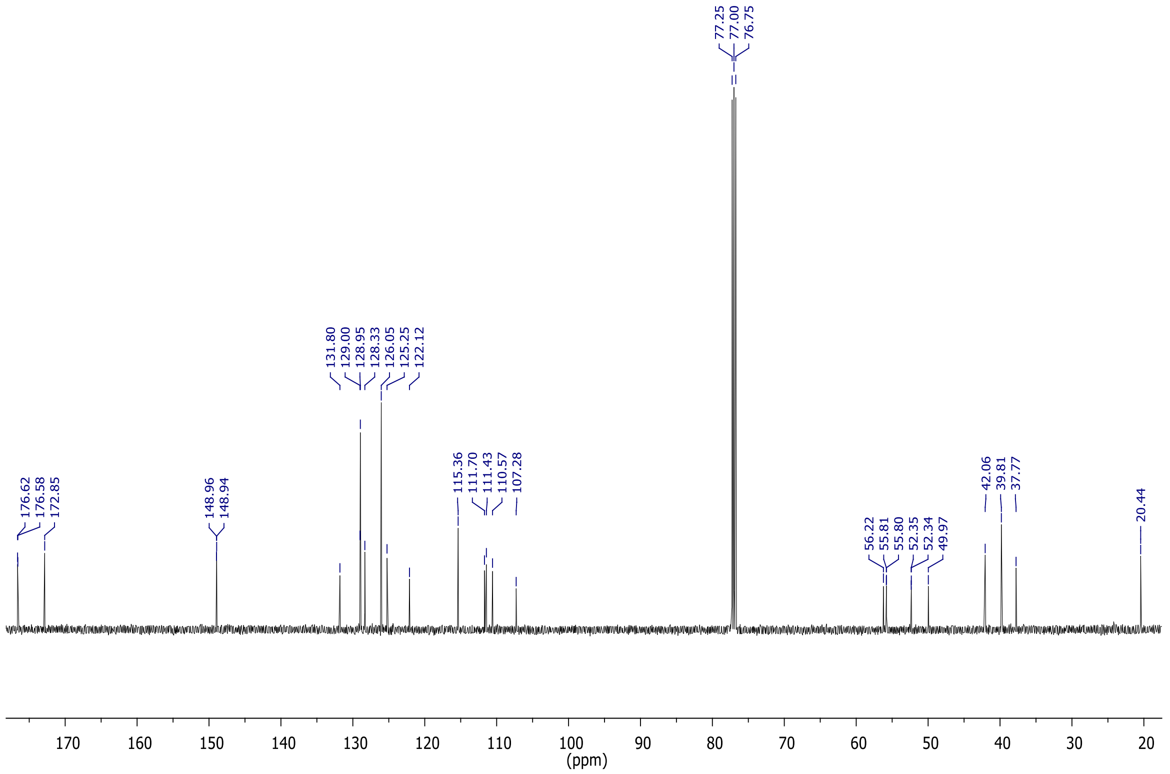

^{1}H NMR (500 MHz, $CDCl_3$) of compound **9m**.

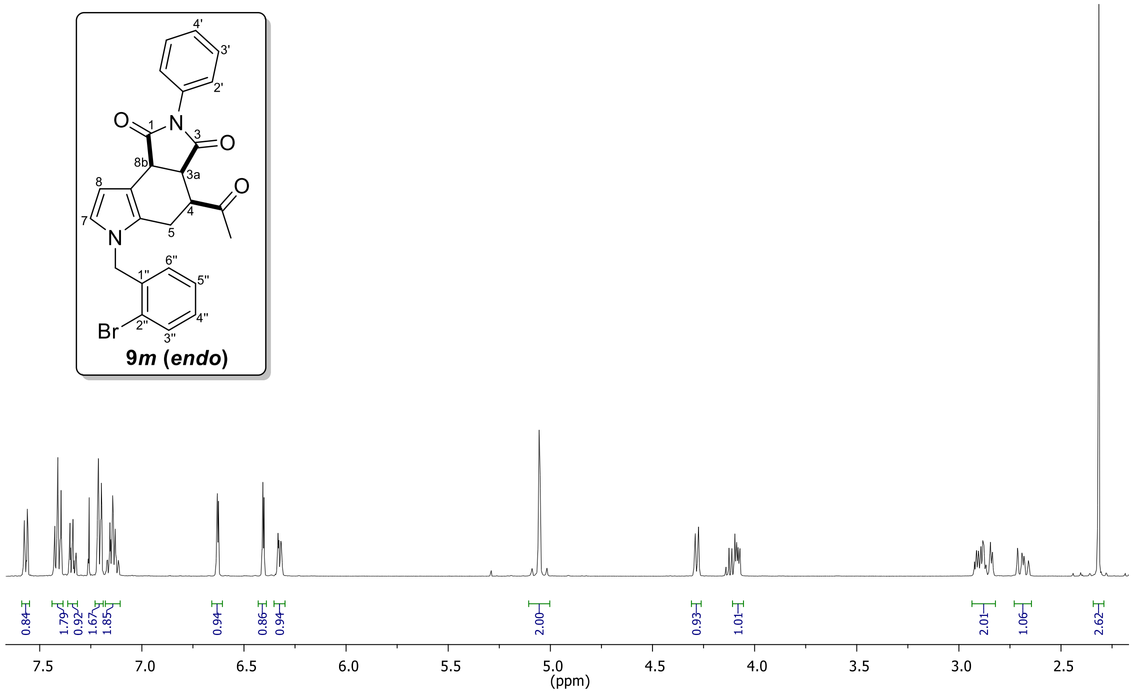

^{13}C NMR (125 MHz, $CDCl_3$) of compound **9m**.

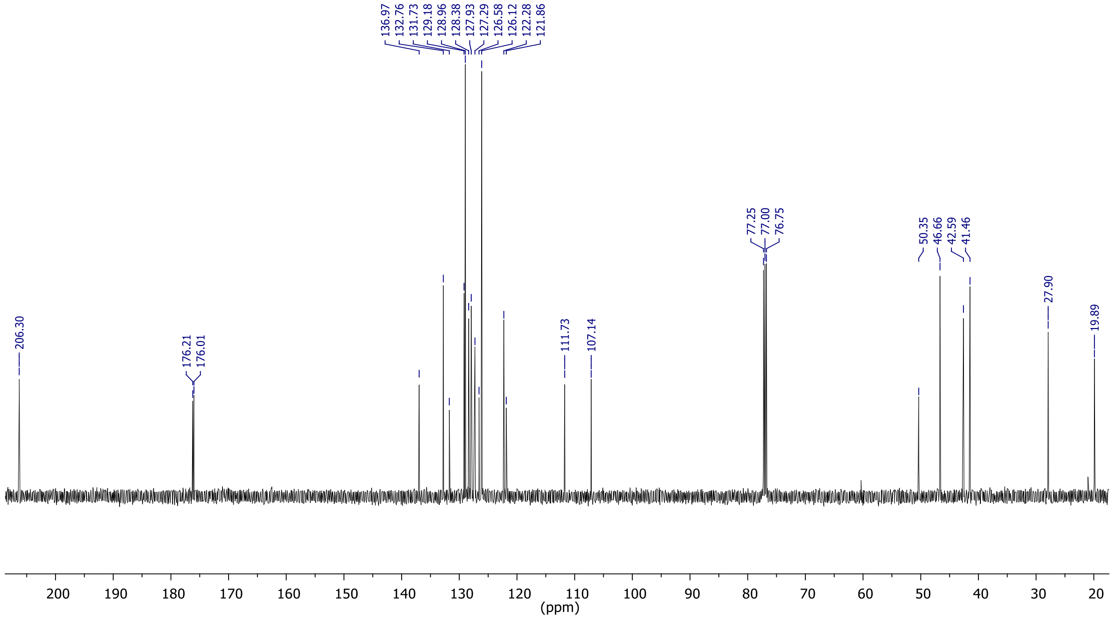

^{1}H NMR (500 MHz, $CDCl_3$) of compound **10m**.

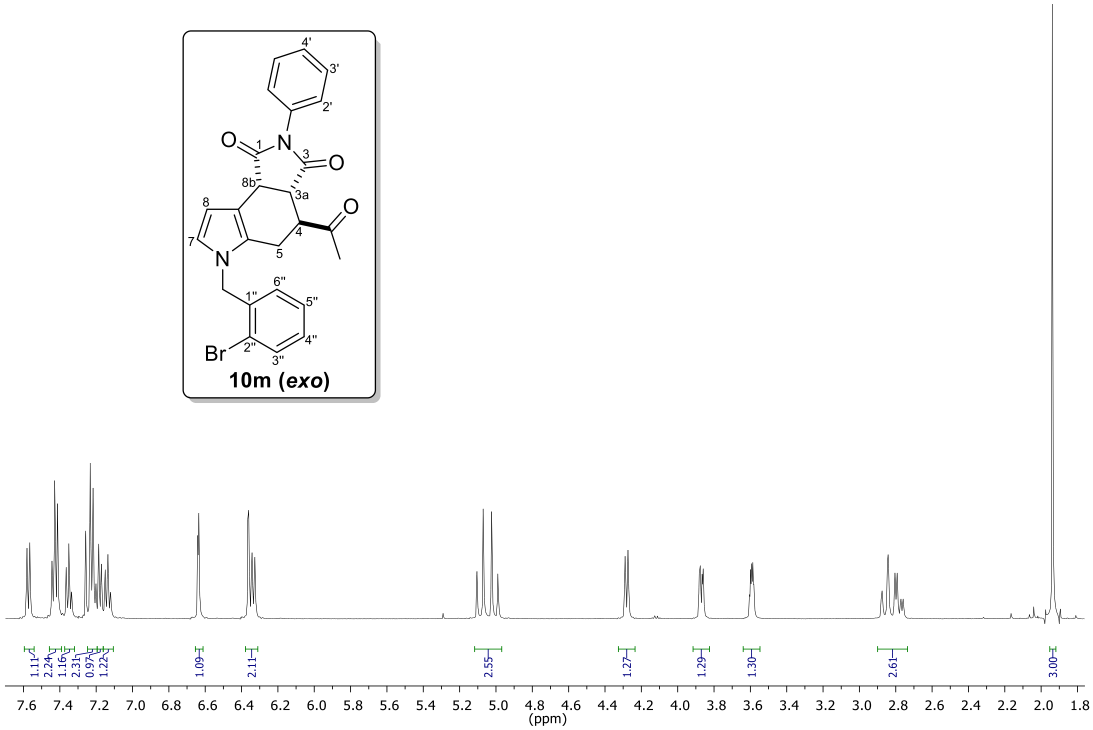

^{13}C NMR (125 MHz, $CDCl_3$) of compound **10m**.

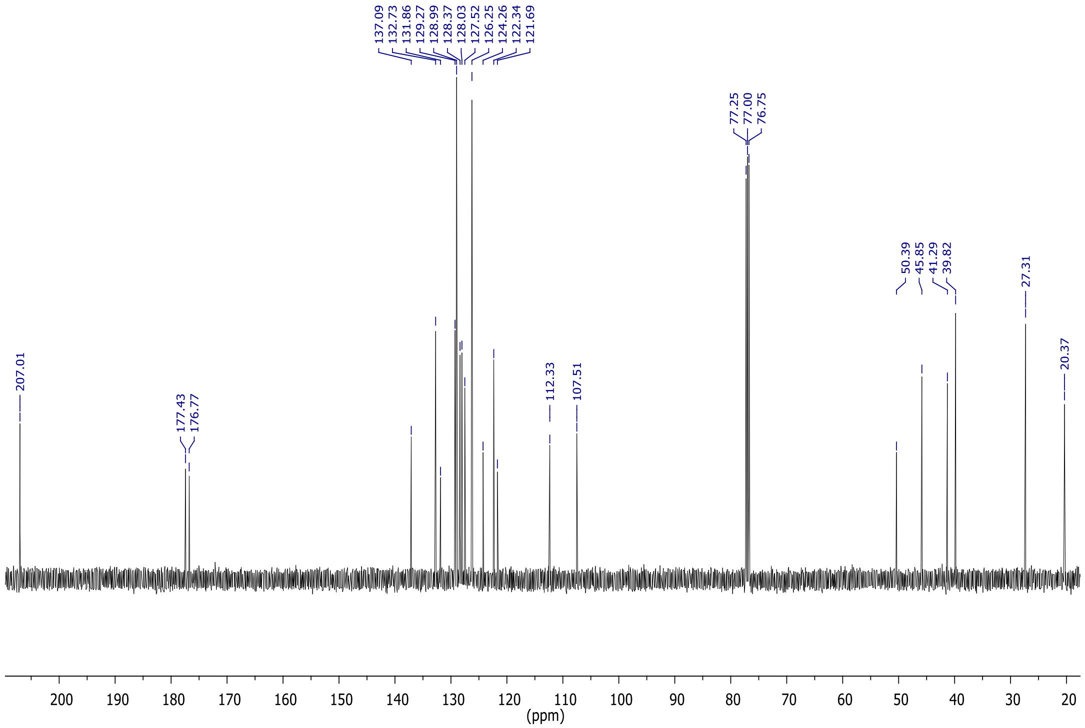

^{1}H NMR (500 MHz, $CDCl_3$) of compound **9n**.

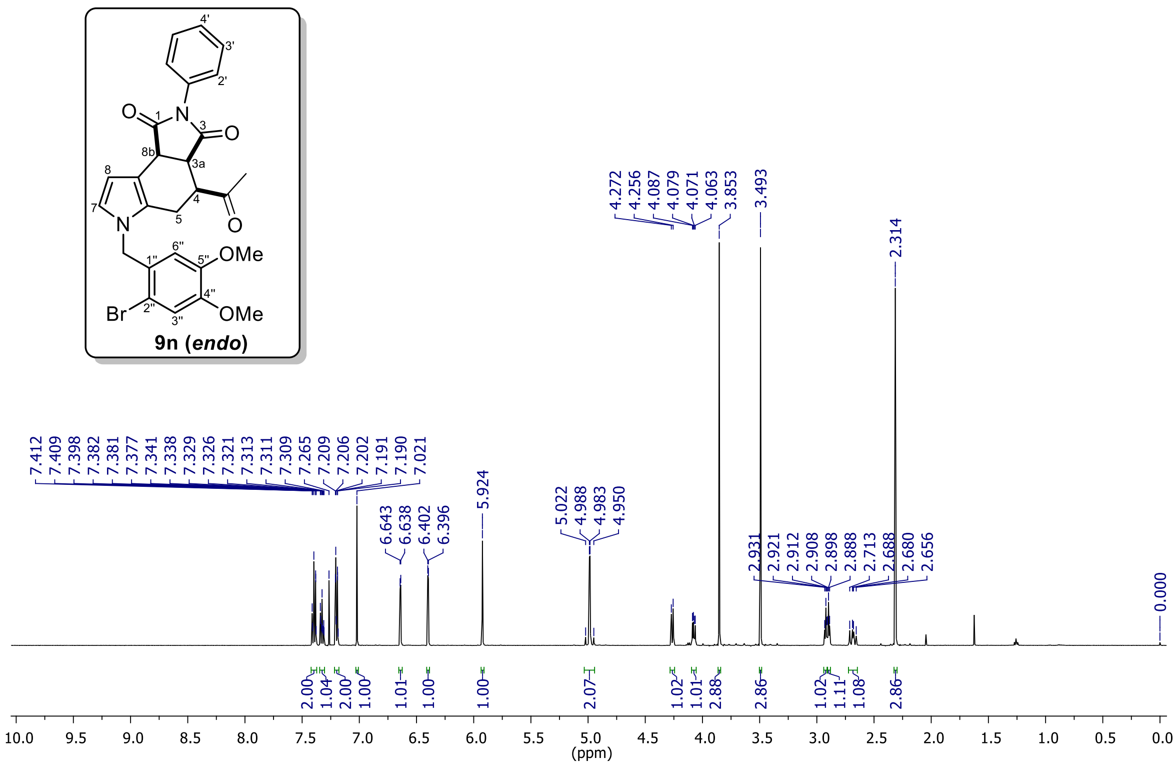

^{13}C NMR (125 MHz, $CDCl_3$) of compound **9n**.

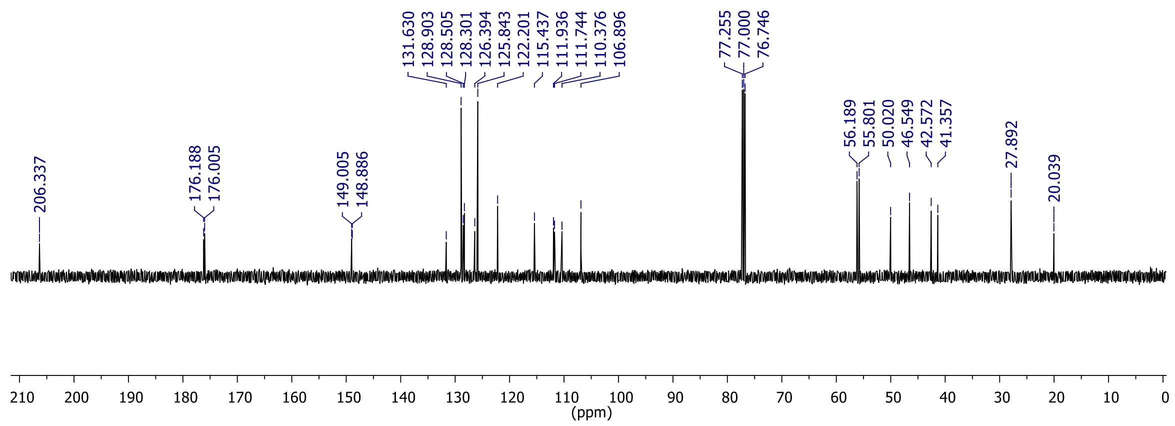

^{1}H NMR (500 MHz, $CDCl_3$) of compound **10n**.

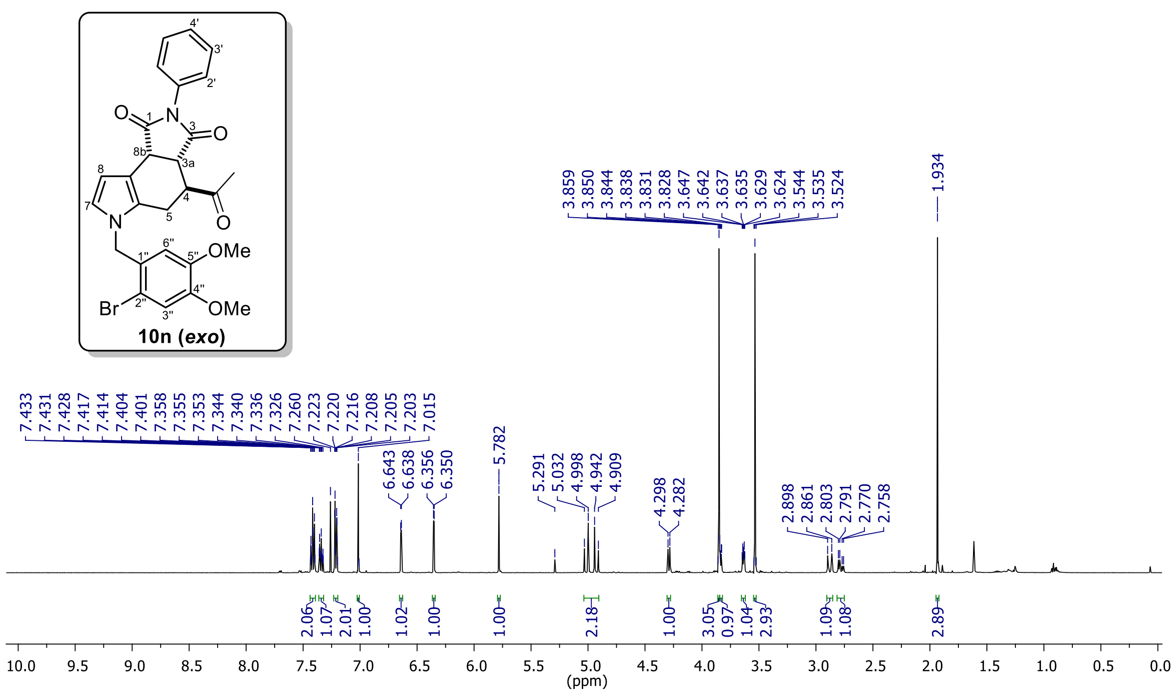

^{13}C NMR (125 MHz, $CDCl_3$) of compound **10n**.

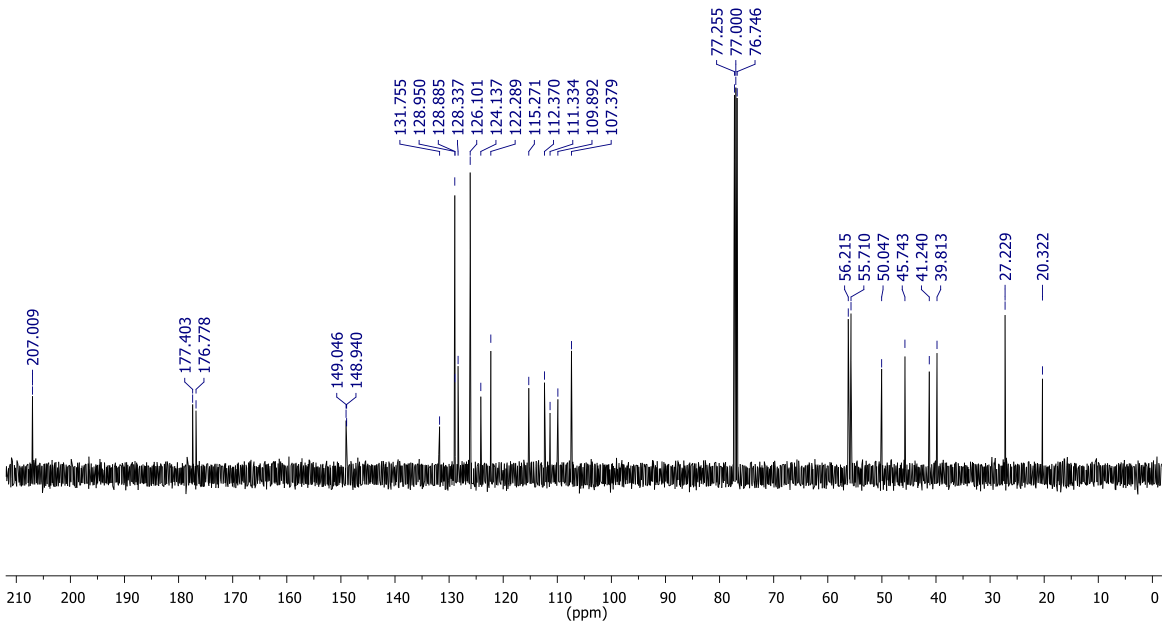

^{1}H NMR (300 MHz, $CDCl_3$) of compound **9o**.

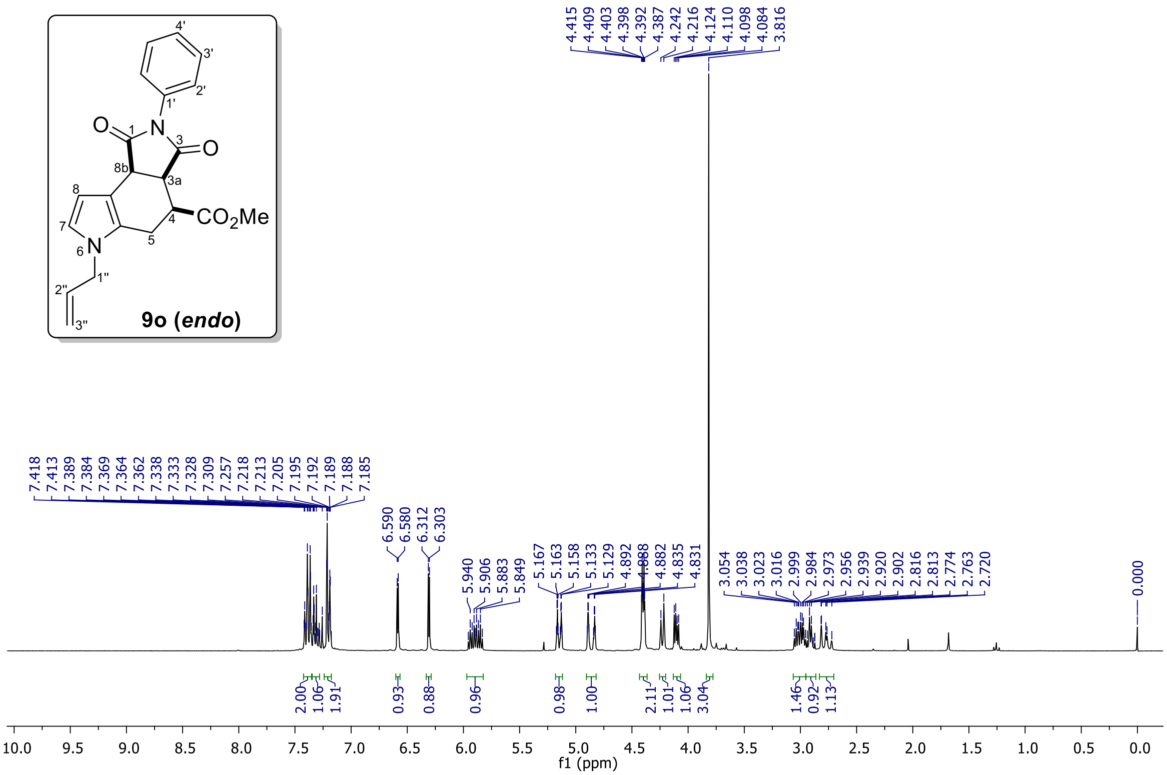

^{13}C NMR (75.4 MHz, $CDCl_3$) of compound **9o**.

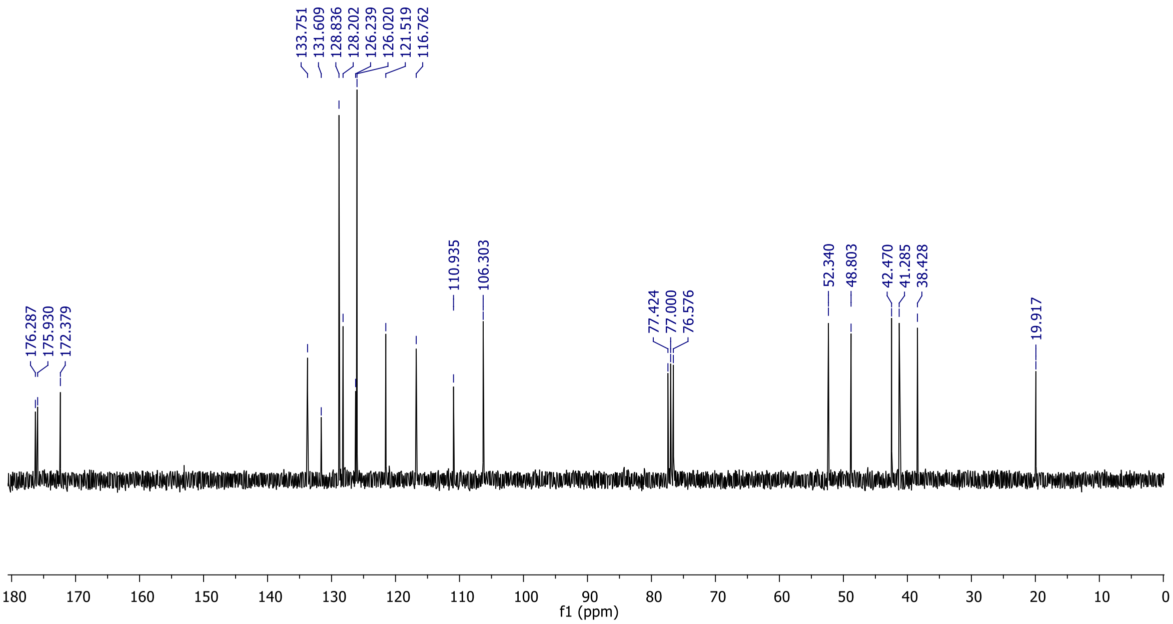

^{1}H NMR (300 MHz, $CDCl_3$) of compound **9p**.

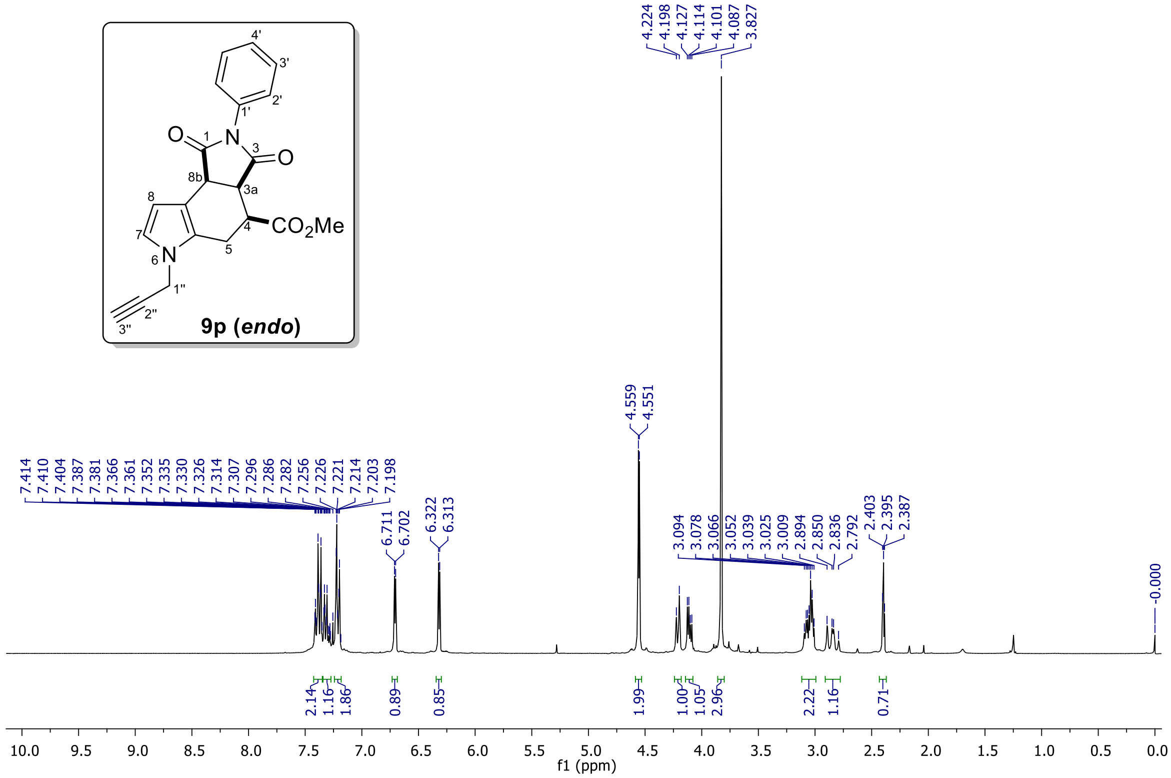

^{13}C NMR (75.4 MHz, $CDCl_3$) of compound **9p**.

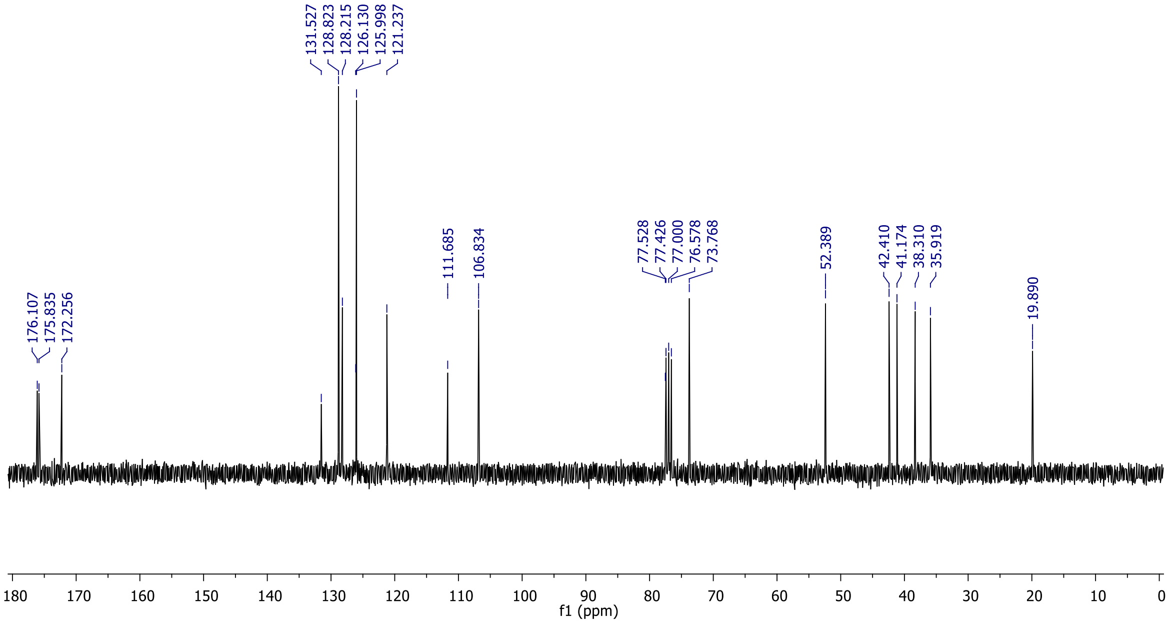

^{1}H NMR (300 MHz, $CDCl_3$) of compound **18a**.

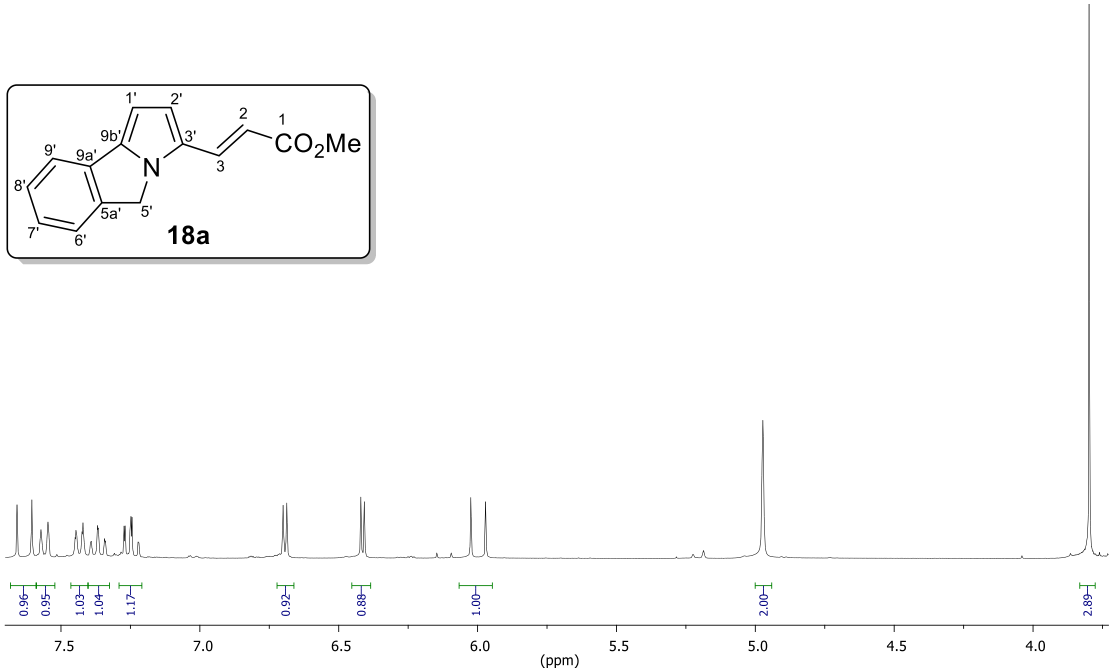

^{13}C NMR (75.4 MHz, $CDCl_3$) of compound **18a**.

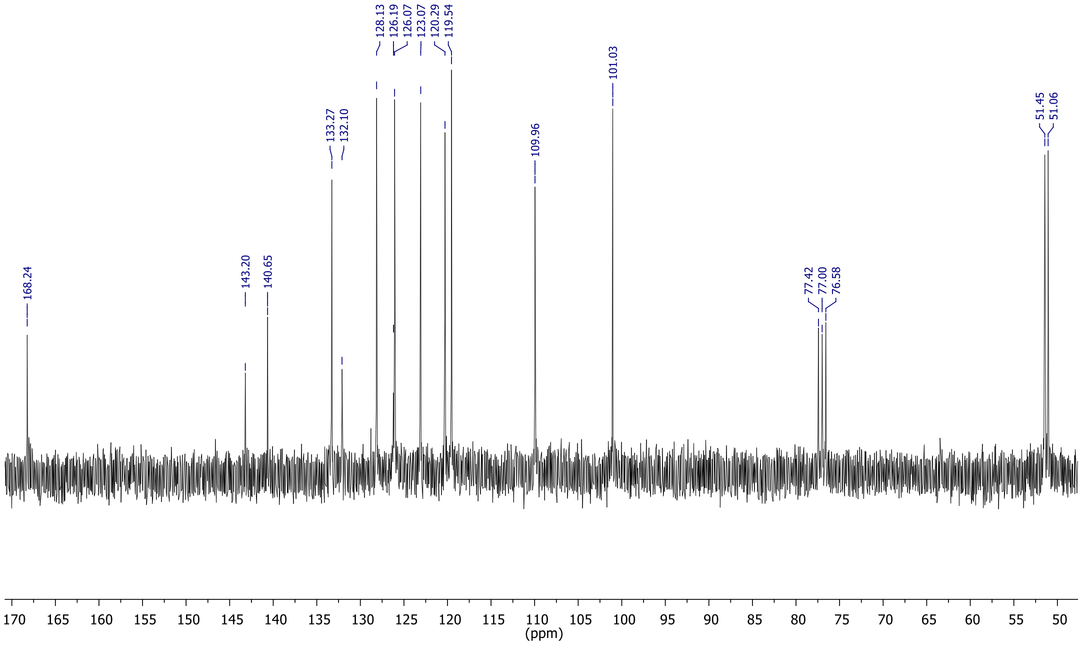

^{1}H NMR (600 MHz, $CDCl_3$) of compound **18b**.

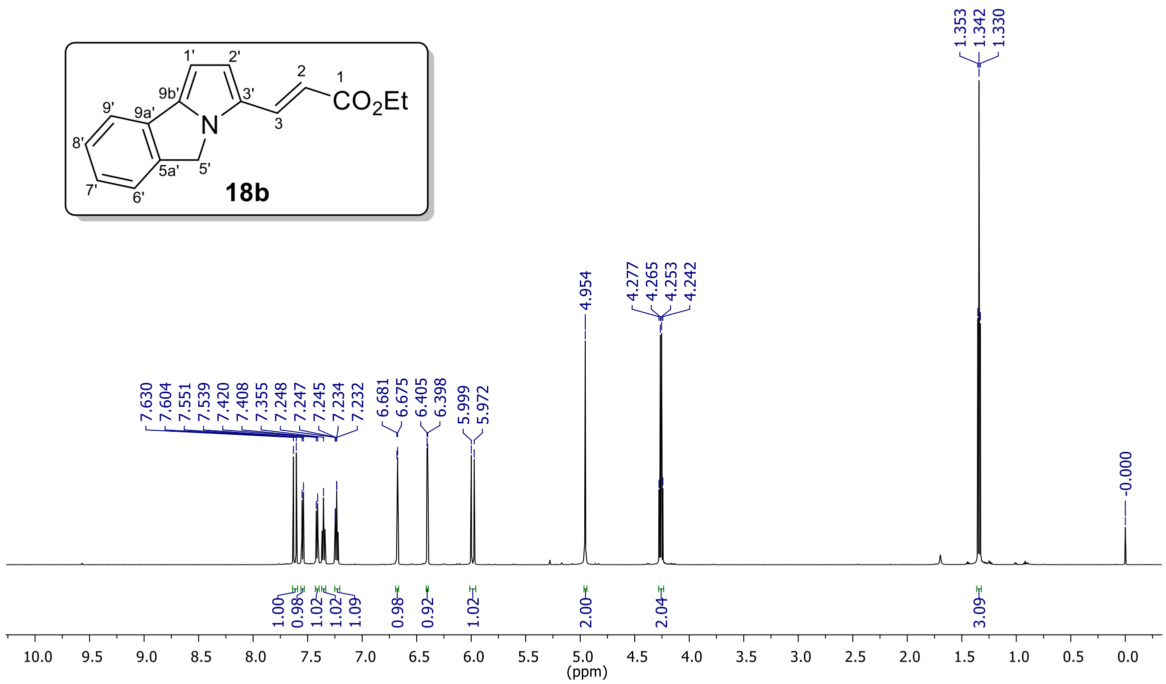

^{13}C NMR (150 MHz, $CDCl_3$) of compound **18b**.

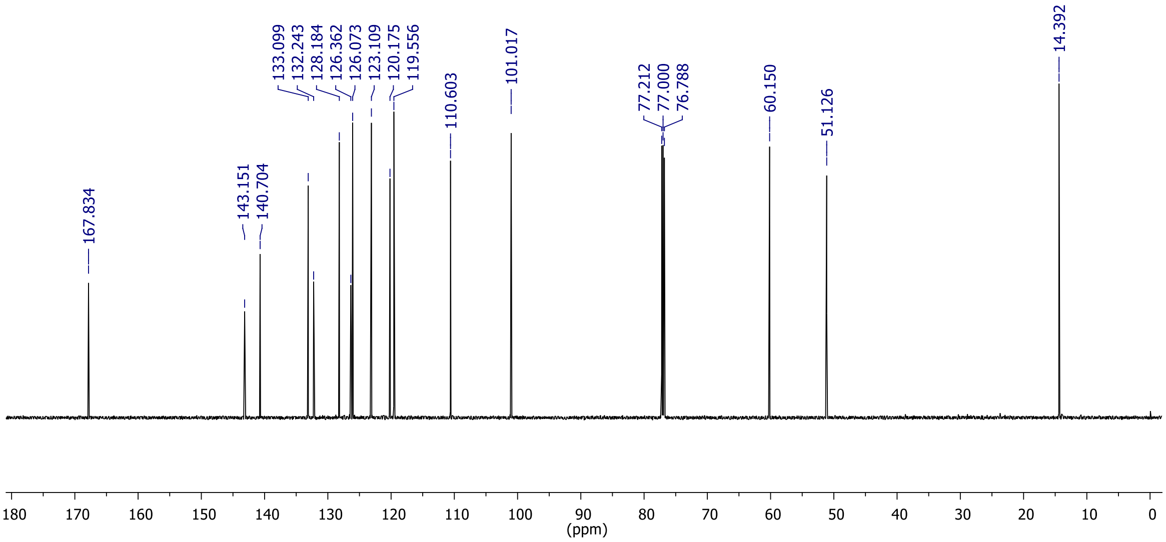

^{1}H NMR(300 MHz, $CDCl_3$) of compound **18c**.

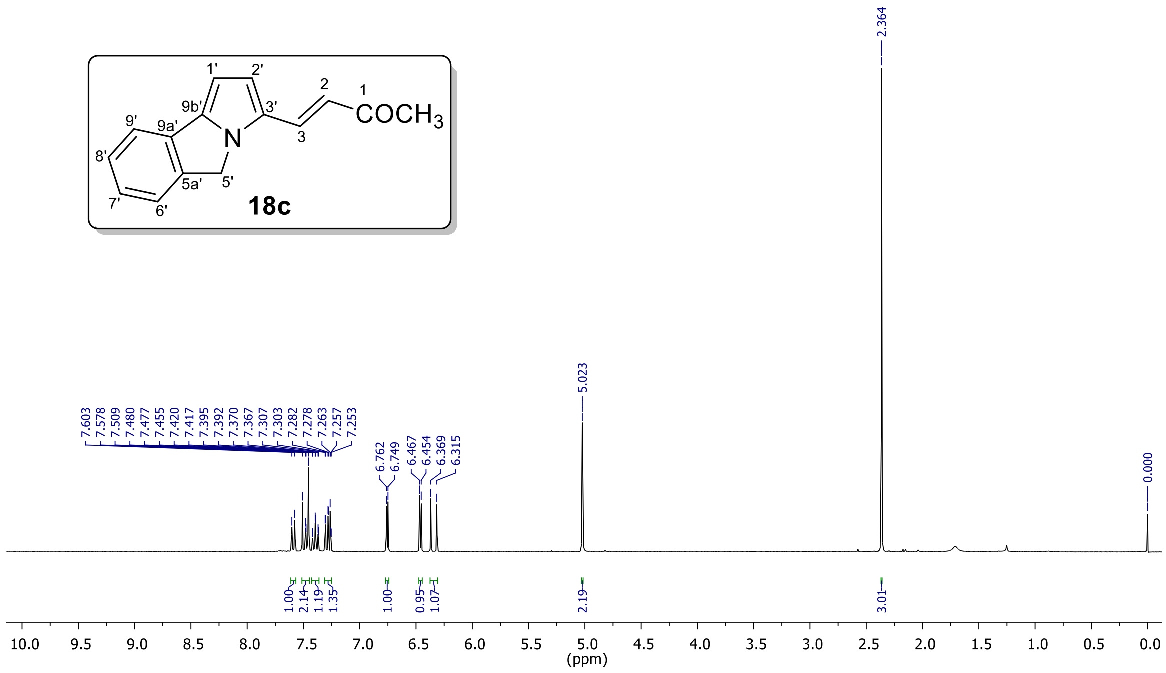

^{13}C NMR (75.4 MHz, $CDCl_3$)of compound **18c**.

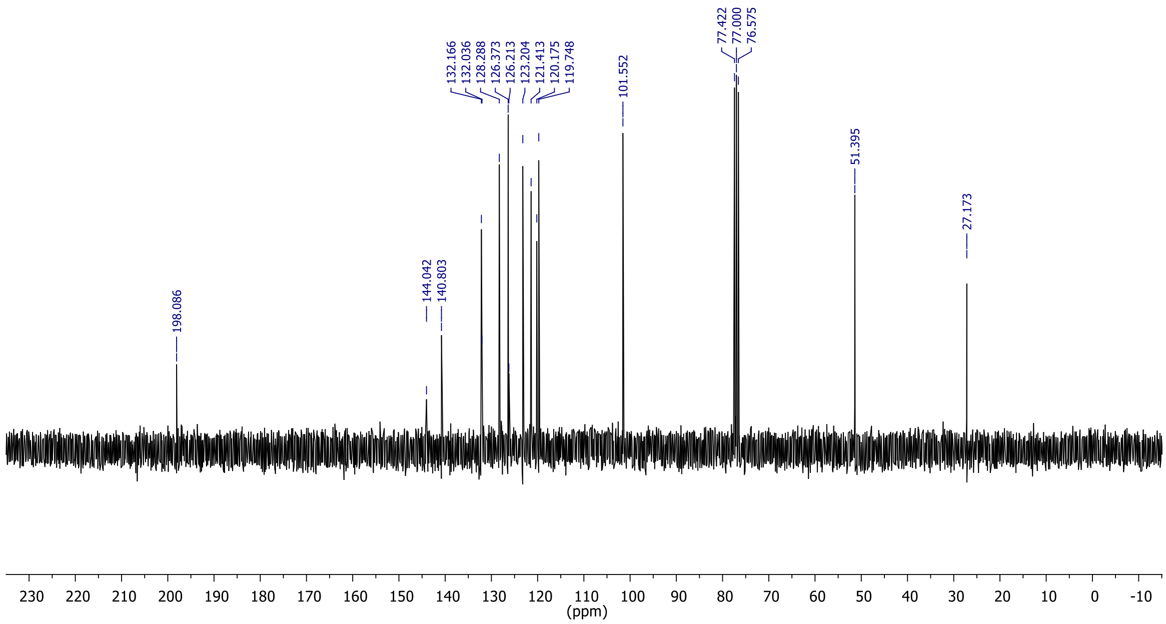

^{1}H NMR (300 MHz, $CDCl_3$) of compound **18d**.

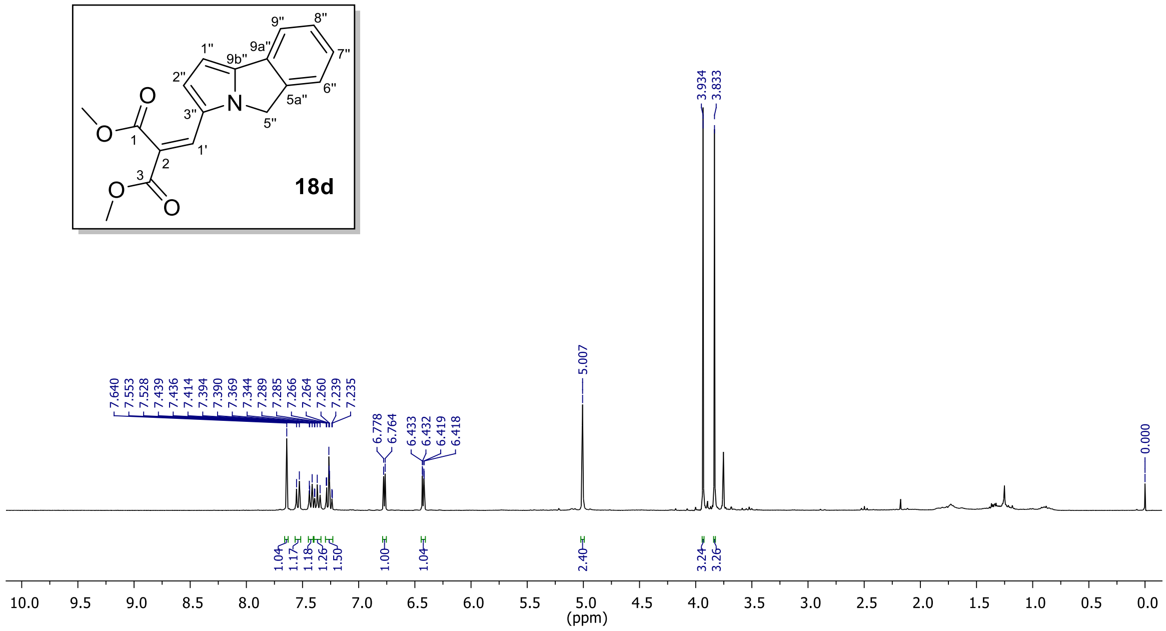

^{13}C NMR (75.4 MHz, $CDCl_3$) of compound **18d**.

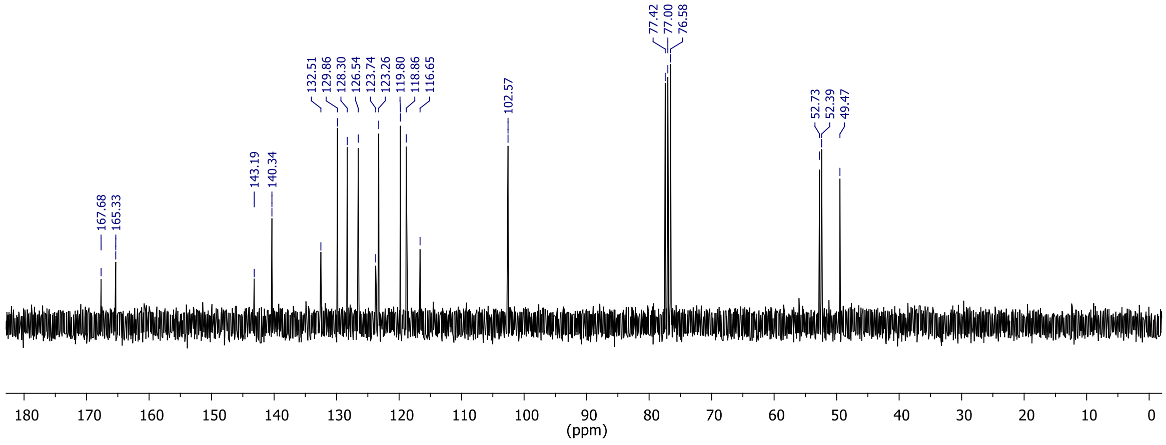

^{1}H NMR(300 MHz, $CDCl_3$) of compound **18e**.

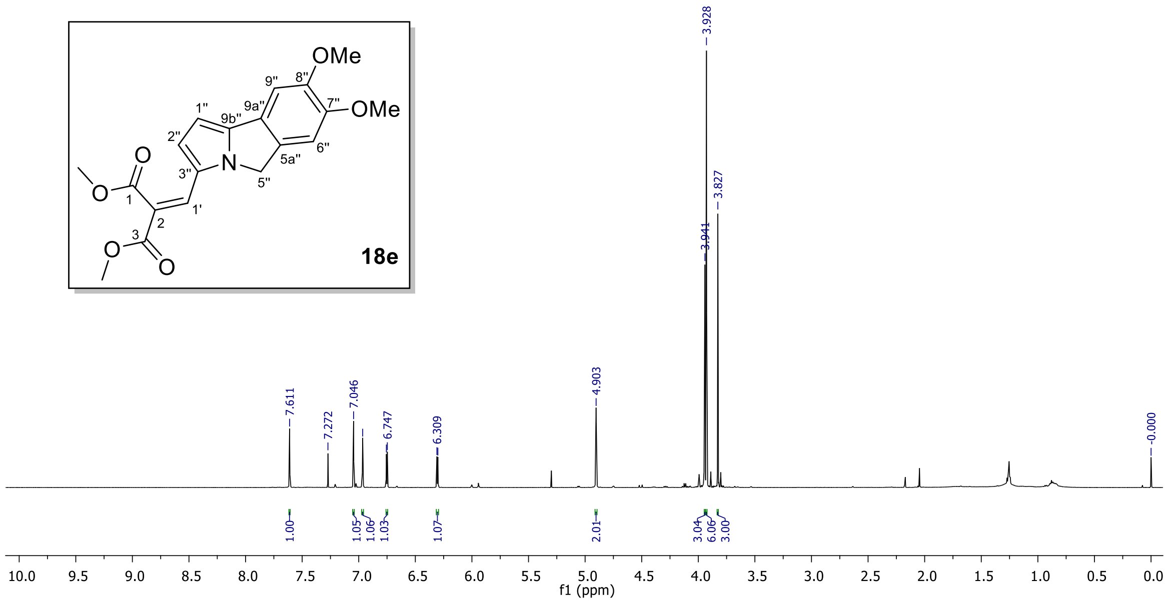

^{13}C NMR (74.5 MHz, $CDCl_3$) of compound **18e**.

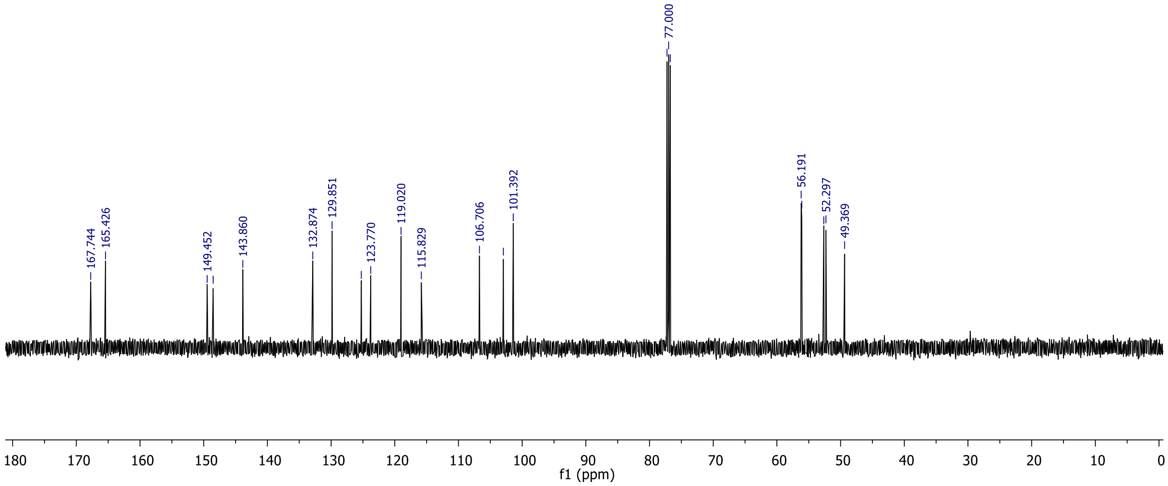

^1H NMR (600 MHz, $CDCl_3$) of compound **11a**.

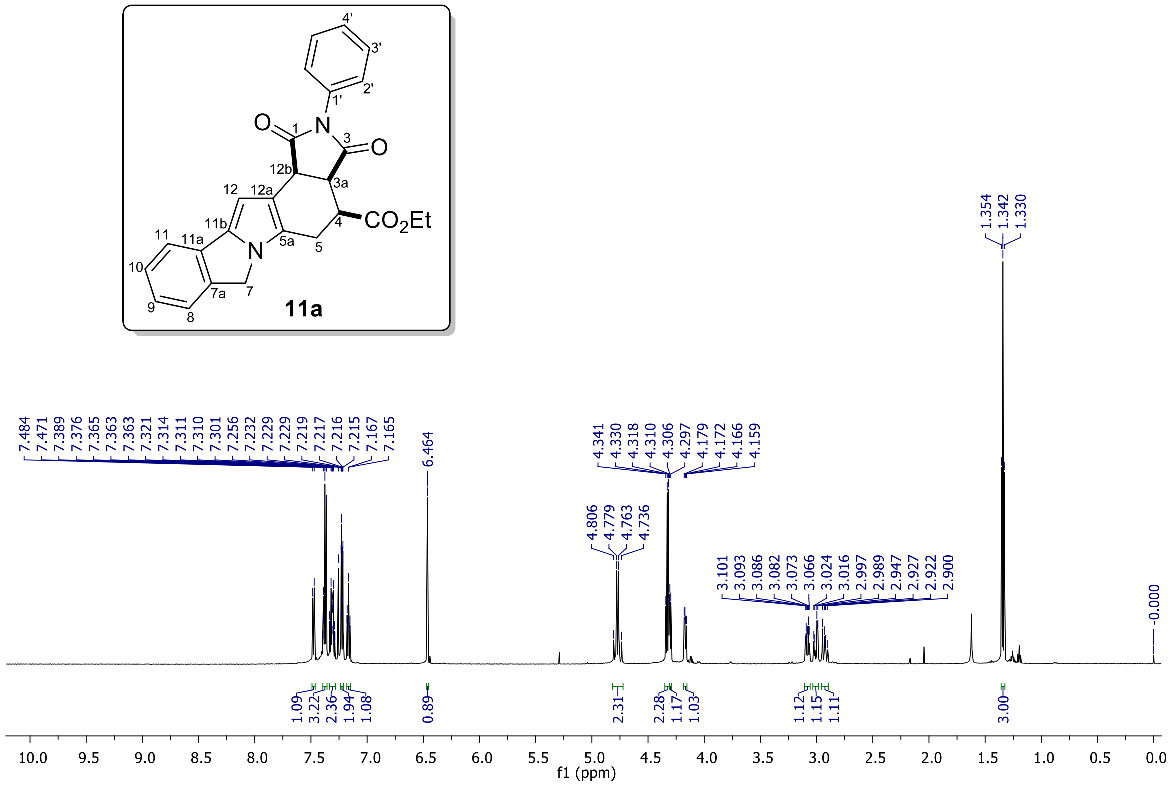

^{13}C NMR (150 MHz, $CDCl_3$) of compound **11a**.

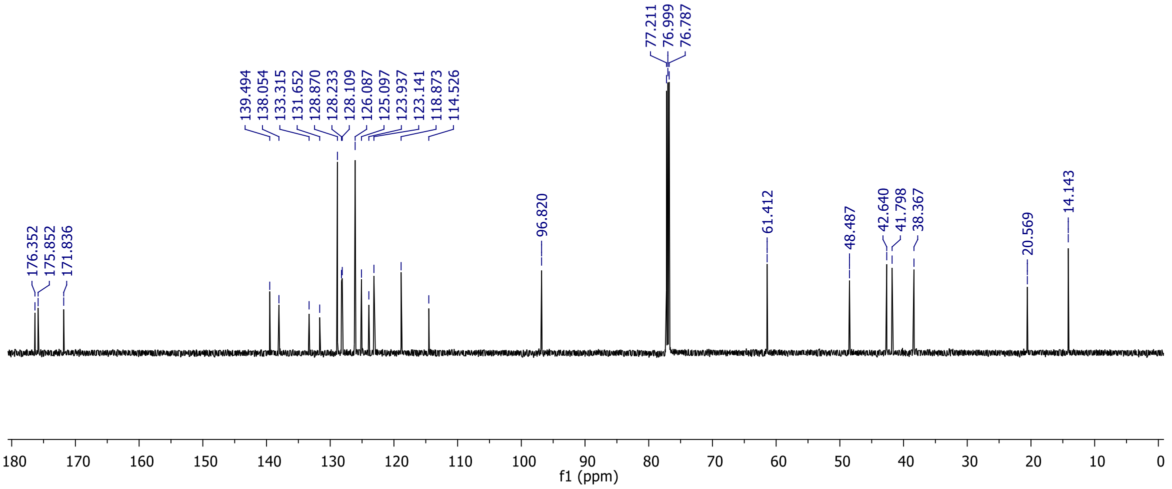

^{1}H NMR (500 MHz, $CDCl_3$) of compound **11b**.

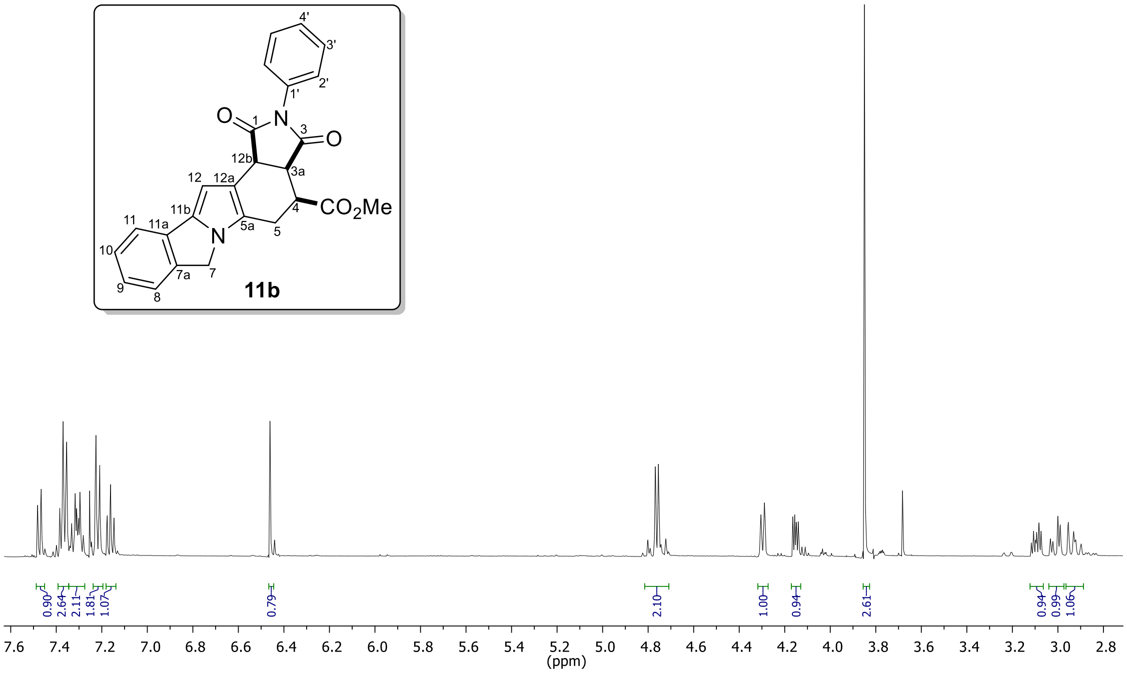

^{13}C NMR (125 MHz, $CDCl_3$) of compound **11b**.

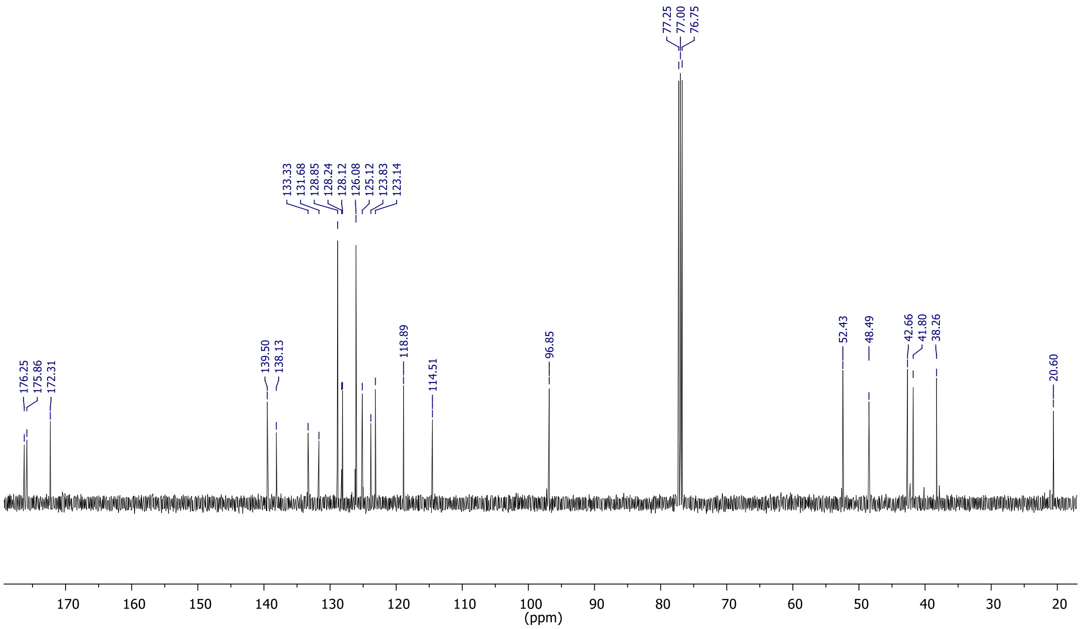

^{1}H NMR (600 MHz, $CDCl_3$) of compound **11c**.

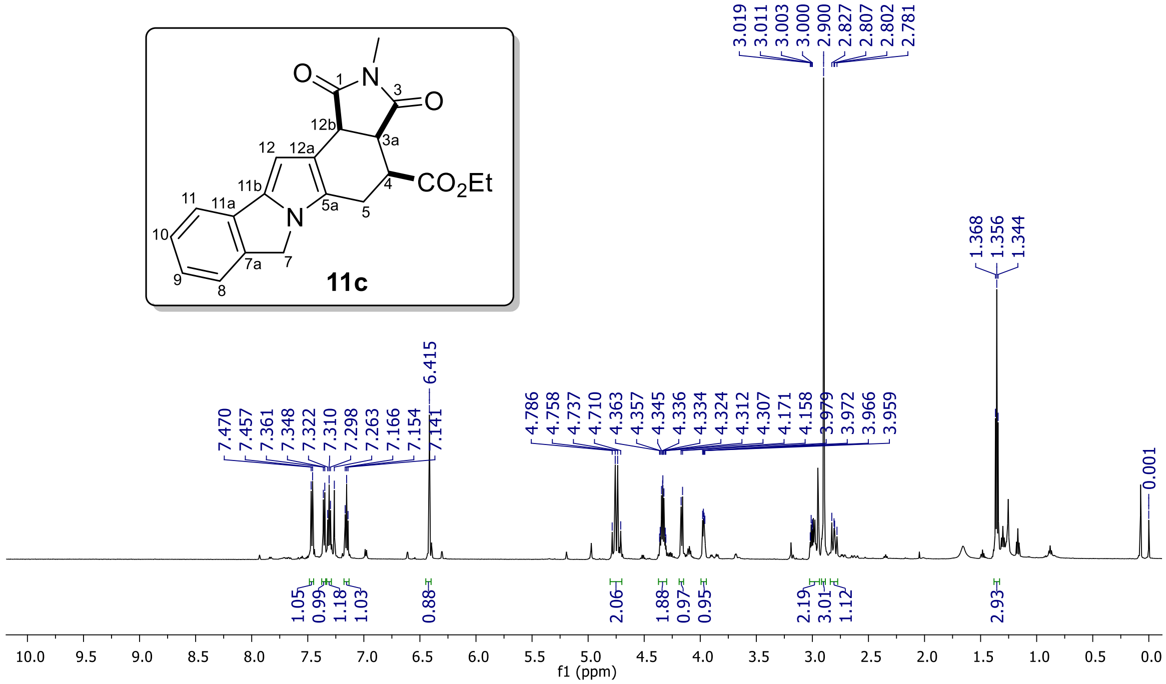

^{13}C NMR (150 MHz, $CDCl_3$) of compound **11c**.

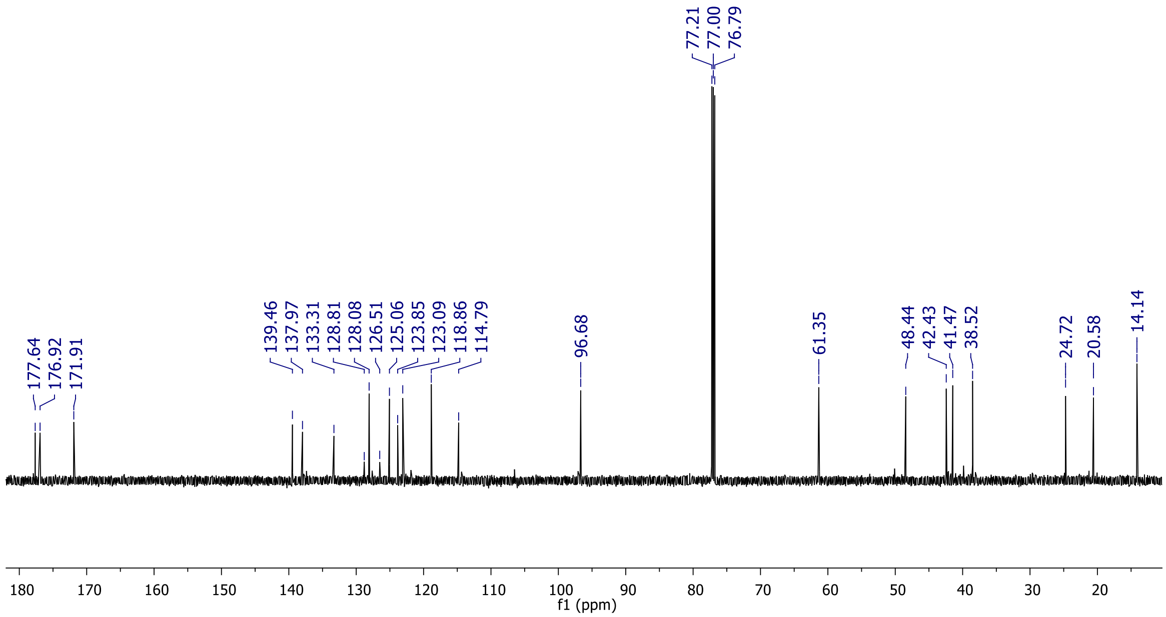

^{1}H NMR (600 MHz, $CDCl_3$) of compound **11d**.

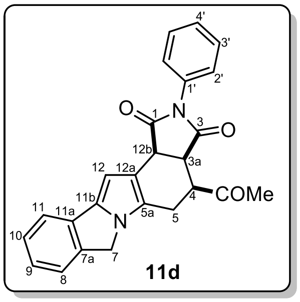

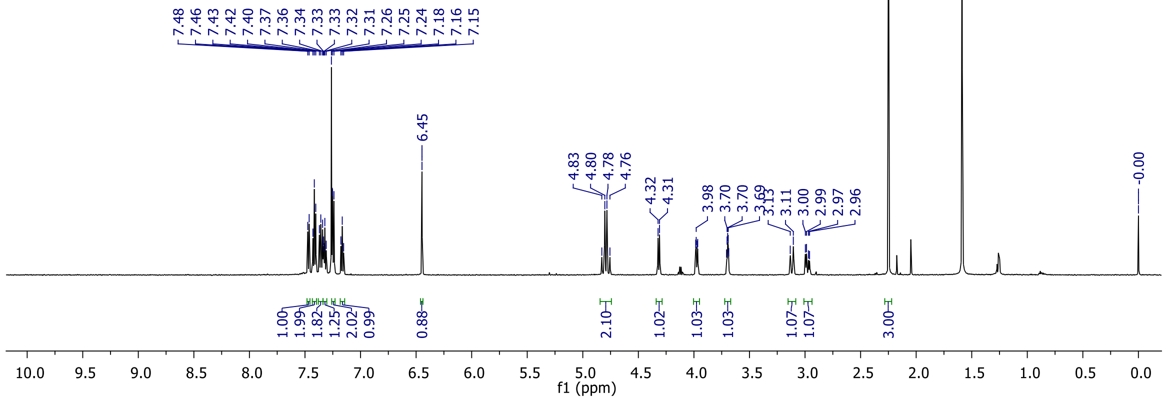

^{13}C NMR (150 MHz, $CDCl_3$) of compound **11d**.

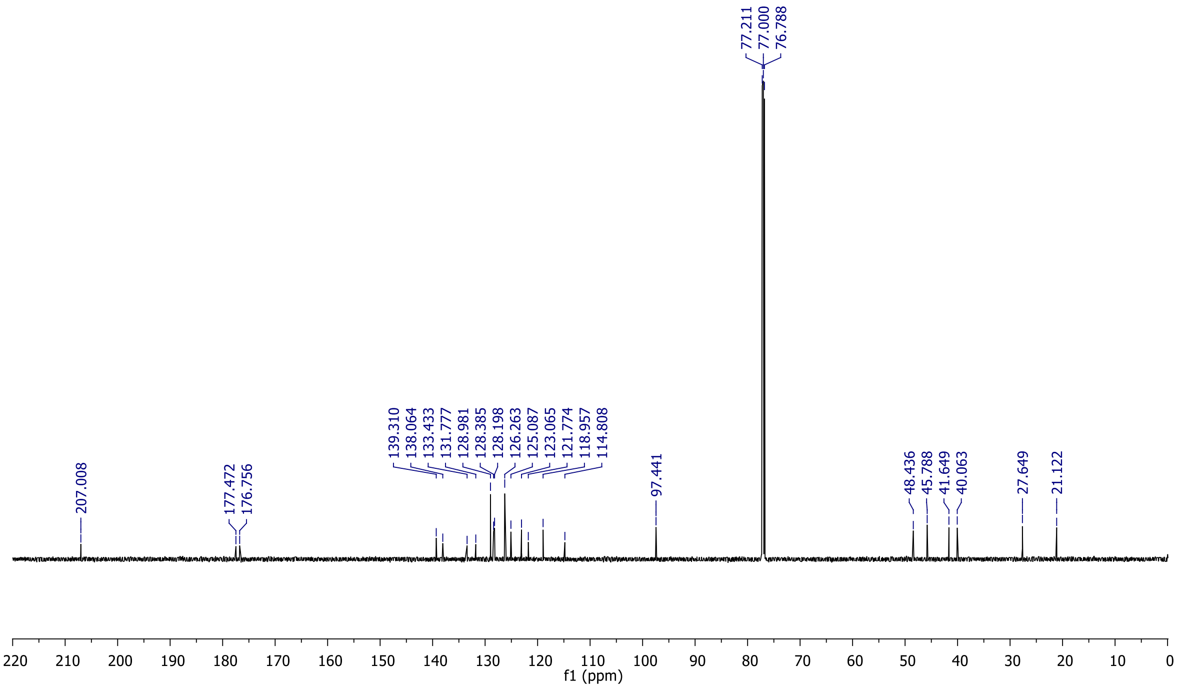

^{1}H NMR (500 MHz, $CDCl_3$) of compound **11e**.

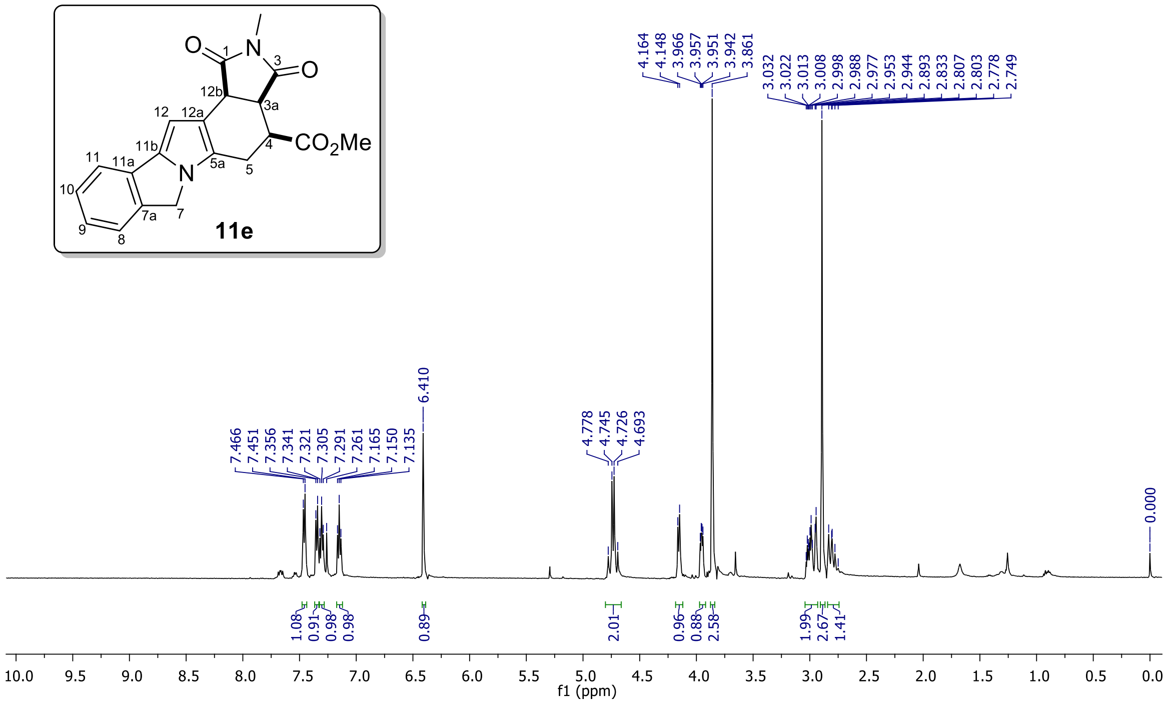

^{13}C NMR(125 MHz, $CDCl_3$) of compound **11e**.

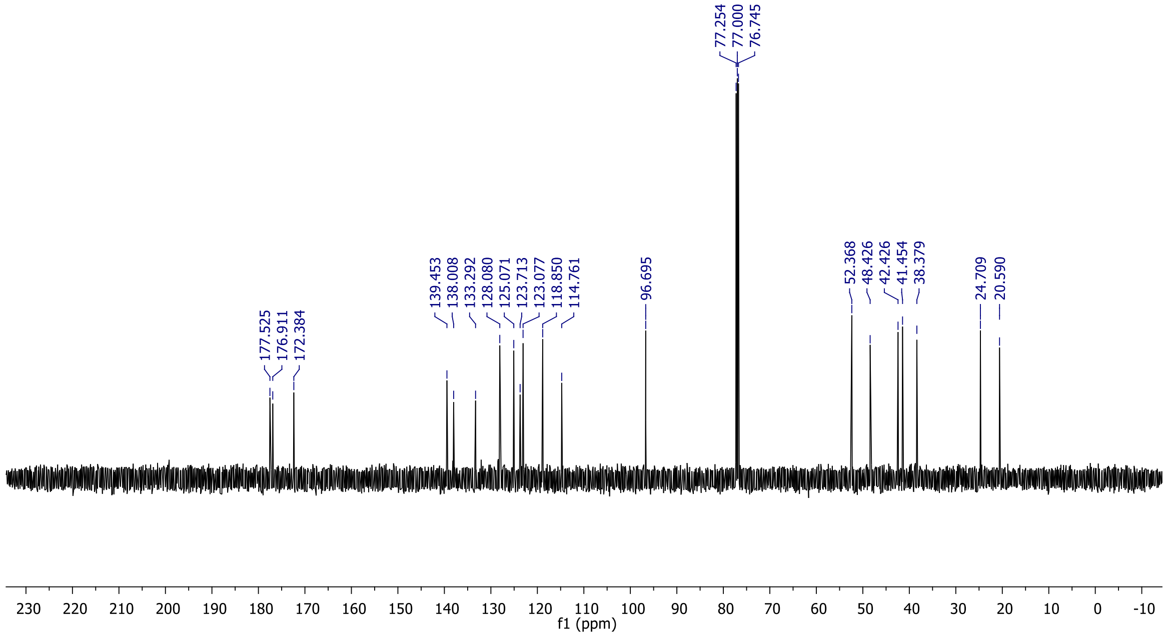

^{1}H NMR (500 MHz, $CDCl_3$) of compound **21a**.

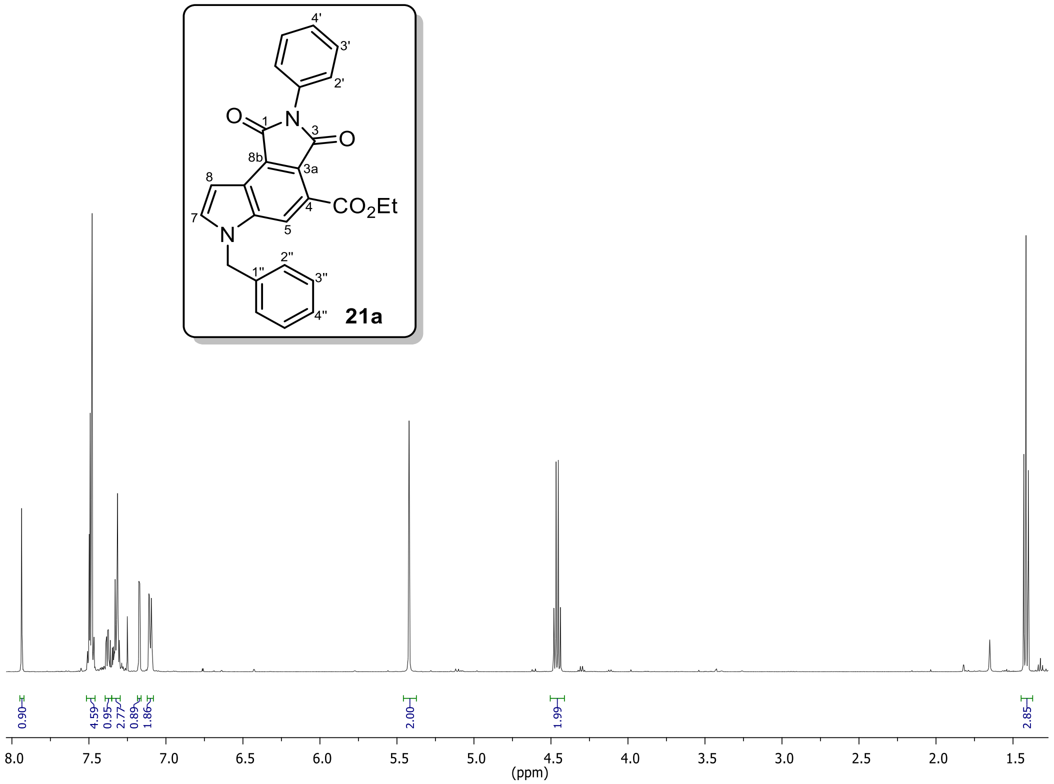

^{13}C NMR (125 MHz, $CDCl_3$) of compound **21a**.

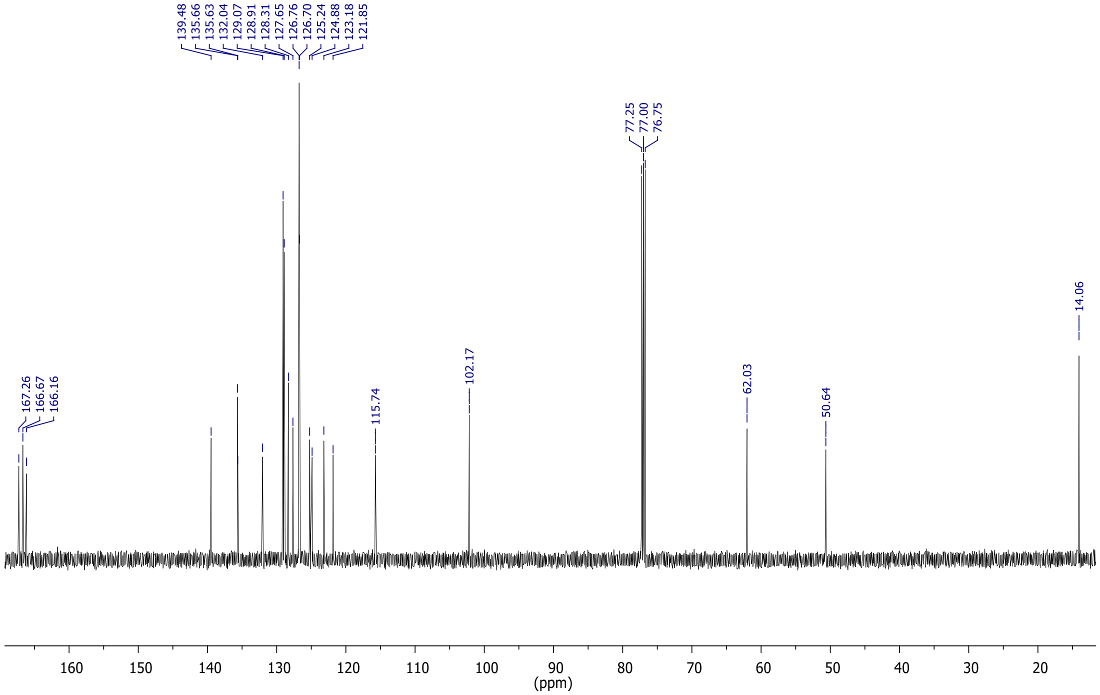

^1H NMR (500 MHz, DMSO-d_6) of compound **21b**.

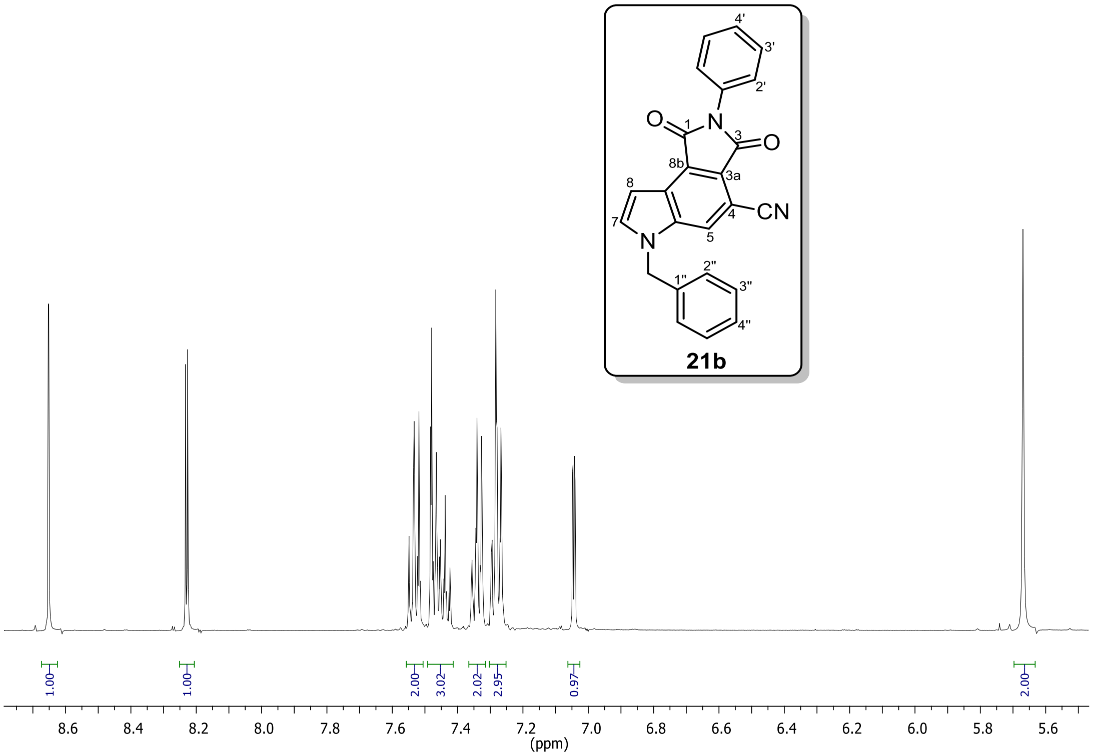

^{13}C NMR (125 MHz, DMSO-d_6) of compound **21b**.

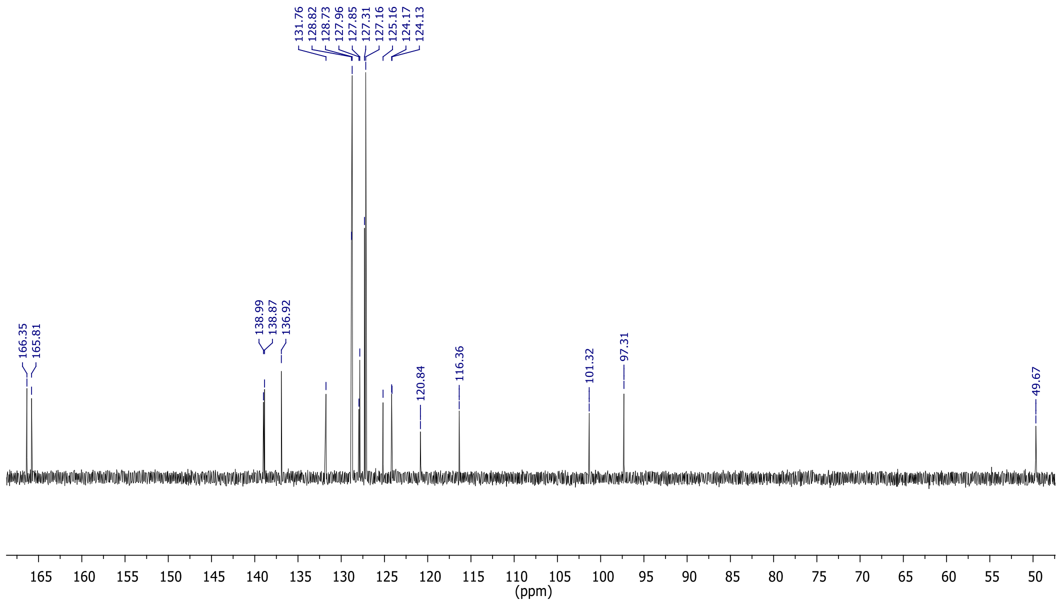

^{1}H NMR (500 MHz, $CDCl_3$) of compound **21c**.

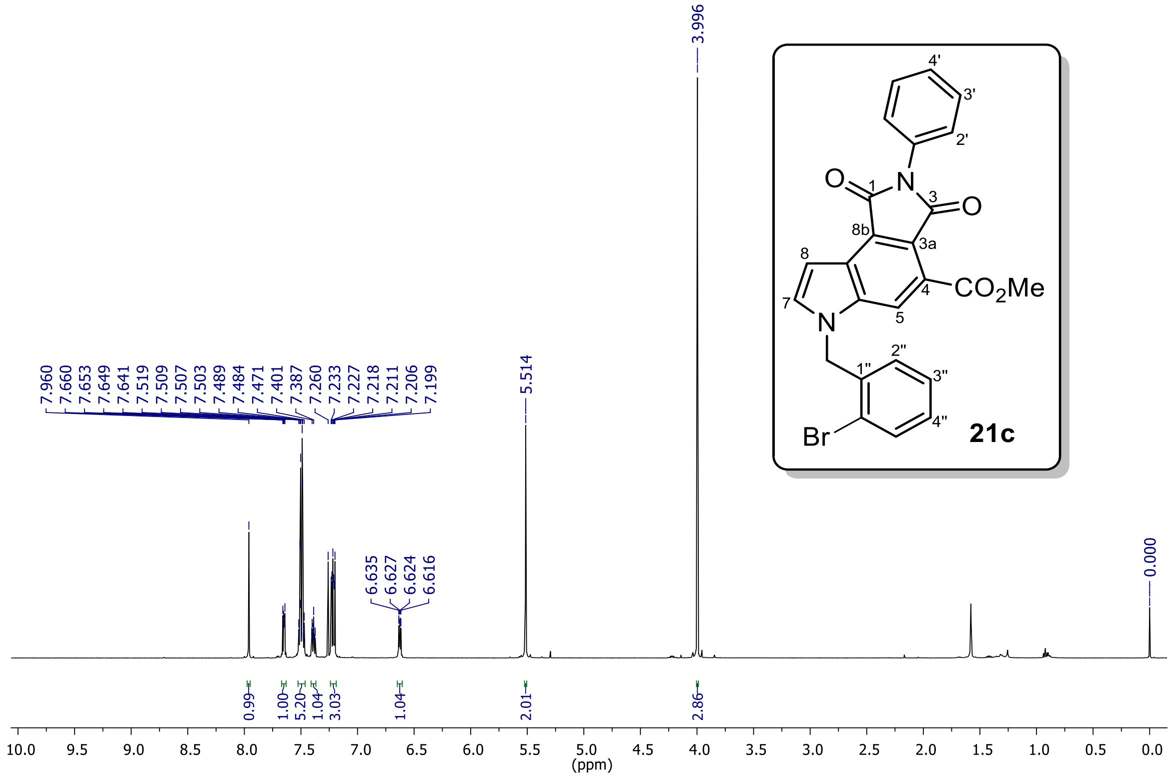

^{13}C NMR (125 MHz, $CDCl_3$) of compound **21c**.

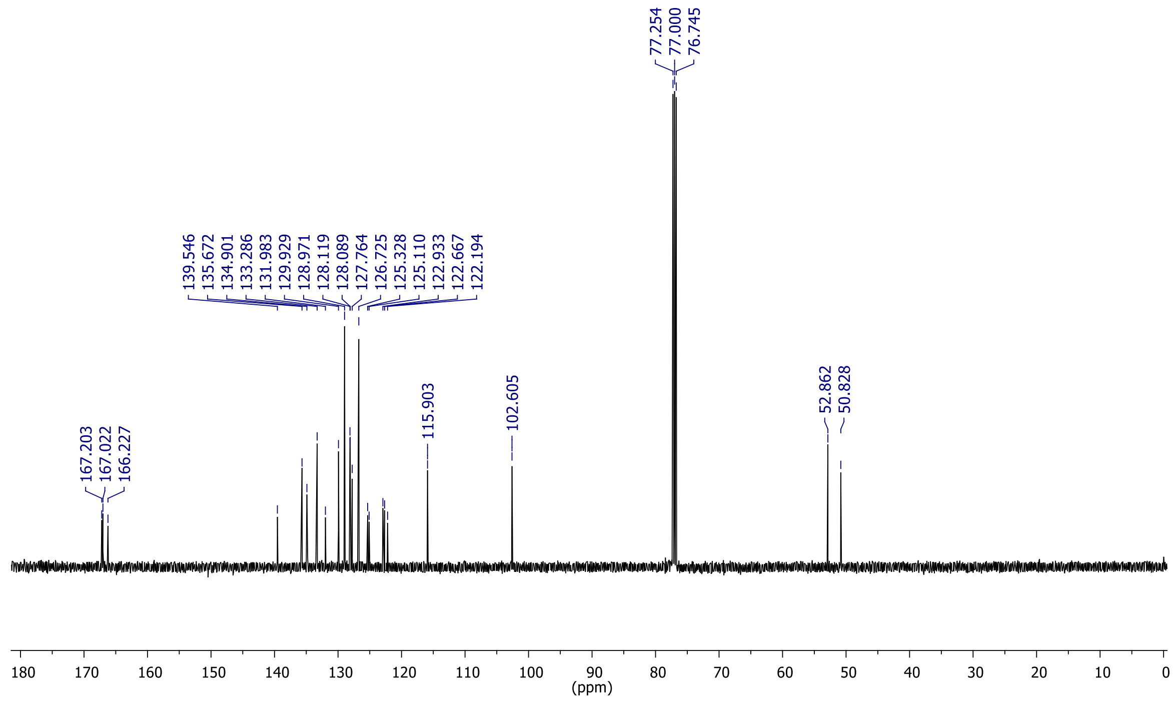

^{1}H NMR (500 MHz, $CDCl_3$) of compound **21d**.

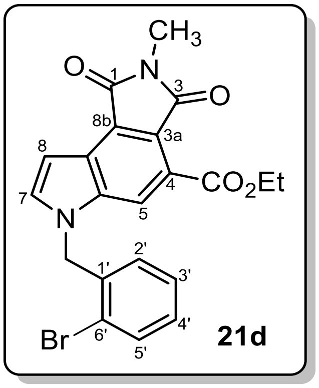

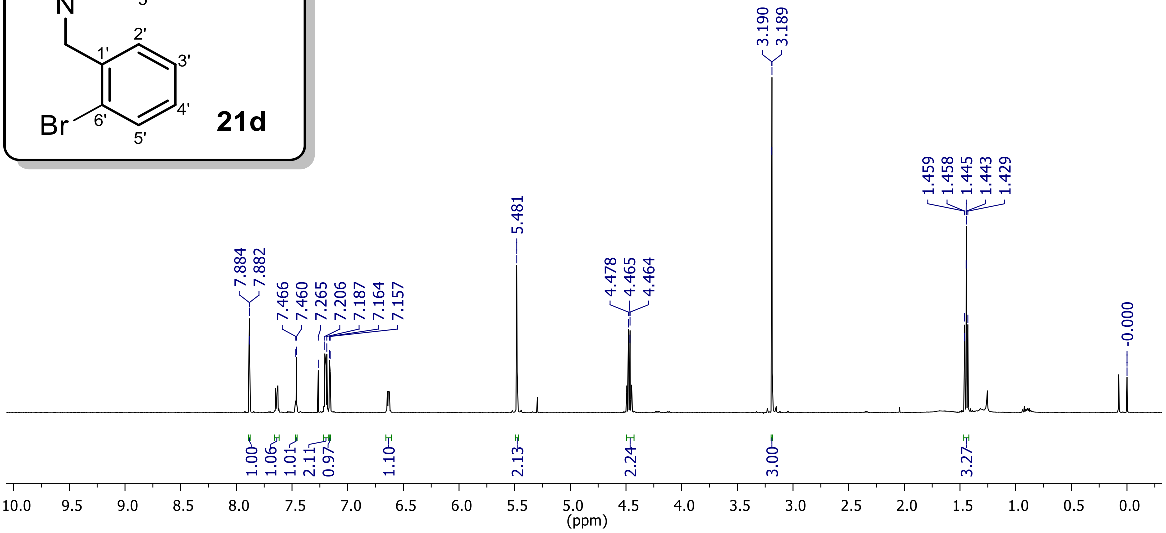

^{13}C NMR (125 MHz, $CDCl_3$) of compound **21d**.

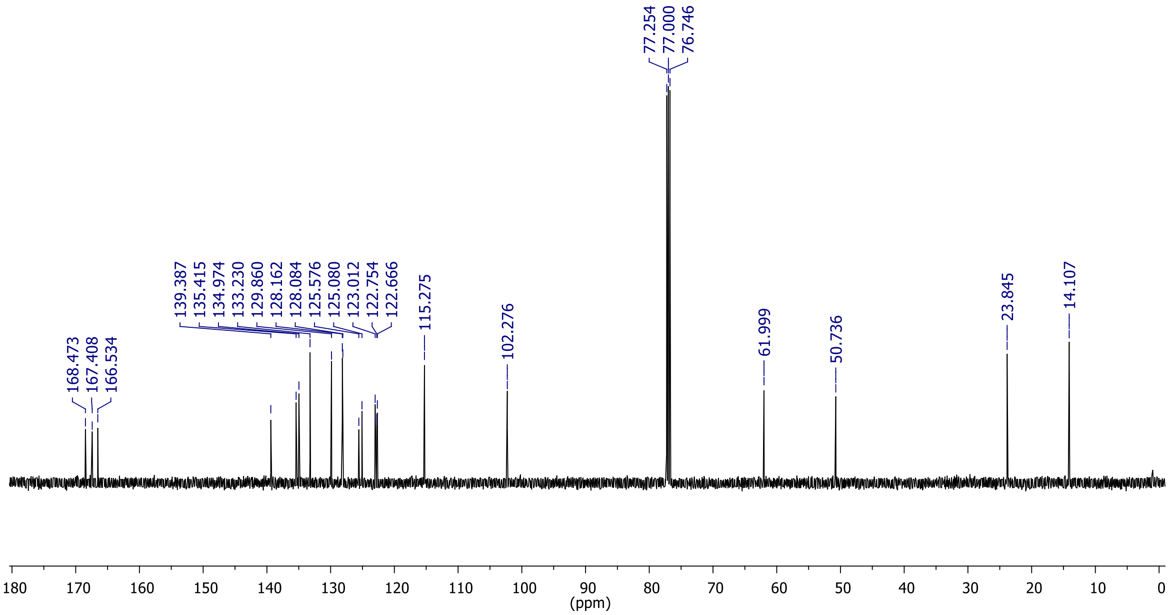

^{1}H NMR (500 MHz, $CDCl_3$) of compound **21e**.

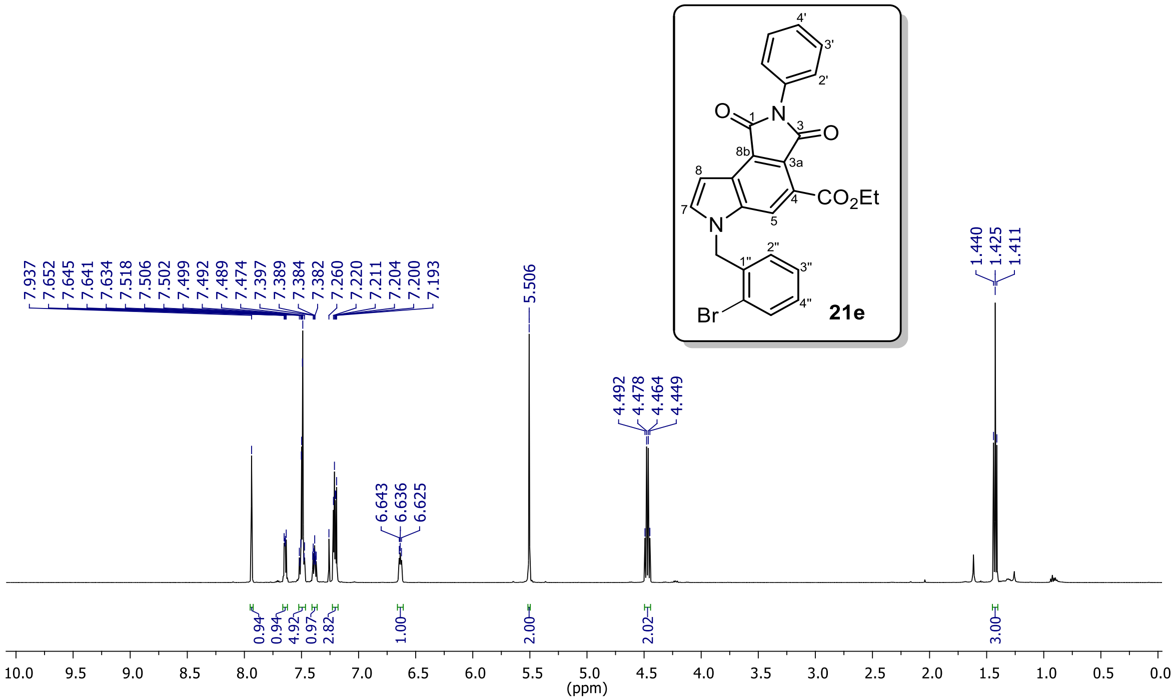

^{13}C NMR (125 MHz, $CDCl_3$) of compound **21e**.

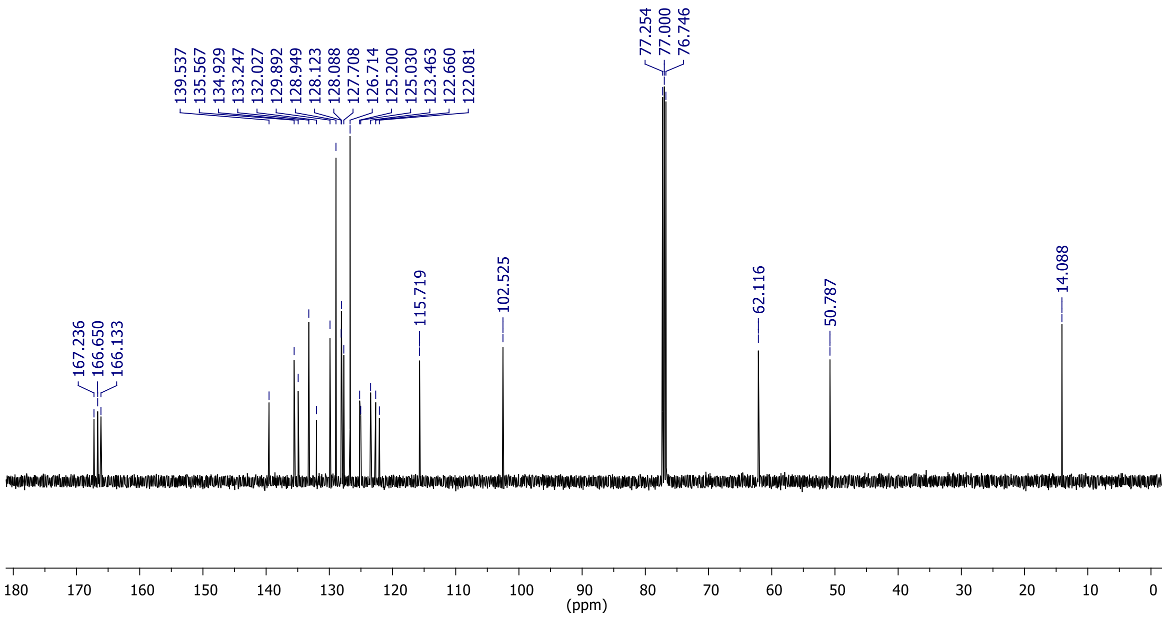

^{1}H NMR (300 MHz, $CDCl_3$) of compound **21f**.

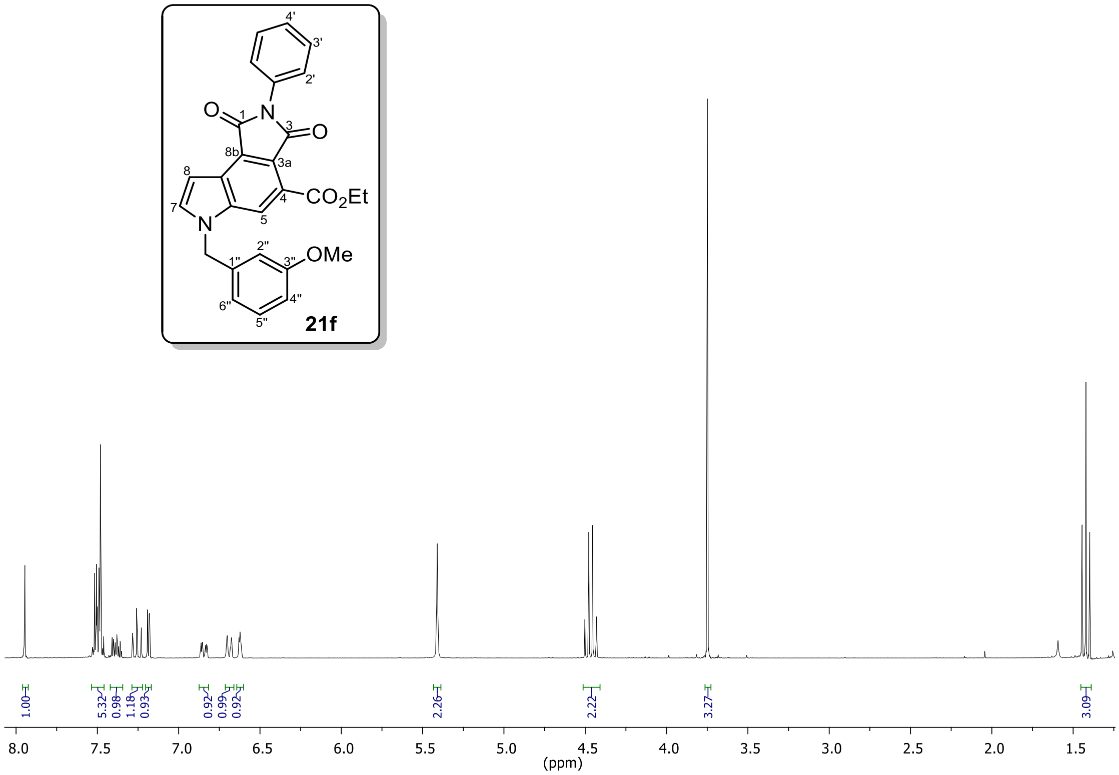

^{13}C NMR (75.4 MHz, $CDCl_3$) of compound **21f**.

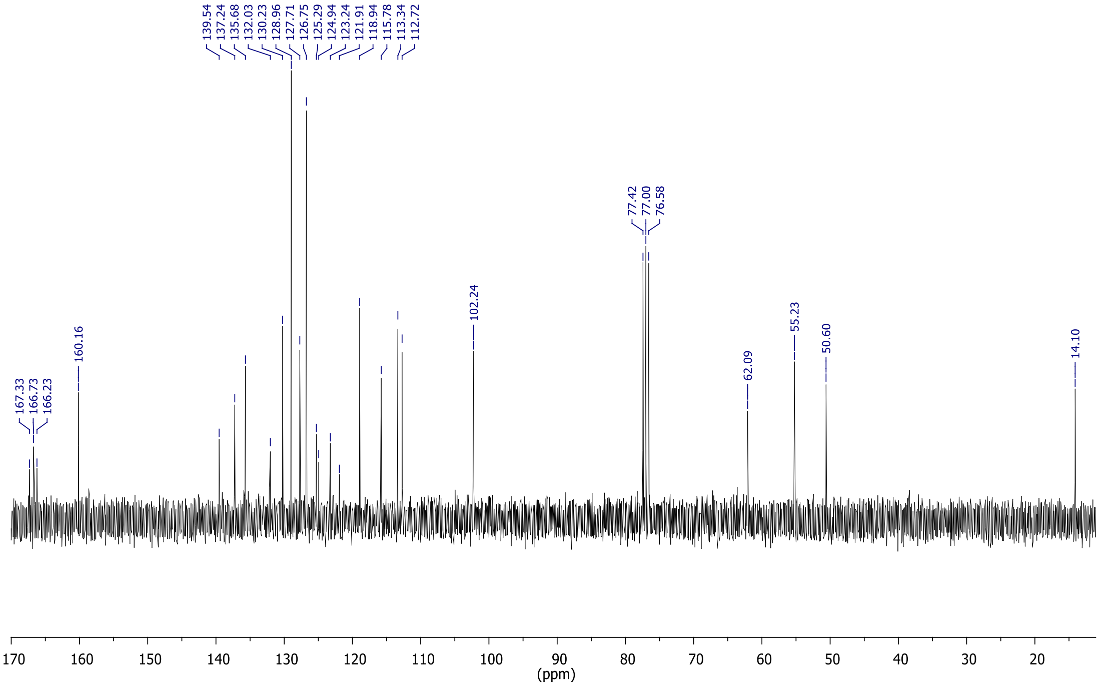

^{1}H NMR (500 MHz, $CDCl_3$) of compound **21g**.

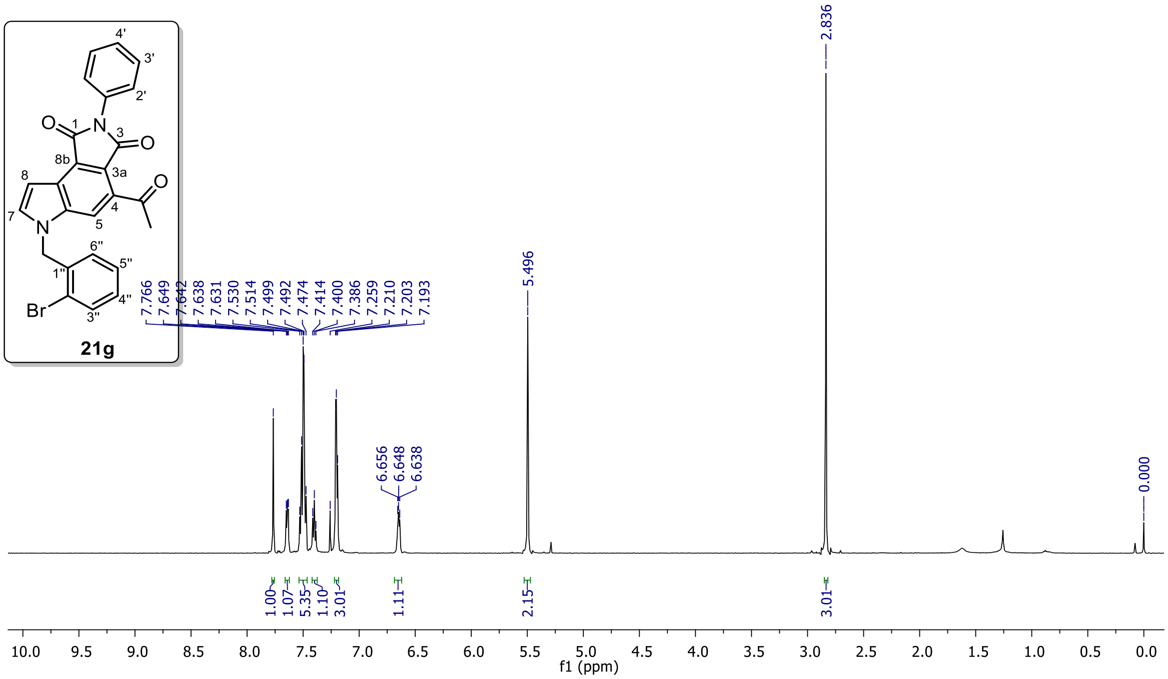

^{13}C NMR (125 MHz, $CDCl_3$) of compound **21g**.

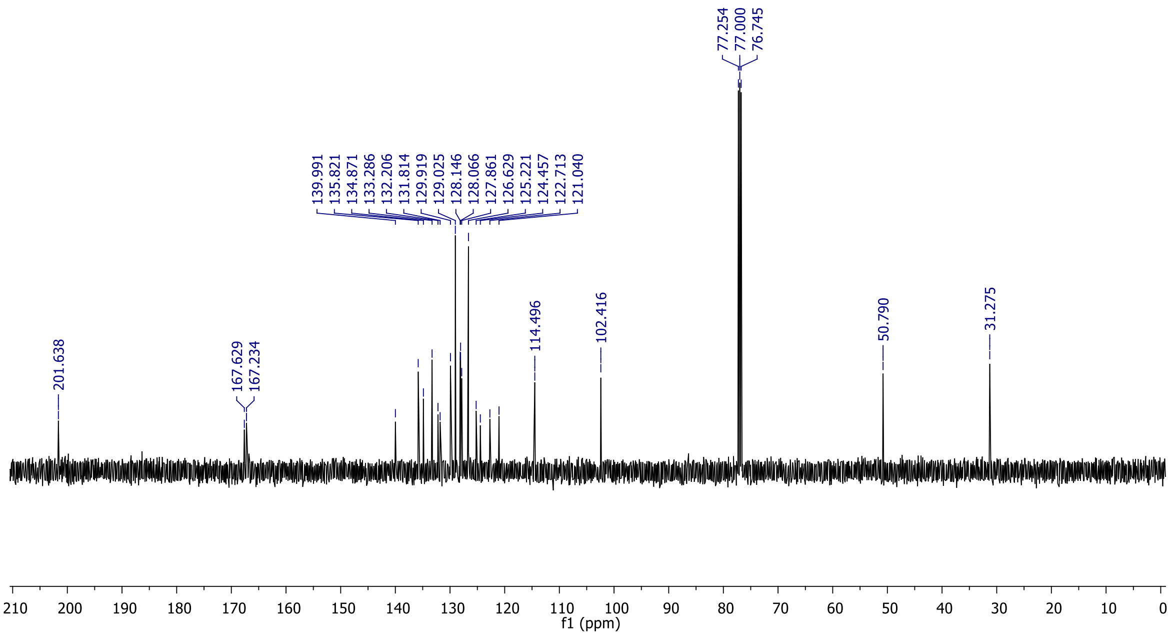

^{1}H NMR (300 MHz, $CDCl_3$) of compound **22a**.

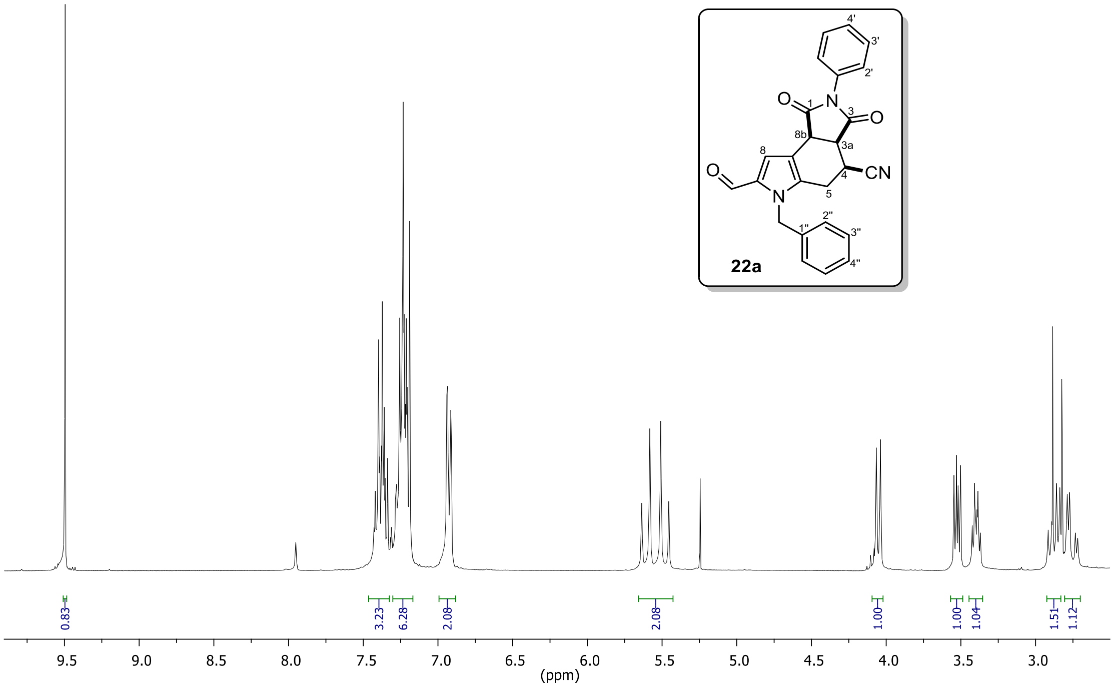

^{13}C NMR (75.4 MHz, $CDCl_3$) of compound **22a**.

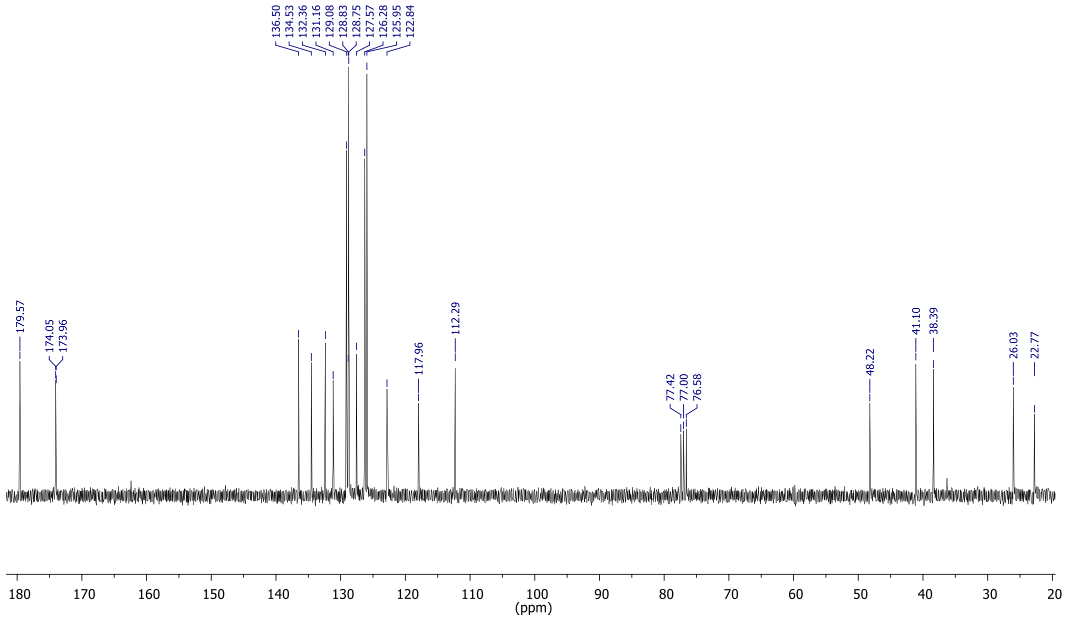

^{1}H NMR (500 MHz, $CDCl_3$) of compound **22b**.

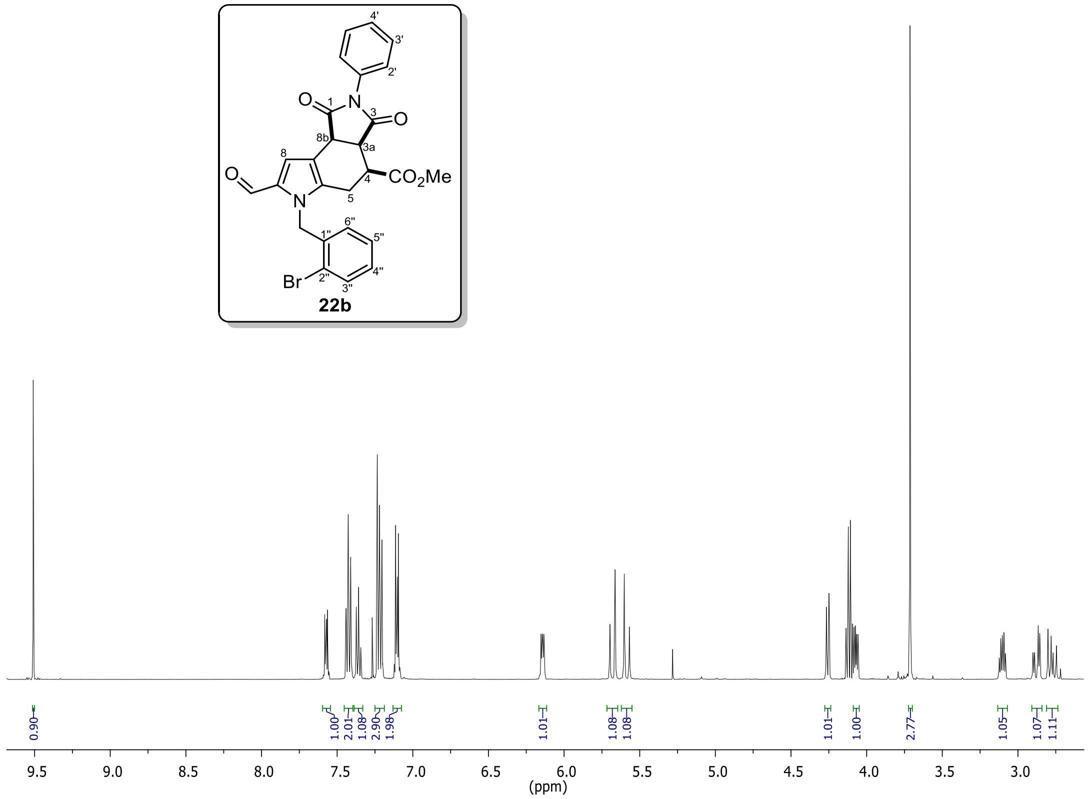

^{13}C NMR (125 MHz, $CDCl_3$) of compound **22b**.

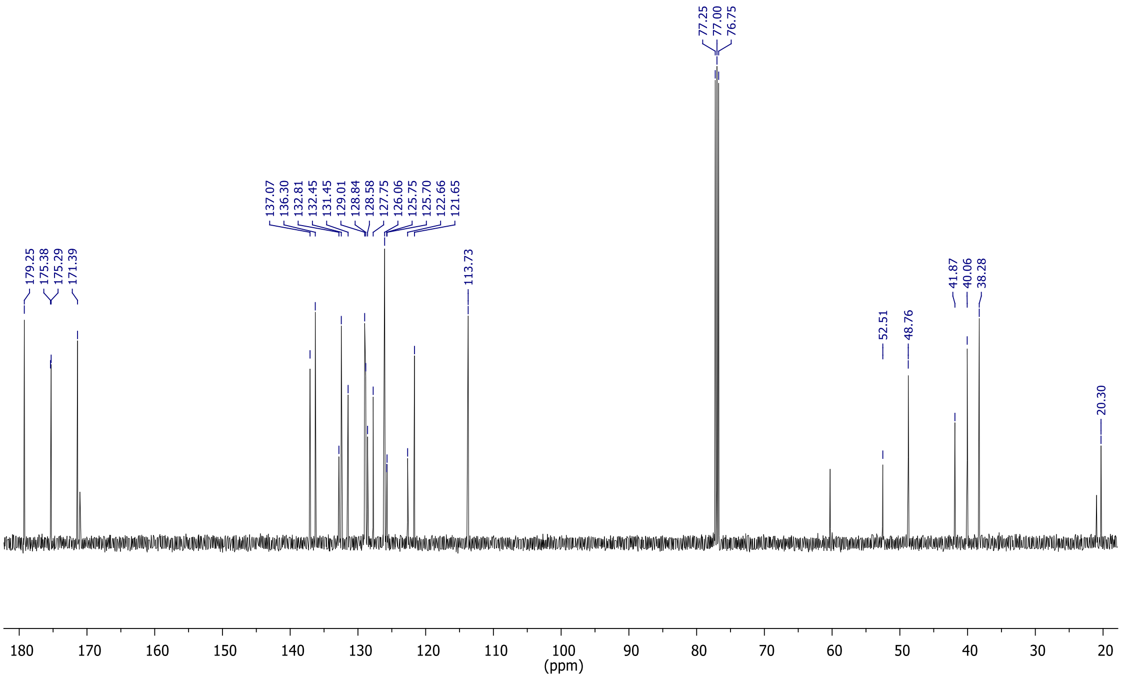

^{1}H NMR (500 MHz, $CDCl_3$) of compound **22c**.

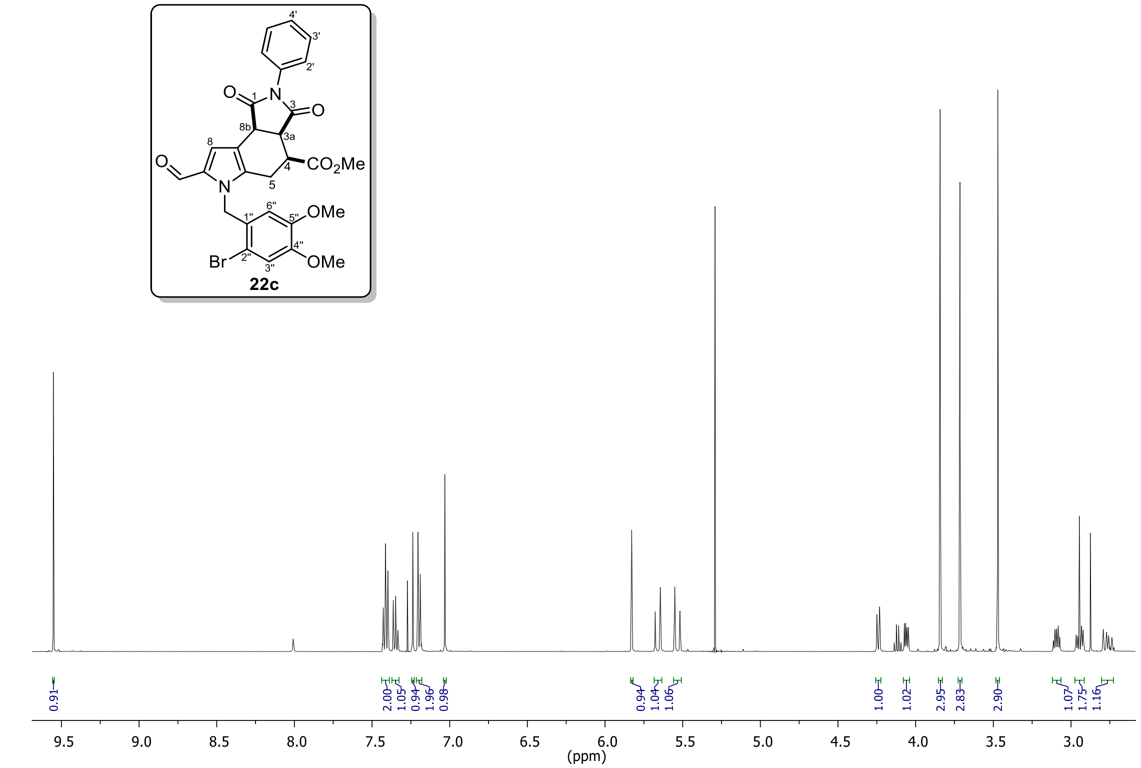

^{13}C NMR (125 MHz, $CDCl_3$) of compound **22c**.

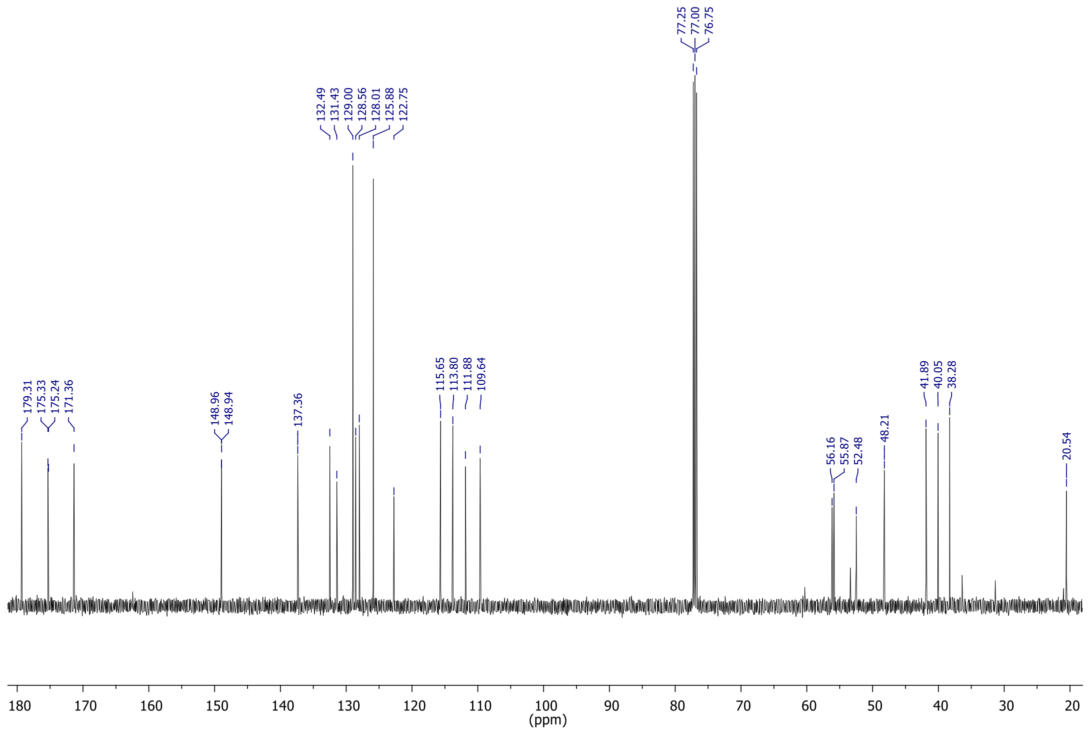

^{1}H NMR (500 MHz, $CDCl_3$) of compound **22d**.

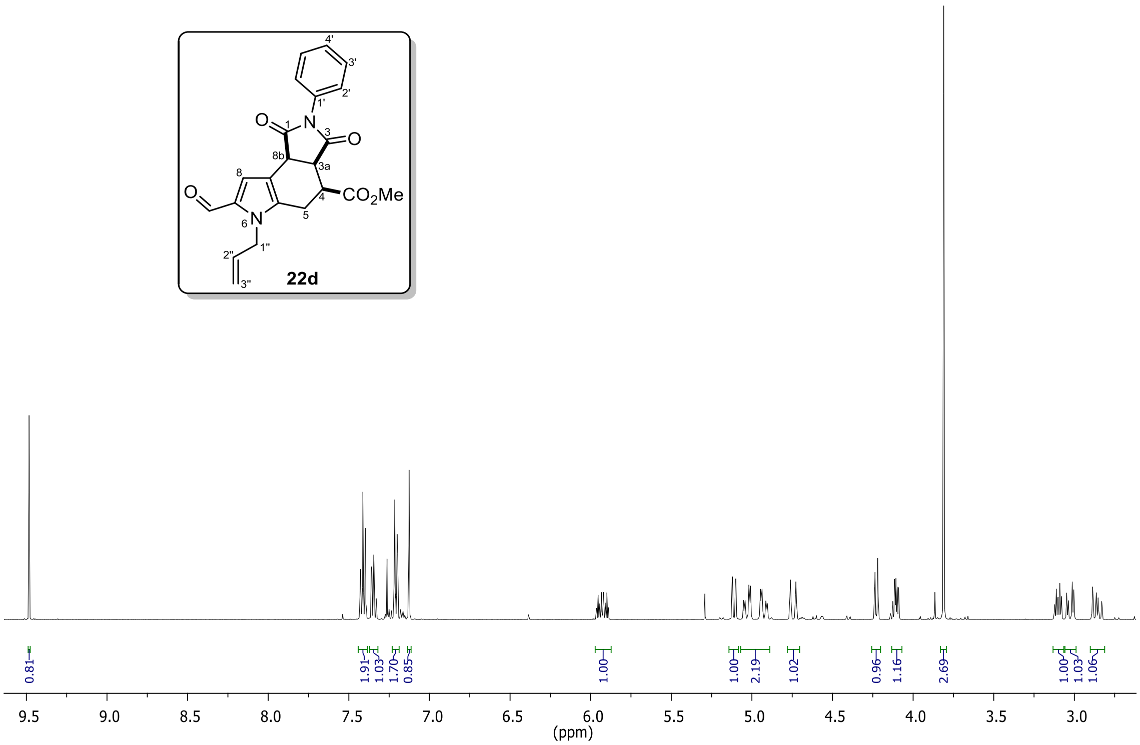

^{13}C NMR (125 MHz, $CDCl_3$) of compound **22d**.

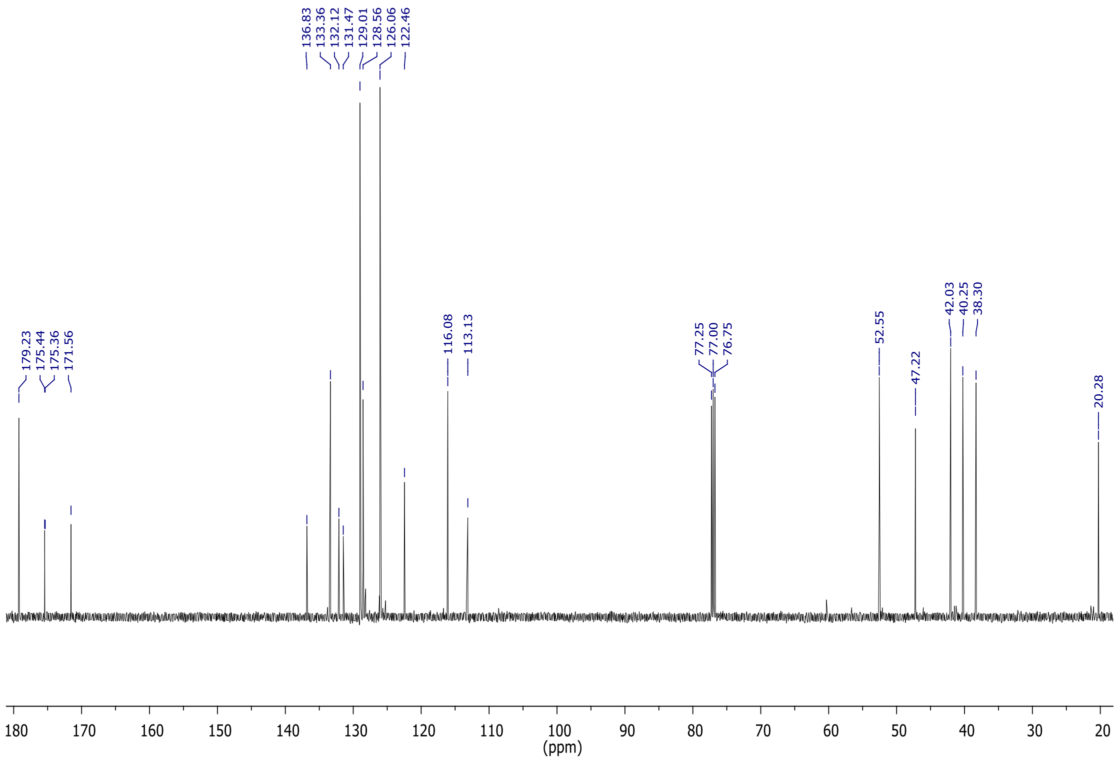

^{1}H NMR (300 MHz, $CDCl_3$) of compound **22e**.

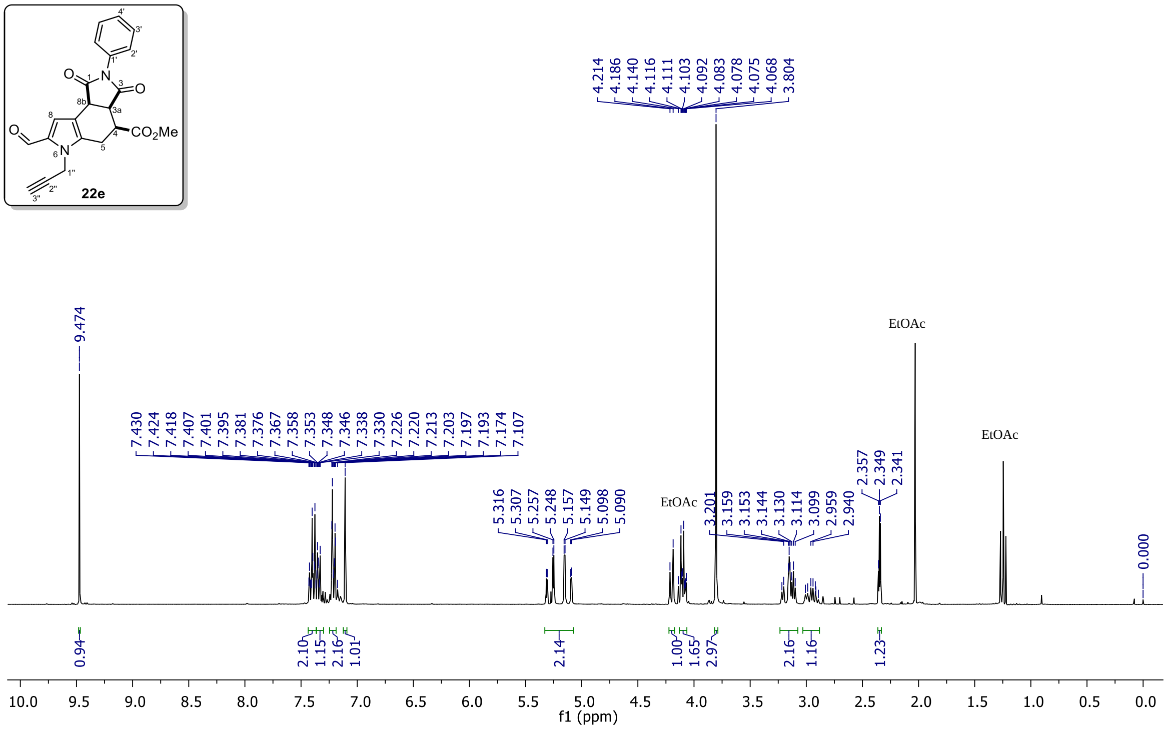

^{13}C NMR (75.4 MHz, $CDCl_3$) of compound **22e**.

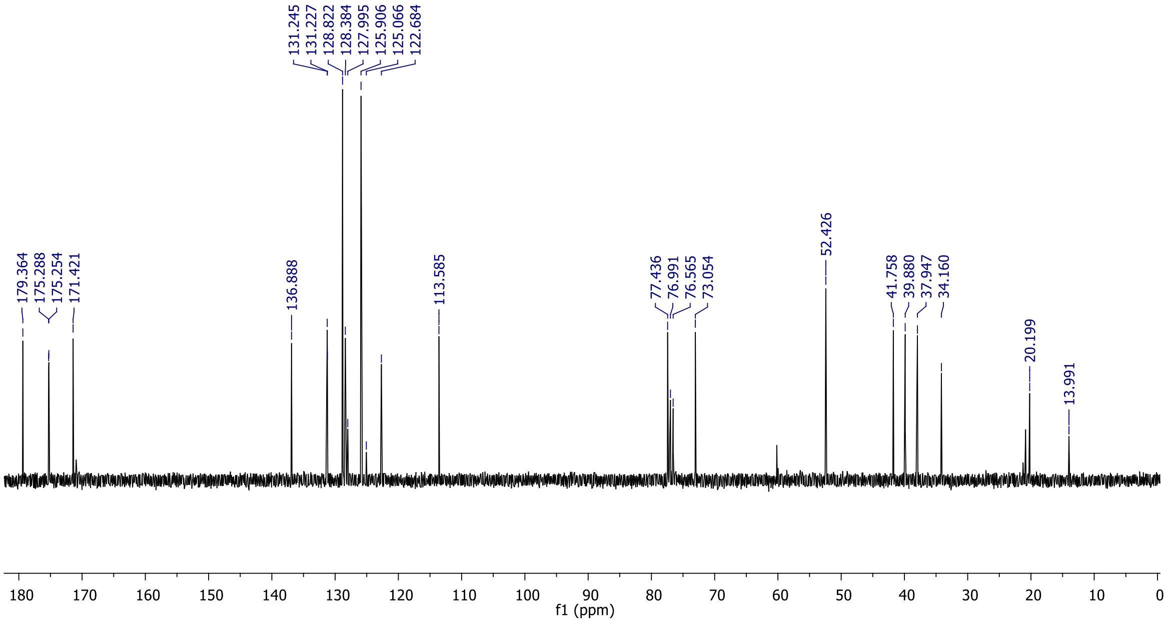

^{1}H NMR (500 MHz, CD_2Cl_2/acetone-d_6/$CDCl_3$, 80:13:7) of compound **12**.

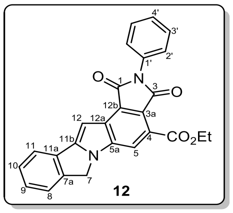

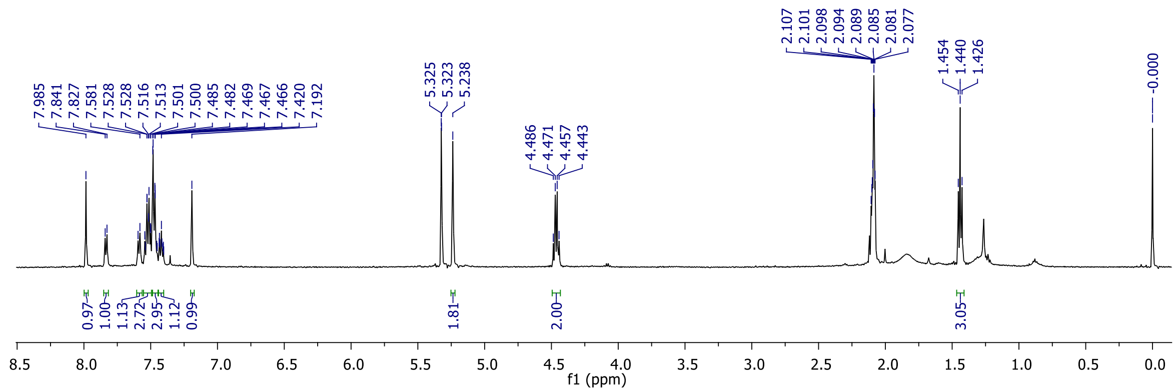

^{13}C NMR (500 MHz, CD_2Cl_2/acetone-d_6/$CDCl_3$, 80:13:7) of compound **12**.

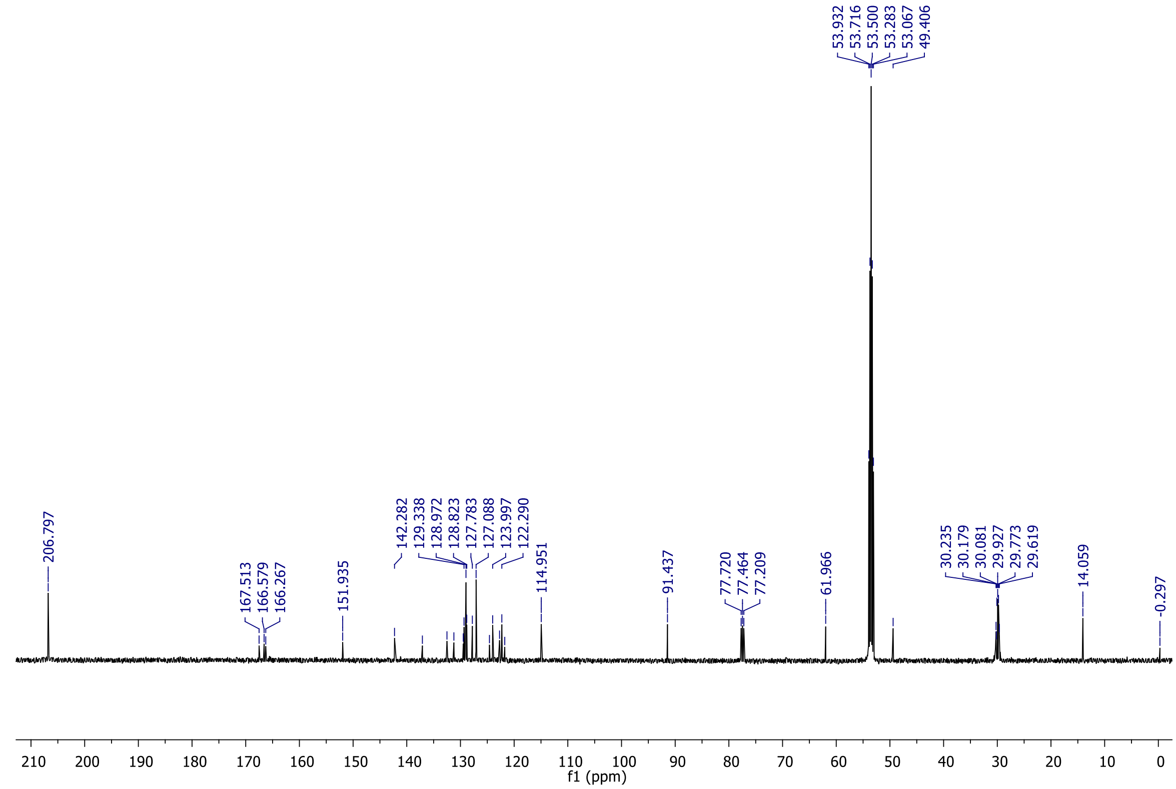

Supplement: File 1 — Experimental and analytical data, X-ray crystallographic structures, NMR-spectra and all calculated data. [file Beilstein_J_Org_Chem-16-1320-s001.pdf]
